# Sulfonylative and Azidosulfonylative Cyclizations by Visible‐Light‐Photosensitization of Sulfonyl Azides in THF

**DOI:** 10.1002/chem.201704380

**Published:** 2017-11-16

**Authors:** Shaoqun Zhu, Atchutarao Pathigoolla, Grace Lowe, Darren A. Walsh, Mick Cooper, William Lewis, Hon Wai Lam

**Affiliations:** ^1^ The GSK Carbon Neutral Laboratories for Sustainable Chemistry University of Nottingham, Jubilee Campus Triumph Road Nottingham NG7 2TU UK; ^2^ School of Chemistry University of Nottingham University Park Nottingham NG7 2RD UK

**Keywords:** azides, cyclization, iridium, photocatalysis, radical reactions

## Abstract

The generation of sulfonyl radicals from sulfonyl azides using visible light and a photoactive iridium complex in THF is described. This process was used to promote sulfonylative and azidosulfonylative cyclizations of enynes to give several classes of highly functionalized heterocycles. The use of THF as the solvent is critical for successful reactions. The proposed mechanism of radical initiation involves the photosensitized formation of a triplet sulfonyl nitrene, which abstracts a hydrogen atom from THF to give a tetrahydrofuran‐2‐yl radical, which then reacts with the sulfonyl azide to generate the sulfonyl radical.

## Introduction

Azides are highly versatile functional groups because they undergo many different reactions.^1^, ^2^ The recent, dramatic increase in the use of visible light photocatalysis in synthesis^3^ has led to its application in reactions of organic azides, resulting in several interesting new processes.^4^ Aryl,4a–4c,4i alkyl,4a alkenyl,4c and acyl4d,4j azides, as well as azidoformates4f have been employed in reductions,4a radical additions to nitriles,4a nitrene insertions,4b,4d rearrangements,4c aziridinations,4c,4f enantioselective enolate aminations,4i and cascade cyclizations.4j Azidoiodanes have also been used in radical azidations.4e,4g,4h

**Scheme 1:**
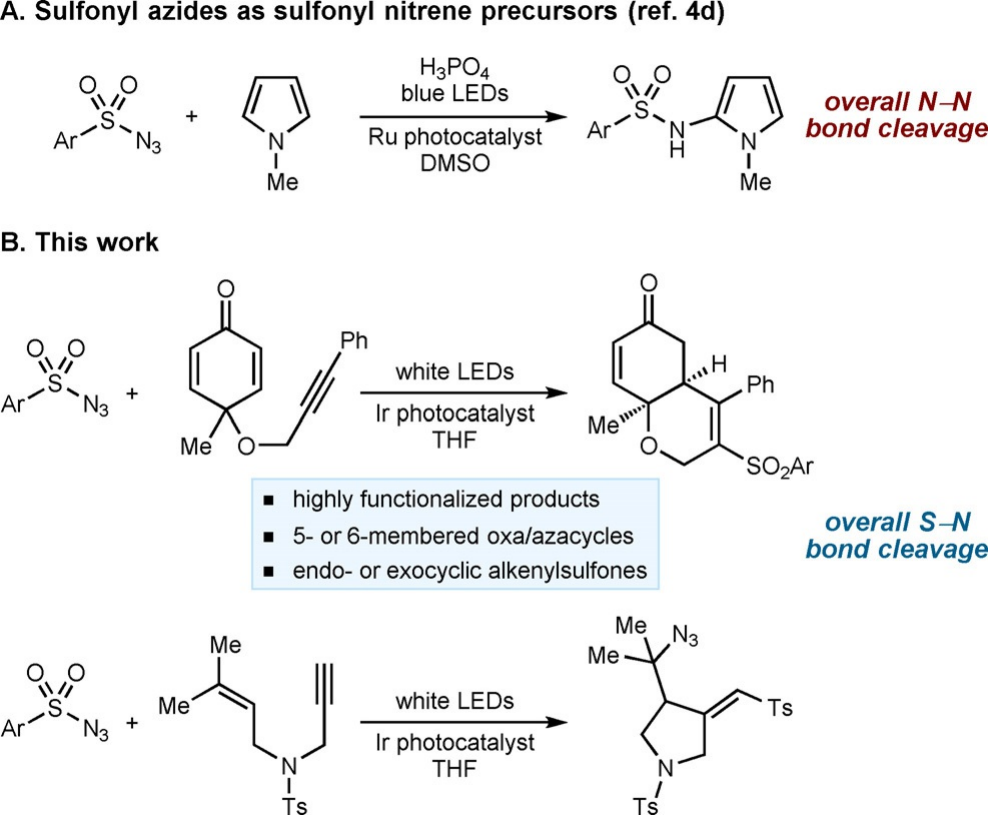
Sulfonyl azides in visible light photocatalysis.

However, sulfonyl azides have hardly been explored in visible light photocatalysis. To our knowledge, the only example reported used a sulfonyl azide as a precursor to a sulfonyl nitrene, which, in the presence of acid, reacted with *N*‐methylpyrrole in a C−H amidation (Scheme 1A).4d Given the versatility of sulfonyl azides,^1^, ^2^ their application in other classes of photocatalytic reactions could lead to valuable new synthetic opportunities. Herein, we describe radical cyclizations of enynes which use sulfonyl azides, visible light, and a photoactive iridium complex to give several classes of highly functionalized oxacycles and azacycles (Scheme 1B). In contrast to the aforementioned example,4d the overall net outcome is not cleavage of a nitrogen–nitrogen bond of the sulfonyl azide, but cleavage of the sulfur–nitrogen bond to give a sulfonyl radical, which is incorporated into the products. Depending upon the enyne, the products can also contain the azide group, a useful handle for further derivatizations.^1^, ^2^

## Results and Discussion

### Sulfonylative cyclizations

Prior reports of photocatalytic reactions of organic azides invoke the formation of reactive intermediates such as nitrogen‐centered radicals,4a,4i,4j azide radicals,4e,4g,4h and nitrenes.4b–4d,4f We therefore hoped that cyclohexa‐2,5‐dienone‐tethered alkyne **1 a**, which contains several unsaturated functional groups, would react productively with *para*‐toluenesulfonyl azide (**2 a**) under photocatalytic conditions (Table 1). Surprisingly, irradiation of a mixture of **1 a** and **2 a** (1.2 equiv) with white LEDs in the presence of 1.0 mol % of [Ir(dtbbpy)(ppy)_2_]PF_6_ (**Ir 1**) at room temperature (22 °C) gave essentially no reaction (<5 % conversion) in toluene, EtOAc, dioxane, MeCN, DCE, DMF, MeOH, or Et_2_O. In contrast, the reaction in THF for 36 h did result in consumption of **1 a** to give 6,6‐bicycle **3 a** in 85 % yield (entry 1).^5^ Unexpectedly, however, and despite the existing precedent,^4^ there was no incorporation of nitrogen into **3 a**. Instead, **3 a** results from addition of a sulfonyl radical^6^ to the alkyne of **1 a**, followed by 6‐*exo*‐*trig* cyclization. Products **4** and **5**, which would be derived from the addition of sulfonamidyl or azide radicals, respectively, were not observed. To our knowledge, the use of sulfonyl azides as sulfonylating agents without simultaneous incorporation of nitrogen is extremely rare.^7^ Raising the temperature to 32 °C increased the yield of **3 a** to 90 % (entry 2). Addition of TsOH⋅H_2_O (0.1 equiv) decreased the reaction time from 36 to 24 h and **3 a** was isolated in 91 % yield (entry 3). Other photocatalysts [Ir(bpy)(ppy)_2_]PF_6_ (**Ir 2**) and [Ir(dtbbpy){dF(CF_3_)ppy}_2_]PF_6_ (**Ir 3**) were also tested, and although these gave good yields of **3 a**, the reaction times were longer (entries 4 and 5). No conversion was observed in the absence of the iridium complex or in the dark, indicating that both the photocatalyst and light are essential.

**Table 1:** Evaluation of conditions for sulfonylative cyclization.^[a]^

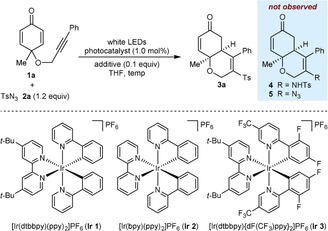					
Entry	Catalyst	*T* [°C]	Additive	*t* [h]	Yield [%]^[b]^
1	**Ir 1**	22	–	36	85
2	**Ir 1**	32	–	36	90
3	**Ir 1**	32	TsOH⋅H_2_O	24	91
4	**Ir 2**	32	TsOH⋅H_2_O	72	92
5	**Ir 3**	32	TsOH⋅H_2_O	32	87

[a] Reactions were conducted with 0.10 mmol of **1 a** in THF (2.5 mL) under a nitrogen atmosphere. [b] Yield of isolated product.

Table 2 presents the reactions of various sulfonyl azides and cyclohexa‐2,5‐dienone‐tethered alkynes **1**, which gave products **3 a**–**3 l** in 55–91 % yield.^5^ Regarding the alkyne substituent R^3^, the process is compatible with phenyl groups (**3 a** and **3 g**–**3 l**) and aryl groups containing alkyl or halide substituents (**3 d** and **3 f**). 3‐Pyridyl or 2‐thienyl groups on the alkyne are also well‐tolerated (**3 b** and **3 e**). A substrate containing a methyl‐substituted alkyne underwent successful sulfonylative cyclization but the product **3 c** was isolated together with an isomer resulting from initial addition of the sulfonyl radical to the methyl‐substituted alkyne carbon, as a 5:1 mixture. Changing the substituent at the quaternary center of the substrates from methyl (**3 a**–**3 c** and **3 g**–**3 l**) to ethyl (**3 d** and **3 e**) or phenyl (**3 f**) is possible, and various other sulfonyl azides are compatible (**3 g**–**3 l**). Finally, by using terminal alkyne **1 g**, the 6,5‐bicycle **6** was formed in 90 % yield.^5^

**Table 2:** Scope of sulfonylative cyclizations.^[a]^

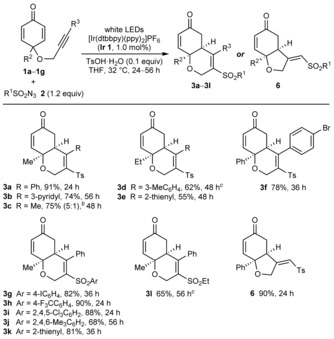

[a] Reactions were conducted with 0.20 mmol of **1** in THF (2.5 mL) under a nitrogen atmosphere. Yields are of isolated products. [b] Product **3 c** was isolated together with a 6,5‐bicyclic isomer resulting from initial addition of the sulfonyl radical to the methyl‐substituted alkyne carbon, in a 5 : 1 ratio (see the Supporting Information). [c] Using 3.0 equivalents of the sulfonyl azide.

### Azidosulfonylative cyclizations

Although the sulfonylative cyclizations shown in Table 2 represent a new mode of reactivity of sulfonyl azides in the presence of visible light and a photoactive complex, we were interested in whether the same reaction system could insert nitrogen functionality into the products. Pleasingly, by replacing the electron‐deficient alkene in the enyne with a more electron‐rich alkene, the reaction pathway is switched over to azidosulfonylative cyclization (Table 3).^8^ For example, irradiation of 1,6‐enyne **7 a** and *para*‐toluenesulfonyl azide (**2 a**, 1.5 equiv) with white LEDs in THF at 32 °C, in the presence of 1.0 mol % of **Ir 1** gave, after 36 h, azidosulfonylation product **8 a** in 45 % NMR yield along with the non‐azidated product **9 a** in 12 % NMR yield (entry 1).^5^ Increasing the quantity of **2 a** to 2.0 equivalents gave a slightly higher yield of **8 a** (entry 2). As with the sulfonylative cyclizations (Table 1), **Ir 2** and **Ir 3** were inferior to **Ir 1** (Table 3, entries 3 and 4). However, with **Ir 1**, increasing the quantity of **2 a** further to 3.0 equivalents led to a notable increase in conversion and a faster reaction, and **8 a** was isolated in 65 % yield after 18 h with none of **9 a** detected (entry 6).

**Table 3:** Evaluation of conditions for azidosulfonylative cyclization.^[a]^

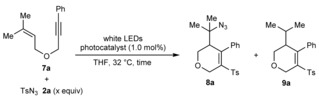						
Entry	Catalyst	**2 a** [equiv]	*t* [h]	Conv [%]^[b]^	Yield **8 a** [%]^[b]^	Yield **9 a** [%]^[b]^
1	**Ir 1**	1.5	36	66	45	12
2	**Ir 1**	2.0	36	87	48	8
3	**Ir 2**	2.0	36	28	15	0
4	**Ir 3**	2.0	36	44	20	0
5	**Ir 1**	3.0	18	>95	70 (65)^[c]^	<5

[a] Reactions were conducted with 0.40 mmol of **1 a** in THF (2.0 mL) under a nitrogen atmosphere. [b] Determined by ^1^H NMR analysis with 1,3,5‐trimethoxybenzene as an internal standard. [c] Yield of isolated product.

With effective conditions available, the scope of this process was explored (Table 4).^5^ Sulfonyl azides containing various aryl or alkyl substituents reacted successfully with **7 a** to give dihydropyrans **8 a**–**8 f** (entries 1–6). Variation of the aryl substituent of the alkyne to 4‐chlorophenyl, 3‐methoxyphenyl, or 2‐thienyl groups was tolerated (entries 7–9), as was cyclization onto a cyclohexylidene group (entries 10 and 12). By using substrates containing terminal alkynes, tetrahydrofurans **10 a**–**10 c** containing exocyclic alkenylsulfones were produced in 66–88 % yield (entries 11–13). Replacement of the ether tether with a sulfonamide led to various azacycles **8 k**, **10 d**, and **10 e** (entries 14–16).

**Table 4:** Scope of azidosulfonylative cyclizations.^[a]^

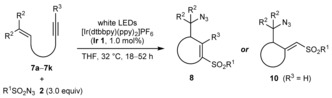					
Entry	Substrate	Product	R	*t* [h]	Yield [%]^[b]^
1	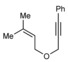	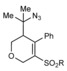	**8 a** 4‐MeC_6_H_4_	18	65
2			**8 b** 4‐IC_6_H_4_	36	83
3			**8 c** 4‐F_3_CC_6_H_4_	36	89
4			**8 d** 2‐naphthyl	36	73
5			**8 e** 2,4,5‐Cl_3_C_6_H_2_	36	91
6			**8 f** Et	52	80
7	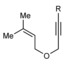	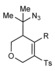	**8 g** 4‐ClC_6_H_4_	48	46
8			**8 h** 3‐MeOC_6_H_4_	15	75
9			**8 i** 2‐thienyl	52	40
10	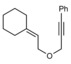	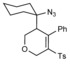	**8 j**	36	61
11	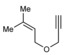	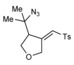	**10 a**	24	76
12	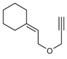	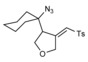	**10 b**	24	88
13	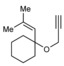	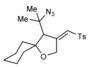	**10 c**	36	66
14	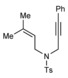	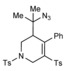	**8 k**	36	45
15	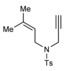	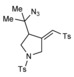	**10 d**	24	93
16	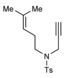	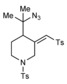	**10 e**	36	54

[a] Reactions were conducted with 0.40 mmol of **7** in THF (2.0 mL) under a nitrogen atmosphere. [b] Yield of isolated product.

Conducting the reactions on a larger scale at higher concentrations allowed the catalyst loading to be reduced to 0.5 mol% and importantly, the quantity of the sulfonyl azide to be lowered to 1.5 equivalents. For example, cyclization of **7 g** on a 2.0 mmol scale at 0.4 m concentration gave **8 k** in 46 % yield [Eq. (1)], while cyclization of **7 e** on a 3.0 mmol scale at 0.6 m concentration gave **10 b** in 74 % yield [Eq. (2)]. A small quantity of diene **11** was also isolated from the latter reaction.


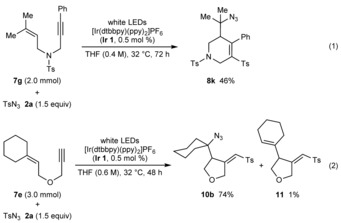


### Initial mechanistic considerations

Given that the only reported example of a visible light photocatalytic reaction of a sulfonyl azide proceeds through a sulfonyl nitrene (Scheme 1A),4d the generation of sulfonyl radicals in the reactions described herein was intriguing from a mechanistic standpoint. The observation that THF is a uniquely effective solvent suggests the reaction medium plays a key role in radical initiation. The reactions shown in Table 2 result from overall addition of a sulfonyl group and a hydrogen atom to the substrate. We therefore assumed that, in addition to its suspected role in radical initiation, the effectiveness of THF in the sulfonylative cyclizations arises from its ability to act as a hydrogen atom donor.^9^

To shed light on this latter issue, **1 a** was reacted with *para*‐toluenesulfonyl azide (**2 a**) in [D_8_]‐THF with **Ir 2** as the photocatalyst [Eq. (3)]. With the standard quantity of **2 a** (1.2 equiv), this reaction was much slower than the corresponding reaction using non‐deuterated THF (Table 1, entry 4). However, increasing the quantity of **2 a** to 10.0 equivalents and raising the temperature to 50 °C gave, after 96 h, a 45 % yield of a mixture of isotopologues **3 a**, [D]‐**3 a**, and [D_2_]‐**3 a**, which contain different numbers of deuterium atoms at the methylene carbon adjacent to the carbonyl group.^10^ The major component was the monodeuterated compound [D]‐**3 a** (likely a mixture of diastereomers), while the non‐deuterated compound **3 a** was a minor component. Mass spectrometry suggested a trace (ca. <5 %) of the di‐deuterated compound [D_2_]‐**3 a** was present. This result is consistent with the final product‐forming step being hydrogen/deuterium abstraction from THF, which may be rate‐limiting. The presence of all three isotopologues may be explained by reversible, acid‐catalyzed hydrogen–deuterium exchange through enol intermediates.


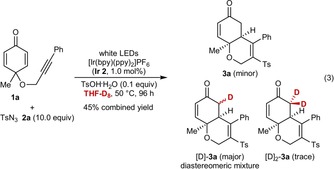


### Proposed radical chain mechanisms

**Scheme 2:**
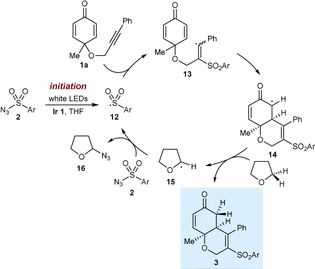
Proposed mechanism for sulfonylative cyclization.

We consider it likely that the sulfonylative cyclizations operate through radical chain mechanisms (Scheme 2).^11^ First, irradiation of the sulfonyl azide **2 a** in the presence of the iridium complex and THF produces the sulfonyl radical **12**. Possible pathways for this initiation are discussed below. Addition of **12** to the alkyne of the substrate **1 a** gives an alkenyl radical **13**, which cyclizes onto one of the alkenes to give a new radical **14**. It is well‐known that electrophilic enolate radicals such as **14** do not react with sulfonyl azides to give azidation products.2d However, a hydrogen abstraction from THF, as suggested by the results of Equation (3), would give product **3 a** along with the nucleophilic tetrahydrofuran‐2‐yl radical **15**.^9^ In a chain propagation step, **15** could react with the sulfonyl azide to give azide **16** and regenerate the sulfonyl radical **12**. The beneficial effect of TsOH⋅H_2_O is not currently known.

**Scheme 3:**
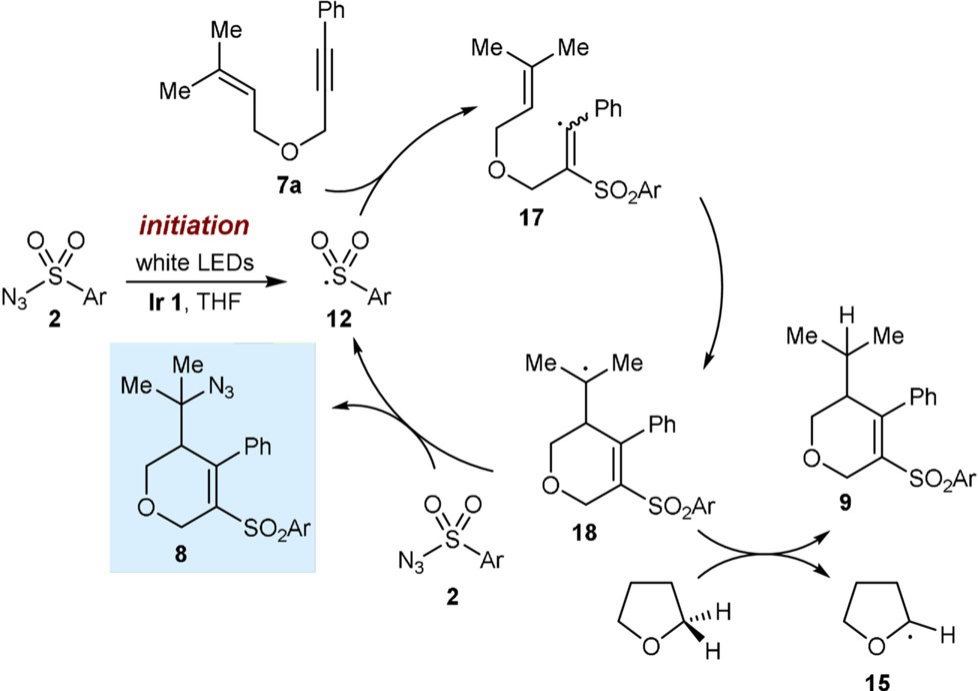
Proposed mechanism for azidosulfonylative cyclization.

We believe the azidosulfonylative cyclizations also proceed through a radical chain cycle (Scheme 3).^8^,^11^ After radical initiation, the sulfonyl radical **12** adds to the alkyne of **7 a** to give alkenyl radical **17**, which undergoes 6‐*exo*‐*trig* cyclization onto the alkene to give tertiary radical **18**. Azidation of **18** with the sulfonyl azide **2** in a chain propagation step gives the product **8** and regenerates the sulfonyl radical **12**.2d The formation of the non‐azidated byproduct **9** (Table 3) can be explained by radical **18** undergoing competitive hydrogen atom abstraction with the solvent THF.

### The role of THF in radical initiation

Although both the sulfonylative and azidosulfonylative cyclizations are readily explained by radical chain mechanisms (Schemes 2 and 3), the question remains of how the combination of visible light, photoactive iridium complex, THF, and the sulfonyl azide leads to the generation of sulfonyl radicals.

In principle, single‐electron‐transfer from the photoexcited iridium complex to the electrophilic sulfonyl azide, followed by fragmentation of the resulting radical anion would give an azide anion and the requisite sulfonyl radical **12**. Single‐electron‐transfer to organic azides has been postulated in photocatalytic reactions.4a,4i However, the reduction potential *E*_1/2_^red^ of *para*‐toluenesulfonyl azide (**2 a**) was measured by cyclic voltammetry to be −1.22 V versus SCE in MeCN,^12^ and it would appear that the photoexcited states of the iridium complexes **Ir 1**–**3** are insufficiently reducing to promote this electron transfer efficiently (**Ir 1**, E*^III/IV^=−0.96 V vs. SCE;3e **Ir 2**, E*^III/IV^=−0.85 V vs. SCE,^13^ and **Ir 3**, E*^III/IV^=−0.89 V vs. SCE3e). The superiority of THF over other solvents is also not readily explained by an electron transfer mechanism.

**Scheme 4:**
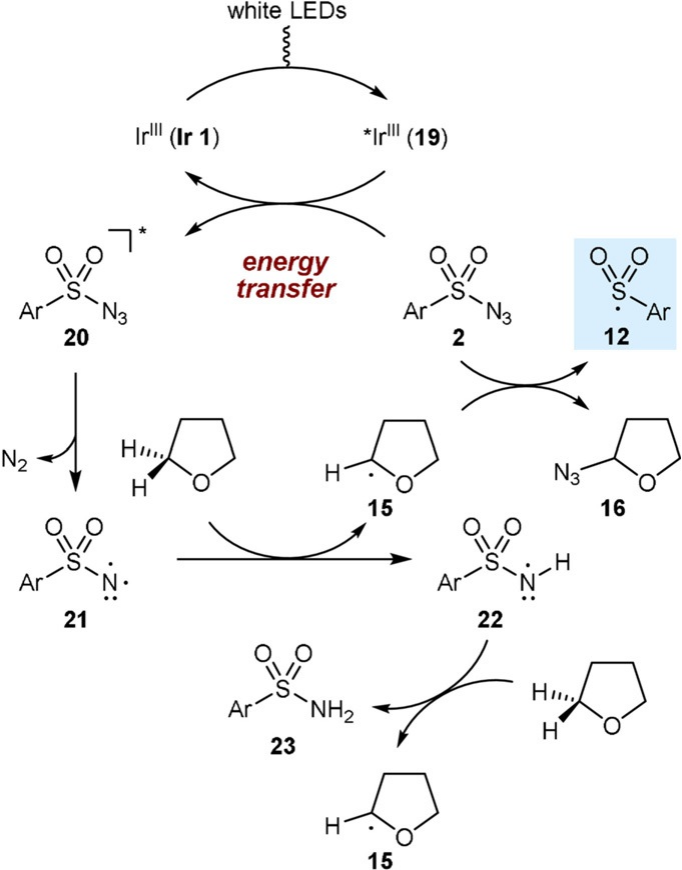
Radical initiation by triplet sensitization.

A second mechanism that we consider more likely begins with irradiation of **Ir 1** (depicted as Ir^III^) to give the photoexcited ^*^Ir^III^ species **19** (Scheme 4). Triplet sensitization of the sulfonyl azide by an energy transfer mechanism gives **20**, which then loses dinitrogen to give a triplet nitrene **21**. This pathway is consistent with the only reported example of a visible light photocatalytic reaction of a sulfonyl azide (Scheme 1A), which also proceeds through a sulfonyl nitrene.4d The formation of a sulfonyl nitrene from UV irradiation of a sulfonyl azide with benzophenone as a triplet sensitizer is also known.^14^ Furthermore, other electron‐deficient azides such as acyl azides and azidoformates are known to produce nitrenes by triplet sensitization with photoactive metal complexes.4b–4d,4f The triplet nitrene **21** could then abstract a hydrogen atom from THF to give tetrahydrofuran‐2‐yl radical **15** and sulfonamidyl radical **22**. In relevant precedent, it is known that triplet sulfonyl nitrenes can abstract a hydrogen atom from the methine carbon of *i‐*PrOH.^14^ Azidation of **15** with the sulfonyl azide would then provide the sulfonyl radical **12** to enter the chain mechanisms shown in Schemes 2 and 3. The sulfonamidyl radical **22** could then undergo a second hydrogen abstraction with THF to give *para*‐toluenesulfonamide (**23**). It should be noted that we did observe the formation of small quantities of **23** in all of the reactions reported in Tables 2 and 4, which lends some support for the participation of triplet nitrene intermediates.

Furthermore, reaction of 1,6‐enyne **7 a** with **2 a** in DCE rather than THF gave aziridine **24** in 42 % yield [Eq. (4)]. Evidently, in the absence of THF, the putative triplet nitrene **21** reacts with the alkene of **7 a** to give **24**, presumably by a stepwise radical addition and ring closure as described by Yoon and co‐workers.4f 
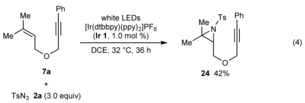


### Implications for other reactions

As discussed above, our collective results point to the formation of tetrahydrofuran‐2‐yl radical **15** from the reaction of THF with a triplet sulfonyl nitrene **21** derived from a sulfonyl azide **2** (Scheme 4). Although this process leads to the generation of sulfonyl radicals by subsequent reaction of **15** with the sulfonyl azide **2**, we questioned whether **15** could be formed by the reaction of THF with triplet nitrenes derived from azides that are unreactive toward **15**. If so, it might be possible to utilize **15** in a carbon–carbon bond‐forming reaction.

In the event, irradiation of phenyl acrylate (**25**) in THF in the presence of **Ir 1** (1.0 mol %) and benzyl azidoformate (**26**, 0.2 equiv) gave addition product **27** in an unoptimized 42 % yield [Eq. (5)]. The reaction of phenyl vinyl ketone (**28**) gave similar results, producing **29** in 34 % yield [Eq. (6)]. No reaction was observed when these reactions were repeated in the absence of the azide or the photocatalyst under otherwise identical conditions.


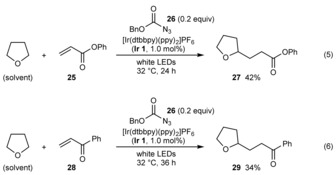


## Conclusions

We have described sulfonylative and azidosulfonylative cyclizations of enynes that give several classes of highly functionalized heterocycles. These reactions operate through radical chain mechanisms, with the combination of sulfonyl azide, THF, visible light, and a photoactive iridium complex serving as a “smart initiation”11a system for the generation of sulfonyl radicals. Radical initiation begins with the photosensitized formation of a triplet nitrene from the sulfonyl azide, followed by hydrogen atom transfer from THF to the nitrene to give a tetrahydrofuran‐2‐yl radical, which then reacts with the sulfonyl azide to produce the sulfonyl radical. By using an azidoformate instead of the sulfonyl azide, the tetrahydrofuran‐2‐yl radical can be intercepted by electron‐deficient alkenes. This work further demonstrates that spin‐selective formation of triplet nitrenes from organic azides using visible light photocatalysis can serve as a powerful platform for new reaction development.^15^

## Conflict of interest

The authors declare no conflict of interest.

## Supporting information

As a service to our authors and readers, this journal provides supporting information supplied by the authors. Such materials are peer reviewed and may be re‐organized for online delivery, but are not copy‐edited or typeset. Technical support issues arising from supporting information (other than missing files) should be addressed to the authors.

Supplementary
