# Supplementary material for: Sulfonylative and Azidosulfonylative Cyclizations by Visible‐Light‐Photosensitization of Sulfonyl Azides in THF
Source: Chemistry. 2017 Nov 16;23(69):17598–604. doi: 10.1002/chem.201704380 (PMC5765429; doi:10.1002/chem.201704380)
Supplement: Supplementary file 1 — Supplementary [file CHEM-23-17598-s001.pdf]

CHEMISTRY
A European Journal

Supporting Information

Sulfonylative and Azidosulfonylative Cyclizations by Visible-Light-Photosensitization of Sulfonyl Azides in THF

Shaoqun Zhu,[a, b] Atchutarao Pathigoolla,[a, b] Grace Lowe,[a, b] Darren A. Walsh,[a, b] Mick Cooper,[b] William Lewis,[b] and Hon Wai Lam*[a, b]

chem_201704380_sm_miscellaneous_information.pdf

Supporting Information

Contents **Page**

Contents	Page
General Information	2
Light Source for Photochemical Experiments	3
Preparation of Sulfonyl Azides	3
Preparation of Enynes	4
Sulfonylative Cyclizations of Enynes	11
Azidosulfonylative Cyclizations of Enynes	20
Sulfonylative Cyclization in THF-D_8	31
Aziridination of 1,6-Enyne **7a**	35
Addition of Tetrahydrofuran-2-yl Radical to Electron-Deficient Alkenes	36
Dihydroxylation of **3c**	37
Cyclic Voltammetry of TsN_3	39
NMR Spectra	41
References	88

General Information

THF, toluene, and diethyl ether used in reactions were dried and purified by passage through activated alumina columns using a solvent purification system. "Petrol" refers to petroleum ether boiling in the range 40–60°C. All commercially available dry solvents were purchased: 2-Me-THF, 1,4-dioxane, MeCN, from Acros Organics and DMF, DMSO from Sigma-Aldrich. Thin layer chromatography (TLC) was performed on Merck DF-Alufoilien 60F254 0.2 mm precoated plates. Product spots were visualized by UV light at 254 nm. Flash column chromatography was carried out using silica gel (Fisher Scientific 60Å particle size 35-70 micron). Melting points were recorded on a Gallenkamp melting point apparatus and are uncorrected. Infra-red spectra were recorded on a Shimadzu IRAffinity-1 instrument on the neat compound using the attenuated total reflectance sampling technique. For ^{1}H NMR spectra, chemical shifts (δ) are quoted in parts per million (ppm) downfield of tetramethylsilane, using residual protonated solvent as internal standard ($CDCl_3$ at 7.26 ppm and DMSO-d_6 at 2.54 ppm). Abbreviations used in the description of resonances are: s (singlet), d (doublet), t (triplet), q, (quartet), quin (quintet), app (apparent), br (broad). Coupling constants (*J*) are quoted to the nearest 0.1 Hz. For proton-decoupled ^{13}C NMR spectra, chemical shifts (δ) are quoted in parts per million (ppm) downfield of tetramethylsilane, using deuterated solvent as internal standard ($CDCl_3$ at 77.0 ppm and DMSO-d_6 at 40.45 ppm). High resolution mass spectra were recorded using electrospray ionization (ESI) or electron impact ionization (EI) techniques. [Ir(bpy)(ppy)$_2$]PF_6 was prepared according to a previously reported procedure.[1] [Ir(dtbbpy)(ppy)$_2$]PF_6 and [Ir(dtbbpy){dF(CF_3)ppy}$_2$]PF_6 were purchased from Sigma-Aldrich.

Light Source for Photochemical Experiments

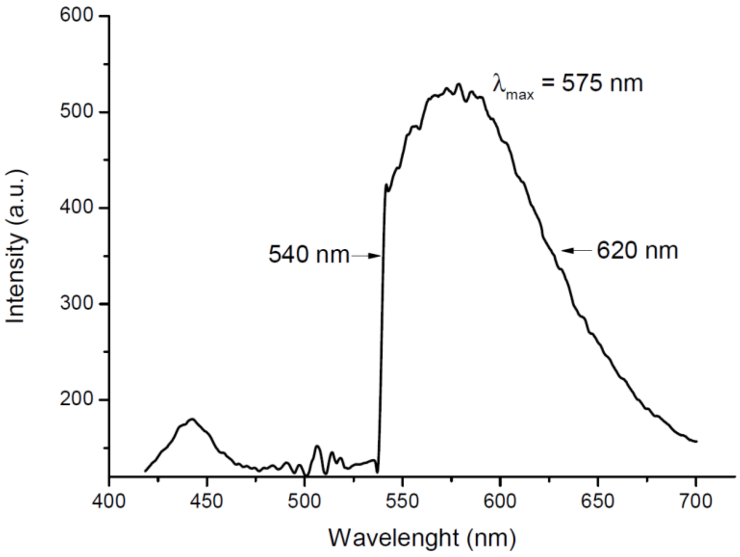

The emission spectrum of the light source was recorded using a spectroscopy system equipped with a spectrograph (Shamrock 303i, Andor technologies, UK) and a thermoelectrically cooled Raman CCD camera (iDus401, Andor technologies, UK):

The light source used for photochemical experiments was a DK-00280 desk lamp (Lamp Rating 220–240 V~50 Hz, LED Rating 3 V 0.5 W x 12), purchased from Argos: http://www.argos.co.uk/static/Product/partNumber/2266433.htm

Preparation of Sulfonyl Azides[2a]

$$RSO_2Cl + NaN_3 \xrightarrow[0\ °C\ to\ RT]{acetone/H_2O} RSO_2N_3$$

A solution of sodium azide (**caution, highly toxic and possible explosion hazard!**, 780 mg, 12.0 mmol) in H_2O (20 mL) was slowly added to a solution of the sulfonyl chloride (10 mmol) in acetone (25 mL) which was cooled in an ice bath, and the mixture was stirred for 2 h while being allowed to warm to room temperature. The reaction was concentrated under reduced pressure to remove the acetone and extracted with Et_2O (2 × 40 mL). The combined organic layers were dried ($NaSO_4$) and concentrated under reduced pressure (temperature below 25 °C) and purified by column chromatography (EtOAc/petrol) to give the sulfonyl azides. All spectroscopic data were in accordance with those reported in the literature.[2 b–g]

Preparation of Enynes

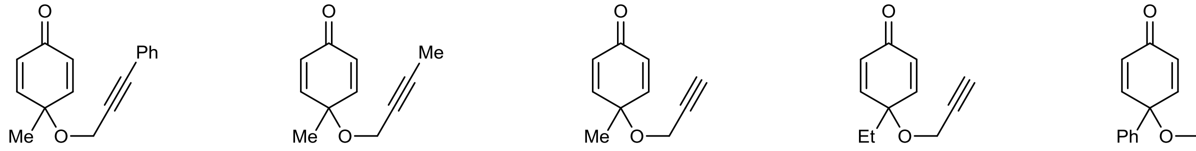

1a **1c** **S1** **S2** **1g**

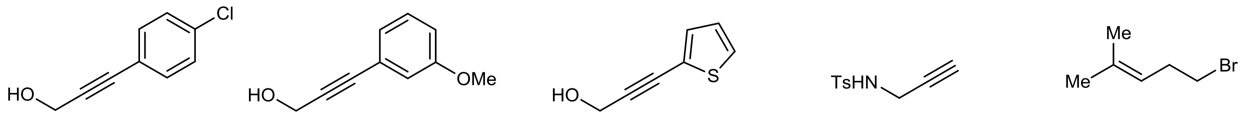

S3 **S4** **S5** **S6** **S7**

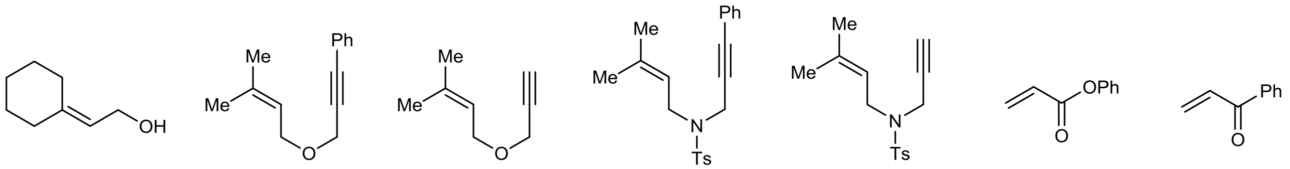

S8 **7a** **7f** **7i** **7j** **25** **28**

Compounds **1a**,[3] **1c**,[4] **S1**,[5] **S2**,[6] **1g**,[7] **S3**,[8] **S4**,[9] **S5**,[8] **S6**,[10] **S7**,[11] **S8**,[12] **7a**,[13] **7f**,[14] **7i**,[10] **7j**,[10] **25**[15] and **28**[15] were prepared according to previously reported procedures. Citations to references containing characterization data for these compounds are as follows: **S3**–**S5**,[16] **S6**,[17] **S7**,[18] **S8**,[19] **7a**,[13] **7f**,[14] **7i**,[13] **7j**,[20] **25**,[15] and **28**.[15]

General Procedure A

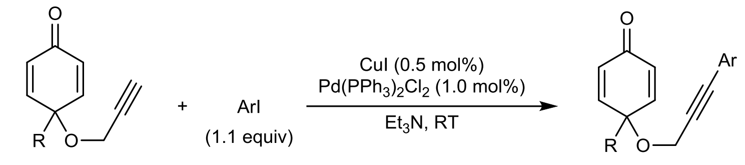

S1, **S2**, or **1g** **1a**, **1b**, **1d–1f**

A solution of terminal alkyne **S1**, **S2**, or **1g** (2.00 mmol) and the appropriate aryl iodide (2.20 mmol) in Et_3N (12 mL) was added to a flask containing CuI (2.0 mg, 0.01 mmol) and $Pd(PPh_3)_2Cl_2$ (15.0 mg, 0.02 mmol) under N_2, and the mixture was stirred at room temperature for the indicated time. The reaction was diluted with Et_2O (50 mL) washed with 10% aqueous HCl solution (20 mL), H_2O (20 mL), and brine (20 mL), dried ($NaSO_4$), filtered, and concentrated *in vacuo*. Purification of the residue by column chromatography (10% EtOAc/petrol) gave the alkynyl cyclohexadienone.

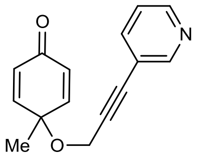

4-Methyl-4-[(3-(pyridin-3-yl)prop-2-yn-1-yl)oxy]cyclohexa-2,5-dienone (1b). The title compound was prepared according to General Procedure A from terminal alkyne **S1** (324 mg, 2.00 mmol) and 3-iodopyridine (451 mg, 2.20 mmol) for a reaction time

of 18 h and purified by column chromatography (33% EtOAc/petrol) to give a colorless amorphous powder (325 mg, 68%). R_f = 0.42 (50% EtOAc/petrol); IR 2980, 2132 (C≡C), 1663 (C=O), 1630, 1476, 1445, 1407, 1380, 1189, 1079, 1035, 859, 803, 702 cm^{-1}; ^{1}H NMR (400 MHz, $CDCl_3$) δ 8.67 (1H, dd, *J* = 2.0, 0.7 Hz, Ar**H**), 8.55 (1H, dd, *J* = 4.9, 1.7 Hz, Ar**H**), 7.71 (1H, dt, *J* = 7.9, 1.9 Hz, Ar**H**), 7.26 (1H, ddd, *J* = 7.9, 4.9, 0.9 Hz, Ar**H**), 6.94–6.85 (2H, m, 2 x C**H**=CHC=O), 6.40–6.32 (2H, m, 2 x C**H**C=O), 4.25 (2H, d, *J* = 4.1 Hz, OC**H**$_2$), 1.53 (3H, s, C**H**$_3$); ^{13}C NMR (101 MHz, $CDCl_3$) δ 184.9 (C), 152.3 (CH), 150.6 (2 x CH), 149.0 (CH), 138.6 (CH), 130.6 (2 x CH), 123.0 (CH), 119.5 (C), 89.1 (C), 83.4 (C), 73.3 (C), 54.3 (CH_2), 26.3 (CH_3); HRMS (ESI) Exact mass calculated for $[C_{15}H_{13}NO_2Na]^+$ $[M+Na]^+$: 262.0838, found: 262.0841.

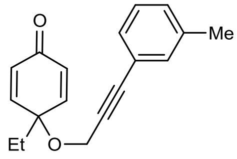

4-Ethyl-4-{[3-(*m*-tolyl)prop-2-yn-1-yl]oxy}cyclohexa-2,5-dien-1-one (1d). The title compound was prepared according to General Procedure A from terminal alkyne **S2** (352 mg, 2.00 mmol) and 1-iodo-3-methylbenzene (283 µL, 2.20 mmol) for a reaction time of 18 h and purified by column chromatography (15% EtOAc/petrol) to give a colorless amorphous powder (511 mg, 96%). R_f = 0.42 (11% EtOAc/petrol); IR 2870, 2239 (C≡C), 1663 (C=O), 1624, 1482, 1370, 1270, 1195, 1057, 876, 787, 690 cm^{-1}; ^{1}H NMR (400 MHz, $CDCl_3$) δ 7.25–7.18 (3H, m, Ar**H**), 7.16–7.13 (1H, m, Ar**H**), 6.87–6.82 (2H, m, 2 x C**H**=CHC=O), 6.43–6.39 (2H, m, 2 x C**H**C=O), 4.26 (2H, s, OC**H**$_2$), 2.33 (3H, s, ArC**H**$_3$), 1.87 (2H, q, *J* = 7.6 Hz, C**H**$_2$CH$_3$), 0.87 (3H, t, *J* = 7.6 Hz, CH$_2$C**H**$_3$); ^{13}C NMR (101 MHz, $CDCl_3$) δ 185.5 (C), 150.2 (2 x CH), 138.0 (C), 132.3 (CH), 131.6 (2 x CH), 129.5 (CH), 128.8 (CH), 128.2 (CH), 122.2 (C), 86.9 (C), 85.6 (C), 77.2 (C), 54.5 (CH_2), 32.3 (CH_2), 21.2 (CH_3), 7.9 (CH_3); HRMS (ESI) Exact mass calculated for $[C_{18}H_{18}O_2Na]^+$ $[M+Na]^+$: 289.1199, found: 289.1210.

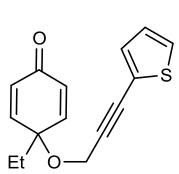

4-Ethyl-4-{[3-(thiophen-2-yl)prop-2-yn-1-yl]oxy}cyclohexa-2,5-dien-1-one (1e). The title compound was prepared according to General Procedure A from terminal alkyne **S2** (352 mg, 2.00 mmol) and 2-iodothiophene (243 *µ*L, 2.20 mmol) for a reaction time of 24 h and purified by column chromatography (10% EtOAc/petrol) to give a colorless amorphous powder (362 mg, 70%). R_f = 0.38 (17% EtOAc/petrol); IR 2965, 2934, 2230, 2194 (C≡C), 1663 (C=O), 1630, 1377, 1038, 1008, 856, 700 cm^{-1}; ^{1}H NMR (400 MHz, $CDCl_3$) δ 7.27 (1H, dd, *J* = 5.1, 1.2 Hz, Ar**H**), 7.20 (1H, dd, *J* = 3.6, 1.2 Hz, Ar**H**), 6.97 (1H, dd, *J* = 5.2, 3.6 Hz, Ar**H**), 6.85–6.81 (2H, m, 2 x C**H**=CHC=O), 6.43–6.39 (2H, m, 2 x C**H**C=O), 4.27 (2H, s, OC**H**$_2$), 1.86 (2H, q, *J* = 7.6 Hz, C**H**$_2$CH$_3$), 0.86 (3H, t, *J* = 7.6 Hz, CH$_2$C**H**$_3$); ^{13}C NMR (101 MHz, $CDCl_3$) δ 185.4 (C), 150.1 (2 x CH), 132.5 (CH), 131.7 (2 x CH), 127.6 (CH), 126.9 (CH), 122.3 (C), 90.0 (C), 80.2

(C), 77.2 (C), 54.5 (CH_2), 32.3 (CH_2), 7.8 (CH_3); HRMS (ESI) Exact mass calculated for $[C_{15}H_{14}SO_2]^+$ $[M+H]^+$: 281.0607, found: 281.0618.

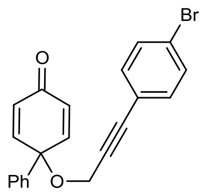

1-{[3-(4-Bromophenyl)prop-2-yn-1-yl]oxy}-[1,1'-biphenyl]-4(1*H*)-one (1f). The title compound was prepared according to General Procedure A from terminal alkyne **1g** (449 mg, 2.00 mmol) and 1-bromo-4-iodobenzene (622 mg, 2.20 mmol) for a reaction time of 20 h and purified by column chromatography (10% EtOAc/petrol) to give a colorless amorphous powder (485 mg, 64%). R_f = 0.45 (17% EtOAc/petrol); IR 2862, 2246 (C≡C), 1662 (C=O), 1625, 1483, 1371, 1057, 1013, 864, 753, 696 cm^{-1}; ^1H NMR (400 MHz, $CDCl_3$) δ 7.55–7.45 (4H, m, Ar**H**), 7.43–7.29 (5H, m, Ar**H**), 6.99–6.91 (2H, m, 2 x C**H**=CHC=O), 6.49–6.41 (2H, m, 2 x C**H**C=O), 4.48 (2H, s, OC**H**$_2$); ^{13}C NMR (101 MHz, $CDCl_3$) δ 185.4 (C), 149.6 (2 x CH), 137.6 (C), 133.1 (2 x CH), 131.6 (2 x CH), 130.0 (2 x CH), 128.9 (2 x CH), 128.6 (CH), 125.8 (2 x CH), 123.0 (C), 121.4 (C), 87.0 (C), 85.9 (C), 77.2 (C), 54.2 (CH_2); HRMS (ESI) Exact mass calculated for $[C_{21}H_{15}BrO_2Na]^+$ $[M+Na]^+$: 401.0148, found: 401.0158.

1-Chloro-4-{3-[(3-methylbut-2-en-1-yl)oxy]prop-1-yn-1-yl}benzene (7b)

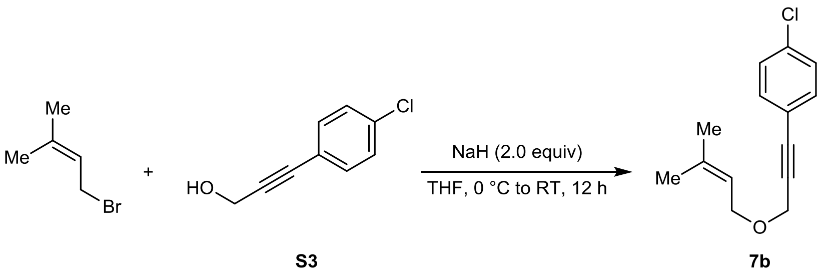

A solution of propargylic alcohol **S3**[8] (1.00 g, 6.00 mmol) in THF (5 mL) was added to a solution of NaH (480 mg, 60% in paraffin oil, 12.0 mmol) in THF (20 mL) at 0 °C. After 30 min, a solution of 1-bromo-3-methylbut-2-ene (1.0 mL, 8.66 mmol) in THF (5 mL) was added over 5 min by syringe. The reaction mixture was warmed to room temperature and stirred for 12 h. The reaction was quenched carefully with ice-cooled water and extracted with Et_2O (2 × 25 mL). The extracts were washed with H_2O (10 mL), brine (10 mL), dried ($NaSO_4$), filtered, and concentrated *in vacuo*. Purification of the residue by column chromatography (10% Et_2O/petrol) gave **7b** as a colorless oil (1.20 g, 85%). R_f = 0.40 (10% Et_2O/petrol); IR 2971, 2932, 2913, 2851, 1738, 1488, 1441, 1352, 1258, 1067, 1014, 826, 753 cm^{-1}; ^1H NMR (400 MHz, $CDCl_3$) δ 7.41–7.34 (2H, m, Ar**H**), 7.31–7.25 (2H, m, Ar**H**), 5.39 (1H, app tdq, J = 7.1, 2.8, 1.4 Hz, CH_2C**H**=C), 4.35 (2H, s, C**H**$_2$C≡C), 4.13 (2H, d, J = 7.1 Hz, C**H**$_2$CH=C), 1.78 (3H, d, J = 1.4 Hz, C**H**$_3$), 1.73 (3H, d, J = 1.3 Hz, C**H**$_3$); ^{13}C NMR (101 MHz, $CDCl_3$) δ 138.2 (C),

134.3 (C), 132.8 (2 x CH), 128.5 (2 x CH), 121.2 (C), 120.2 (CH), 86.5 (C), 84.7 (C), 66.0 (CH_2), 57.4 (CH_2), 25.7 (CH_3), 18.0 (CH_3); HRMS (ESI) Exact mass calculated for $[C_{14}H_{15}ClONa]^+$ $[M+Na]^+$: 257.0704, found: 257.0700.

1-Methoxy-3-{3-[(3-methylbut-2-en-1-yl)oxy]prop-1-yn-1-yl}benzene (7c)

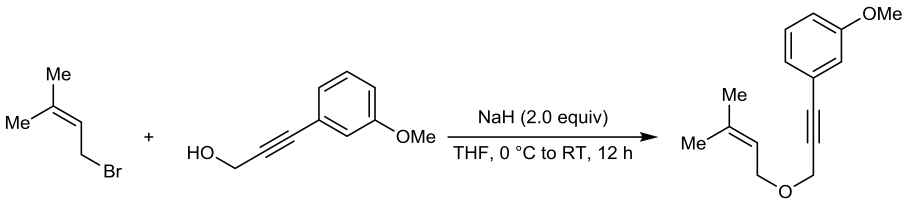

A solution of propargylic alcohol **S4**[9] (300 mg, 1.85 mmol) in THF (5 mL) was added to a solution of NaH (148 mg, 60% in paraffin oil, 3.70 mmol) in THF (20 mL) at 0 °C. After 30 min, a solution of 1-bromo-3-methylbut-2-ene (0.3 mL, 2.6 mmol) in THF (2 mL) was added over 5 min by syringe. The reaction mixture was warmed to room temperature and stirred for 12 h. The reaction was quenched carefully with ice-cooled water and extracted with Et_2O (2 × 25 mL). The extracts were washed with H_2O (10 mL), brine (10 mL), dried ($NaSO_4$), filtered, and concentrated *in vacuo*. Purification of the residue by column chromatography (10% Et_2O/petrol) gave **7c** as a colorless oil (340 mg, 80%). R_f = 0.45 (10% Et_2O/petrol); IR 2967, 2934, 2851, 2228 (C≡C), 1715, 1597, 1480, 1317, 1287, 1200, 1163, 1067, 1043, 779, 686 cm^{-1}; ^{1}H NMR (400 MHz, $CDCl_3$) δ 7.24–7.18 (1H, m, Ar**H**), 7.04 (1H, dt, *J* = 7.6, 1.2 Hz, Ar**H**), 6.98 (1H, dd, *J* = 2.7, 1.4 Hz, Ar**H**), 6.87 (1H, ddd, *J* = 8.4, 2.6, 1.0 Hz, Ar**H**), 5.38 (1H, app tdq, *J* = 7.8, 2.8, 1.4 Hz, CH_2C**H**=C), 4.35 (2H, s, C**H**$_2$C≡C), 4.12 (2H, d, *J* = 7.1 Hz, C**H**$_2$CH=C), 3.79 (3H, s, OC**H**$_3$), 1.77 (3H, d, *J* = 1.3 Hz, CC**H**$_3$), 1.73 (3H, d, *J* = 1.4 Hz, CC**H**$_3$); ^{13}C NMR (101 MHz, $CDCl_3$) δ 159.2 (C), 138.3 (C), 129.3 (CH), 124.2 (CH), 123.7 (C), 120.3 (CH), 116.6 (CH), 114.9 (CH), 85.9 (C), 85.3 (C), 66.0 (CH_2), 57.5 (CH_2), 55.2 (CH_3), 25.8 (CH_3), 18.0 (CH_3); HRMS (ESI) Exact mass calculated for $[C_{15}H_{18}O_2Na]^+$ $[M+Na]^+$: 253.1199, found: 253.1203.

2-{3-[(3-Methylbut-2-en-1-yl)oxy]prop-1-yn-1-yl}thiophene (7d)

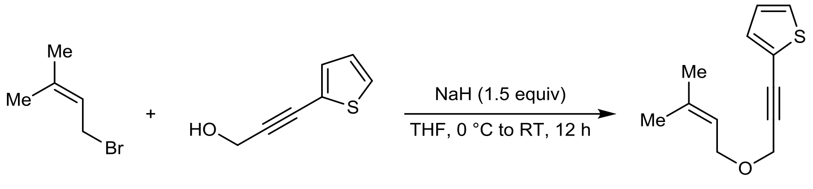

A solution of propargylic alcohol **S5**[8] (500 mg, 3.62 mmol) in THF (5 mL) was added to a solution of NaH (217 mg, 60% in paraffin oil, 5.43 mmol) in THF (20 mL) at 0 °C. After 30 min, a solution of 1-bromo-3-methylbut-2-ene (0.63 mL, 5.45 mmol) in THF (2 mL) was added over 5 min by syringe. The reaction mixture was warmed to room temperature and stirred for 12 h. The reaction was quenched carefully with ice-cooled water and extracted with Et_2O (2 × 25 mL). The extracts were washed with H_2O (10 mL), brine (10 mL), dried ($NaSO_4$), filtered, and concentrated *in vacuo*. Purification of the residue by column chromatography (10% Et_2O/petrol) gave **7d** as a colorless oil (595 mg, 80%). R_f = 0.55 (10% Et_2O/petrol); IR 3104, 2971, 2931, 2913, 2851, 2219 (C≡C), 1738, 1673, 1438, 1376, 1357, 1189, 1064, 930, 848, 699 cm^{-1}; ^{1}H NMR (400 MHz, $CDCl_3$) δ 7.25 (1H, dd, J = 5.2, 1.2 Hz, Ar**H**), 7.21 (1H, dd, J = 3.6, 1.2 Hz, Ar**H**), 6.96 (1H, dd, J = 5.2, 3.6 Hz, Ar**H**), 5.37 (1H, app tdq, J = 7.2, 2.9, 1.5 Hz, CH_2C**H**=C), 4.36 (2H, s, C**H_2**C≡C), 4.11 (2H, app dt, J = 7.2, 0.9 Hz, C**H_2**CH=C), 1.77 (3H, s, C**H_3**), 1.72 (3H, d, J = 1.3 Hz, C**H_3**); ^{13}C NMR (101 MHz, $CDCl_3$) δ 138.4 (C), 132.2 (CH), 127.2 (CH), 126.9 (CH), 122.7 (C), 120.2 (CH), 89.5 (C), 79.2 (C), 66.0 (CH_2), 57.6 (CH_2), 25.8 (CH_3), 18.1 (CH_3); HRMS (ESI) Exact mass calculated for $[C_{12}H_{14}SONa]^+$ $[M+Na]^+$: 229.0658, found: 229.0656.

[3-(2-Cyclohexylideneethoxy)prop-1-yn-1-yl]benzene (7e)

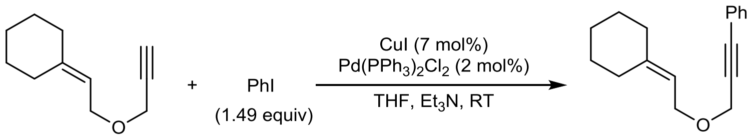

A solution of iodobenzene (0.66 mL, 5.92 mmol), CuI (53 mg, 0.28 mmol), $Pd(PPh_3)_2Cl_2$ (56.0 mg, 0.08 mmol), and Et_3N (1.1 mL, 7.89 mmol) in THF (5 mL) was stirred at room temperature for 30 min. A solution of alkyne **7g** (see next page, 650 mg, 3.96 mmol) in THF (5 mL) was then added and the mixture was stirred for 12h. The reaction was diluted with Et_2O (100 mL), washed with 10% aqueous HCl solution (50 mL), H_2O (20 mL), and brine (20 mL), dried ($NaSO_4$), filtered, and concentrated *in vacuo*. Purification of the residue by column chromatography (10% Et_2O /petrol) gave **7e** as a colorless oil (760 mg, 80%). R_f = 0.30 (10% Et_2O/petrol); IR 2926, 2852, 1738, 1667, 1489, 1442, 1351, 1338, 1069, 1027, 754, 690, 526 cm^{-1}; ^{1}H NMR (400 MHz, $CDCl_3$) δ 7.48–7.41 (2H, m, Ar**H**), 7.35–7.27

(3H, m, Ar**H**), 5.32 (1H, app tquin, *J* = 7.1, 1.2 Hz, CH_2C**H**=C), 4.36 (2H, s, C**H**$_2$C≡C), 4.15 (2H, d, *J* = 7.2 Hz, C**H**$_2$CH=C), 2.24 (2H, t, *J* = 5.4 Hz, C**H**$_2$C=CH), 2.14 (2H, d, *J* = 5.7 Hz, C**H**$_2$C=CH), 1.59-1.53 (6H, m, CH_2(C**H**$_2$)$_3$$CH_2$); ^{13}C NMR (101 MHz, $CDCl_3$) δ 146.2 (C), 131.7 (2 x CH), 128.3 (CH), 128.2 (2 x CH), 122.8 (C), 116.9 (CH), 85.9 (C), 85.5 (C), 65.1 (CH_2), 57.3 (CH_2), 37.1 (CH_2), 29.0 (CH_2), 28.4 (CH_2), 27.8 (CH_2), 26.7 (CH_2); HRMS (ESI) Exact mass calculated for $[C_{17}H_{20}ONa]^+$ $[M+Na]^+$: 263.1406, found: 263.1403.

[2-(Prop-2-yn-1-yloxy)ethylidene]cyclohexane (7g)

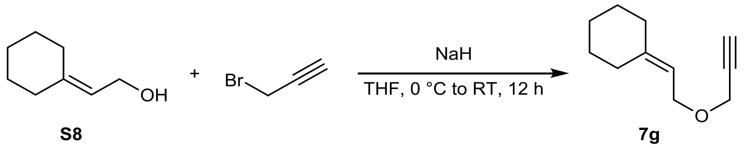

2-Cyclohexylideneethan-1-ol (**S8**, 440 mg, 3.49 mmol) was added to a solution of NaH (267 mg, 60% in paraffin oil, 6.68 mmol) in THF (25 mL) at 0 °C under N_2. After 30 min, a solution of propargyl bromide (0.45 mL, 80% in toluene, 4.0 mmol) in THF (10 mL) was added over 5 min, the mixture was warmed to room temperature and stirred for 12 h. The reaction was quenched carefully with ice-cooled water and extracted with Et_2O (2 × 25 mL). The extracts were washed with H_2O (10 mL), brine (10 mL), dried ($NaSO_4$), filtered, and concentrated *in vacuo*. Purification of the residue by column chromatography (10% Et_2O/petrol) gave **7g** as a yellow oil (490 mg, 85%). IR 3305, 2927, 2853, 2114 (C≡C), 1667, 1446, 1338, 1157, 1071, 935, 826, 659, 625 cm^{-1}; ^{1}H NMR (400 MHz, $CDCl_3$) δ 5.30–5.25 (1H, m, CH_2C**H**=C), 4.14 (2H, d, *J* = 2.4 Hz, C**H**$_2$C≡C), 4.09 (2H, d, *J* = 7.1 Hz, CH_2C**H**=C), 2.42 (1H, t, *J* = 2.4 Hz, C≡C**H**), 2.24–2.20 (2H, m, C**H**$_2$C=CH), 2.15–2.12 (2H, m, C**H**$_2$C=CH), 1.59–1.53 (6H, m, CH_2(C**H**$_2$)$_3$$CH_2$); ^{13}C NMR (101 MHz, $CDCl_3$) δ 146.1 (C), 116.7 (CH), 80.0 (C), 73.9 (CH), 64.9 (CH_2), 56.4 (CH_2), 37.0 (CH_2), 28.9 (CH_2), 28.3 (CH_2), 27.7 (CH_2), 26.6 (CH_2).

1-(2-Methylprop-1-en-1-yl)-1-(prop-2-yn-1-yloxy)cyclohexane (7h)

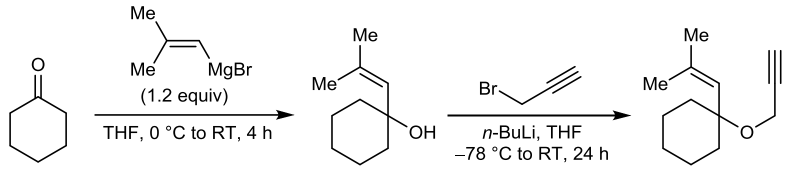

To a solution of cyclohexanone (2.00 g, 20.4 mmol) in THF (50 mL) at 0 °C was added a solution of 2-methyl-1-propenylmagnesium bromide (0.5 M in THF, 49 mL, 24.5 mmol) over 10 min. The mixture was warmed to room temperature, stirred for 4 h, and then quenched carefully with saturated aqueous

NH_4Cl solution (50 mL). The mixture was extracted with Et_2O (2 × 40 mL) and the combined organic layers were washed with H_2O (20 mL) and brine (20 mL), dried ($NaSO_4$), filtered, and concentrated *in vacuo* to leave alcohol **S9** (2.62 g, 85%) which was used directly in the next step without further purification.

Alcohol **S9** was dissolved in THF (60 mL), the solution was cooled to –78 ºC, and then *n*-BuLi (2.5 M in hexanes, 8.1 mL, 20.3 mmol) was added dropwise over 5 min. The solution was stirred at –78 ºC for 15 min, at 0 °C for 30 min, and then cooled back to –78 ºC before propargyl bromide (2.83 mL, 80% in toluene, 25.3 mmol) was added dropwise over 5 min. The mixture was stirred for 24 h while being allowed to warm gradually to room temperature. The reaction was quenched carefully with saturated aqueous NH_4Cl solution (50 mL), and extracted with Et_2O (2 × 40 mL). The combined organic layers were dried ($NaSO_4$), filtered, and concentrated *in vacuo*. Purification of the residue by column chromatography (5–10% Et_2O/petrol) gave **7h** as a colorless oil (89 mg, 2.3%). R_f = 0.50 (10% Et_2O/petrol); IR 3309, 2928, 2856, 1662, 1447, 1376, 1260, 1164, 1149, 1063, 621, cm^{-1}; ^{1}H NMR (400 MHz, $CDCl_3$) δ 4.97 (1H, hept, *J* = 1.5 Hz, =C**H**), 3.96 (2H, d, *J* = 2.5 Hz, OC**H**$_2$C≡C), 2.34 (1H, t, *J* = 2.5 Hz, OCH_2C≡C**H**), 1.84 (3H, d, *J* = 1.3 Hz, C**H**$_3$), 1.76–1.70 (2H, m, (C**H**$_2$)$_5$), 1.73 (3H, d, *J* = 1.3 Hz, C**H**$_3$), 1.65 (2H, ddd, *J* = 12.5, 8.0, 3.5 Hz, (C**H**$_2$)$_5$), 1.57–1.51 (2H, m, (C**H**$_2$)$_5$), 1.45–1.30 (4H, m, (C**H**$_2$)$_5$); ^{13}C NMR (101 MHz, $CDCl_3$) δ 137.2 (C), 126.5 (CH), 82.0 (C), 77.6 (C), 72.5 (CH), 49.8 (CH_2), 36.5 (2 x CH_2), 27.1 (CH_3), 25.6 (CH_2), 22.3 (2 x CH_2), 18.7 (CH_3).

4-Methyl-*N*-(4-methylpent-3-en-1-yl)-*N*-(prop-2-yn-1-yl)benzenesulfonamide (7k)

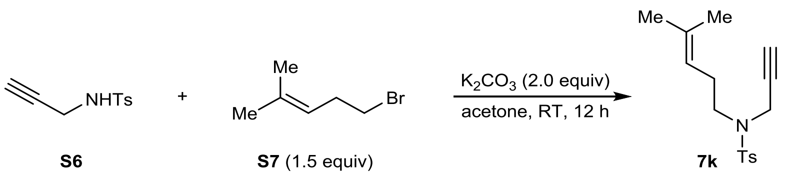

To a solution of 4-methyl-*N*-(prop-2-yn-1-yl)benzenesulfonamide (**S6**, 2.00 g, 9.56 mmol) and 5-bromo-2-methylpent-2-ene **S7** (2.34 g, 14.4 mmol) in acetone (50 mL) at room temperature was added K_2CO_3 (2.64 g, 19.1 mmol) in one portion. The mixture was stirred for 12 h, filtered, and concentrated *in vacuo*. Purification of the residue by column chromatography (20% Et_2O/petrol) gave **7k** as a colorless oil (1.11 g, 40%). R_f = 0.35 (20% Et_2O/petrol); IR 3274, 2970, 2922, 2869, 1739, 1598, 1448, 1331, 1157, 1091, 905, 833, 702, 657, 575, 545 cm^{-1}; ^{1}H NMR (400 MHz, $CDCl_3$) δ 7.78–7.70 (2H, m, Ar**H**), 7.34–7.25 (2H, m, Ar**H**), 5.09 (1H, app tdq, *J* = 7.2, 2.9, 1.5 Hz, CH_2C**H**=C), 4.17 (2H, d, *J* = 2.5 Hz, C**H**$_2$C≡C), 3.23–3.16 (2H, m, C**H**$_2$$CH_2$CH=C), 2.44 (3H, s, ArC**H**$_3$), 2.29 (2H, q, *J* = 7.4 Hz, C**H**$_2$CH=C), 2.06 (1H, t, *J* = 2.5 Hz, CH_2C≡C**H**), 1.71 (3H, d, *J* = 1.4 Hz, C**H**$_3$), 1.63 (3H, d, *J* = 1.3 Hz,

C**H**3); ^{13}C NMR (101 MHz, $CDCl_3$) δ 143.3 (C), 135.9 (C), 134.5 (C), 129.4 (2 x CH), 127.6 (2 x CH), 119.8 (CH), 76.7 (C), 73.6 (CH), 46.0 (CH_2), 36.3 (CH_2), 26.7 (CH_2), 25.6 (CH_3), 21.4 (CH_3), 17.7 (CH_3); HRMS (ESI) Exact mass calculated for $[C_{16}H_{21}NSO_2Na]^+$ $[M+Na]^+$: 314.1185, found: 314.1190.

Sulfonylative Cyclizations of 1,6-Enynes: General Procedure B

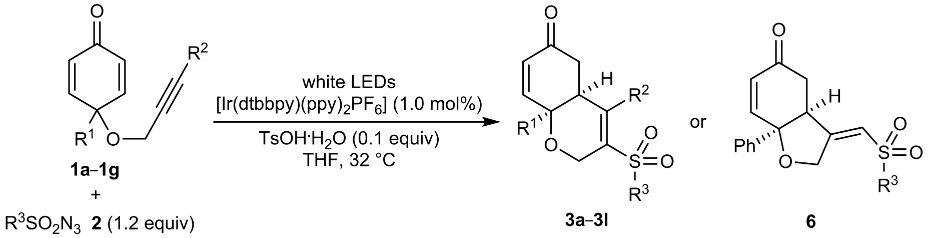

An oven-dried microwave tube equipped with a stirrer bar was charged with $[Ir(dtbbpy)(ppy)_2]PF_6$ (1.8 mg, 0.002 mmol), *p*-toluenesulfonic acid monohydrate (3.8 mg, 0.02 mmol) and the cyclohexadienone-containing 1,6-enyne **1** (0.20 mmol). The tube was sealed with a septum-lined cap and purged with nitrogen for 1 h. A solution of the sulfonyl azide **2** (0.24–1.00 mmol) in THF (2.5 mL) was then added. The tube was then immersed sideways into a sand bath heated to 32 °C, and irradiated by white LEDs (see General Information for details of the light source) for 24–56 h with stirring (see photograph below).

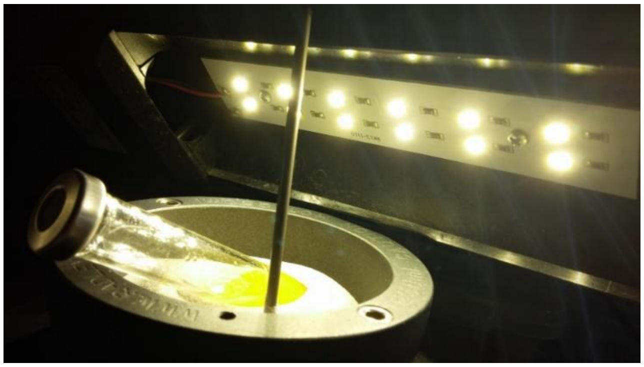

After the 1,6-enyne was completely consumed (monitored by TLC), the crude mixture was directly purified by flash column chromatography on silica gel (EtOAc/petrol) to give the products **3** or **6**.

Caution! The proposed mechanism of these reactions involves the formation of azide **16**, which, being a low-molecular weight organic azide, could be an explosion hazard (see *Angew. Chem. Int. Ed.* **2005**, *44*, 5188 and references cited therein). Although no problems were encountered in this study, appropriate precautions should be taken.

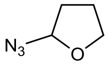

16

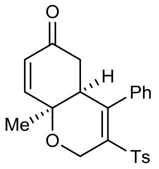

(±)-(4a*S*,8a*S*)-8a-Methyl-4-phenyl-3-tosyl-4a,5-dihydro-2*H*-chromen-6(8a*H*)-one (3a). The title compound was prepared according to General Procedure B from enyne **1a** (47.6 mg, 0.20 mmol) and 4-methylbenzenesulfonyl azide **2a** (47.3 mg, 0.24 mmol) for a reaction time of 24 h and purified by column chromatography (25% EtOAc/petrol) to give a colorless solid (71.8 mg, 91%). R_f = 0.40 (33% EtOAc/petrol); m.p. 119–121 °C (petrol); IR 2975, 1686 (C=O), 1373, 1316, 1148, 1112, 1086, 666, 542 cm^{-1}; ^1H NMR (400 MHz, $CDCl_3$) δ 7.36–7.20 (5H, m, Ar**H**), 7.11 (2H, d, *J* = 8.0 Hz, Ar**H**), 6.85 (2H, br s, Ar**H**), 6.75 (1H, d, *J* = 10.2 Hz, C**H**=CHC=O), 6.07 (1H, d, *J* = 10.2 Hz, C**H**C=O), 4.70 (H, dd, *J* = 17.5, 2.3 Hz, OC**H**$_2$), 4.68 (H, dd, *J* = 17.5, 2.3 Hz, OC**H**$_2$), 2.74–2.60 (1H, m, C**H**CH_2), 2.46–2.31 (2H, m, C**H**$_2$C=O), 2.35 (3H, s, ArC**H**$_3$), 1.52 (3H, s, OCC**H**$_3$); ^{13}C NMR (101 MHz, $CDCl_3$) δ 196.5 (C), 150.1 (CH), 148.0 (C), 144.0 (C), 137.6 (C), 136.8 (C), 135.1 (C), 130.2 (2 x CH), 129.2 (2 x CH), 128.1 (CH), 127.7 (2 x CH), 127.2 (2 x CH), 126.2 (CH), 70.3 (C), 61.1 (CH_2), 45.9 (CH), 37.9 (CH_2), 23.4 (CH_3), 21.4 (CH_3); HRMS (ESI) Exact mass calculated for $[C_{23}H_{22}SO_4Na]^+$ $[M+Na]^+$: 417.1131, found: 417.1135.

Slow diffusion of petrol into a solution of **3a** in CH_2Cl_2 gave crystals that were suitable for X-ray diffraction:

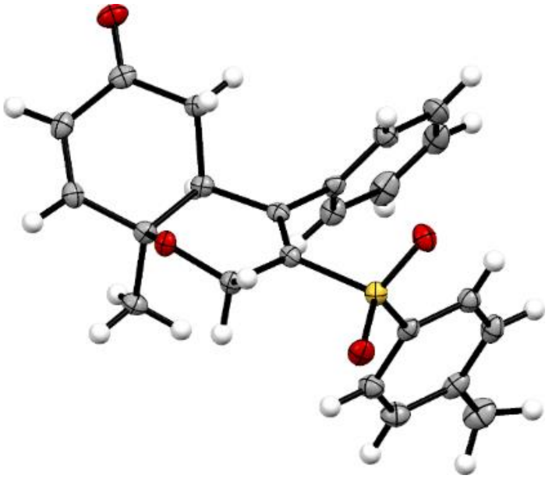

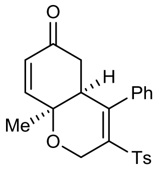

3a
CCDC 1492585

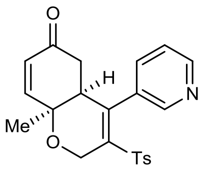

(±)-(4a*S*,8a*S*)-8a-Methyl-4-(pyridin-3-yl)-3-tosyl-4a,5-dihydro-2*H*-chromen-6(8a*H*)-one (3b). The title compound was prepared according to General Procedure B from enyne **1b** (47.8 mg, 0.20 mmol) and 4-methylbenzenesulfonyl azide **2a** (47.3 mg, 0.24 mmol) for a reaction time of 56 h and purified by column chromatography (50% EtOAc/petrol) to give a colorless gum (58.5 mg, 74%). R_f = 0.35 (67% EtOAc/petrol); IR 2974, 2933, 2362, 1686 (C=O), 1411, 1317, 1150, 1112, 813, 670 cm^{-1}; ^1H NMR (400 MHz, $CDCl_3$) δ 8.58 (1H, d, *J* = 3.7 Hz, Ar**H**), 8.01 (1H, s, Ar**H**), 7.36 (1H, br s, Ar**H**), 7.30–7.24 (3H, m, Ar**H**), 7.21–7.17 (2H, m, Ar**H**), 6.74 (1H, d, *J* = 10.2 Hz, C**H**=CHC=O), 6.07 (1H, d, *J* = 10.2 Hz, C**H**C=O), 4.69 (1H, dd,

J = 17.7, 2.2 Hz, OC**H**2), 4.67 (1H, dd, *J* = 17.7, 2.2 Hz, OC**H**2), 2.71–2.68 (1H, m, C**H**CH2), 2.46–2.39 (1H, m, C**H**2C=O), 2.32 (1H, dd, *J* = 16.2, 7.6 Hz, C**H**2C=O), 2.40 (3H, s, ArC**H**3), 1.51 (3H, s, OCC**H**3); ^{13}C NMR (101 MHz, $CDCl_3$) δ 196.0 (C), 150.0 (CH), 149.4 (CH), 144.9 (C), 144.4 (C), 139.2 (C), 137.3 (C), 131.5 (C), 130.4 (CH), 129.8 (CH), 129.7 (2 x CH), 128.6 (CH), 127.2 (2 x CH), 122.7 (CH), 70.7 (C), 61.3 (CH_2), 46.1 (CH), 37.9 (CH_2), 23.6 (CH_3), 21.6 (CH_3); HRMS (ESI) Exact mass calculated for $[C_{22}H_{21}NSO_4Na]^+$ $[M+Na]^+$: 418.1083, found: 418.1085.

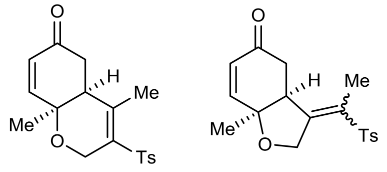

3c S10

(±)-(4a*S*,8a*S*)-4,8a-Dimethyl-3-tosyl-4a,5-dihydro-2*H*-chromen-6(8a*H*)-one (3c). The title compound was prepared according to General Procedure B from enyne **1c** (35.2 mg, 0.2 mmol) and 4-methylbenzenesulfonyl azide **2a** (47.3 mg, 0.24 mmol) for a reaction time of 48 h. Purification by column chromatography (25% EtOAc/petrol) gave a 5:1 mixture of **3c**:**S10** as a colorless gum (49.8 mg, 75%). R_f = 0.50 (33% EtOAc/petrol); IR 2975, 1685, 1643 (C=O), 1302, 1144, 1092, 666, 610, 544 cm^{-1}.

Data for **3c**: ^{1}H NMR (400 MHz, $CDCl_3$) δ 7.74 (2H, d, *J* = 8.2 Hz, Ar**H**), 7.35 (2H, d, *J* = 8.2 Hz, Ar**H**), 6.65 (1H, d, *J* = 10.2 Hz, C**H**=CHC=O), 6.01 (1H, d, *J* = 10.2 Hz, C**H**C=O), 4.47 (2H, s, OC**H**2), 2.65 (1H, dd, *J* = 16.0, 5.0 Hz, C**H**2C=O), 2.60-2.46 (2H, C**H**2C=O and C**H**CH2), 2.46 (3H, s, ArC**H**3), 2.15 (3H, t, *J* = 2.0 Hz, SC=CC**H**3), 1.40 (3H, s, OCC**H**3); ^{13}C NMR (101 MHz, $CDCl_3$) δ 196.7 (C), 150.3 (CH), 145.3 (C), 144.6 (C), 138.2 (C), 133.5 (C), 130.0 (CH), 129.9 (2 x CH), 126.9 (2 x CH), 70.2 (C), 61.4 (CH_2), 45.6 (CH), 38.3 (CH_2), 23.3 (CH_3), 21.6 (CH_3), 18.3 (CH_3); HRMS (ESI) Exact mass calculated for $[C_{18}H_{20}SO_4Na]^+$ $[M+Na]^+$: 355.0975, found: 355.0980.

Characteristic signals for **S10**: ^{1}H NMR (400 MHz, $CDCl_3$) δ 7.69 (2H, d, *J* = 8.2 Hz, Ar**H**), 6.63 (1H, d, *J* = 10.2 Hz, C**H**=CHC=O), 6.32 (1H, d, *J* = 10.2 Hz, C**H**C=O), 5.10 (1H, dq, *J* = 16.7, 1.9 Hz, OC**H**2), 4.91 (1H, dquin, *J* = 16.7, 2.1 Hz, OC**H**2), 3.09 (1H, t, *J* = 7.2 Hz, C**H**CH2), 1.91 (3H, td, *J* = 2.0, 1.0 Hz, SCC**H**3), 1.42 (3H, s, OCC**H**3).

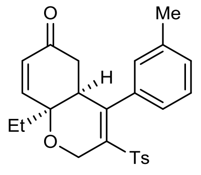

(±)-(4a*S*,8a*S*)-8a-Ethyl-4-(*m*-tolyl)-3-tosyl-4a,8a-dihydro-2*H*-chromen-6(5*H*)-one (3d). The title compound was prepared according to General Procedure B from enyne **1d** (53.3 mg, 0.2 mmol) and 4-methylbenzenesulfonyl azide **2a** (118 mg, 0.6 mmol) for a reaction time of 48 h and purified by column chromatography (40% EtOAc/petrol) to give a colorless gum (52.4 mg, 62%). R_f = 0.25 (33% EtOAc/petrol); IR 2921, 1704 (C=O), 1292, 1145, 1090, 890, 791, 665 cm^{-1}; ^{1}H NMR (400 MHz, $CDCl_3$) δ 7.20 (2H, d, *J* = 8.4 Hz,

ArH), 7.14 (1H, t, *J* = 7.6 Hz, ArH), 7.10-7.08 (3H, m, ArH), 6.82 (1H, d, *J* = 10.3 Hz, CH=CHC=O), 6.69 (1H, br s, ArH), 6.43 (1H, br s, ArH), 6.10 (1H, d, *J* = 10.3 Hz, CHC=O), 4.76–4.66 (2H, m, OCH_2), 2.72–2.69 (1H, m, $CHCH_2$), 2.45–2.31 (2H, m, $CHCH_2$), 2.38 (3H, s, $ArCH_3$), 2.14 (3H, s, $ArCH_3$), 1.86 (2H, q, *J* = 7.5 Hz, CH_2CH_3), 1.05 (3H, t, *J* = 7.5 Hz, CH_2CH_3); ^{13}C NMR (101 MHz, $CDCl_3$) δ 196.8 (C), 149.2 (CH), 148.4 (C), 143.9 (C), 138.0 (C), 137.5 (C), 136.9 (C), 135.2 (C), 131.2 (2 x CH), 129.1 (3 x CH), 128.9 (2 x CH), 127.8 (CH), 127.5 (CH), 72.7 (C), 61.2 (CH_2), 43.8 (CH), 38.0 (CH_2), 29.2 (CH_2), 21.5 (CH_3), 21.2 (CH_3), 7.8 (CH_3); HRMS (ESI) Exact mass calculated for $[C_{25}H_{26}SO_4Na]^+$ $[M+Na]^+$: 445.1444, found: 445.1437.

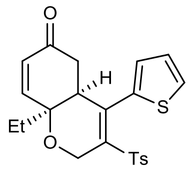

(±)-(4a*S*,8a*S*)-8a-Ethyl-4-(thiophen-2-yl)-3-tosyl-4a,8a-dihydro-2*H*-chromen-6(5)-one (3e). The title compound was prepared according to General Procedure B from enyne **1e** (51.6 mg, 0.20 mmol) and 4-methylbenzenesulfonyl azide **2a** (47.3 mg, 0.24 mmol) for a reaction time of 48 h and purified by column chromatography (25% EtOAc/petrol) to give a colorless solid (45.6 mg, 55%). R_f = 0.38 (33% EtOAc/petrol); m.p. 124–126 °C (petrol); IR 2966, 2203, 1709, 1687 (C=O), 1314, 1148, 706, 674 cm^{-1}; ^{1}H NMR (400 MHz, $CDCl_3$) δ 7.35–7.29 (3H, m, ArH), 7.14 (2H, d, *J* = 8.0 Hz, ArH), 7.03–6.97 (2H, m, ArH), 6.81 (1H, d, *J* = 10.3 Hz, CH=CHC=O), 6.13 (1H, d, *J* = 10.3 Hz, CHC=O), 4.74 (2H, d, *J* = 2.0 Hz, OCH_2), 2.77–2.73 (1H, m, $CHCH_2$), 2.45 (1H, dd, *J* = 16.5, 5.3 Hz, CH_2C=O), 2.42-2.36 (1H, m, CH_2C=O), 2.40 (3H, s, $ArCH_3$), 1.85 (2H, q, *J* = 7.5 Hz, CH_2CH_3), 1.05 (3H, t, *J* = 7.5 Hz, CH_2CH_3); ^{13}C NMR (101 MHz, $CDCl_3$) δ 196.6 (C), 148.8 (CH), 144.2 (C), 141.4 (C), 140.1 (C), 137.4 (C), 134.9 (C), 131.3 (CH), 131.2 (CH), 129.3 (2 x CH), 127.6 (CH), 127.4 (2 x CH), 127.0 (CH), 73.0 (C), 61.7 (CH_2), 44.7 (CH), 38.6 (CH_2), 29.2 (CH_2), 21.6 (CH_3), 7.8 (CH_3); HRMS (ESI) Exact mass calculated for $[C_{22}H_{22}O_4S_2Na]^+$: 437.0852, found: 437.0846.

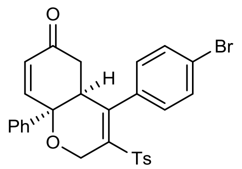

(±)-(4a*S*,8a*S*)-4-(4-Bromophenyl)-8a-phenyl-3-tosyl-4a,5-dihydro-2*H*-chromen-6(8a*H*)-one (3f). The title compound was prepared according to General Procedure B from enyne **1f** (75.8 mg, 0.20 mmol) and 4-methylbenzenesulfonyl azide **2a** (47.3 mg, 0.24 mmol) according to General Procedure A for a reaction time of 36 h and purified by column chromatography (25% EtOAc/petrol) to give a colorless solid (83.5 mg, 78%); m.p. decomposed at 275 °C (petrol). R_f = 0.42 (33% EtOAc/petrol); IR 3060, 1713, 1688 (C=O), 1316, 1151, 1012, 904, 669, 595 cm^{-1}; ^{1}H NMR (400 MHz, $CDCl_3$) δ 7.56–7.35 (7H, m, ArH), 7.08–7.02 (4H, m, ArH), 6.86 (1H, d, *J* = 10.2 Hz, CH=CHC=O), 6.78 (2H, d, *J* = 7.7 Hz, ArH), 6.22 (1H, d, *J* = 10.2 Hz, CHC=O), 4.79 (1H, dd, *J* = 17.7, 2.2 Hz, OCH_2), 4.63 (1H, dd, *J* = 17.7, 2.2

Hz, OC**H**$_2$), 3.26–3.21 (1H, m, C**H**CH$_2$), 2.47–2.42 (2H, m, C**H**$_2$C=O), 2.40 (3H, s, C**H**$_3$); ^{13}C NMR (101 MHz, CDCl$_3$) δ 196.6 (C), 149.0 (CH), 147.0 (C), 144.3 (C), 139.2 (C), 138.0 (C), 137.5 (C), 133.9 (C), 131.2 (2 x CH), 131.0 (CH), 129.2 (2 x CH), 129.1 (2 x CH), 128.8 (CH), 127.2 (2 x CH), 126.1 (2 x CH), 122.9 (C), 75.0 (C), 62.0 (CH$_2$), 44.8 (CH), 37.9 (CH$_2$), 21.6 (CH$_3$), one signal could not be identified, most likely due to it being broad; HRMS (ESI) Exact mass calculated for [$C_{28}H_{23}$Br sO_4Na]$^+$ [M+Na]$^+$: 557.0393, found: 557.0368.

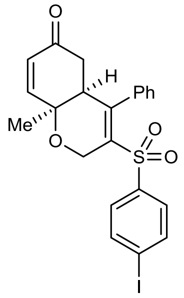

(±)-(4a*S*,8a*S*)-3-[(4-Iodophenyl)sulfonyl]-8a-methyl-4-phenyl-4a,5-dihydro-2*H*-chromen-6(8a*H*)-one (3g). The title compound was prepared according to General Procedure C from enyne **1a** (47.6 mg, 0.20 mmol) and 4-iodobenzenesulfonyl azide **2b** (74.2 mg, 0.24 mmol) for a reaction time of 36 h and purified by column chromatography (25% EtOAc/petrol) to give a colorless solid (83.0 mg, 82%). R_f = 0.42 (33% EtOAc/petrol); m.p. 161–164 °C (petrol); IR 3083, 2978, 1684 (C=O), 1314, 1148, 1005, 701, 576 cm^{-1}; ^{1}H NMR (400 MHz, CDCl$_3$) 7.69–7.59 (2H, m, Ar**H**), 7.39–7.19 (3H, m, Ar**H**), 7.05–6.98 (2H, m, Ar**H**), 6.81 (2H, br s, Ar**H**), 6.76 (1H, dd, *J* = 10.2, 5.3 Hz, C**H**=CHC=O), 6.08 (1H, d, *J* = 10.2 Hz, C**H**C=O), 4.76 (1H, dd, *J* = 17.5, 2.2 Hz, OC**H**$_2$), 4.71 (1H, dd, *J* = 17.5, 2.2, OC**H**$_2$), 2.72–2.67 (1H, m, CHC**H**$_2$), 2.42 (1H, dd, *J* = 16.4, 5.3 Hz, C**H**$_2$C=O), 2.35 (1H, dd, *J* = 16.4, 7.8 Hz, C**H**$_2$C=O), 1.53 (3H, s, C**H**$_3$); ^{13}C NMR (101 MHz, CDCl$_3$) δ 196.4 (C), 150.0 (CH), 148.9 (C), 140.4 (C), 137.9 (2 x CH), 136.9 (C), 135.0 (CH), 130.5 (2 x CH), 128.6 (2 x CH), 128.5 (CH), 128.1 (2 x CH), 109.9 (C), 101.0 (C), 70.6 (C), 61.1 (CH$_2$), 46.1 (CH), 38.1 (CH$_2$), 23.7 (CH$_3$); HRMS (ESI) Exact mass calculated for $C_{22}H_{19}ISO_4Na$ [M+Na]$^+$: 528.9941, found: 528.9936.

Slow diffusion of petrol into a solution of **3g** in CH_2Cl_2 gave crystals that were suitable for X-ray diffraction:

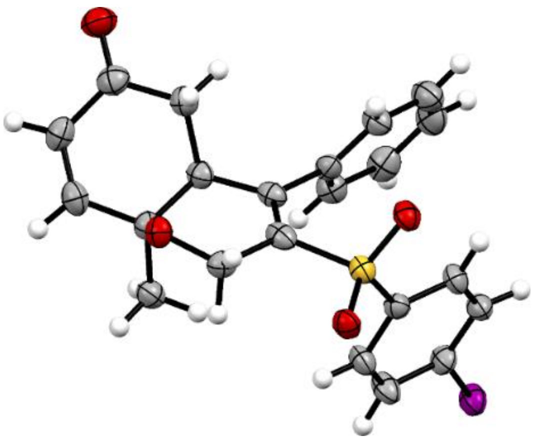

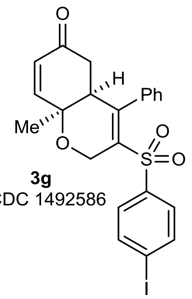

3g
CCDC 1492586

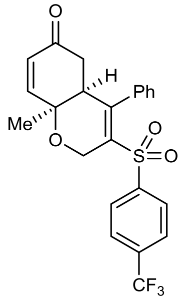

(±)-(4a*S*,8a*S*)-8a-Methyl-4-phenyl-3-{[4-(trifluoromethyl)phenyl]sulfonyl}-4a,5-dihydro-2*H*-chromen-6(8a*H*)-one (3h). The title compound was prepared according to General Procedure B from enyne **1a** (47.6 mg, 0.20 mmol) and 4-(trifluoromethyl)benzenesulfonyl azide **2c** (60.2 mg, 0.24 mmol) for a reaction time of 24 h and purified by column chromatography (25% EtOAc/petrol) to give a colorless solid (80.7 mg, 90%). R_f = 0.30 (33% EtOAc/petrol); m.p. 144–146 °C (petrol); IR 2924, 2854, 2196, 1685 (C=O), 1604, 1507, 1244, 1165, 1081, 1068, 1018, 761, 699, 674, 567, 542 cm^{-1}; ^{1}H NMR (400 MHz, $CDCl_3$) δ 7.54 (2H, d, *J* = 8.3 Hz, Ar**H**), 7.42 (2H, d, *J* = 8.3 Hz, Ar**H**), 7.34–7.29 (1H, m, Ar**H**), 7.21 (2H, br s, Ar**H**), 6.98–6.55 (2H, m, Ar**H**), 6.77 (1H, d, *J* = 10.2 Hz, C**H**=CHC=O), 6.10 (1H, d, *J* = 10.2 Hz, C**H**C=O), 4.78 (2H, d, *J* = 2.1 Hz, OC**H**$_2$), 2.73–2.69 (1H, m, C**H**CH$_2$), 2.47 (1H, dd, *J* = 16.4, 5.3 Hz, C**H**$_2$C=O), 2.34 (1H, dd, *J* = 16.4, 7.7 Hz, C**H**$_2$C=O), 1.55 (3H, s, C**H**$_3$); ^{13}C NMR (101 MHz, $CDCl_3$) δ 196.3 (C), 150.0 (CH), 149.5 (C), 144.2 (C), 136.9 (C), 134.6 (C, q, *J* = 32.9 Hz), 134.4 (C), 130.5 (CH), 128.6 (2 x CH), 128.1 (2 x CH), 127.9 (3 x CH), 125.6 (2 x CH, q, *J* = 3.7 Hz), 122.9 (C, q, *J* = 273.6 Hz), 70.7 (C), 61.0 (CH_2), 46.1 (CH), 38.1 (CH_2), 23.7 (CH_3); HRMS (ESI) Exact mass calculated for $[C_{23}H_{19}F_3SO_4Na]^+$ $[M+Na]^+$: 471.0848, found: 471.0841.

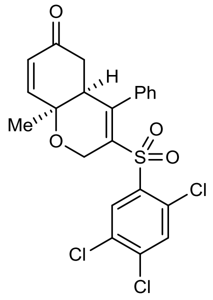

(±)-(4a*S*,8a*S*)-8a-Methyl-4-phenyl-3-[(2,4,5-trichlorophenyl)sulfonyl]-4a,5-dihydro-2*H*-chromen-6(8a*H*)-one (3i). The title compound was prepared according to General Procedure B from enyne **1a** (47.6 mg, 0.20 mmol) and 2,4,5-trichlorobenzenesulfonyl azide **2d** (68.8 mg, 0.24 mmol) for a reaction time of 24 h and purified by column chromatography (25% EtOAc/petrol) to give a colorless solid (85.1 mg, 88%). R_f = 0.38 (33% EtOAc/petrol); m.p. 220–222 °C (petrol); IR 3281, 1679 (C=O), 1440, 1355, 1169, 1068, 688, 538 cm^{-1}; ^{1}H NMR (400 MHz, $CDCl_3$) δ 7.45 (1H, s, Ar**H**), 7.25 (1H, t, *J* = 7.4 Hz Ar**H**), 7.09 (1H, s, Ar**H**), 7.05 (3H, br s, Ar**H**), 6.78 (1H, d, *J* = 10.2 Hz, C**H**=CHC=O), 6.56 (1H, br s, Ar**H**), 6.09 (1H, d, *J* = 10.2 Hz, C**H**C=O), 4.87 (1H, dd, *J* = 17.4, 2.2 Hz, OC**H**$_2$), 4.78 (1H, dd, *J* = 17.4, 2.1 Hz, OC**H**$_2$), 2.76–2.71 (1H, m, C**H**CH$_2$), 2.48–2.36 (2H, m, C**H**$_2$C=O), 1.63 (3H, s, C**H**$_3$); ^{13}C NMR (101 MHz, $CDCl_3$) δ 196.5 (C), 150.2 (CH), 148.3 (C), 138.2 (C), 137.4 (C), 136.4 (C), 134.0 (C), 132.3 (2 x CH), 131.9 (2 x CH), 131.8 (C), 130.4 (C), 130.3 (2 x CH), 128.9 (CH), 127.7 (CH), 70.6 (C), 60.3 (CH_2), 45.5 (CH), 38.0 (CH_2), 23.9 (CH_3); HRMS (ESI) Exact mass calculated for $[C_{22}H_{17}Cl_3SO_4Na]^+$ $[M+Na]^+$: 504.9805, found: 504.9798.

Slow diffusion of petrol into a solution of **3i** in CH_2Cl_2 gave crystals that were suitable for X-ray diffraction:

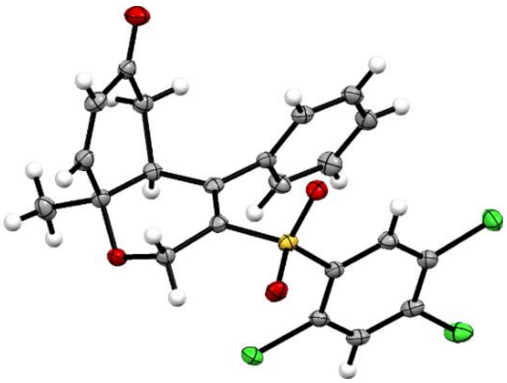

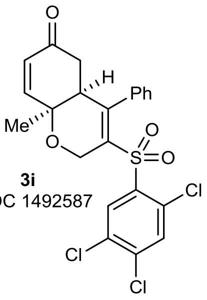

3i
CCDC 1492587

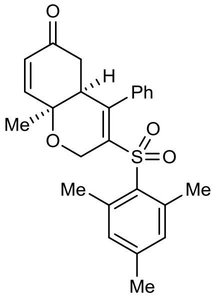

(±)-(4a*S*,8a*S*)-3-(Mesitylsulfonyl)-8a-methyl-4-phenyl-4a,5-dihydro-2*H*-chromen-6(8a*H*)-one (3j). The title compound was prepared according to General Procedure B from enyne **1a** (47.6 mg, 0.20 mmol) and 2,4,6-trimethylbenzenesulfonyl azide **2e** (54.0 mg, 0.24 mmol) for a reaction time of 56 h and purified by column chromatography (25% EtOAc/petrol) to give a colorless solid (57.5 mg, 68%). R_f = 0.45 (33% EtOAc/petrol); m.p. 142–144 °C (petrol); IR 2973, 1686 (C=O), 1300, 1141, 1109, 789, 769, 659 cm^{-1}; ^{1}H NMR (400 MHz, $CDCl_3$) δ 7.18–7.10 (1H, m, Ar**H**), 7.05 (2H, t, *J* = 7.5 Hz, Ar**H**), 6.83 (1H, d, *J* = 10.2 Hz, C**H**=CHC=O), 6.67 (4H s, Ar**H**), 6.11 (1H, d, *J* = 10.2 Hz, C**H**C=O), 4.78 (H, dd, *J* = 17.4, 2.2 Hz, OC**H**$_2$), 4.68 (H, dd, *J* = 17.4, 2.5 Hz, OC**H**$_2$), 2.76–2.73 (1H, m, C**H**CH_2), 2.45 (1H, dd, *J* = 16.5, 5.4 Hz, C**H**$_2$C=O), 2.36-2.29 (1H, m, C**H**$_2$C=O), 2.29 (6H, s, 2 x ArC**H**$_3$), 2.23 (3H s, ArC**H**$_3$), 1.60 (3H, s, OCC**H**$_3$); ^{13}C NMR (101 MHz, $CDCl_3$) δ 196.7 (C), 151.2 (CH), 144.8 (C), 142.9 (C), 139.4 (2 x C), 139.1 (C), 134.7 (C), 134.4 (C), 131.7 (2 x CH), 130.6 (2 x CH), 127.84 (2 x CH), 127.79 (CH), 71.3 (C), 60.3 (CH_2), 45.9 (CH), 37.8 (CH_2), 24.2 (CH_3), 21.9 (2 x CH_3), 20.8 (CH_3); HRMS (ESI) Exact mass calculated for $[C_{25}H_{26}SO_4Na]^+$ $[M+Na]^+$: 445.1444, found: 445.1444.

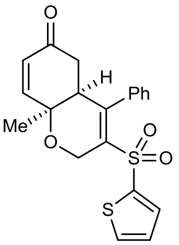

(±)-(4a*S*,8a*S*)-8a-Methyl-4-phenyl-3-(thiophen-2-ylsulfonyl)-4a,5-dihydro-2*H*-chromen-6(8a*H*)-one (3k). The title compound was prepared according to General Procedure B from enyne **1a** (47.6 mg, 0.20 mmol) and thiophene-2-sulfonyl azide **2f** (45.4 mg, 0.24 mmol) for a reaction time of 36 h and purified by column chromatography (25% EtOAc/petrol) to give a colorless solid (62.6 mg, 81%). R_f = 0.45 (33% EtOAc/petrol); m.p. 128–130 °C (petrol); IR 3101, 2977, 2160, 1964, 1685 (C=O), 1402, 1373, 1322, 1148, 1111, 1090, 1016, 771, 729, 701, 671, 628, 571, 540, 485 cm^{-1}; ^{1}H NMR (400 MHz, $CDCl_3$) δ 7.59 (1H, dd, *J* = 5.0, 1.3 Hz, Ar**H**), 7.39–7.27 (3H, m, Ar**H**), 7.01 (1H, dd, *J* = 3.8, 1.3 Hz, Ar**H**), 6.96 (2H, br s, Ar**H**), 6.90 (1H, dd, *J* = 5.0, 3.8 Hz, Ar**H**), 6.75 (1H, d, *J* = 10.2 Hz,

C**H**=CHC=O), 6.08 (1H, d, *J* = 10.2 Hz, C**H**C=O), 4.77-4.67 (2H, m, OC**H**$_2$), 2.74–2.70 (1H, m, C**H**CH$_2$), 2.43 (1H, dd, *J* = 14.7, 4.0 Hz, C**H**$_2$C=O), 2.38 (1H, dd, *J* = 14.7, 6.1 Hz, C**H**$_2$C=O), 1.54 (3H, s, OCC**H**$_3$); ^{13}C NMR (101 MHz, CDCl$_3$) δ 196.6 (C), 150.0 (CH), 148.9 (C), 141.6 (C), 136.7 (C), 135.2 (C), 134.1 (CH), 133.8 (CH), 132.2 (CH), 130.4 (CH), 128.4 (CH), 128.0 (2 x CH), 127.2 (2 x CH), 70.4 (C), 60.9 (CH$_2$), 46.2 (CH), 38.0 (CH$_2$), 23.5 (CH$_3$); HRMS (ESI) Exact mass calculated for $C_{20}H_{18}S_2O_4Na$ [M+Na]$^+$:409.0539, found: 409.0541.

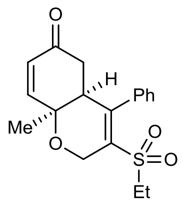

(±)-(4a*S*,8a*S*)-3-(Ethylsulfonyl)-8a-methyl-4-phenyl-4a,5-dihydro-2*H*-chromen-6(8a*H*)-one (3l). The title compound was prepared according to General Procedure B from enyne **1a** (47.6 mg, 0.20 mmol) and ethanesulfonyl azide **2g** (81.1 mg, 0.6 mmol) for a reaction time of 56 h and purified by column chromatography (25% EtOAc/petrol) to give a colorless solid (43.2 mg, 65%). R_f = 0.50 (33% EtOAc/petrol); m.p. 102–104 °C (petrol); IR 2978, 2938, 1686, 1639 (C=O), 1308, 1277, 1141, 1129, 1091, 888, 774, 703, 532, 503 cm^{-1}; ^{1}H NMR (400 MHz, CDCl$_3$) δ 7.51–7.35 (3H, m, Ar**H**), 7.20 (2H, dd, *J* = 6.4, 2.9 Hz, Ar**H**), 6.77 (1H, d, *J* = 10.2 Hz, C**H**=CHC=O), 6.10 (1H, d, *J* = 10.2 Hz, C**H**C=O), 4.64 (1H, dd, *J* = 17.7, 2.2 Hz, OC**H**$_2$), 4.59 (1H, dd, *J* = 17.7, 2.2 Hz, OC**H**$_2$), 2.89–2.85 (1H, m, C**H**CH$_2$), 2.64–2.55 (1H, m, C**H**$_2$CH$_3$), 2.51–2.39 (3H, m, C**H**$_2$CH$_3$ and C**H**$_2$C=O), 1.61 (3H, s, OCC**H**$_3$), 1.18 (3H, t, *J* = 7.4 Hz, CH$_2$C**H**$_3$); ^{13}C NMR (101 MHz, CDCl$_3$) δ 196.5 (C), 150.2 (CH), 148.3 (C), 135.2 (C), 134.6 (C), 130.4 (2 x CH), 129.0 (CH), 128.4 (2 x CH), 70.6 (C), 61.3 (CH$_2$), 49.3 (CH$_2$), 45.9 (CH), 38.1 (CH$_2$), 23.7 (CH$_3$), 6.8 (CH$_3$), one signal could not be identified, most likely due to it being broad; HRMS (ESI) Exact mass calculated for [$C_{18}H_{20}S_2O_4Na$]$^+$ [M+Na]$^+$: 355.0975, found: 355.0984.

Slow diffusion of petrol into a solution of **3l** in CH_2Cl_2 gave crystals that were suitable for X-ray diffraction:

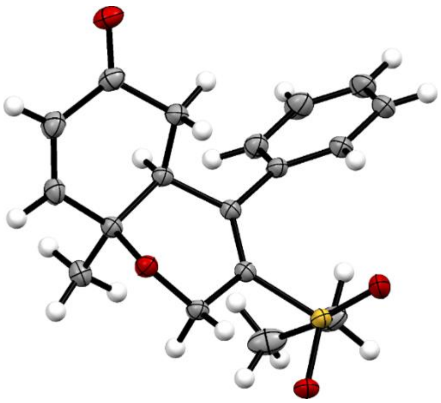

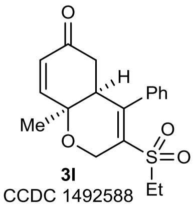

3l
CCDC 1492588

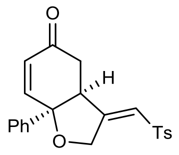

(±)-(3a*S*,7a*S*,Z)-7a-Phenyl-3-(tosylmethylene)-2,3,3a,7a-tetrahydrobenzofuran-5(4*H*)-one (6). The title compound was prepared according to General Procedure B from enyne **1g** (44.9 mg, 0.20 mmol) and 4-methylbenzenesulfonyl azide **2a** (47.3 mg, 0.24 mmol) for a reaction time of 24 h and purified by column chromatography (25% EtOAc/petrol) to give a colorless solid (68.5 mg, 90%). R_f = 0.35 (33% EtOAc/petrol); m.p. 122–125 °C (petrol); IR 3038, 2924, 1687 (C=O), 1337, 1302, 1311, 1302, 1291, 1147, 1086, 1051, 1018, 761, 667, 541 cm^{-1}; ^{1}H NMR (400 MHz, $CDCl_3$) δ 7.78–7.69 (2H, m, Ar**H**), 7.57–7.46 (2H, m, Ar**H**), 7.46–7.31 (5H, m, Ar**H**), 6.68 (1H, dd, *J* = 10.3, 1.8 Hz, C**H**=CHC=O), 6.21 (1H, dd, *J* = 10.3, 1.0 Hz, C**H**C=O), 6.14 (1H, q, *J* = 2.6 Hz, =C**H**S), 5.35 (1H, dd, *J* = 17.6, 2.4 Hz, OC**H**$_2$), 4.90 (1H, dt, *J* = 17.4, 2.5 Hz, OC**H**$_2$), 3.28–3.14 (1H, m, C**H**CH_2), 2.74 (1H, dd, *J* = 17.4, 5.1 Hz, C**H**$_2$C=O), 2.62 (1H, ddd, *J* = 17.4, 2.9, 1.1 Hz, C**H**$_2$C=O), 2.46 (3H, s, C**H**$_3$); ^{13}C NMR (101 MHz, $CDCl_3$) δ 194.7 (C), 158.4 (C), 148.4 (CH), 144.8 (C), 138.2 (C), 137.7 (C), 131.5 (CH), 130.0 (2 x CH), 128.9 (2 x CH), 128.8 (CH), 127.1 (2 x CH), 125.3 (2 x CH), 122.3 (CH), 82.8 (C), 69.0 (CH_2), 51.3 (CH), 35.8 (CH_2), 21.6 (CH_3); HRMS (ESI) Exact mass calculated for $[C_{22}H_{20}SO_4Na]^+$ $[M+Na]^+$: 403.0975, found: 403.0983.

Slow diffusion of petrol into a solution of **6** in CH_2Cl_2 gave crystals that were suitable for X-ray diffraction:

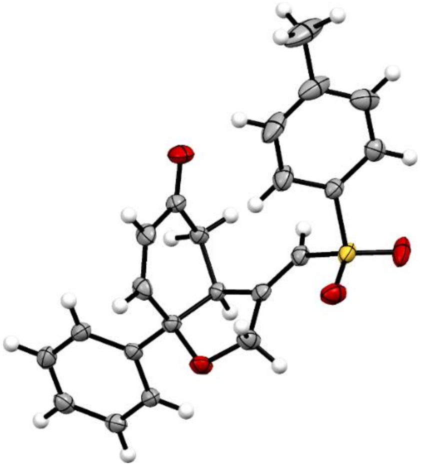

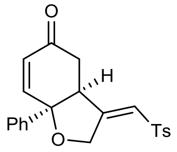

6
CCDC 1492589

Azidosulfonylative Cyclizations of Enynes: General Procedure C

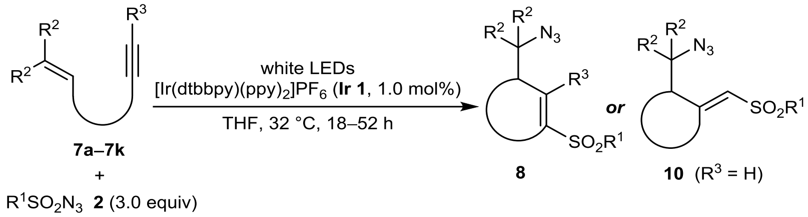

An oven-dried microwave tube equipped with a stirrer bar was charged with [Ir(dtbbpy)(ppy)$_2$]PF$_6$ (3.7 mg, 0.004 mmol) and the enyne **7** (0.40 mmol). The tube was sealed with a septum-lined cap and purged with nitrogen for 1 h. A solution of the sulfonyl azide **2** (1.2 mmol) in THF (2.0 mL) was then added. The tube was then immersed sideways into a sand bath heated to 32 °C, and irradiated by white LEDs (see General Information for details of the light source) for 18–52 h with stirring (see photograph on page S11). After the enyne was completely consumed (monitored by TLC), the crude mixture was directly purified by flash column chromatography on silica gel (EtOAc/petrol) to give the products.

Caution! The proposed mechanism of these reactions involves the formation of azide **16**, which, being a low-molecular weight organic azide, could be an explosion hazard (see *Angew. Chem. Int. Ed.* **2005**, *44*, 5188 and references cited therein). Although no problems were encountered in this study, appropriate precautions should be taken.

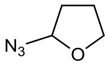

16

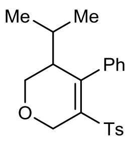

3-Isopropyl-4-phenyl-5-tosyl-3,6-dihydro-2*H*-pyran (9a). The title compound was obtained as a colorless gum; R_f = 0.58 (33% EtOAc/petrol); IR 3017, 2970, 2359, 1739, 1366, 1229, 1216, 1206, 909, 772, 518 cm^{-1}; ^{1}H NMR (400 MHz, CDCl$_3$) δ 7.31–7.13 (6H, m, Ar**H**), 7.10–7.03 (2H, m, Ar**H**), 6.92 (1H, br s, Ar**H**), 4.69 (1H, dd, *J* = 16.6, 1.6 Hz, OC**H**$_2$C=C), 4.44 (1H, dd, *J* = 16.6, 3.0 Hz, OC**H**$_2$C=C), 4.04 (1H, dd, *J* = 11.9, 2.6 Hz, OC**H**$_2$CH), 3.69–3.60 (1H, m, OC**H**$_2$CH), 2.36 (3H, s, ArC**H**$_3$), 2.18 (1H, dt, *J* = 4.2, 1.2 Hz, OCH$_2$C**H**), 1.66-1.58 (1H, m, C**H**(CH$_3$)$_2$), 0.87 (3H, d, *J* = 6.8 Hz, CHC**H**$_3$), 0.85 (3H, d, *J* = 6.8 Hz, CHC**H**$_3$), ^{13}C NMR (101 MHz, CDCl$_3$) δ 150.5 (C), 143.6 (C), 138.3 (C), 138.1 (C), 136.9 (C), 129.1 (2 x CH), 127.9 (CH), 127.6 (2 x CH), 127.4 (2 x CH), 65.3 (CH$_2$), 64.4 (CH$_2$), 47.3 (CH), 28.3 (CH), 21.5 (CH$_3$), 21.2 (CH$_3$), 17.8 (CH$_3$), one signal could not be identified, most likely due to it being broad; HRMS (ESI) Exact mass calculated for [C$_{21}$H$_{24}$SO$_3$Na]$^+$ [M+Na]$^+$: 379.1319, found: 379.1338.

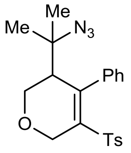

3-(2-Azidopropan-2-yl)-4-phenyl-5-tosyl-3,6-dihydro-2*H*-pyran (8a). The title compound was prepared according to General Procedure C from enyne **7a** (80.1 mg, 0.40 mmol) and 4-methylbenzenesulfonyl azide **2a** (237 mg, 1.20 mmol) for a reaction time of 18 h and purified by column chromatography (15% EtOAc/petrol) to give a colorless solid (103 mg, 65%). R_f = 0.55 (33% EtOAc/petrol); m.p. 116–120 °C (petrol); IR 2970, 2109, 2069, 1738, 1370, 1298, 1208, 1142, 1130, 669, 576, 529 cm^{-1}; ^{1}H NMR (400 MHz, $CDCl_3$) δ 7.59–7.05 (6H, m, Ar**H**), 7.02–7.01 (2H, d, *J* = 8.1 Hz, Ar**H**), 6.46 (1H, br s, Ar**H**), 4.82 (1H, dd, *J* = 17.5, 0.9 Hz, OC**H**$_2$C=C), 4.53 (1H, dd, *J* = 17.5, 2.7 Hz, OC**H**$_2$C=C), 4.35 (1H, dd, *J* = 12.0, 1.1 Hz, OC**H**$_2$CH), 3.50 (1H, dd, *J* = 12.0, 3.4 Hz, OC**H**$_2$CH), 2.34 (3H, s, ArC**H**$_3$), 2.33–2.31 (1H, m, OCH$_2$C**H**), 1.32 (3H, s, C(C**H**$_3$)$_2$), 1.01 (3H, s, C(C**H**$_3$)$_2$); ^{13}C NMR (101 MHz, $CDCl_3$) δ 147.5 (C), 143.7 (C), 140.7 (C), 138.0 (C), 137.9 (C), 129.0 (2 x CH), 128.3 (CH), 127.6 (2 x CH), 127.2 (2 x CH), 65.8 (CH_2), 65.7 (CH_2), 64.3 (C), 50.6 (CH), 26.3 (CH_3), 25.3 (CH_3), 21.5 (CH_3), one signal could not be identified, most likely due to it being broad; HRMS (ESI) Exact mass calculated for $[C_{21}H_{23}SO_3N_3Na]^+$ $[M+Na]^+$: 420.1352, found: 420.1350.

Slow diffusion of petrol into a solution of **8a** in CH_2Cl_2 gave crystals that were suitable for X-ray diffraction:

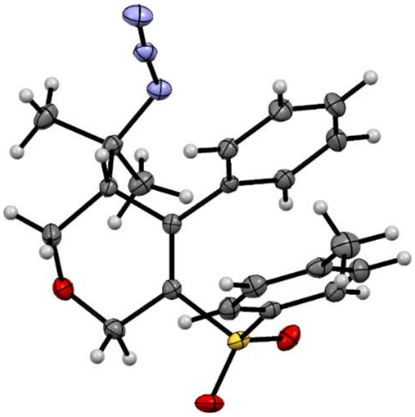

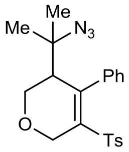

8a
CCDC 1537345

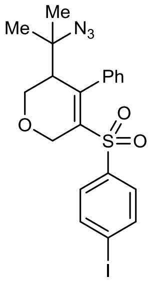

3-(2-Azidopropan-2-yl)-5-[(4-iodophenyl)sulfonyl]-4-phenyl-3,6-dihydro-2*H*-pyran (8b). The title compound was prepared according to General Procedure C from enyne **7a** (80.1 mg, 0.40 mmol) and 4-iodobenzenesulfonyl azide **2b** (371 mg, 1.20 mmol) for a reaction time of 36 h and purified by column chromatography (15% EtOAc/petrol) to give a colorless solid (169 mg, 83%). R_f = 0.45 (33% EtOAc/petrol); m.p. 124–128 °C (petrol); IR 2966, 2933, 2116, 1736, 1308, 1568, 1384, 1299, 1142, 1129, 736, 699, 596, 560, 537 cm^{-1}; ^{1}H NMR (400 MHz, $CDCl_3$) δ 7.61–7.54 (2H, m, Ar**H**), 7.50 (1H, br s, Ar**H**), 7.32–7.19 (2H, m, Ar**H**), 7.10 (1H, br s, Ar**H**), 6.98–6.81 (2H, m, Ar**H**), 6.43 (1H, br s, Ar**H**), 4.86 (1H, dd, *J* =

17.5, 0.9 Hz, OC**H**$_2$C=C), 4.57 (1H, dd, *J* = 17.5, 2.7 Hz, OC**H**$_2$C=C), 4.38 (1H, dd, *J* = 12.1, 1.1 Hz, OC**H**$_2$CH), 3.52 (1H, dd, *J* = 12.1, 3.4 Hz, OC**H**$_2$CH), 2.36–2.34 (1H, m, OCH$_2$C**H**), 1.34 (3H, s, C**H**$_3$), 1.05 (3H, s, C**H**$_3$); ^{13}C NMR (101 MHz, CDCl$_3$) δ 148.4 (C), 140.42 (C), 140.38 (C), 137.8 (C), 137.6 (2 x CH), 128.5 (CH), 128.4 (2 x CH), 127.9 (2 x CH), 100.5 (C), 65.69 (CH$_2$), 65.67 (CH$_2$), 64.2 (C), 50.5 (CH), 26.2 (CH$_3$), 25.3 (CH$_3$), one signal could not be identified, most likely due to it being broad; HRMS (ESI) Exact mass calculated for [$C_{20}H_{20}SO_3N_3INa$]$^+$ [M+Na]$^+$: 532.0162, found: 532.0164.

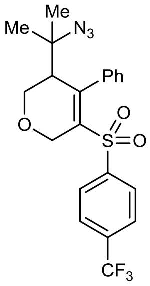

3-(2-Azidopropan-2-yl)-4-phenyl-5-{[4-(trifluoromethyl)phenyl]sulfonyl}-3,6-dihydro-2*H*-pyran (8c). The title compound was prepared according to General Procedure C from enyne **7a** (80.1 mg, 0.40 mmol) and 4-(trifluoromethyl)benzenesulfonyl azide **2c** (301 mg, 1.20 mmol) for a reaction time of 36 h and purified by column chromatography (15% EtOAc/petrol) to give a colorless solid (161 mg, 89%). R$_f$ = 0.45 (33% EtOAc/petrol); m.p. 132–136 °C (petrol); IR 2965, 2117, 2101, 1318, 1298, 1149, 1127, 1061, 701, 603, cm^{-1}; ^{1}H NMR (400 MHz, CDCl$_3$) δ 7.50 (1H, br s, Ar**H**), 7.44 (2H, d, *J* = 8.2 Hz, Ar**H**), 7.40–7.28 (3H, m, Ar**H**), 7.28–7.20 (1H, m, Ar**H**), 6.94 (1H, br s, Ar**H**), 6.30 (1H, br s, Ar**H**), 4.87 (1H, dd, *J* = 17.6, 0.9 Hz, OC**H**$_2$C=C), 4.60 (1H, dd, *J* = 17.6, 2.8 Hz, OC**H**$_2$C=C), 4.37 (1H, dd, *J* = 12.1, 1.1 Hz, OC**H**$_2$CH), 3.52 (1H, dd, *J* = 12.1, 3.5 Hz, OC**H**$_2$CH), 2.33–2.31 (1H, m, OCH$_2$C**H**), 1.32 (3H, s, C**H**$_3$), 1.03 (3H, s, C**H**$_3$); ^{13}C NMR (101 MHz, CDCl$_3$) δ 148.9 (C), 144.2 (C), 140.3 (C), 137.6 (C), 134.2 (C, q, *J* = 33.0 Hz), 128.5 (2 x CH), 127.6 (2 x CH), 126.6 (C, q, *J* = 273.6 Hz), 125.4 (2 x CH, q, *J* = 3.7 Hz), 65.69 (CH$_2$), 65.65 (CH$_2$), 64.1 (C), 50.4 (CH), 26.1 (CH$_3$), 25.3 (CH$_3$), one signal could not be identified, most likely due to it being broad; HRMS (ESI) Exact mass calculated for [$C_{21}H_{20}SO_3N_3F_3Na$]$^+$ [M+Na]$^+$: 474.1070, found: 474.1077.

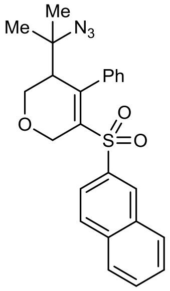

3-(2-Azidopropan-2-yl)-5-(naphthalen-2-ylsulfonyl)-4-phenyl-3,6-dihydro-2*H*-pyran (8d). The title compound was prepared according to General Procedure C from enyne **7a** (80.1 mg, 0.40 mmol) and naphthalene-2-sulfonyl azide **2h** (278 mg, 1.20 mmol) for a reaction time of 36 h and purified by column chromatography (15% EtOAc/petrol) to give a colorless solid (127 mg, 73%). R$_f$ = 0.62 (33% EtOAc/petrol); m.p. 147–151 °C (petrol); IR 2922, 2112, 2076, 1292, 1258, 1142, 1124, 813, 749, 703, 638, 571, 532 cm^{-1}; ^{1}H NMR (400 MHz, CDCl$_3$) δ 7.81 (1H, dd, *J* = 8.1, 1.2 Hz, Ar**H**), 7.75 (1H, d, *J* = 8.7 Hz, Ar**H**), 7.69–7.45 (5H, m, Ar**H**), 7.40 (2H, dd, *J* = 8.7, 1.9 Hz, Ar**H**), 7.12 (1H, tt, *J* = 7.5, 1.3 Hz, Ar**H**), 6.68 (1H, br s, Ar**H**), 6.26 (1H, br s, Ar**H**), 4.92 (1H, dd, *J* = 17.5, 1.0 Hz, OC**H**$_2$C=C), 4.63 (1H, dd, *J* = 17.5, 2.8 Hz, OC**H**$_2$C=C), 4.36 (1H, dd, *J* = 12.0, 1.1 Hz, OC**H**$_2$CH), 3.51 (1H, dd, *J* = 12.1, 3.5 Hz,

OC**H**$_2$CH), 2.31–2.28 (1H, m, OCH$_2$C**H**), 1.32 (3H, s, C**H**$_3$), 1.02 (3H, s, C**H**$_3$); ^{13}C NMR (101 MHz, $CDCl_3$) δ 148.1 (C), 140.5 (C), 137.6 (C), 137.0 (C), 134.6 (C), 131.5 (C), 129.5 (CH), 129.3 (CH), 129.0 (CH), 128.8 (CH), 128.4 (CH), 127.6 (CH), 127.5 (2 x CH), 127.3 (CH), 121.6 (CH), 65.9 (CH_2), 65.7 (CH_2), 64.2 (C), 50.5 (CH), 26.2 (CH_3), 25.3 (CH_3), one signal could not be identified, most likely due to it being broad; HRMS (ESI) Exact mass calculated for $[C_{24}H_{23}SO_3N_3Na]^+$ $[M+Na]^+$: 456.1352, found: 456.1355.

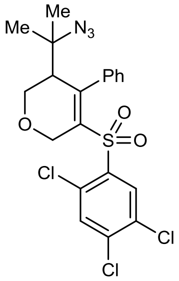

3-(2-Azidopropan-2-yl)-4-phenyl-5-[(2,4,5-trichlorophenyl)sulfonyl]-3,6-dihydro-2*H*-pyran (8e). The title compound was prepared according to General Procedure C from enyne **7a** (80.1 mg, 0.40 mmol) and 2,4,5-trichlorobenzenesulfonyl azide **2d** (344 mg, 1.20 mmol) for a reaction time of 36 h and purified by column chromatography (15% EtOAc/petrol) to give a colorless solid (177 mg, 91%). R_f = 0.65 (33% EtOAc/petrol); m.p. 134–138 °C (petrol); IR 2983, 2106, 2071, 1438, 1327, 1143, 1131, 1060, 871, 698, 558, 539 cm^{-1}; ^{1}H NMR (400 MHz, $CDCl_3$) δ 7.44 (1H, dt, *J* = 7.9, 1.5 Hz, Ar**H**), 7.38 (1H, s, Ar**H**), 7.31 (1H, td, *J* = 7.6, 1.4 Hz, Ar**H**), 7.21 (1H, tt, *J* = 7.5, 1.3 Hz, Ar**H**), 7.14 (1H, s, Ar**H**), 6.88–6.78 (1H, m, Ar**H**), 6.44 (1H, dt, *J* = 7.9, 1.5 Hz, Ar**H**), 4.80 (1H, dd, *J* = 17.4, 4.0 Hz, OC**H**$_2$C=C), 4.74 (1H, dd, *J* = 17.4, 2.4 Hz, OC**H**$_2$C=C), 4.39 (1H, dd, *J* = 12.1, 1.0 Hz, OC**H**$_2$CH), 3.62 (1H, dd, *J* = 12.1, 3.5 Hz, OC**H**$_2$CH), 2.40 (1H, app ddt, *J* = 3.6, 2.4, 1.1 Hz, OCH$_2$C**H**), 1.35 (3H, s, C**H**$_3$), 1.06 (3H, s, C**H**$_3$); ^{13}C NMR (101 MHz, $CDCl_3$) δ 147.9 (C), 139.1 (C), 137.8 (C), 137.3 (C), 137.1 (C), 132.4 (CH), 131.7 (C), 131.6 (CH), 131.1 (CH), 129.9 (C), 128.9 (CH), 127.6 (CH), 127.3 (CH), 125.6 (CH), 65.5 (CH_2), 64.9 (CH_2), 64.1 (C), 50.1 (CH), 26.1 (CH_3), 25.4 (CH_3); HRMS (ESI) Exact mass calculated for $[C_{20}H_{18}SCl_3O_3N_3Na]^+$ $[M+Na]^+$: 508.0027, found: 508.0043.

Slow diffusion of petrol into a solution of **8e** in CH_2Cl_2 gave crystals that were suitable for X-ray diffraction:

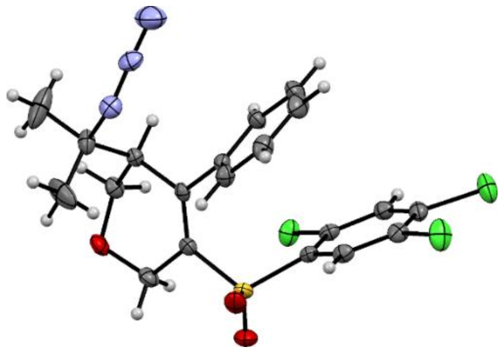

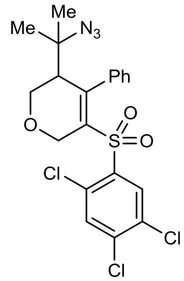

8e
CCDC 1537346

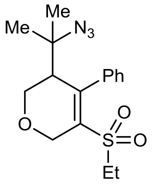

3-(2-Azidopropan-2-yl)-5-(ethylsulfonyl)-4-phenyl-3,6-dihydro-2*H*-pyran (8f). The title compound was prepared according to General Procedure C from enyne **7a** (80.1 mg, 0.40 mmol) and ethanesulfonyl azide **2g** (244 mg, 2.00 mmol) for a reaction time of 52 h and purified by column chromatography (15% EtOAc/petrol) to give a colorless solid (107 mg, 80%). R_f = 0.55 (33% EtOAc/petrol); m.p. 79–83 °C (petrol); IR 2959, 2925, 2107, 1303, 1271, 1131, 781, 726, 704, 508 cm^{-1}; ^{1}H NMR (400 MHz, $CDCl_3$) δ 7.63–7.10 (5H, m, Ar**H**), 4.70 (1H, dd, *J* = 17.7, 0.9 Hz, OC**H**$_2$C=C), 4.40 (1H, dd, *J* = 12.0, 1.2 Hz, OC**H**$_2$CH), 4.36 (1H, dd, *J* = 17.7, 2.7 Hz, OC**H**$_2$C=C), 3.57 (1H, dd, *J* = 12.0, 3.4 Hz, OC**H**$_2$CH), 2.59–2.51 (1H, m, OCH$_2$C**H**), 2.49–2.40 (1H, m, SC**H**$_2$), 2.25–2.11 (1H, m, SC**H**$_2$), 1.36 (3H, s, C(C**H**$_3$)$_2$), 1.14–1.11 (3H, t, *J* = 7.4 Hz, SCH$_2$C**H**$_3$), 1.09 (3H, s, C(C**H**$_3$)$_2$); ^{13}C NMR (101 MHz, $CDCl_3$) δ 147.1 (C), 138.1 (C), 137.9 (C), 129.1 (2 x CH), 128.3 (3 x CH), 65.78 (CH_2), 65.75 (CH_2), 64.3 (C), 50.1 (CH), 48.7 (CH_2), 26.2 (CH_3), 25.4 (CH_3), 7.1 (CH_3); HRMS (ESI) Exact mass calculated for $[C_{16}H_{21}SO_3N_3Na]^+$ $[M+Na]^+$: 358.1196, found: 358.1196.

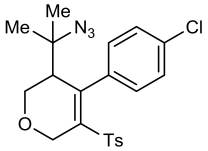

3-(2-Azidopropan-2-yl)-4-(4-chlorophenyl)-5-tosyl-3,6-dihydro-2*H*-pyran (8g). The title compound was prepared according to General Procedure C from enyne **7b** (93.9 mg, 0.40 mmol) and 4-methylbenzenesulfonyl azide **2a** (236.6 mg, 1.20 mmol) for a reaction time of 48 h and purified by column chromatography (15% EtOAc/petrol) to give a colorless solid (79.5 mg, 46%). R_f = 0.52 (33% EtOAc/petrol); m.p. 132–135 °C; IR 2978, 2920, 2874, 2105, 2066, 1594, 1486, 1288, 1242, 1141, 1090, 1016, 822, 805, 711, 669, 652, 571, 536 cm^{-1}; ^{1}H NMR (400 MHz, $CDCl_3$) δ 7.30 (2H, br s, Ar**H**), 7.19 (2H, d, *J* = 8.4 Hz, Ar**H**), 7.09 (2H, d, *J* = 8.1 Hz, Ar**H**), 7.04 (1H, br s, Ar**H**), 6.40 (1H, br s, Ar**H**), 4.80 (1H, dd, *J* = 17.6, 0.9 Hz, OC**H**$_2$C=C), 4.51 (1H, dd, *J* = 17.6, 2.7 Hz, OC**H**$_2$C=C), 4.30 (1H, dd, *J* = 12.1, 1.1 Hz, OC**H**$_2$CH), 3.49 (1H, dd, *J* = 12.1, 3.4 Hz, OC**H**$_2$CH), 2.37 (3H, s, ArC**H**$_3$), 2.32–2.24 (1H, m, OCH$_2$C**H**), 1.32 (3H, s, C(C**H**$_3$)$_2$), 1.08 (3H, s, C(C**H**$_3$)$_2$); ^{13}C NMR (101 MHz, $CDCl_3$) δ 146.2 (C), 144.1 (C), 141.4 (C), 137.7 (C), 136.5 (C), 134.4 (C), 132.7 (2 x CH), 129.2 (2 x CH), 127.8 (2 x CH), 127.2 (2 x CH), 65.7 (CH_2), 65.6 (CH_2), 64.2 (C), 50.4 (CH), 26.0 (CH_3), 25.5 (CH_3), 21.5 (CH_3); HRMS (ESI) Exact mass calculated for $[C_{21}H_{22}ClSO_3N_3Na]^+$ $[M+Na]^+$: 454.0963, found: 454.0964.

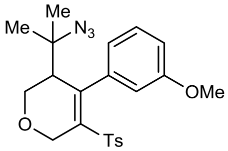

3-(2-Azidopropan-2-yl)-4-(3-methoxyphenyl)-5-tosyl-3,6-dihydro-2*H*-pyran (8h). The title compound was prepared according to General Procedure C from enyne **7c** (92.1 mg, 0.40 mmol) and 4-methylbenzenesulfonyl azide **2a** (236.6 mg, 1.20 mmol) for a reaction time of 15 h and purified by column chromatography (15% EtOAc/petrol) to give a colorless solid (128 mg, 75%). R_f = 0.58 (33% EtOAc/petrol); m.p. 116–120 °C; IR 2987, 2939, 2835, 2106, 1601, 1575, 1284, 1261, 1137, 1090, 1048, 783, 703, 647, 602, 567, 541 cm^{-1}; ^{1}H NMR (400 MHz, DMSO-d_6, 80 °C) δ 7.28–7.21 (2H, m, Ar**H**), 7.19–7.14 (2H, m, Ar**H**), 7.11 (1H, t, *J* = 7.9 Hz, Ar**H**), 6.81 (1H, ddd, *J* = 8.3, 2.6, 1.0 Hz, Ar**H**), 6.66 (1H, br s, Ar**H**), 6.46 (1H, br s, Ar**H**), 4.64 (1H, dd, *J* = 17.1, 1.0 Hz, OC**H**$_2$C=C), 4.51 (1H, dd, *J* = 17.1, 2.6 Hz, OC**H**$_2$C=C), 4.22 (1H, dd, *J* = 12.1, 1.3 Hz, OC**H**$_2$CH), 3.67 (3H, s, OC**H**$_3$), 3.59 (1H, dd, *J* = 12.1, 3.5 Hz, OC**H**$_2$CH), 2.58 (1H, app ddt, *J* = 3.7, 2.4, 1.1 Hz, OCH$_2$C**H**), 2.34 (3H, s, ArC**H**$_3$), 1.24 (3H, s, C(C**H**$_3$)$_2$), 1.05 (3H, s, C(C**H**$_3$)$_2$); ^{13}C NMR (101 MHz, DMSO-d_6 at 80 °C) δ 158.1 (C), 147.0 (C), 143.1 (C), 139.3 (C), 138.9 (C), 137.7 (C), 128.6 (2 x CH), 127.7 (CH), 126.5 (2 x CH), 121.4 (CH), 113.6 (CH), 64.8 (CH_2), 64.7 (CH_2), 63.8 (C), 54.6 (CH_3), 49.2 (CH), 25.1 (CH_3), 25.0 (CH_3), 20.4 (CH_3) one signal could not be identified, most likely due to it being broad; HRMS (ESI) Exact mass calculated for $[C_{22}H_{25}SO_4N_3Na]^+$ $[M+Na]^+$: 450.1458, found: 450.1453.

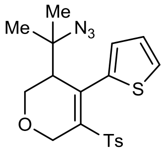

3-(2-Azidopropan-2-yl)-4-(thiophen-2-yl)-5-tosyl-3,6-dihydro-2*H*-pyran (8i). The title compound was prepared according to General Procedure C from enyne **7d** (82.5 mg, 0.40 mmol) and 4-methylbenzenesulfonyl azide **2a** (236.6 mg, 1.20 mmol) for a reaction time of 52 h and purified by preparative TLC to give a colorless solid (64.6 mg, 40%) (The yield is lower than expected because of difficulties in separating minor impurities.) R_f = 0.60 (33% EtOAc/petrol); m.p. 114–118 °C; IR 2969, 2924, 2873, 2110, 1297, 1289, 1142, 1129, 1090, 806, 709, 576, 536 cm^{-1}; ^{1}H NMR (400 MHz, $CDCl_3$) δ 7.28 (2H, td, *J* = 4.8, 1.2 Hz, Ar**H**), 7.26–7.21 (2H, m, Ar**H**), 7.07 (2H, d, *J* = 8.1 Hz, Ar**H**), 6.96 (1H, dd, *J* = 5.1, 3.6 Hz, Ar**H**), 4.85 (1H, dd, *J* = 18.0, 1.0 Hz, OC**H**$_2$C=C), 4.57 (1H, dd, *J* = 18.0, 2.8 Hz, OC**H**$_2$C=C), 4.36 (1H, dd, *J* = 12.1, 1.1 Hz, OC**H**$_2$CH), 3.47 (1H, dd, *J* = 12.1, 3.3 Hz, OC**H**$_2$CH), 2.35 (3H, s, ArC**H**$_3$), 2.28-2.25 (1H, m, OCH$_2$C**H**), 1.34 (3H, s, C(C**H**$_3$)$_2$), 1.05 (3H, s, C(C**H**$_3$)$_2$); ^{13}C NMR (101 MHz, $CDCl_3$) δ 143.8 (C), 142.8 (C), 140.5 (C), 138.9 (C), 137.4 (C), 131.6 (CH), 129.1 (2 x CH), 128.1 (CH), 127.3 (2 x CH), 126.8 (CH), 66.2 (CH_2), 65.5 (CH_2), 64.4 (C), 51.8 (CH), 25.8 (CH_3), 24.9 (CH_3), 21.6 (CH_3); HRMS (ESI) Exact mass calculated for $[C_{19}H_{21}S_2O_3N_3Na]^+$ $[M+Na]^+$: 426.0917, found: 426.0912.

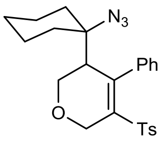

3-(1-Azidocyclohexyl)-4-phenyl-5-tosyl-3,6-dihydro-2*H*-pyran (8j). The title compound was prepared according to General Procedure C from enyne **7e** (96.1 mg, 0.40 mmol) and 4-methylbenzenesulfonyl azide **2a** (237 mg, 1.20 mmol) for a reaction time of 36 h and purified by column chromatography (15% EtOAc/petrol) to give a colorless solid (106 mg, 61%). R_f = 0.45 (33% EtOAc/petrol); m.p. 158–161 °C (petrol); IR 2937, 2857, 2098, 2076, 1289, 1206, 1143, 1088, 679, 565, 517 cm^{-1}; ^{1}H NMR (400 MHz, $CDCl_3$) δ 7.49 (1H, br s, Ar**H**), 7.32–7.20 (2H, m, Ar**H**), 7.19–7.04 (3H, m, Ar**H**), 7.04–6.95 (2H, m, Ar**H**), 6.48 (1H, br s, Ar**H**), 4.83 (1H, dd, *J* = 17.5, 0.9 Hz, OC**H**$_2$C=C), 4.57 (1H, dd, *J* = 17.5, 2.6 Hz, OC**H**$_2$C=C), 4.34 (1H, dd, *J* = 12.1, 1.1 Hz, OC**H**$_2$CH), 3.50 (1H, dd, *J* = 12.1, 3.2 Hz, OC**H**$_2$CH), 2.42–2.37 (1H, m, OCH$_2$C**H**), 2.34 (3H, s, ArC**H**$_3$), 1.68–1.55 (2H, m, (C**H**$_2$)$_5$), 1.50–1.34 (6H, m, (C**H**$_2$)$_5$), 1.35–1.27 (1H, m, (C**H**$_2$)$_5$), 1.11–1.01 (1H, m, (C**H**$_2$)$_5$); ^{13}C NMR (101 MHz, $CDCl_3$) δ 148.0 (C), 143.7 (C), 140.2 (C), 138.2 (C), 138.0 (C), 129.0 (2 x CH), 128.1 (CH), 127.5 (2 x CH), 127.2 (2 x CH), 67.4 (C), 65.8 (CH_2), 65.4 (CH_2), 51.0 (CH), 33.7 (CH_2), 33.6 (CH_2), 24.9 (CH_2), 22.2 (CH_2), 21.9 (CH_2), 21.5 (CH_3), one signal could not be identified, most likely due to it being broad; HRMS (ESI) Exact mass calculated for $[C_{24}H_{27}N_3SO_3Na]^+$ $[M+Na]^+$: 460.1665, found: 460.1665.

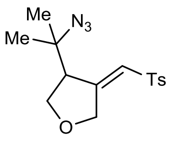

(*Z*)-3-(2-Azidopropan-2-yl)-4-(tosylmethylene)tetrahydrofuran (10a). The title compound was prepared according to General Procedure C from enyne **7f** (49.7 mg, 0.40 mmol) and 4-methylbenzenesulfonyl azide **2a** (237 mg, 1.20 mmol) for a reaction time of 24 h and purified by column chromatography (15% EtOAc/petrol) to give a colorless gum (97.7 mg, 76%). R_f = 0.45 (33% EtOAc/petrol); IR 2922, 2103, 1302, 1143, 1084, 808, 653, 572, 537 cm^{-1}; ^{1}H NMR (400 MHz, $CDCl_3$) δ 7.83–7.74 (2H, m, Ar**H**), 7.35 (2H, dt, *J* = 8.7, 1.4 Hz, Ar**H**), 6.54 (1H, td, *J* = 2.6, 1.7 Hz, SC**H**=C), 4.82 (2H, dt, *J* = 2.7, 1.3 Hz, OC**H**$_2$C=CH), 3.92–3.77 (2H, m, OC**H**$_2$CH), 2.76 (1H, ddq, *J* = 6.7, 4.1, 1.3 Hz, OCH$_2$C**H**), 2.44 (3H, s, ArC**H**$_3$), 1.29 (3H, s, C(C**H**$_3$)$_2$), 1.22 (3H, s, C(C**H**$_3$)$_2$); ^{13}C NMR (101 MHz, $CDCl_3$) δ 156.2 (C), 144.7 (C), 138.1 (C), 130.0 (2 x CH), 127.2 (2 x CH), 124.6 (CH), 70.5 (CH_2), 68.9 (CH_2), 62.8 (C), 54.2 (C), 24.9 (CH_3), 22.2 (CH_3), 21.6 (CH_3); HRMS (ESI) Exact mass calculated for $[C_{15}H_{19}SO_3N_3Na]^+$ $[M+Na]^+$: 344.1039, found: 344.1037.

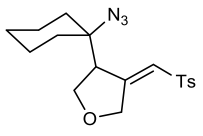

(*Z*)-3-(1-Azidocyclohexyl)-4-(tosylmethylene)tetrahydrofuran (10b). *On a 0.40 mmol scale*: The title compound was prepared according to General Procedure C from enyne **7g** (65.7 mg, 0.40 mmol) and 4-methylbenzenesulfonyl azide **2a** (237 mg, 1.20 mmol) for a reaction time of 24 h and purified by column chromatography (15% EtOAc/petrol) to give a colorless solid (127 mg, 88%). R_f = 0.40 (33% EtOAc/petrol); m.p. 103–106 °C (petrol); IR

2970, 2325, 1739, 1366, 1205, 911, 801, 646 cm^{-1}; ^{1}H NMR (400 MHz, $CDCl_3$) δ 7.80–7.72 (2H, m, Ar**H**), 7.38–7.29 (2H, m, Ar**H**), 6.45 (1H, td, *J* = 2.6, 1.6 Hz, SC**H**=C), 4.85 (1H, dd, *J* = 17.2, 2.6 Hz, OC**H**$_2$C=CH), 4.79 (1H, ddd, *J* = 17.2, 2.6, 1.6 Hz, OC**H**$_2$C=CH), 4.00 (1H, dd, *J* = 9.7, 3.6 Hz, OC**H**$_2$CH), 3.81 (1H, dd, *J* = 9.6, 6.7 Hz, OC**H**$_2$CH), 2.84 (1H, ddt, *J* = 6.8, 3.5, 1.6 Hz, CH_2C**H**), 2.44 (3H, s, ArC**H**$_3$), 1.74–1.64 (2H, m, (C**H**$_2$)$_5$), 1.62–1.49 (4H, m, (C**H**$_2$)$_5$), 1.49–1.43 (2H, m, (C**H**$_2$)$_5$), 1.30–1.22 (1H, m, (C**H**$_2$)$_5$), 1.16–1.03 (1H, m, (C**H**$_2$)$_5$); ^{13}C NMR (101 MHz, $CDCl_3$) δ 156.2 (C), 144.7 (C), 138.0 (C), 130.0 (2 x CH), 127.3 (2 x CH), 124.5 (CH), 70.4 (CH_2), 68.7 (CH_2), 65.8 (C), 54.9 (CH), 32.8 (CH_2), 30.7 (CH_2), 24.9 (CH_2), 21.7 (CH_2), 21.6 (CH_2 and CH_3); HRMS (ESI) Exact mass calculated for $[C_{18}H_{23}SO_3N_3Na]^+$ $[M+Na]^+$: 384.1352, found: 384.1356.

Slow diffusion of petrol into a solution of **10b** in CH_2Cl_2 gave crystals that were suitable for X-ray diffraction:

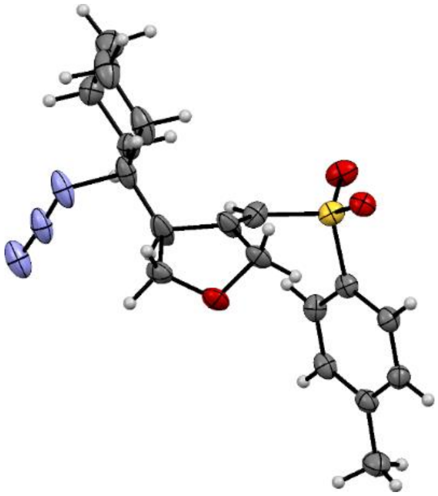

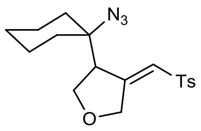

10b
CCDC 1537347

Note: In the tetrahydrofuran ring, the oxygen atom along with the two carbon atoms adjacent to that oxygen atom, are disordered over two positions. The occupancies of the two components were refined competitively, converging to a ratio of 0.50:0.50. This disorder is not shown above, for clarity.

On a 3.0 mmol scale:

(*Z*)-3-(Cyclohex-1-en-1-yl)-4-(tosylmethylene)tetrahydrofuran (11)

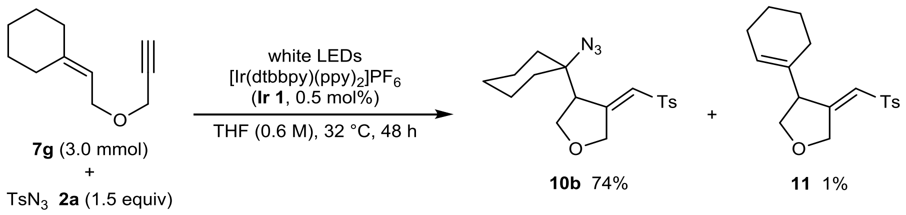

An oven-dried microwave tube equipped with a stirrer bar was charged with $[Ir(dtbbpy)(ppy)_2]PF_6$ (13.7 mg, 0.015 mmol) and enyne **7g** (493 mg, 3.00 mmol). The tube was sealed with a septum-lined

cap and purged with nitrogen for 1 h. A solution of sulfonyl azide **2a** (887 mg, 4.50 mmol) in THF (5.0 mL) was then added. The mixture was stirred at 32 °C under irradiation by white LEDs for 40 h. The crude mixture was directly purified by flash column chromatography on silica gel (25% Et_2O/petrol) to give **10b** as a colorless solid (802 mg, 74%). The fractions containing minor impurities were combined and concentrated *in vacuo* to leave material that was purified further by preparative TLC (20% Et_2O/petrol) to give *diene* **11** (10 mg, 1%,) as a colorless oil which was not completely pure. R_f = 0.30 (25% Et_2O/petrol); IR 2924, 2854, 1738, 1646, 1597, 1456, 1302, 1147, 1086, 1072, 933, 814, 655 cm^{-1}; ^{1}H NMR (400 MHz, $CDCl_3$) δ 7.76 (2H, d, *J* = 8.2 Hz, Ar**H**), 7.34 (2H, d, *J* = 8.2 Hz, Ar**H**), 6.08 (1H, app q, *J* = 2.5 Hz, SC**H**=C), 5.58 (1H, app tt, *J* = 3.7, 1.7 Hz, C=C**H**CH_2), 4.97 (1H, dd, *J* = 17.2, 2.4 Hz, OC**H**$_2$C=CH), 4.75 (1H, dt, *J* = 17.2, 2.4 Hz, OC**H**$_2$C=CH), 4.03–3.98 (1H, m, OC**H**$_2$CH), 3.65 (1H, t, *J* = 8.6 Hz, OC**H**$_2$CH), 3.38 (1H, tt, *J* = 8.1, 2.3 Hz, OCH_2C**H**), 2.45 (3H, s, ArC**H**$_3$), 2.03–1.99 (2H, m, C**H**$_2CH_2CH_2$C**H**$_2$), 1.86–1.71 (2H, m, C**H**$_2CH_2CH_2$C**H**$_2$), 1.57–1.45 (4H, m, CH_2C**H**$_2$C**H**$_2CH_2$); ^{13}C NMR (101 MHz, $CDCl_3$) δ 161.2 (C), 144.4 (C), 138.4 (C), 133.7 (C), 129.9 (2 x CH), 127.8 (CH), 127.1 (2 x CH), 121.5 (CH), 70.6 (CH_2), 70.4 (CH_2), 54.8 (CH), 25.3 (CH_2), 24.8 (CH_2), 22.7 (CH_2), 22.1 (CH_2), 21.6 (CH_3); HRMS (ESI) Exact mass calculated for $[C_{18}H_{22}NaO_3S]^+$ $[M+Na]^+$: 341.1182, found: 341.1180.

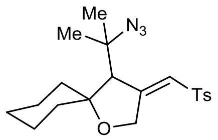

(*Z*)-4-(2-Azidopropan-2-yl)-3-(tosylmethylene)-1-oxaspiro[4.5]decane (10c). The title compound was prepared according to General Procedure C from enyne **7h** (76.9 mg, 0.40 mmol) and sulfonyl azide **2a** (237 mg, 1.20 mmol) for a reaction time of 36 h and purified by column chromatography (15% EtOAc/petrol) to give a colorless solid (103 mg, 66%). R_f = 0.42 (33% EtOAc/petrol); m.p. 142–146 °C (petrol); IR 2930, 2856, 2098, 1312, 1145, 1057, 813, 780, 653, 537 cm^{-1}; ^{1}H NMR (400 MHz, $CDCl_3$) δ 7.82–7.71 (2H, m, Ar**H**), 7.35 (2H, d, *J* = 8.0 Hz, Ar**H**), 6.42 (1H, td, *J* = 2.6, 1.3 Hz, SC**H**=C), 4.83 (1H, dd, *J* = 17.8, 2.5 Hz, OC**H**$_2$C=CH), 4.71 (1H, ddd, *J* = 17.7, 2.7, 1.7 Hz, OC**H**$_2$C=CH), 2.44 (3H, s, ArC**H**$_3$), 2.32 (1H, d, *J* = 1.5 Hz, C**H**CN_3), 1.68–1.56 (5H, m, (C**H**$_2$)$_5$), 1.51 (2H, ddt, *J* = 13.2, 6.6, 3.4 Hz, (C**H**$_2$)$_5$), 1.42 (1H, dt, *J* = 12.7, 3.7 Hz, (C**H**$_2$)$_5$), 1.33 (3H, s, C(C**H**$_3$)$_2$), 1.28 (3H, s, C(C**H**$_3$)$_2$), 1.24–1.15 (2H, m, (C**H**$_2$)$_5$); ^{13}C NMR (101 MHz, $CDCl_3$) δ 159.1 (C), 144.6 (C), 138.2 (C), 130.0 (2 x CH), 127.1 (2 x CH), 125.7 (CH), 83.9 (C), 66.4 (CH_2), 62.7 (C), 61.8 (CH), 36.3 (CH_2), 32.5 (CH_2), 25.5 (CH_3), 25.4 (CH_2), 24.2 (CH_3), 22.5 (CH_2), 22.1 (CH_2), 21.6 (CH_3); HRMS (ESI) Exact mass calculated for $[C_{20}H_{27}SO_3N_3Na]^+$ $[M+Na]^+$: 412.1665, found: 412.1663.

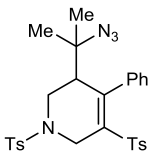

3-(2-Azidopropan-2-yl)-4-phenyl-1,5-ditosyl-1,2,3,6-tetrahydropyridine (8k). *On a 0.40 mmol scale*: The title compound was prepared according to General Procedure C from enyne **7i** (141.4 mg, 0.40 mmol) and 4-methylbenzenesulfonyl azide **2a** (237 mg, 1.20 mmol) for a reaction time of 36 h and purified by column chromatography (15% EtOAc/petrol) to give a colorless solid (99 mg, 64%). R_f = 0.55 (33% EtOAc/petrol); m .p. 165–168 °C (petrol); IR 2970, 2922, 2104, 1739, 1353, 1163, 1140, 807, 694, 562, 543 cm^{-1}; ^{1}H NMR (400 MHz, $CDCl_3$) δ 7.79–7.70 (2H, m, Ar**H**), 7.39 (2H, d, *J* = 8.0 Hz, Ar**H**), 7.29 (1H, br s, Ar**H**), 7.24 (2H, d, *J* = 12.2 Hz, Ar**H**), 7.13–7.06 (2H, m, Ar**H**), 7.02 (1H, br s, Ar**H**), 7.00 (2H, d, *J* = 8.1 Hz, Ar**H**), 6.37 (1H, s, br Ar**H**), 4.62 (1H, dt, *J* = 17.4, 1.1 Hz, NC**H**$_2$C=C), 4.12 (1H, dt, *J* = 12.2, 1.3 Hz, NC**H**$_2$CH), 3.66 (1H, dd, *J* = 17.4, 2.3 Hz, NC**H**$_2$C=C), 2.55–2.51 (1H, m, NC**H**$_2$CH), 2.51–2.43 (1H, m, NCH_2C**H**CN_3), 2.48 (3H, s, ArC**H**$_3$), 2.34 (3H, s, ArC**H**$_3$), 1.40 (3H, s, C(C**H**$_3$)$_2$), 1.18 (3H, s, C(C**H**$_3$)$_2$); ^{13}C NMR (101 MHz, $CDCl_3$) δ 148.3 (C), 144.4 (C), 143.9 (C), 138.6 (C), 137.7 (2 x C), 132.0 (C), 130.0 (2 x CH), 129.1 (2 x CH), 128.3 (CH), 127.9 (2 x CH), 127.5 (2 x CH), 127.3 (2 x CH), 64.2 (C), 51.8 (CH), 45.9 (CH_2), 44.8 (CH_2), 25.6 (CH_3), 25.5 (CH_3), 21.6 (CH_3), 21.5 (CH_3), one signal could not be identified, most likely due to it being broad; HRMS (ESI) Exact mass calculated for $[C_{28}H_{30}N_4S_2O_4Na]^+$ $[M+Na]^+$: 573.1601, found: 573.1602.

On a 2.00 mmol scale:

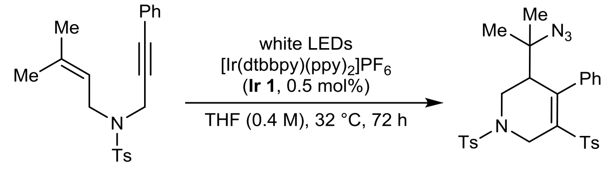

An oven-dried microwave tube equipped with a stirrer bar was charged with $[Ir(dtbbpy)(ppy)_2]PF_6$ (9.1 mg, 0.01 mmol) and enyne **7i** (707 mg, 2.00 mmol). The tube was sealed with a septum-lined cap and purged with nitrogen for 1 h. A solution of sulfonyl azide **2a** (591 mg, 3.00 mmol) in THF (5.0 mL) was then added. The mixture was stirred at 32 °C under irradation by white LEDs for 72 h. The crude mixture was directly purified by flash column chromatography on silica gel (25% EtOAc/petrol) to give **8k** as a colorless solid (510 mg, 46%).

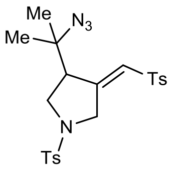

(*Z*)-3-(2-Azidopropan-2-yl)-1-tosyl-4-(tosylmethylene)pyrrolidine (10d). The title compound was prepared according to General Procedure C from enyne **7j** (111 mg, 0.40 mmol) and 4-methylbenzenesulfonyl azide **2a** (237 mg, 1.20 mmol) for a reaction time of 24 h and purified by column chromatography (15% EtOAc/petrol) to give a colorless gum (177 mg, 93%). R_f = 0.45 (33% EtOAc/petrol); IR 2973, 2104, 1343, 1290, 1144, 1086, 812, 662, 597, 577, 546 cm^{-1}; ^{1}H NMR (400 MHz, $CDCl_3$) δ 7.75–7.65 (4H, m, Ar**H**), 7.36–7.29 (4H, m, Ar**H**), 6.45 (1H, td, *J* = 2.6, 1.6 Hz, SC**H**=C), 4.37 (1H, dd, *J* = 17.8, 2.5 Hz, NC**H**$_2$C=C), 4.26 (1H, ddd, *J* = 17.8, 2.7, 1.8 Hz, NC**H**$_2$C=C), 3.31 (1H, dd, *J* = 10.5, 4.1 Hz, NC**H**$_2$CH), 3.17 (1H, dd, *J* = 10.5, 7.7 Hz, NC**H**$_2$CH), 2.66 (1H, ddt, *J* = 7.7, 4.0, 1.7 Hz, NCH_2C**H**), 2.43 (6H, s, 2 x ArC**H**$_3$), 1.25 (3H, s, C(C**H**$_3$)$_2$), 1.15 (3H, s, C(C**H**$_3$)$_2$); ^{13}C NMR (101 MHz, $CDCl_3$) δ 151.5 (C), 144.9 (C), 144.3 (C), 137.6 (C), 131.5 (C), 130.0 (2 x CH), 129.9 (2 x CH), 127.8 (2 x CH), 127.2 (2 x CH), 126.8 (CH), 62.9 (C), 52.9 (CH), 50.9 (CH_2), 48.2 (CH_2), 24.9 (CH_3), 22.1 (CH_3), 21.6 (CH_3), 21.5 (CH_3); HRMS (ESI) Exact mass calculated for $[C_{22}H_{26}S_2O_4N_4Na]^+$ $[M+Na]^+$: 497.1288, found: 497.1298.

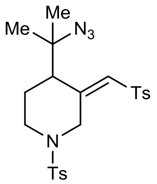

(*Z*)-4-(2-Azidopropan-2-yl)-1-tosyl-3-(tosylmethylene)piperidine (10e). The title compound was prepared according to General Procedure C from enyne **7k** (117 mg, 0.40 mmol) and 4-methylbenzenesulfonyl azide **2a** (237 mg, 1.20 mmol) for a reaction time of 36 h and purified by column chromatography (15% EtOAc/petrol) to give a colorless gum (106 mg, 54%). R_f = 0.55 (33% EtOAc/petrol); IR 2970, 2926, 2100, 1342, 1303, 1144, 1085, 813, 659, 545 cm^{-1}; ^{1}H NMR (400 MHz, $CDCl_3$) δ 7.94–7.88 (2H, m, Ar**H**), 7.75–7.69 (2H, m, Ar**H**), 7.40-7.35 (4H, m, Ar**H**), 6.34 (1H, d, *J* = 0.9 Hz, SC**H**=C), 5.07 (1H, d, *J* = 14.1 Hz, NC**H**$_2$C=C), 3.90–3.81 (1H, m, NC**H**$_2$C=C), 3.32–3.18 (2H, m, NC**H**$_2$CH$_2$), 2.46 (6H, s, 2 x ArC**H**$_3$), 2.10–2.03 (1H, m, CH_2C**H**), 2.00–1.81 (2H, m, NCH_2C**H**$_2$), 1.32 (3H, s, C(C**H**$_3$)$_2$), 1.27 (3H, s, C(C**H**$_3$)$_2$); ^{13}C NMR (101 MHz, $CDCl_3$) δ 148.0 (C), 144.7 (C), 143.8 (C), 138.0 (C), 133.2 (C), 131.6 (CH), 130.1 (2 x CH), 129.8 (2 x CH), 127.7 (2 x CH), 127.6 (2 x CH), 62.6 (C), 49.5 (CH), 43.7 (CH_2), 42.7 (CH_2), 26.2 (CH_3), 26.0 (CH_2), 25.1 (CH_3), 21.7 (CH_3), 21.6 (CH_3); HRMS (ESI) Exact mass calculated for $[C_{23}H_{28}S_2O_4N_4Na]^+$ $[M+Na]^+$: 511.1444, found: 511.1444.

Sulfonylative Cyclization in THF-D8

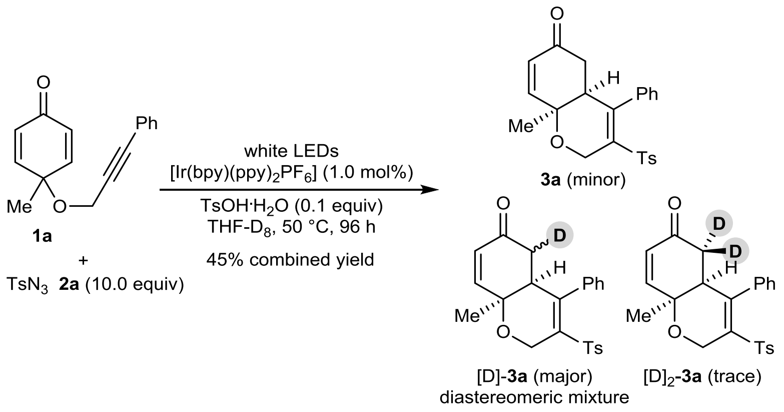

An oven-dried microwave tube equipped with a stirrer bar was charged with $[Ir(bpy)(ppy)_2]PF_6$ (**Ir 2**, 1.6 mg, 0.002 mmol), *p*-toluenesulfonic acid monohydrate (3.8 mg, 0.02 mmol) and the cyclohexadienone-containing 1,6-enyne **1a** (47.6 mg, 0.20 mmol). The tube was sealed with a septum-lined cap and purged with nitrogen for 1 h. A solution of sulfonyl azide **2a** (523 mg, 2.00 mmol) in THF-D_8 (2.5 mL) was then added. The mixture was stirred at 50 °C under irradiation by white LEDs for 96 h. After 1,6-enyne **1a** was completely consumed (monitored by TLC), the crude mixture was directly purified by flash column chromatography on silica gel (25% EtOAc/petrol) to give an inseparable mixture of the products **3a**, [D]-**3a** (likely a mixture of diastereomers), and $[D]_2$-**3a** as a colorless powder (35.6 mg, 45%). The relative quantities of **3a**, [D]-**3a**, and $[D]_2$-**3a** could not be determined accurately, but the ratio of **3a** and [D]-**3a** was estimated by NMR analysis to be *ca*. 4:1 (however, it is recognized that the error in this estimation is large). Only a small trace of $[D]_2$-**3a** (*ca.* <5%) was detected by HRMS analysis.

R_f = 0.40 (33% EtOAc/petrol); IR 2928 (petrol), 2358, 2174, 2041, 1977, 1945, 1740, 1686 (C=O), 1372, 1317, 1150, 1085, 702, 674, 553 cm^{-1}; ^{1}H NMR (400 MHz, $CDCl_3$) δ 7.35–7.23 (5H, m, Ar**H**), 7.12 (2H, d, *J* = 8.3 Hz, Ar**H**), 6.86 (2H, br s, Ar**H**), 6.75 (1H, d, *J* = 10.2 Hz, C**H**=CHC=O), 6.08 (1H, d, *J* = 10.2 Hz, C**H**C=O), 4.82–4.66 (2H, m, OC**H**$_2$), 2.74–2.64 (1H, m, C**H**CHD), 2.47–2.32 (integrates to ~1.2H, m, CH_2C=O of **3a** and C**H**D of [D]-**3a**), 2.40 (3H, s, ArC**H**$_3$), 1.53 (3H, s, OCC**H**$_3$); ^{13}C NMR (101 MHz, $CDCl_3$) Mixture of isotopologues and diastereomers (signals not fully assigned) δ 196.8 (C), 196.7 (C), 150.2 (CH), 150.11 (CH), 150.08 (CH), 148.1 (C), 144.1 (C), 137.8 (C), 137.03 (C), 137.00 (C), 136.96 (C), 135.32 (C), 135.29 (C), 135.27 (C), 130.45 (CH), 130.40 (CH), 130.37 (CH), 129.3 (CH), 128.3 (CH), 127.9 (CH), 127.4 (CH), 70.6 (C), 70.5 (C), 70.4 (C), 61.3 (CH_2), 46.1 (CH), 38.1 (CH_2), 37.8 (CHD, *J* = 21.1 Hz), 23.69 (CH_3), 23.63 (CH_3), 23.60 (CH_3), 21.6 (CH_3); HRMS (ESI) Exact mass calculated for $[C_{23}H_{22}SO_4Na]^+$ $[M+Na]^+$: 417.1131, found: 417.1121. Exact mass calculated for $[C_{23}H_{21}DSO_4Na]^+$ $[M+Na]^+$: 418.1194, found: 418.1186.

In addition to the standard HRMS analysis reported above, an additional HRMS analysis was performed using a Thermo Scientific Orbitrap Q-Exactive (Thermo Scientific, Bremen, Germany) mass spectrometer, operated at a resolution of *ca*. 100,000. The theoretical masses of relevant species are shown below:

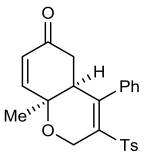

3a

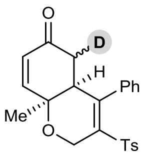

[D]-**3a**

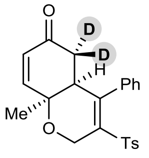

$[D]_2$-**3a**

Theoretical masses:

Parent ion $[C_{23}H_{22}NaO_4S]^+$ $[M+Na]^+$ = 417.1131
^{13}C peak $[^{12}C_{22}{}^{13}CH_{22}NaO_4S]^+$ $[M+Na]^+$ = 418.1165
^{34}S peak $[C_{23}H_{22}NaO_4{}^{34}S]^+$ $[M+Na]^+$ = 419.1089

Parent ion $[C_{23}H_{21}DNaO_4S]^+$ $[M+Na]^+$ = 418.1194
^{13}C peak $[^{12}C_{22}{}^{13}CH_{21}DNaO_4S]^+$ $[M+Na]^+$ = 419.1227
^{34}S peak $[C_{23}H_{21}DNaO_4{}^{34}S]^+$ $[M+Na]^+$ = 420.1152

Parent ion $[C_{23}H_{20}D_2NaO_4S]^+$ $[M+Na]^+$ = 419.1257
^{13}C peak $[^{12}C_{22}{}^{13}CH_{20}D_2NaO_4S]^+$ $[M+Na]^+$ = 420.1290
^{34}S peak $[C_{23}H_{20}D_2NaO_4{}^{34}S]^+$ $[M+Na]^+$ = 421.1215

The figure below illustrates the isotopic pattern acquired by the Orbitrap spectrometer around the region corresponding to the $[M+Na]^+$ ions of the target compounds.

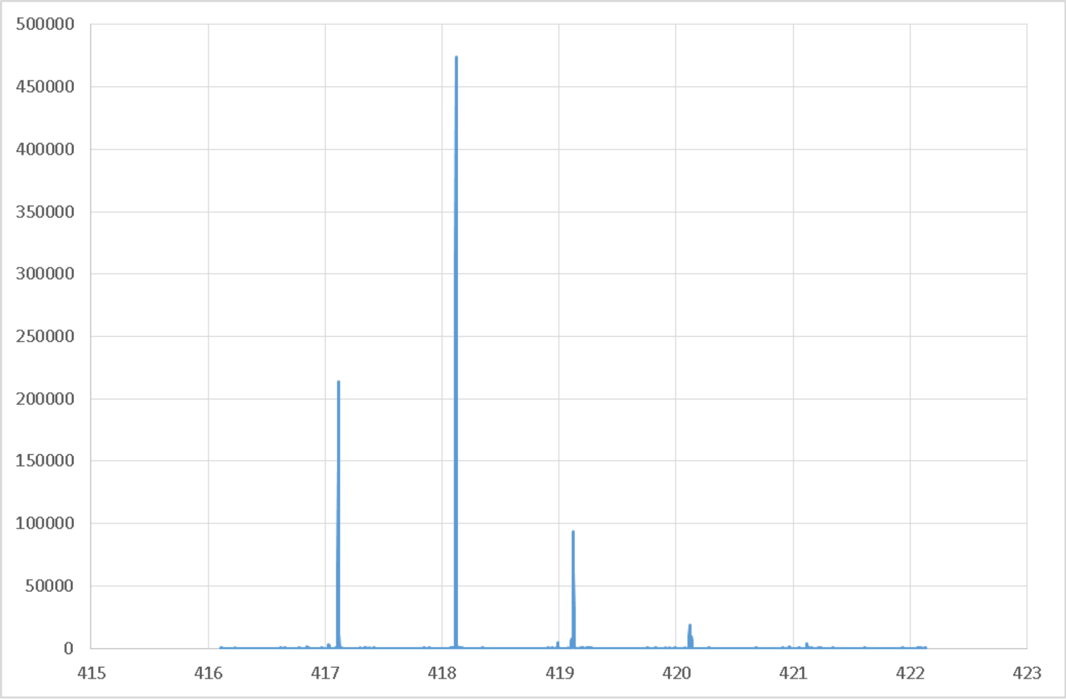

Expansions in the regions 417, 418, 419, and 420, along with likely species responsible for the indicated peaks, are provided in the following figures.

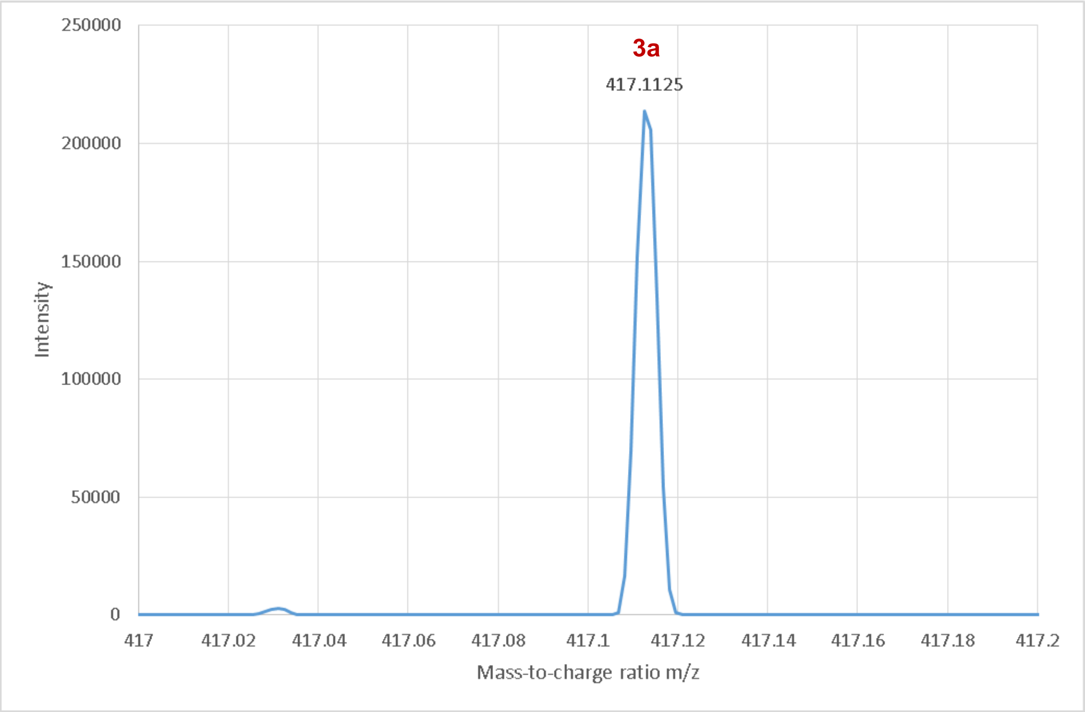

The above expansion clearly indicates the non-deuterated compound **3a** is present: $[C_{23}H_{22}NaO_4S]^+$, *m*/*z* 417.1125, theoretical 418.1131.

[D]-**3a** and **3a** (^{13}C peak) - peaks not resolved

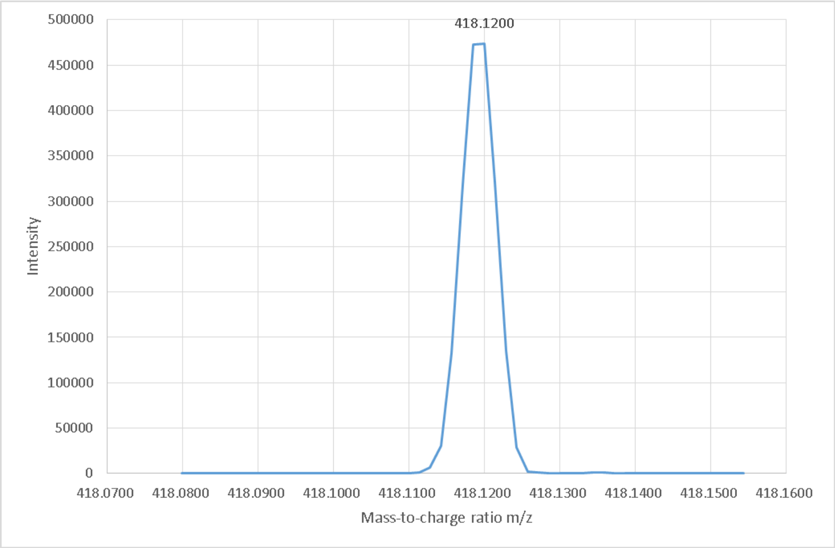

Expansion of the mass scale in the region of *m/z* 418 (above) indicates a single peak at *m*/*z* 418.1200 with no resolution of the peaks attributable to the monodeuterated compound [D]-**3a**: $[C_{23}H_{21}DNaO_4S]^+$, theoretical 418.1194, and the ^{13}C isotope peak of **3a**: $[C_{22}{}^{13}CH_{22}NaO_4S]^+$, theoretical 418.1165.

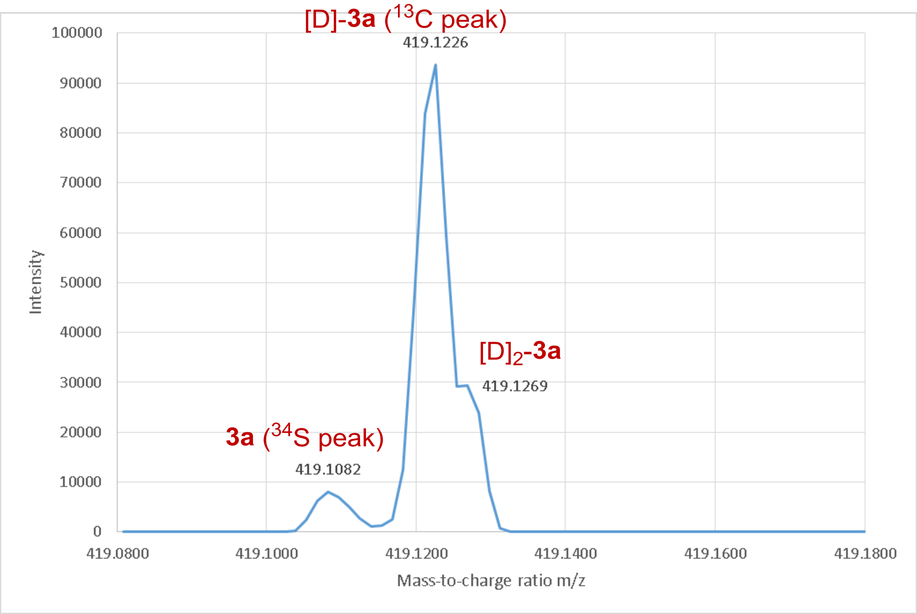

Expansion of the mass scale in the region of *m/z* 419 (above) indicates the partial resolution of the dideuterated compound [D]$_2$-**3a**: [$C_{23}H_{20}D_2NaO_4S$]$^+$, *m/z* 419.1269; theoretical 419.1257, from the more intense ^{13}C isotope signal resulting from the monodeuterated compound [D]-**3a**: [$C_{22}{}^{13}CH_{21}DNaO_4S$]$^+$, *m/z* 419.1226; theoretical 419.1227. In addition, in this view, the ion recorded at *m/z* 419.1082 represents the ^{34}S isotopologue from the non-deuterated compound [$C_{23}H_{23}NaO_4{}^{34}S$]$^+$, theoretical 419.1089.

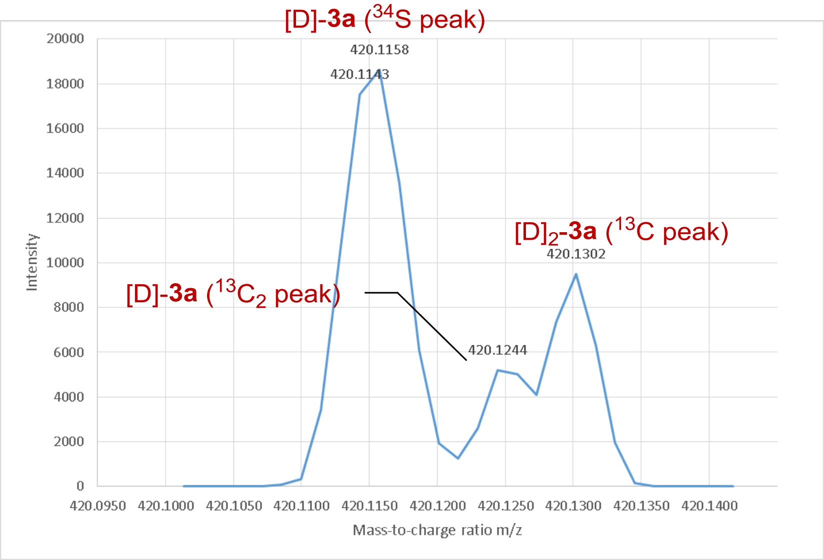

The isotope cluster in the region of *m/z* 420 (above) further validates the presence of the di-deuterated compound [D]$_2$-**3a**, where the ^{13}C isotopologue is clearly detected: [$C_{22}{}^{13}CH_{20}D_2NaO_4S$]$^+$, *m/z* 420.1302; theoretical 420.1290. Other ions plotted in this view are: [$C_{21}{}^{13}C_2H_{21}DNaO_4S$]$^+$, *m/z* 420.1244; theoretical 420.1261 and [$C_{23}H_{21}DNaO_4{}^{34}S$]$^+$, *m/z* 420.1158; theoretical 420.1152.

Aziridination of 1,6-Enyne 7a

2,2-Dimethyl-3-{[(3-phenylprop-2-yn-1-yl)oxy]methyl}-1-tosylaziridine (24)

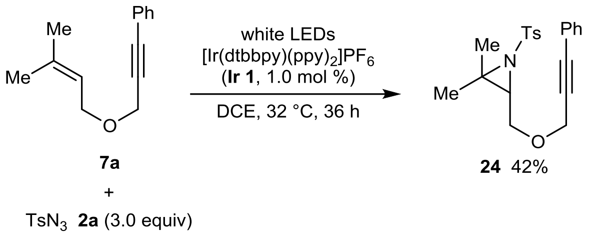

An oven-dried microwave tube equipped with a stirrer bar was charged with $[Ir(dtbbpy)(ppy)_2]PF_6$ (3.7 mg, 0.004 mmol) and enyne **7a** (80.1 mg, 0.40 mmol). The tube was sealed with a septum-lined cap and purged with nitrogen for 1 h. A solution of the sulfonyl azide **2** (1.2 mmol) in DCE (2.0 mL) was then added. The tube was then immersed sideways into a sand bath heated to 32 °C, and irradiated by white LEDs (see General Information for details of the light source) for 36 h (see photograph on page S11). The crude mixture was directly purified by flash column chromatography on silica gel (15% EtOAc/petrol) to give **24** as a yellow oil (62.1 mg, 42%). R_f = 0.30 (25% Et_2O/petrol); IR 2926, 2852, 2125 (C≡C), 1597, 1490, 1319, 1304, 1155, 1085, 931, 814, 757, 708, 670, 582, 528 cm^{-1}; ^{1}H NMR (400 MHz, $CDCl_3$) δ 7.87–7.81 (2H, m, Ar**H**), 7.43–7.37 (2H, m, Ar**H**), 7.34–7.29 (3H, m, Ar**H**), 7.29–7.26 (2H, m, Ar**H**), 4.33–4.18 (2H, m, OC**H**$_2$C≡C), 3.64 (1H, dd, *J* = 10.8, 6.2 Hz, OC**H**$_2$CH), 3.51 (1H, dd, *J* = 10.8, 6.2 Hz, OC**H**$_2$CH), 3.18 (1H, t, *J* = 6.2 Hz, OCH_2C**H**), 2.39 (3H, s, ArC**H**$_3$), 1.76 (3H, s, NCC**H**$_3$), 1.35 (3H, s, NCC**H**$_3$); ^{13}C NMR (101 MHz, $CDCl_3$) δ 143.7 (C), 138.2 (C), 131.7 (2 x CH), 129.4 (2 x CH), 128.6 (CH), 128.3 (2 x CH), 127.3 (2 x CH), 122.4 (C), 86.6 (C), 84.5 (C), 67.2 (CH_2), 58.9 (CH_2), 51.2 (C), 50.1 (CH), 21.6 (CH_3), 21.3 (CH_3), 21.2 (CH_3); HRMS (ESI) Exact mass calculated for $[C_{21}H_{23}SNO_3Na]^+$ $[M+Na]^+$: 392.1291, found: 392.1292.

Addition of Tetrahydrofuran-2-yl Radical to Electron-Deficient Alkenes

Phenyl 3-(tetrahydrofuran-2-yl)propanoate (27)

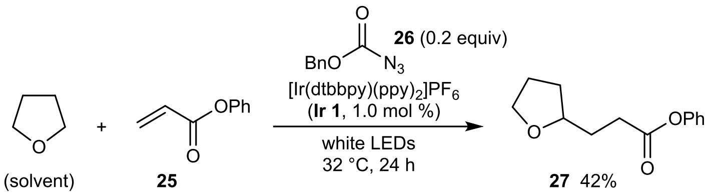

An oven-dried microwave tube equipped with a stirrer bar was charged with $[Ir(dtbbpy)(ppy)_2]PF_6$ (3.7 mg, 0.004 mmol) and phenyl acrylate **25** (59.3 mg, 0.40 mmol). The tube was sealed with a septum-lined cap and purged with nitrogen for 1 h. A solution of benzyl azidoformate **26**[21] (14.2 mg, 0.08 mmol) in THF (2.0 mL) was then added. The tube was immersed sideways into a sand bath heated to 32 °C, and irradiated by white LEDs (see General Information for details of the light source) with stirring for 24 h (see photograph on page S11). The crude mixture was directly purified by flash column chromatography on silica gel (10% EtOAc/petrol) to give **27** as a colorless oil (37.0 mg, 42%). R_f = 0.30 (10% Et_2O/petrol); IR 2980, 2971, 2926, 1754 (O=C), 1376, 1200, 1073, 957, 512 cm^{-1}; ^1H NMR (400 MHz, $CDCl_3$) δ 7.41–7.32 (2H, m, Ar**H**), 7.22 (1H, ddt, *J* = 7.8, 6.9, 1.1 Hz, Ar**H**), 7.11–7.03 (2H, m, Ar**H**), 3.99–3.81 (2H, m, OC**H**$_2$), 3.75 (1H, td, *J* = 7.9, 6.3 Hz, OC**H**), 2.77–2.58 (2H, m, C**H**$_2$C=O), 2.09–1.82 (5H, m, OCH_2(C**H**$_2$)$_2$CHC**H**$_2$), 1.54–1.49 (1H, m, OCH_2(C**H**$_2$)$_2$CHC**H**$_2$); ^{13}C NMR (101 MHz, $CDCl_3$) δ 172.1 (C), 150.8 (C), 129.4 (2 x CH), 125.7 (CH), 121. 6 (2 x CH), 78.1 (CH), 67.8 (CH_2), 31.3 (CH_2), 31.2 (CH_2), 30.7 (CH_2), 25.8 (CH_2); HRMS (ESI) Exact mass calculated for $[C_{13}H_{16}O_3Na]^+$ $[M+Na]^+$: 243.0992, found: 243.0996.

1-Phenyl-3-(tetrahydrofuran-2-yl)propan-1-one (29)

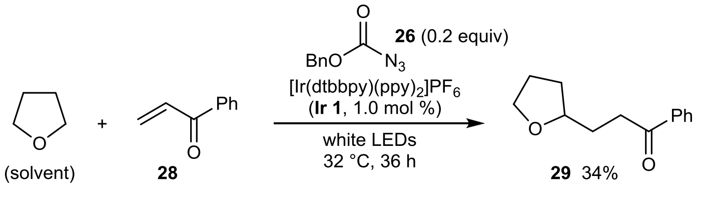

An oven-dried microwave tube equipped with a stirrer bar was charged with $[Ir(dtbbpy)(ppy)_2]PF_6$ (3.7 mg, 0.004 mmol) and phenyl vinyl ketones **28** (52.9 mg, 0.40 mmol). The tube was sealed with a septum-lined cap and purged with nitrogen for 1 h. A solution of benzyl azidoformate **26**[21] (14.2 mg, 0.08 mmol) in THF (2.0 mL) was then added. The tube was then immersed sideways into a sand bath heated to 32 °C, and irradiated by white LEDs (see General Information for details of the light source) with stirring for 36 h (see photograph on page S11). The crude mixture was directly purified by flash

column chromatography on silica gel (10% EtOAc/petrol) to give a colorless gum (27.8 mg, 34%). R_f = 0.25 (10% Et_2O/petrol); IR 2954, 2928, 2858, 1730, 1686 (C=O), 1448, 1069, 743, 691 cm^{-1}; ^1H NMR (400 MHz, $CDCl_3$) δ 8.02–7.92 (2H, m, Ar**H**), 7.58–7.51 (1H, m, Ar**H**), 7.48–7.41 (2H, m, Ar**H**), 3.97–3.81 (2H, m, OC**H**$_2$), 3.73 (1H, td, *J* = 7.9, 6.3 Hz, OC**H**), 3.18 (1H, ddd, *J* = 17.3, 9.1, 5.5 Hz, C**H**$_2$C=O), 3.06 (1H, ddd, *J* = 17.3, 9.0, 6.2 Hz, C**H**$_2$C=O), 2.10–1.95 (2H, m, OCH_2(C**H**$_2$)$_2$), 1.95–1.81 (3H, m, OCH_2(C**H**$_2$)$_2$CHC**H**$_2$), 1.54–1.48 (1H, m, OCH_2(C**H**$_2$)$_2$CHC**H**$_2$); ^{13}C NMR (101 MHz, $CDCl_3$) δ 200.1 (C), 137.0 (C), 132.9 (CH), 128.5 (2 x CH), 128.1 (2 x CH), 78.5 (CH), 67.7 (CH_2), 35.5 (CH_2), 31.5 (CH_2), 30.0 (CH_2), 25.7 (CH_2); HRMS (ESI) Exact mass calculated for $[C_{13}H_{16}O_2Na]^+$ $[M+Na]^+$: 227.1043, found: 227.1046.

Dihydroxylation of 3c

(±)-(4a*S*,7*S*,8*R*,8a*R*)-7,8-Dihydroxy-4,8a-dimethyl-3-tosyl-4a,7,8,8a-tetrahydro-2*H*-chromen-6(5*H*)-one (30)

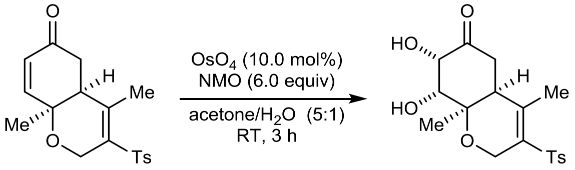

To a vigorously stirred solution of **3c** (166 mg, 0.50 mmol) in acetone (10 mL) and H_2O (2 mL) was added OsO_4 (12.7 mg, 0.005 mmol) followed by *N*-methylmorpholine *N*-oxide (NMO) (234 mg, 2.00 mmol). The reaction mixture was stirred at room temperature for 1.5 h after which additional NMO (117 mg, 1.00 mmol) was added. After stirring for an additional 1.5 h, the solvent was removed in vacuo and the residue was taken up in Et_2O (15 mL) and washed with H_2O (10 mL) and brine (5 mL). The phases were separated and the aqueous layer was extracted with Et_2O (4 x 15 mL). The organic extracts were dried over anhydrous $NaSO_4$ and concentrated then purified by flash chromatography (50% EtOAc/petrol) to give the diol **30** (139 mg, 76%) as a white solid. R_f = 0.50 (50% EtOAc/petrol); m.p. 163–165 °C (petrol); IR 3488, 3335 (OH), 2914, 1718 (C=O), 1318, 1305, 1147, 1111, 1091, 1035, 657, 580 cm^{-1}; ^1H NMR (400 MHz, $CDCl_3$) δ 7.80–7.74 (2H, m, Ar**H**), 7.39–7.35 (2H, m, Ar**H**), 4.62 (1H, dq, *J* = 17.0, 2.2 Hz, OC**H**$_2$), 4.56 (1H, br s, O**H**), 4.53-4.46 (1H, m, OC**H**$_2$), 4.00 (1H, dd, *J* = 3.3, 1.6 Hz, C**H**OH), 3.58 (1H, app d, *J* = 2.8 Hz, C**H**OH), 2.68–2.63 (1H, m, C**H**$_2$C=O), 2.65 (1H, br s, O**H**), 2.46 (3H, s, ArC**H**$_3$), 2.44–2.39 (1H, m, C**H**CH_2), 2.30–2.23 (1H, m, C**H**$_2$C=O), 2.19 (3H, t, *J* = 2.3 Hz, C=CC**H**$_3$), 1.24 (3H, s, OCC**H**$_3$); ^{13}C NMR (101 MHz, $CDCl_3$) δ 207.7 (C), 147.5 (C), 144.7 (C), 138.1 (C), 131.7 (C), 130.0 (2 x CH), 126.9 (2 x CH), 78.5 (CH), 74.3 (CH), 74.2 (C), 61.2 (CH_2), 44.9

(CH), 40.0 (CH_2), 21.6 (CH_3), 19.7 (CH_3), 18.4 (CH_3); HRMS (ESI) Exact mass calculated for $[C_{18}H_{22}SO_6Na]^+$ $[M+Na]^+$: 389.1029, found: 389.1028.

Slow diffusion of petrol into a solution of **30** in CH_2Cl_2 gave crystals that were suitable for X-ray diffraction:

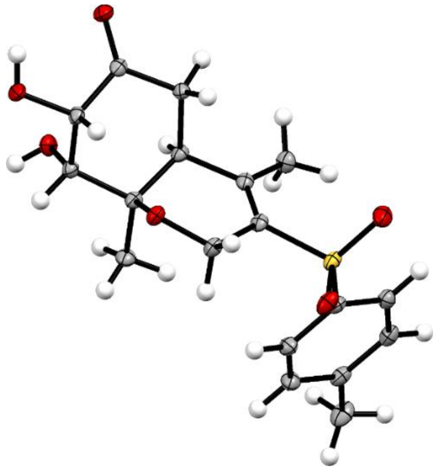

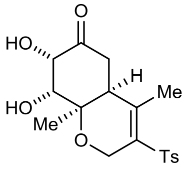

30
CCDC 1492590

Cyclic Voltammetry of TsN_3

Cyclic voltammetry was carried out using a Metrohm Autolab PGSTAT 302N potentiostat and a three-electrode set-up, composed of a 3mm diameter glassy carbon disk working electrode, platinum flag counter electrode, and a silver wire pseudo-reference. Prior to the measurements, both the working and reference electrodes were polished with 0.05 micrometer alumina slurries, while the counter electrode was flame-annealed. The cyclic voltammograms were run at 100 mV/s (scanning to negative potential) in 0.2 M *n*-Bu_4NPF_6 in MeCN purged with argon and kept under an inert atmosphere. The concentration of the analyte was 2 mM. The final values are referenced to SCE by using ferrocene (Fc/Fc^+ couple) as the internal standard (+0.380 V in 0.1 M *n*-Bu_4NPF_6 in MeCN).[22]

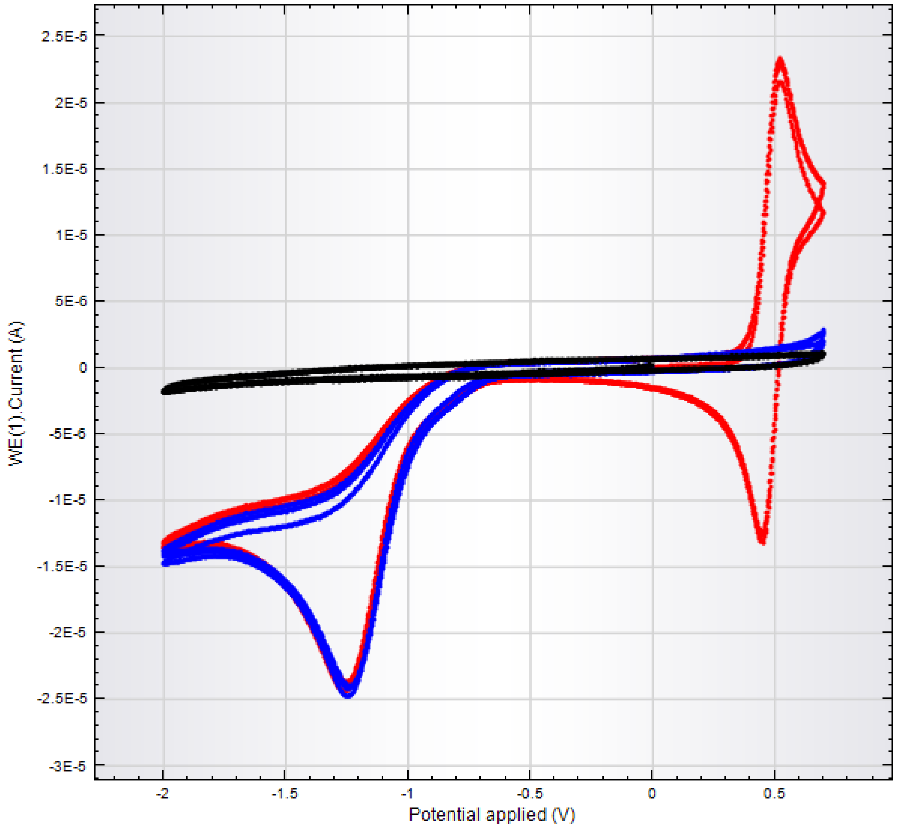

Black line = 0.2 M *n*-Bu_4NPF_6 in MeCN (blank run)

Blue line = 0.002 M TsN_3 in 0.2 M *n*-Bu_4NPF_6 in MeCN

Red line = 0.002 M TsN_3 in 0.2 M *n*-Bu_4NPF_6 in MeCN, with added ferrocene.

Fc vs Ag +0.487 V (from the above voltammogram, measured using NOVA software)

Fc vs SCE +0.380 V (from the literature[22])

Therefore, the conversion factor that needs to be applied = –0.107 V

For TsN_3 vs Ag (from the above voltammogram, measured using NOVA software)

Peak potential	–1.243 V
Half wave potential $E_{1/2}$	–1.109 V
Onset potential	–0.664 V

For TsN_3 vs SCE:

Peak potential	–1.350 V
Half wave potential $E_{1/2}$	–1.216 V
Onset potential	–0.771 V

NMR Spectra

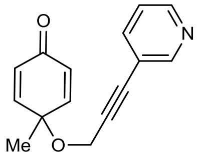

1b

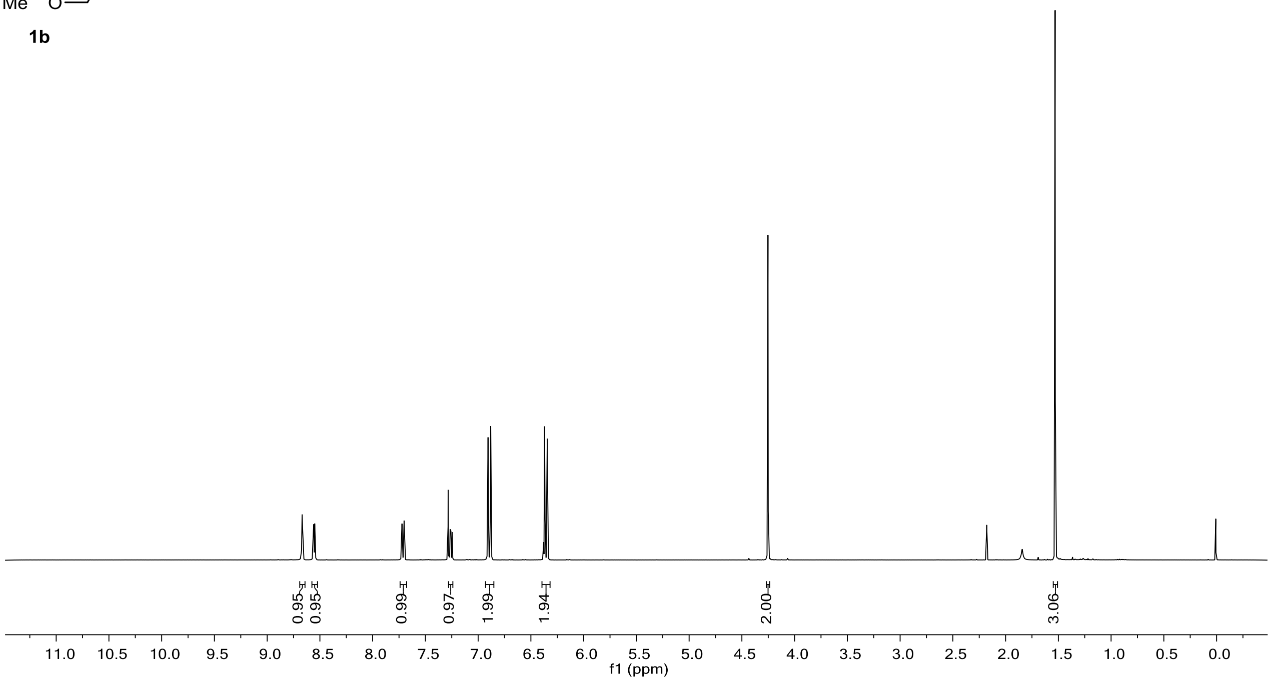

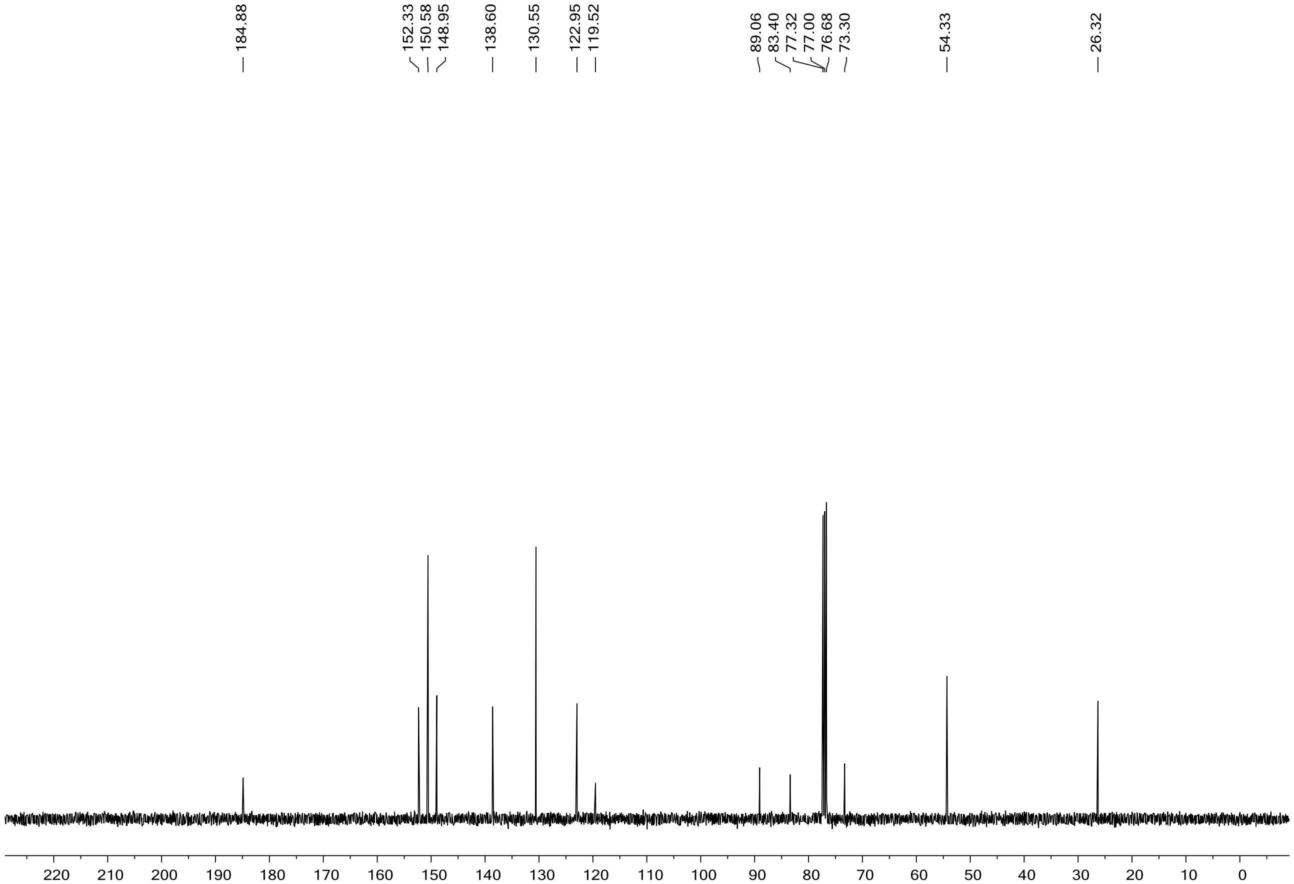

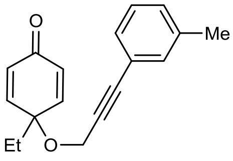
O
Me
Et
O

1d

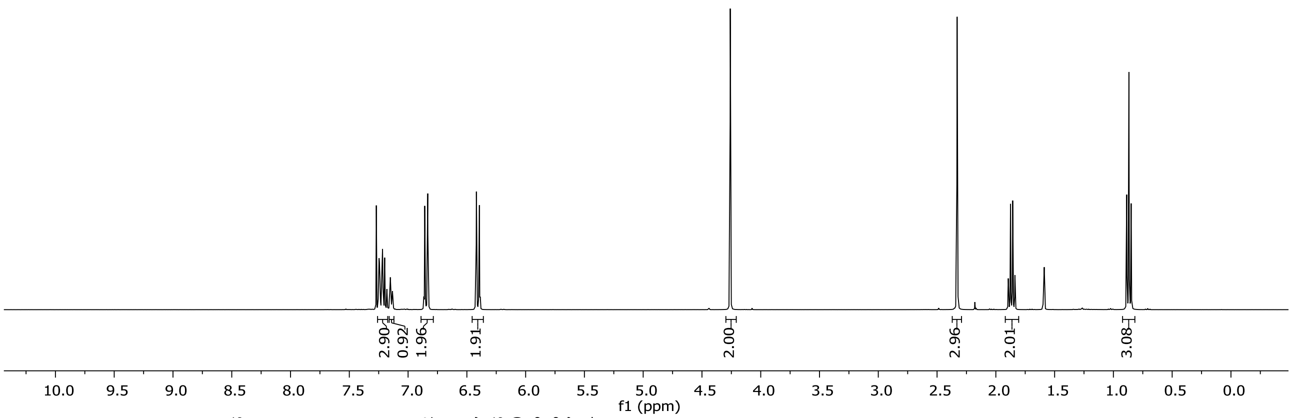
2.90
0.92
1.96
1.91
2.00
2.96
2.01
3.08
10.0 9.5 9.0 8.5 8.0 7.5 7.0 6.5 6.0 5.5 5.0 4.5 4.0 3.5 3.0 2.5 2.0 1.5 1.0 0.5 0.0
f1 (ppm)

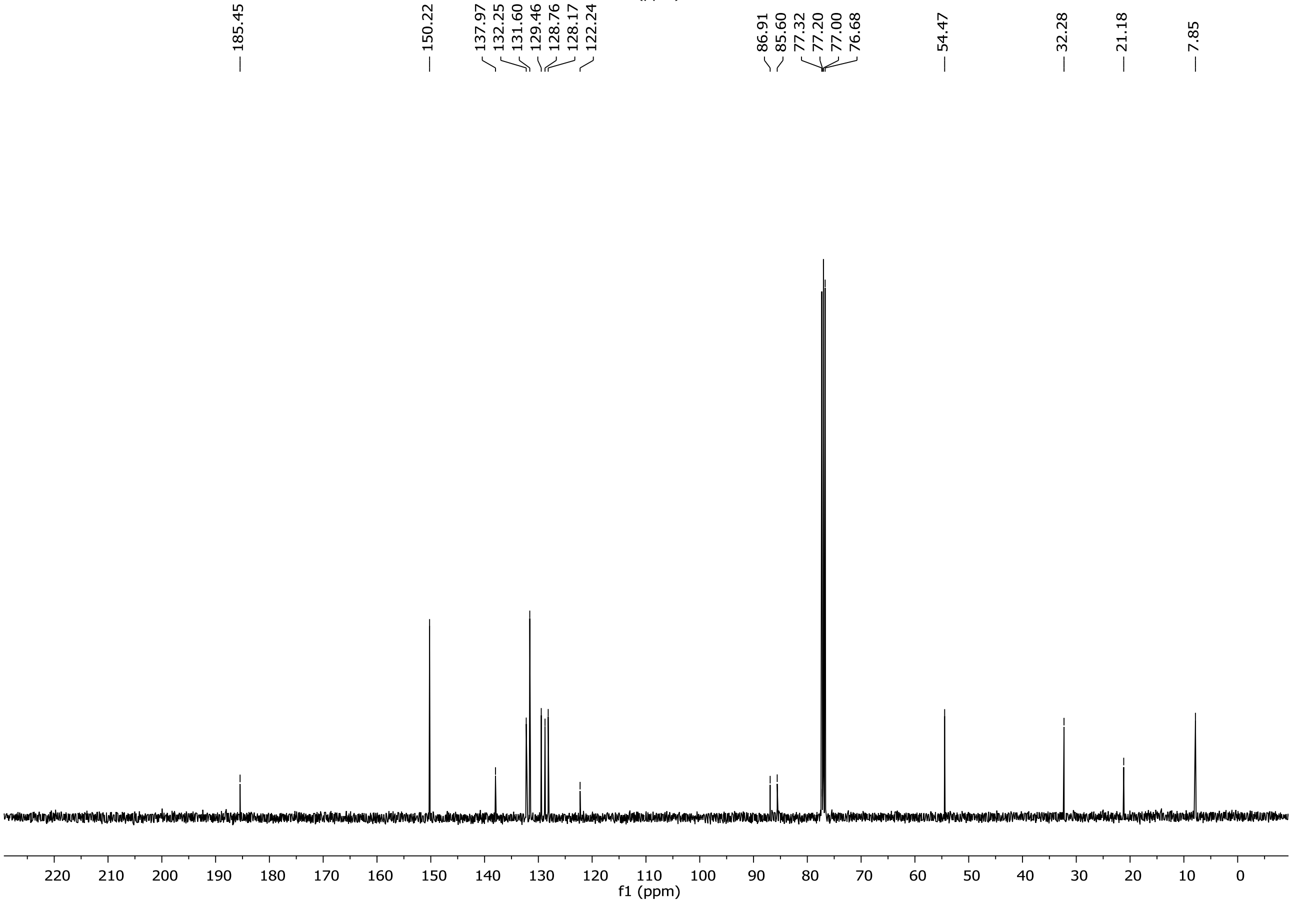
185.45
150.22
137.97
132.25
131.60
129.46
128.76
128.17
122.24
86.91
85.60
77.32
77.20
77.00
76.68
54.47
32.28
21.18
7.85
220 210 200 190 180 170 160 150 140 130 120 110 100 90 80 70 60 50 40 30 20 10 0
f1 (ppm)

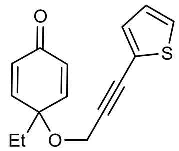
O
S
Et
O

1e

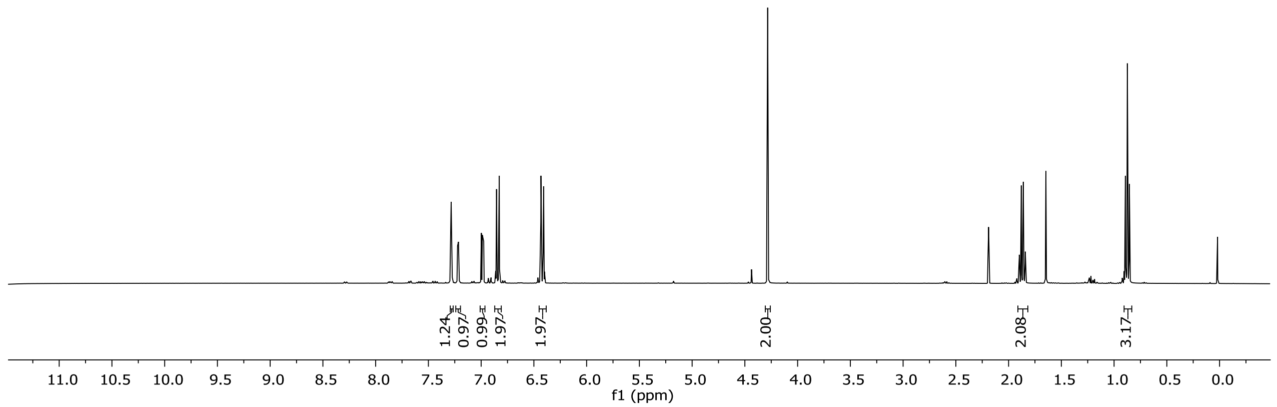
1.24
0.97
0.99
1.97
1.97
2.00
2.08
3.17
11.0 10.5 10.0 9.5 9.0 8.5 8.0 7.5 7.0 6.5 6.0 5.5 5.0 4.5 4.0 3.5 3.0 2.5 2.0 1.5 1.0 0.5 0.0
f1 (ppm)

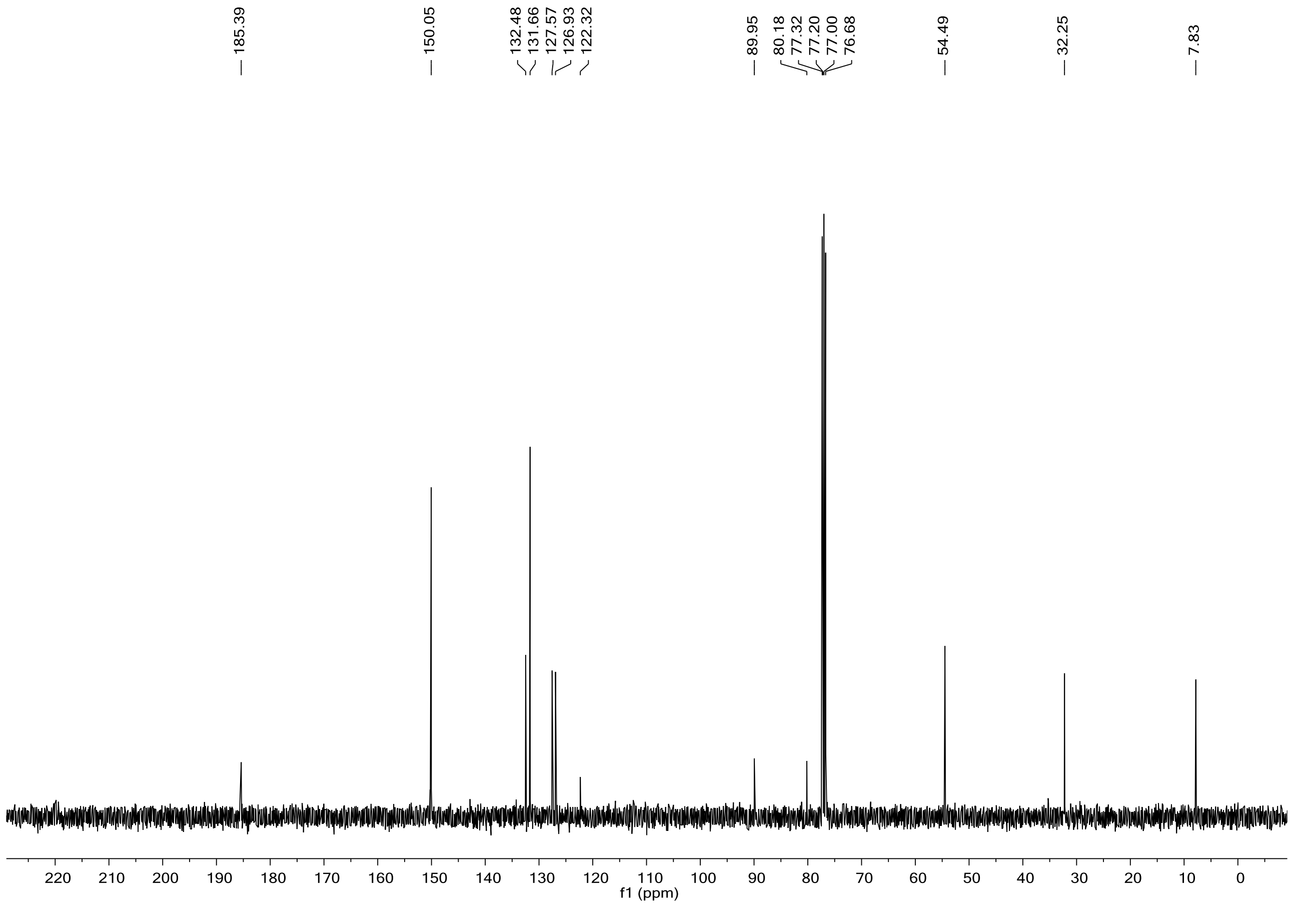
185.39
150.05
132.48
131.66
127.57
126.93
122.32
89.95
80.18
77.32
77.20
77.00
76.68
54.49
32.25
7.83
220 210 200 190 180 170 160 150 140 130 120 110 100 90 80 70 60 50 40 30 20 10 0
f1 (ppm)

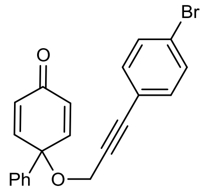
Br
O
Ph
O

1f

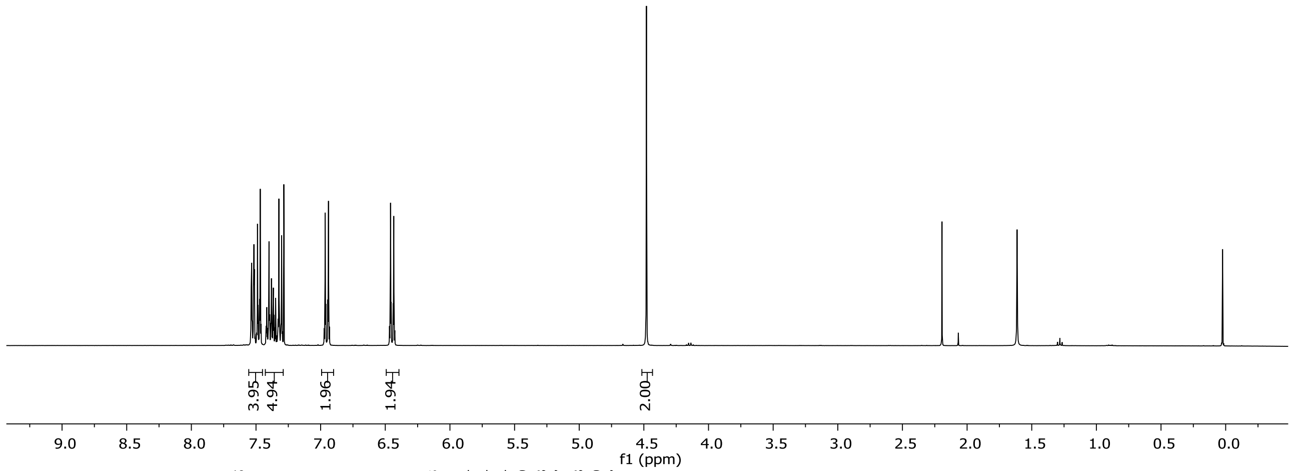
3.95
4.94
1.96
1.94
2.00
9.0 8.5 8.0 7.5 7.0 6.5 6.0 5.5 5.0 4.5 4.0 3.5 3.0 2.5 2.0 1.5 1.0 0.5 0.0
f1 (ppm)

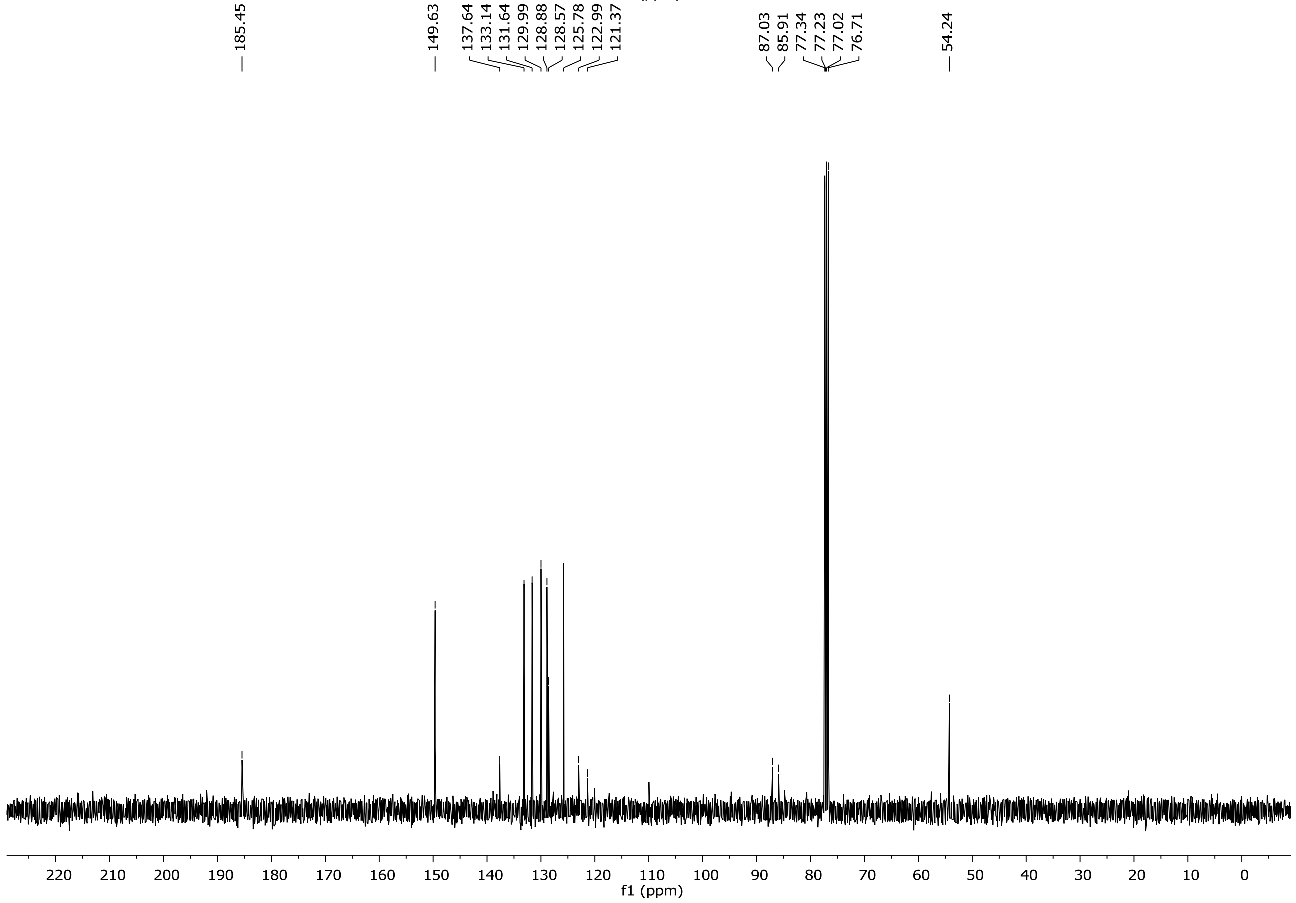
185.45
149.63
137.64
133.14
131.64
129.99
128.88
128.57
125.78
122.99
121.37
87.03
85.91
77.34
77.23
77.02
76.71
54.24
220 210 200 190 180 170 160 150 140 130 120 110 100 90 80 70 60 50 40 30 20 10 0
f1 (ppm)

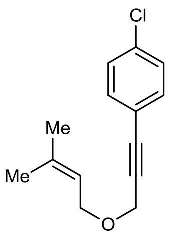
Cl
Me
Me
O

7b

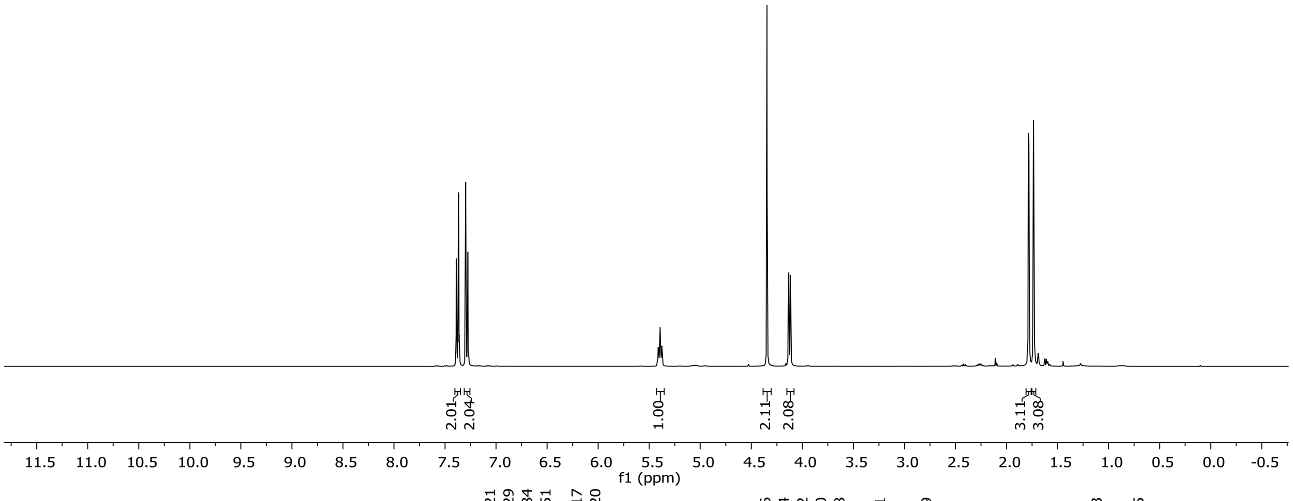
2.01
2.04
1.00
2.11
2.08
3.11
3.08
11.5 11.0 10.5 10.0 9.5 9.0 8.5 8.0 7.5 7.0 6.5 6.0 5.5 5.0 4.5 4.0 3.5 3.0 2.5 2.0 1.5 1.0 0.5 0.0 -0.5
f1 (ppm)

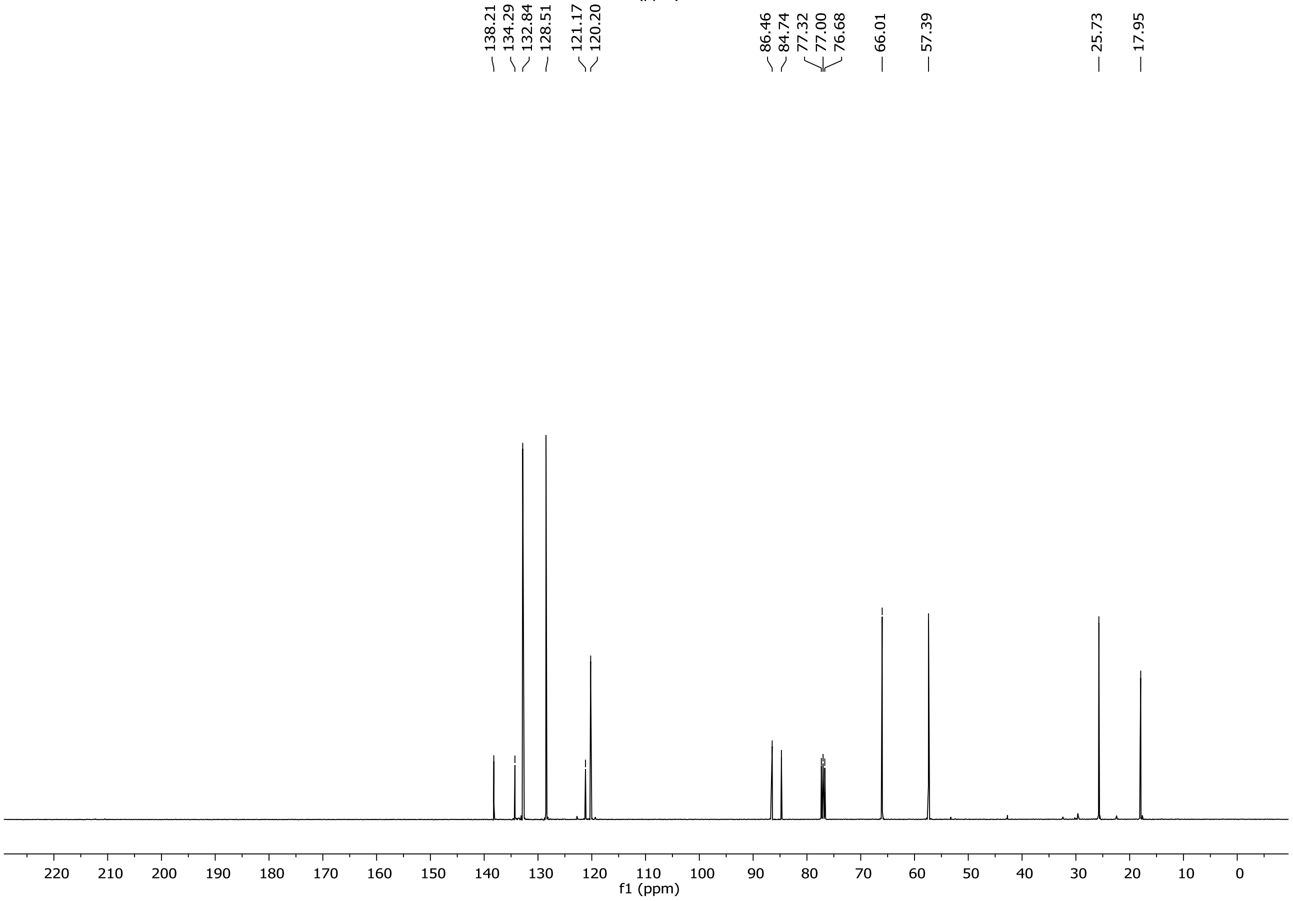
138.21
134.29
132.84
128.51
121.17
120.20
86.46
84.74
77.32
77.00
76.68
66.01
57.39
25.73
17.95
220 210 200 190 180 170 160 150 140 130 120 110 100 90 80 70 60 50 40 30 20 10 0
f1 (ppm)

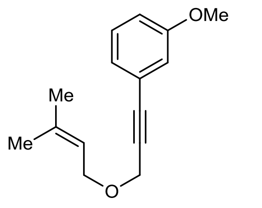
OMe
Me
Me
O

7c

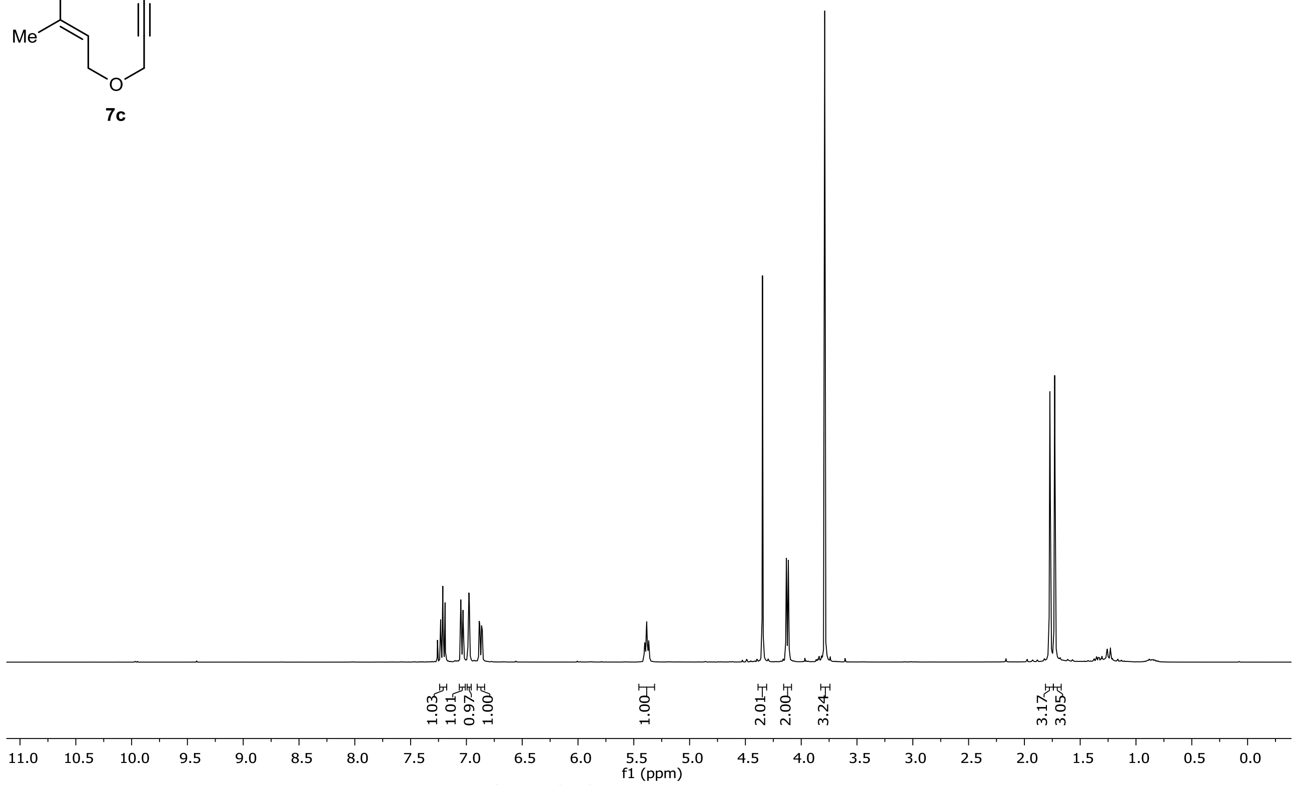
1.03
1.01
0.97
1.00
1.00
2.01
2.00
3.24
3.17
3.05
11.0 10.5 10.0 9.5 9.0 8.5 8.0 7.5 7.0 6.5 6.0 5.5 5.0 4.5 4.0 3.5 3.0 2.5 2.0 1.5 1.0 0.5 0.0
f1 (ppm)

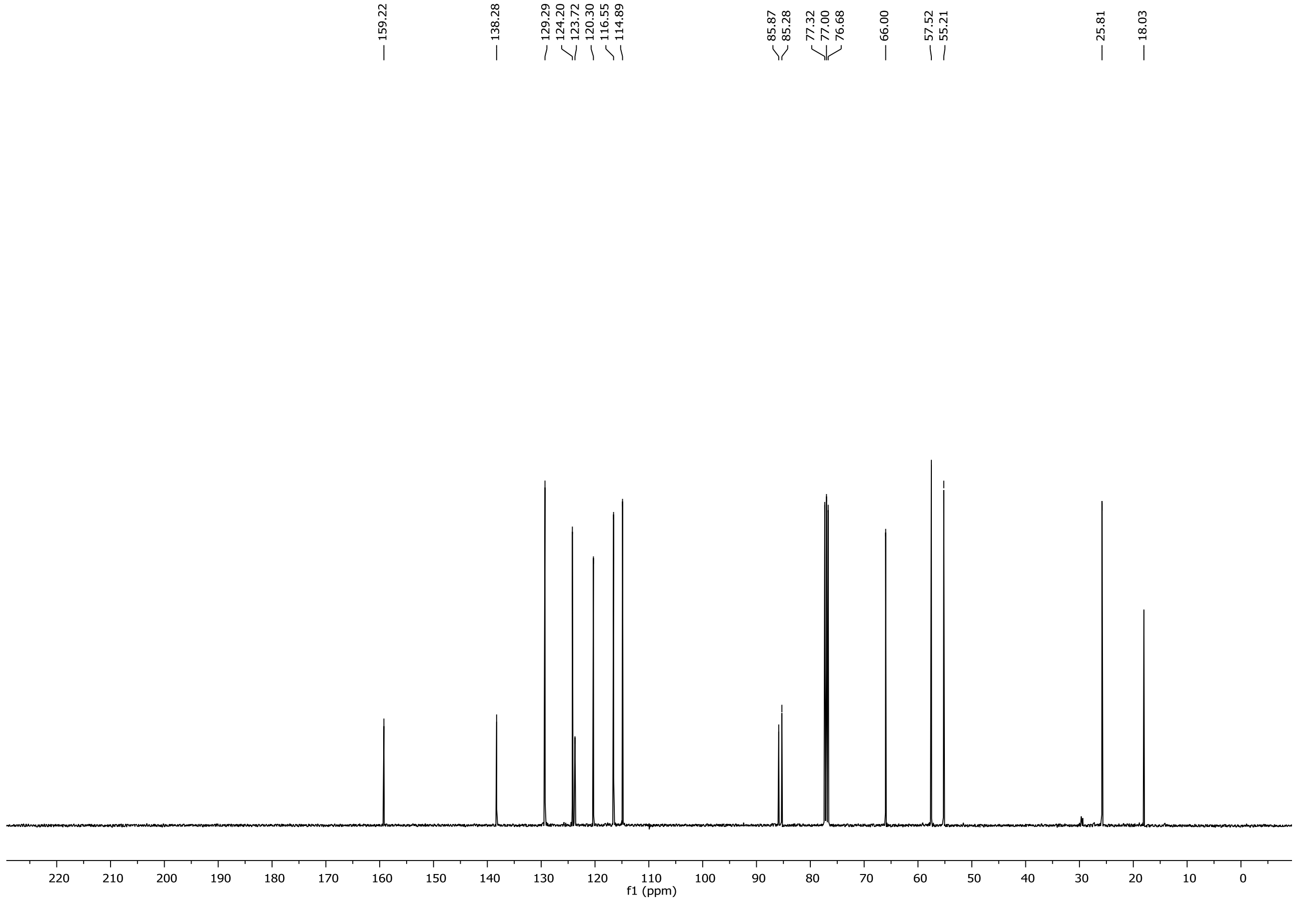
159.22
138.28
129.29
124.20
123.72
120.30
116.55
114.89
85.87
85.28
77.32
77.00
76.68
66.00
57.52
55.21
25.81
18.03
220 210 200 190 180 170 160 150 140 130 120 110 100 90 80 70 60 50 40 30 20 10 0
f1 (ppm)

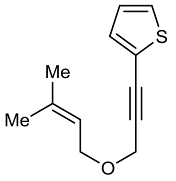
S
Me
Me
O

7d

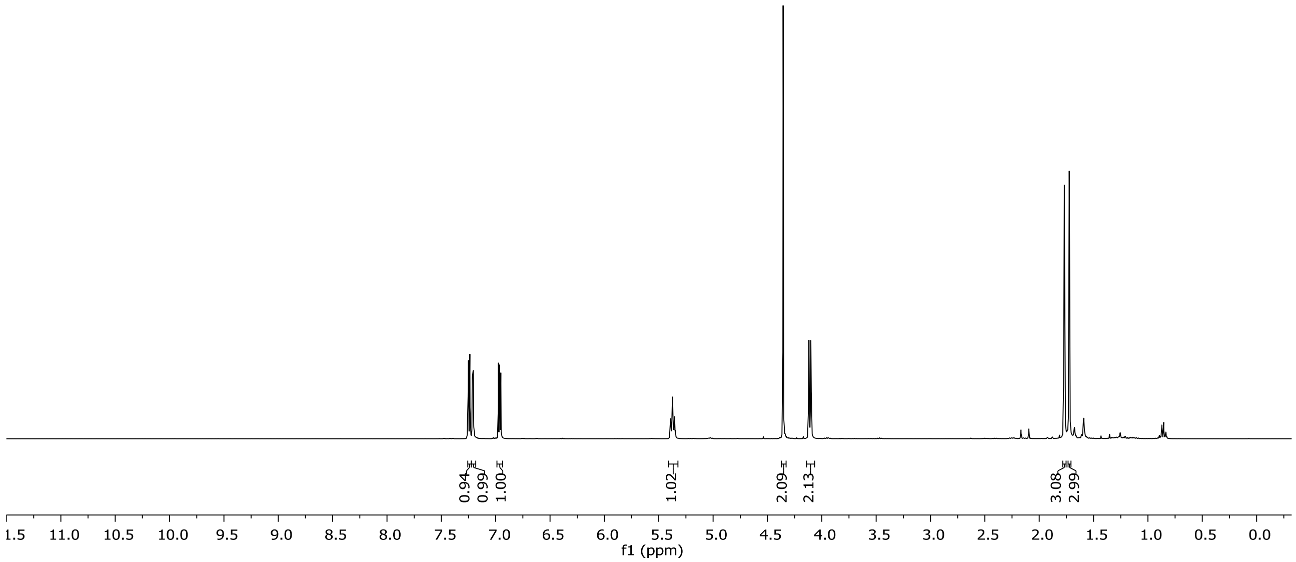
0.94
0.99
1.00
1.02
2.09
2.13
3.08
2.99
f1 (ppm)

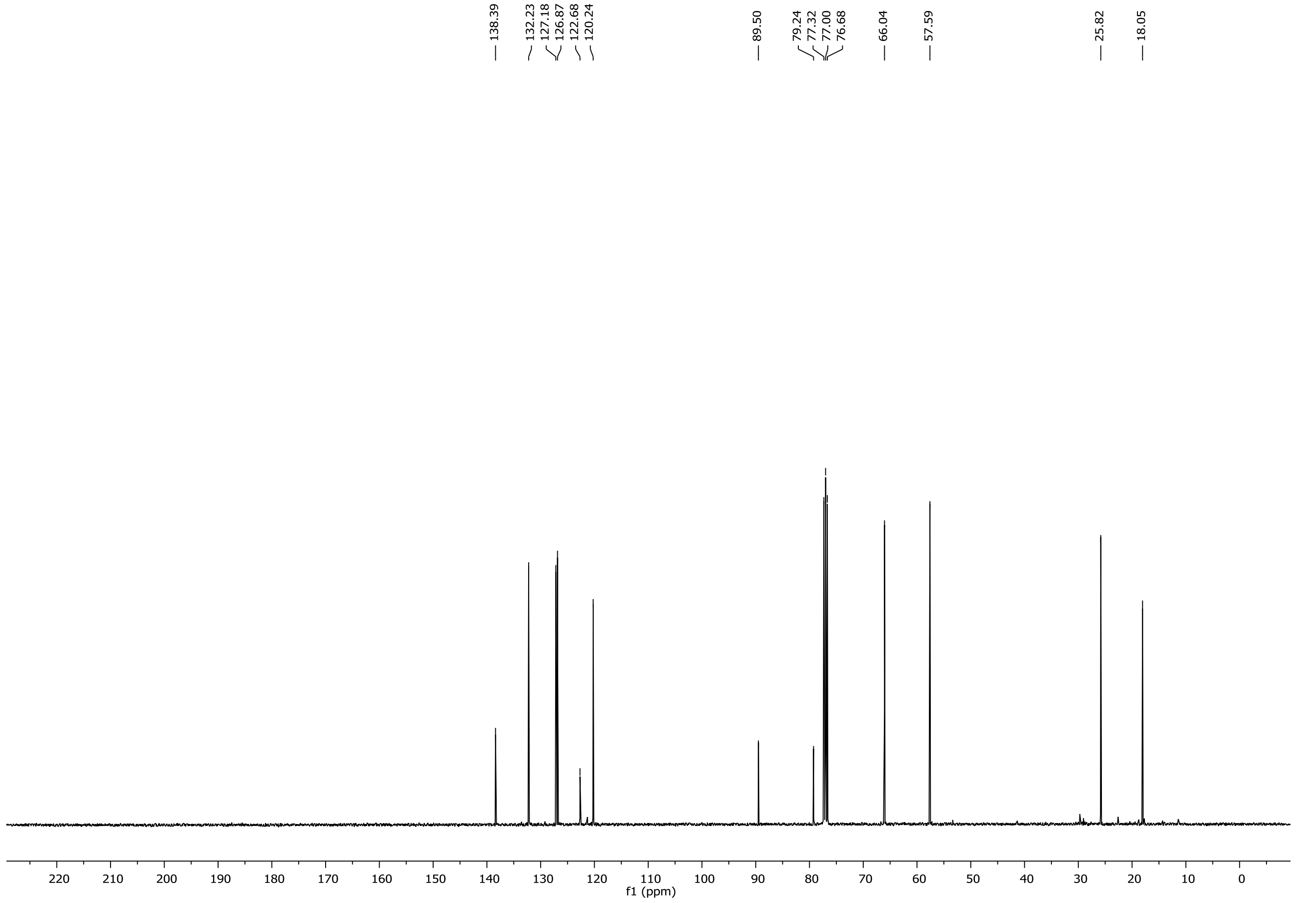
138.39
132.23
127.18
126.87
122.68
120.24
89.50
79.24
77.32
77.00
76.68
66.04
57.59
25.82
18.05
f1 (ppm)

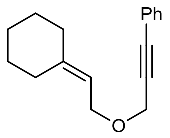
Ph
O

7e

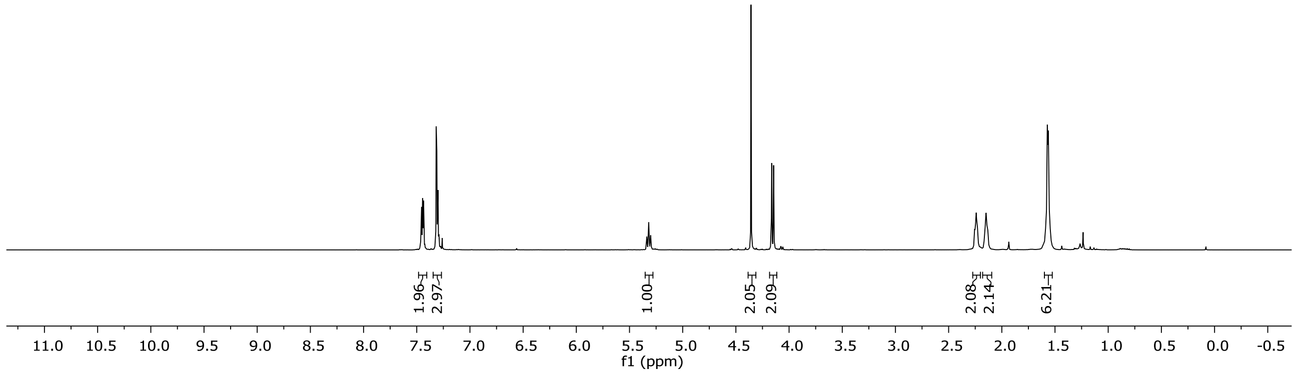
1.96
2.97
1.00
2.05
2.09
2.08
2.14
6.21
11.0
10.5
10.0
9.5
9.0
8.5
8.0
7.5
7.0
6.5
6.0
5.5
5.0
4.5
4.0
3.5
3.0
2.5
2.0
1.5
1.0
0.5
0.0
-0.5
f1 (ppm)

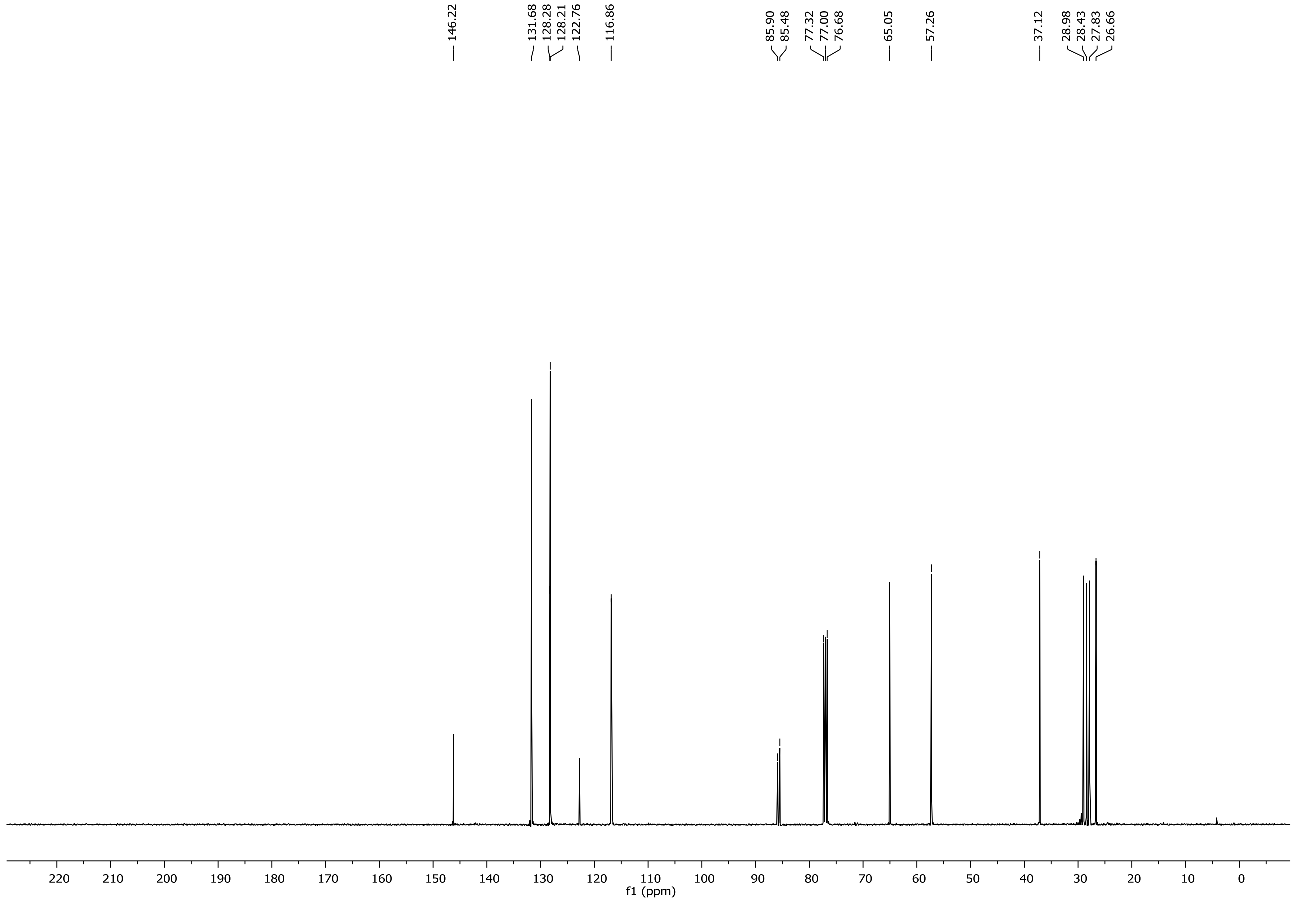
146.22
131.68
128.28
128.21
122.76
116.86
85.90
85.48
77.32
77.00
76.68
65.05
57.26
37.12
28.98
28.43
27.83
26.66
220
210
200
190
180
170
160
150
140
130
120
110
100
90
80
70
60
50
40
30
20
10
0
f1 (ppm)

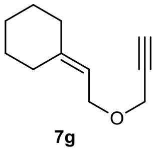

7g

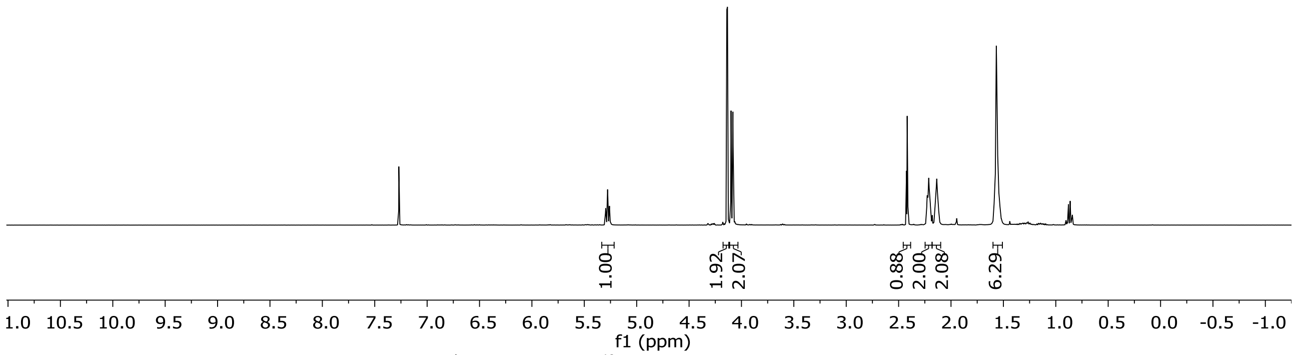
1.00
1.92
2.07
0.88
2.00
2.08
6.29
f1 (ppm)

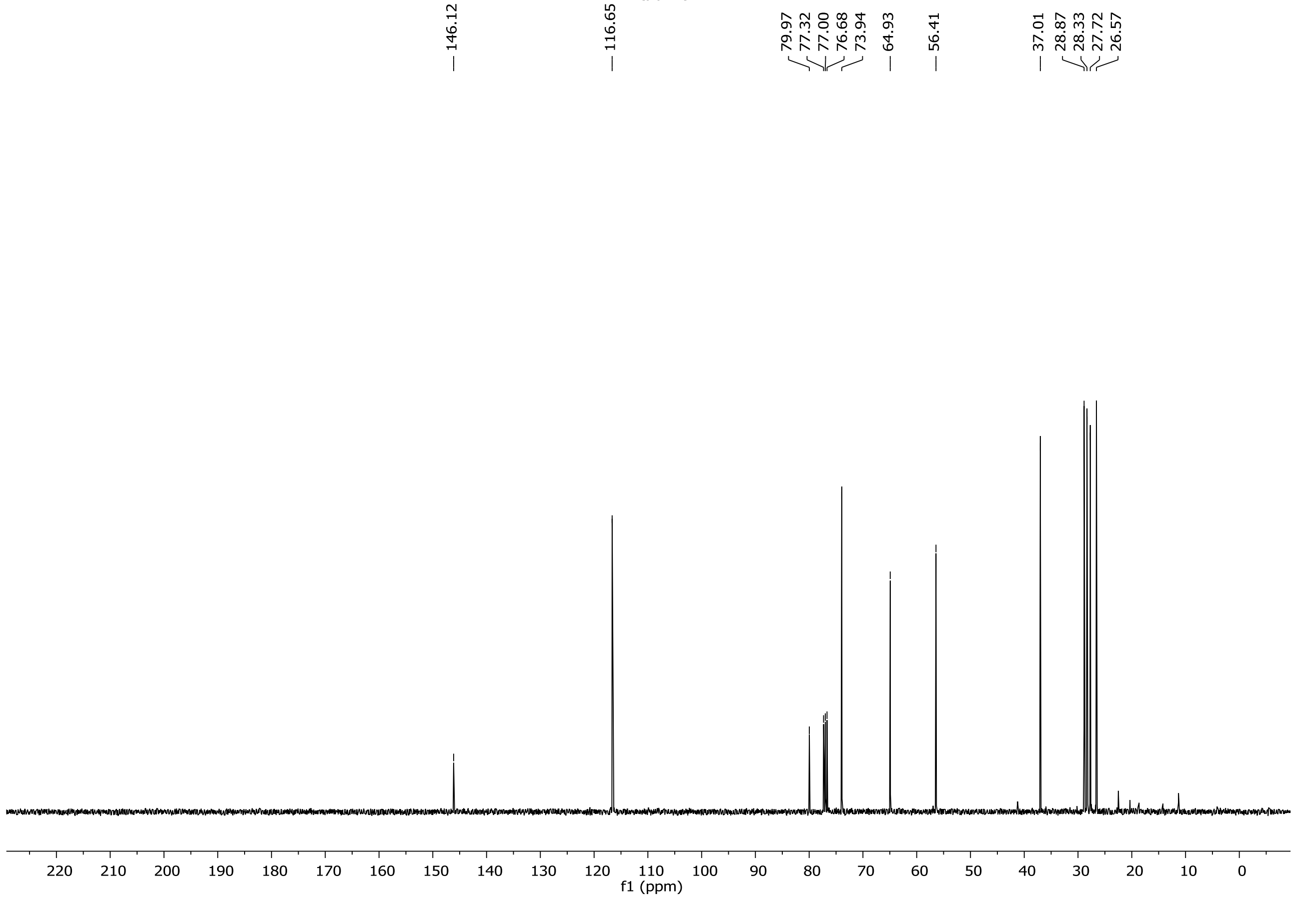
146.12
116.65
79.97
77.32
77.00
76.68
73.94
64.93
56.41
37.01
28.87
28.33
27.72
26.57
f1 (ppm)

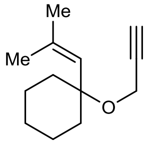
Me
Me
O

7h

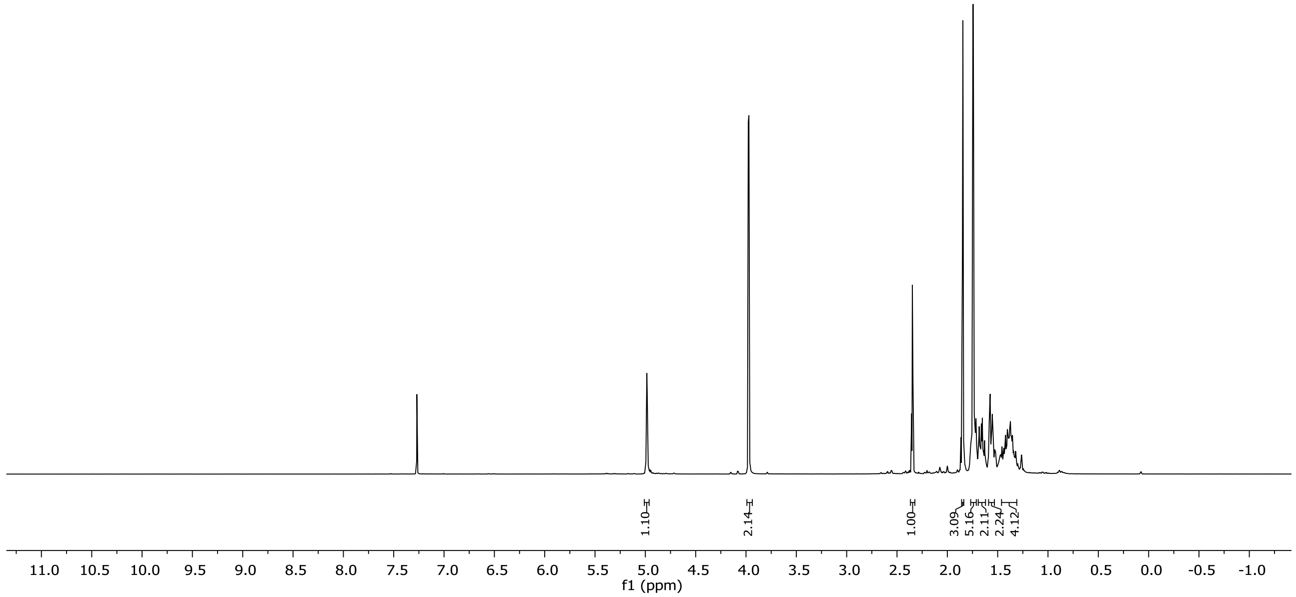
1.10
2.14
1.00
3.09
5.16
2.11
2.24
4.12
11.0 10.5 10.0 9.5 9.0 8.5 8.0 7.5 7.0 6.5 6.0 5.5 5.0 4.5 4.0 3.5 3.0 2.5 2.0 1.5 1.0 0.5 0.0 -0.5 -1.0
f1 (ppm)

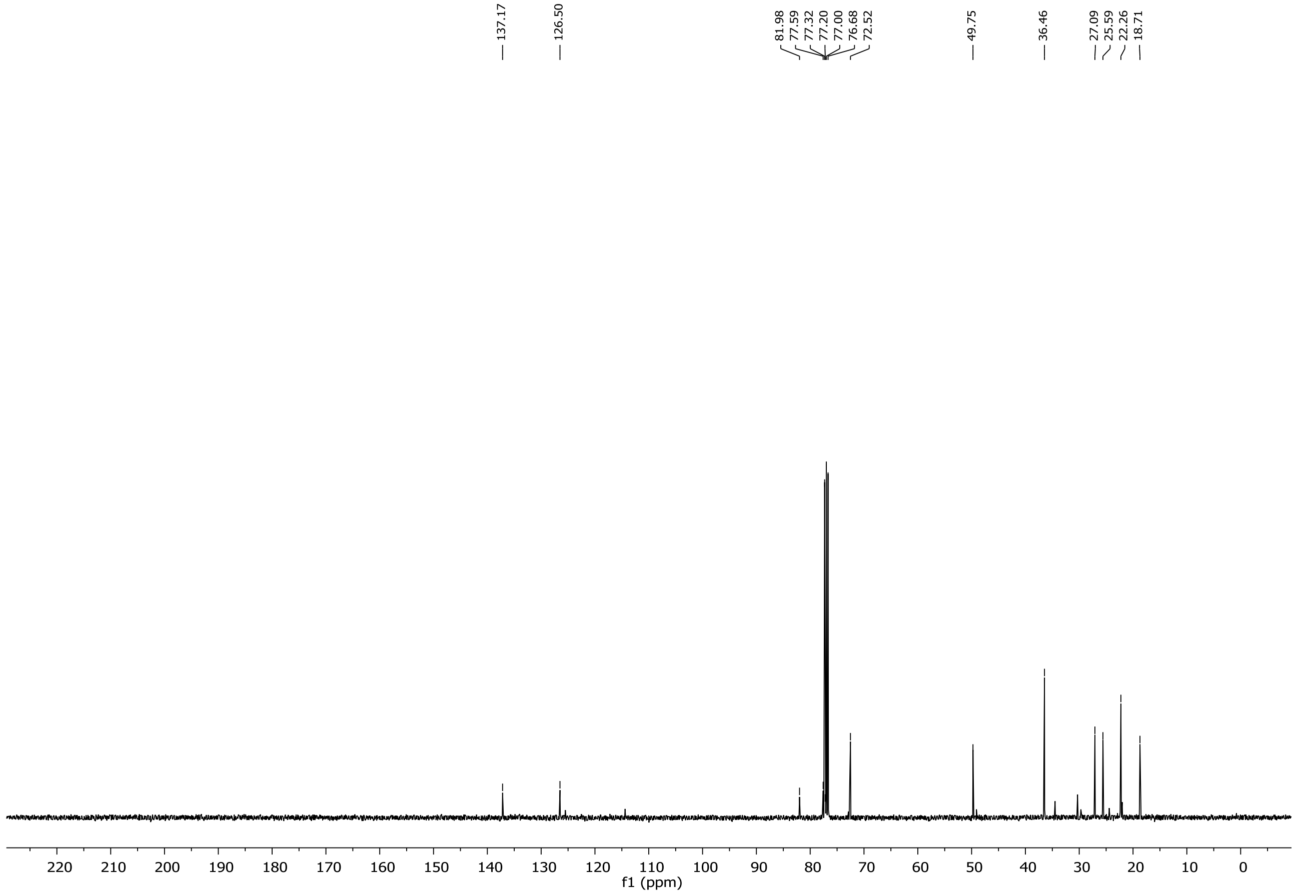
137.17
126.50
81.98
77.59
77.32
77.20
77.00
76.68
72.52
49.75
36.46
27.09
25.59
22.26
18.71
220 210 200 190 180 170 160 150 140 130 120 110 100 90 80 70 60 50 40 30 20 10 0
f1 (ppm)

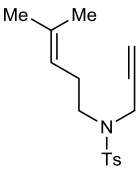
Me
Me
N
Ts

7k

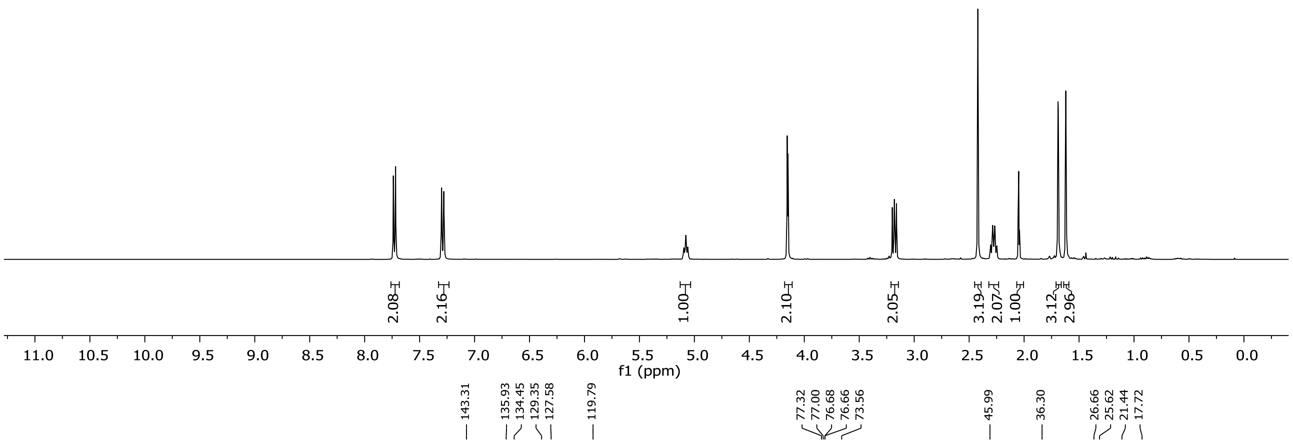
2.08
2.16
1.00
2.10
2.05
3.19
2.07
1.00
3.12
2.96
f1 (ppm)
143.31
135.93
134.45
129.35
127.58
119.79
77.32
77.00
76.68
76.66
73.56
45.99
36.30
26.66
25.62
21.44
17.72

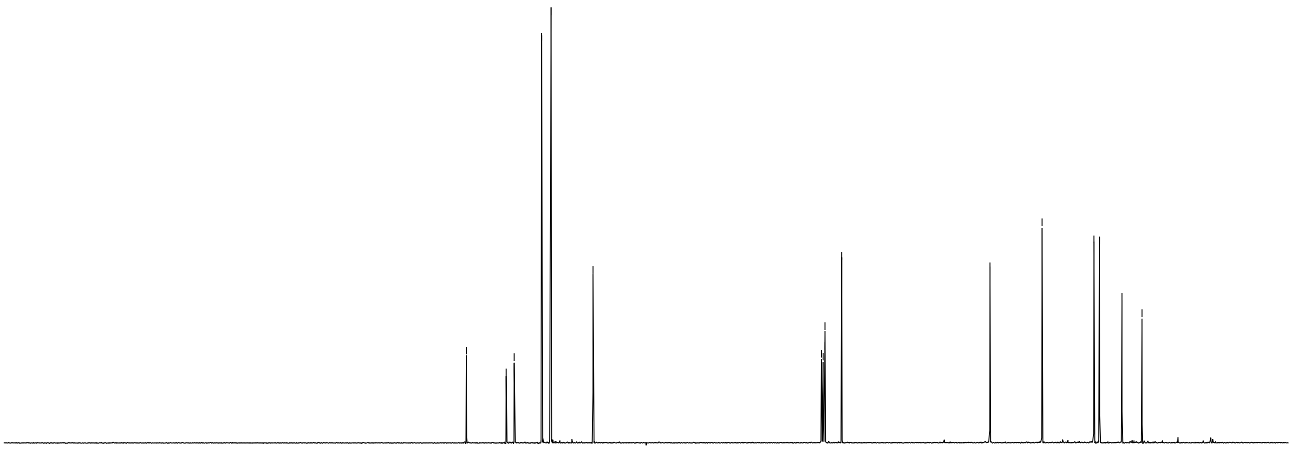
f1 (ppm)

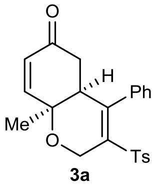
O
H
Ph
Me
O
Ts

3a

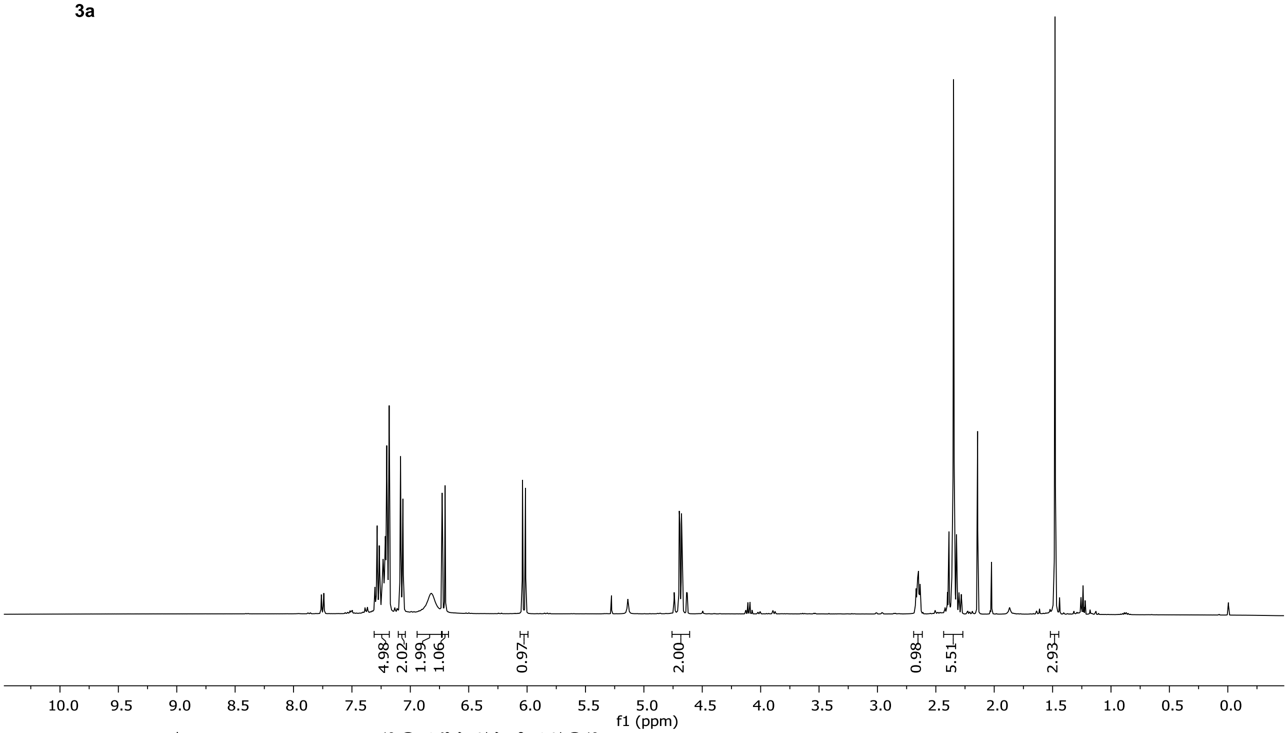
4.98
2.02
1.99
1.06
0.97
2.00
0.98
5.51
2.93
10.0
9.5
9.0
8.5
8.0
7.5
7.0
6.5
6.0
5.5
5.0
4.5
4.0
3.5
3.0
2.5
2.0
1.5
1.0
0.5
0.0
f1 (ppm)

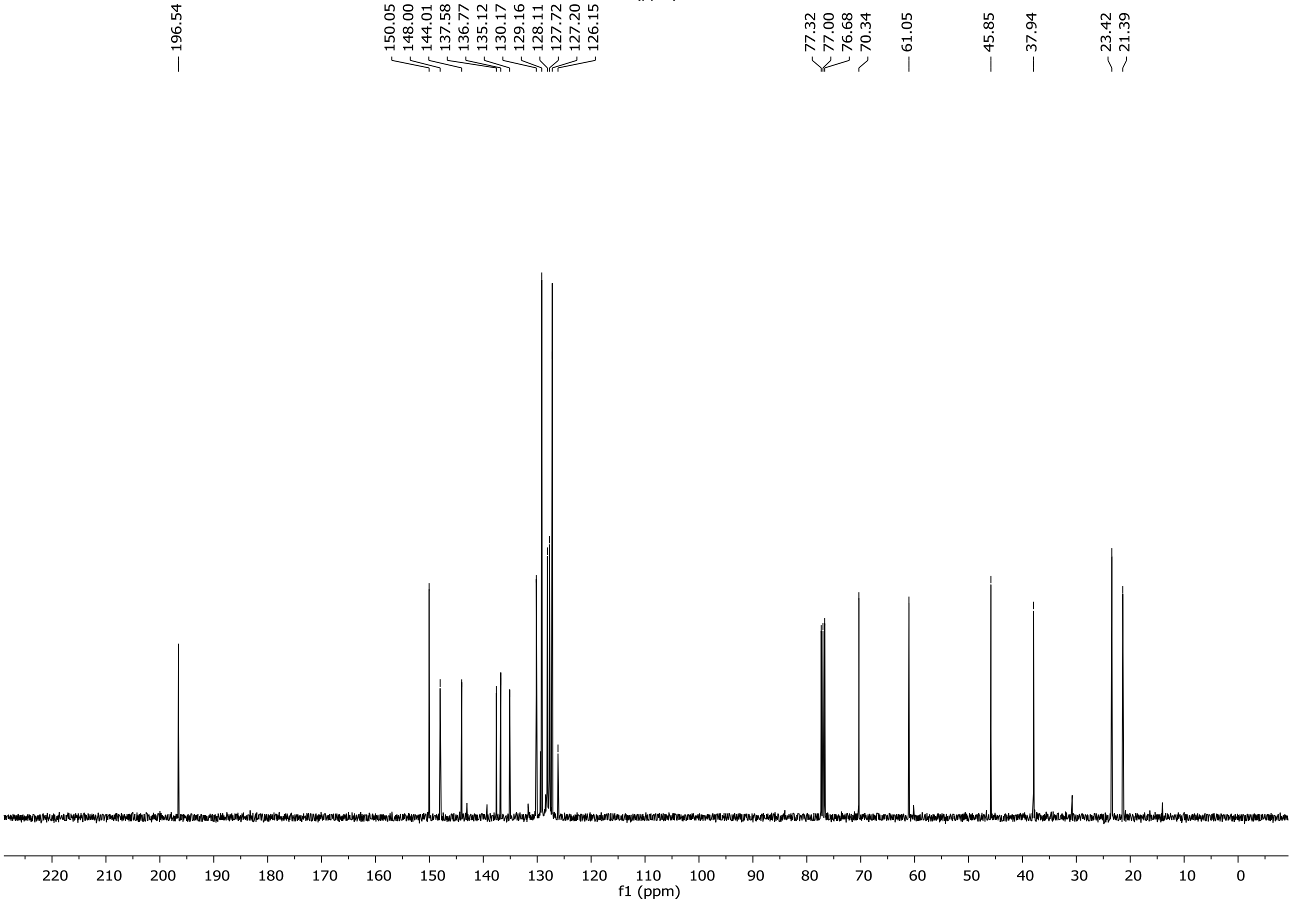
196.54
150.05
148.00
144.01
137.58
136.77
135.12
130.17
129.16
128.11
127.72
127.20
126.15
77.32
77.00
76.68
70.34
61.05
45.85
37.94
23.42
21.39
220
210
200
190
180
170
160
150
140
130
120
110
100
90
80
70
60
50
40
30
20
10
0
f1 (ppm)

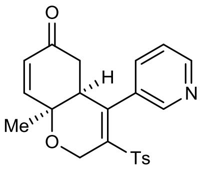
O
H
N
Me
O
Ts

3b

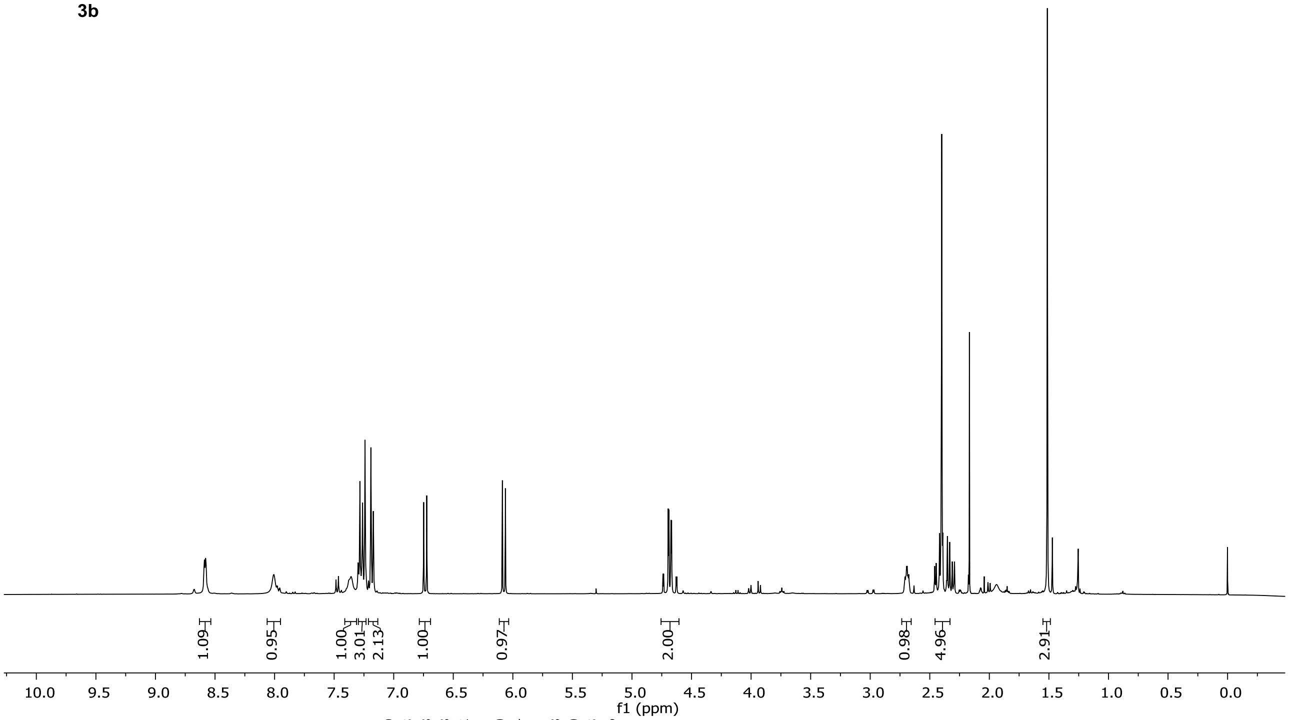
1.09
0.95
1.00
3.01
2.13
1.00
0.97
2.00
0.98
4.96
2.91
10.0 9.5 9.0 8.5 8.0 7.5 7.0 6.5 6.0 5.5 5.0 4.5 4.0 3.5 3.0 2.5 2.0 1.5 1.0 0.5 0.0
f1 (ppm)

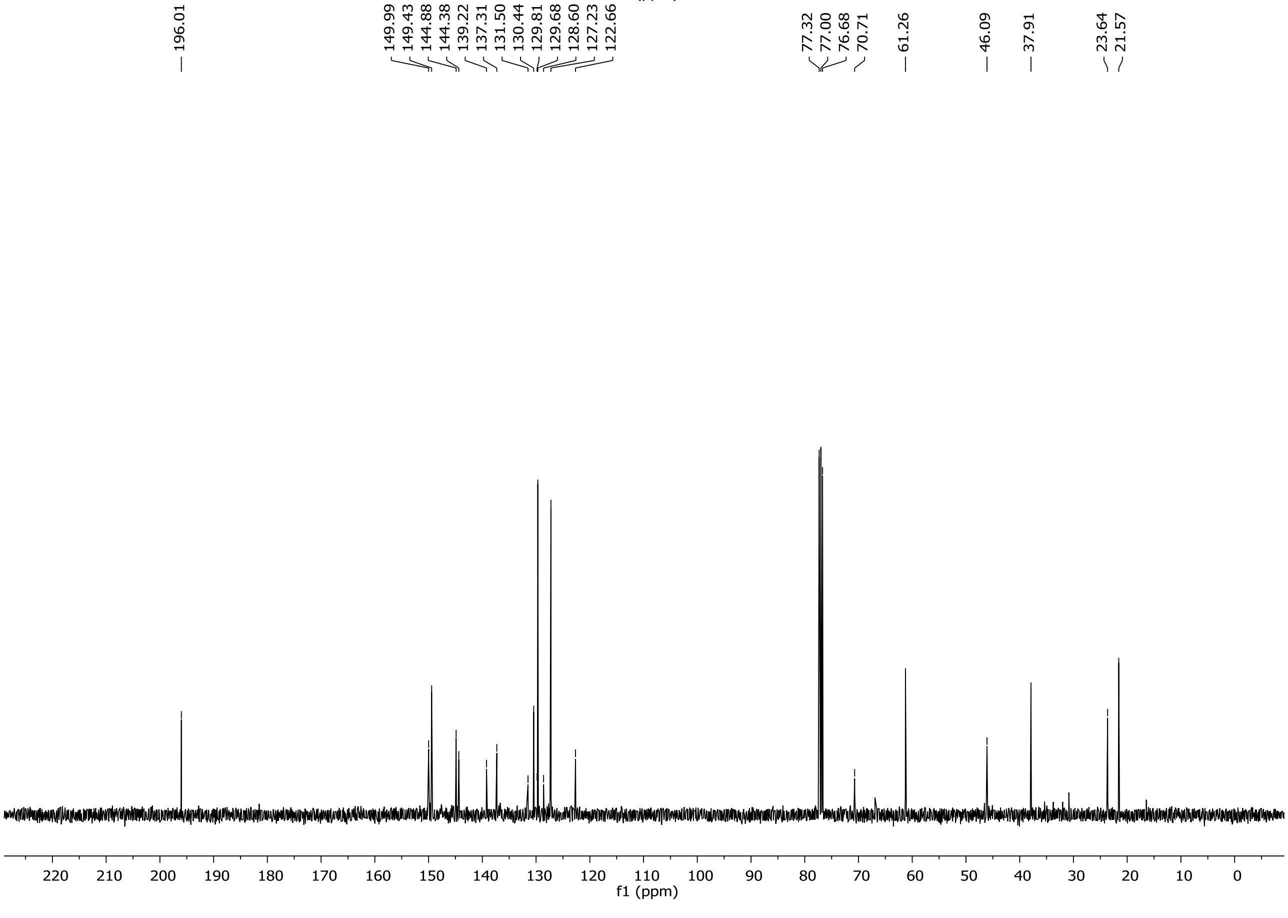
196.01
149.99
149.43
144.88
144.38
139.22
137.31
131.50
130.44
129.81
129.68
128.60
127.23
122.66
77.32
77.00
76.68
70.71
61.26
46.09
37.91
23.64
21.57
220 210 200 190 180 170 160 150 140 130 120 110 100 90 80 70 60 50 40 30 20 10 0
f1 (ppm)

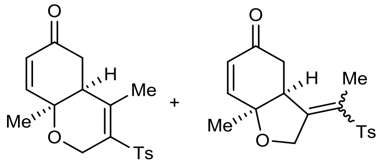
O
H
Me
Me
O
Ts
+
O
H
Me
Me
O
Ts

3c **S10**

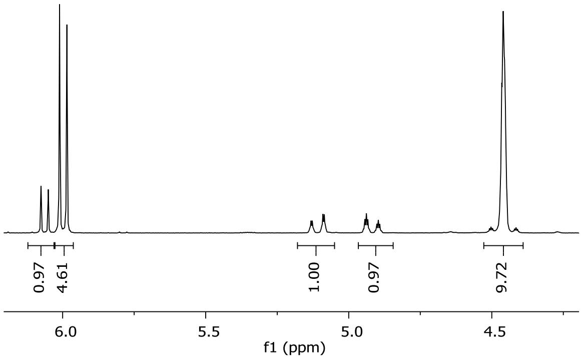
0.97
4.61
1.00
0.97
9.72
6.0
5.5
5.0
4.5
f1 (ppm)

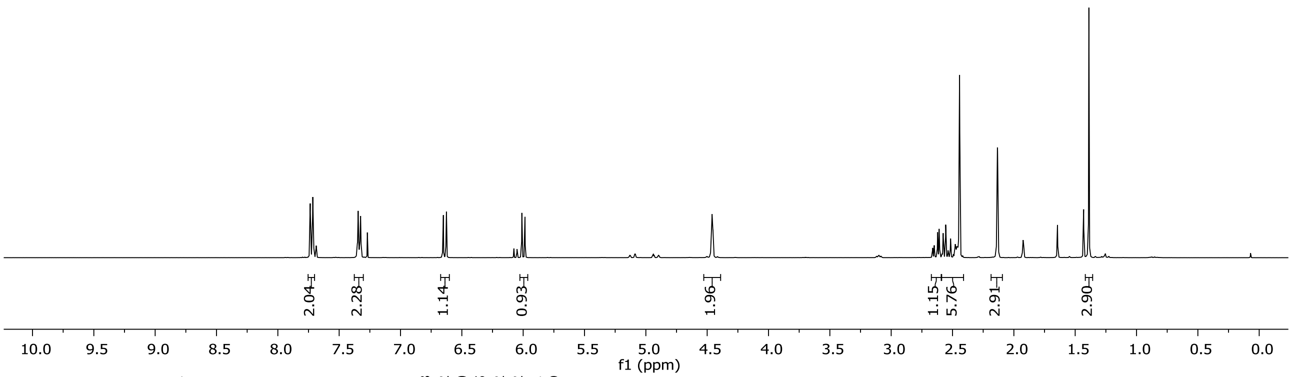
2.04
2.28
1.14
0.93
1.96
1.15
5.76
2.91
2.90
10.0 9.5 9.0 8.5 8.0 7.5 7.0 6.5 6.0 5.5 5.0 4.5 4.0 3.5 3.0 2.5 2.0 1.5 1.0 0.5 0.0
f1 (ppm)

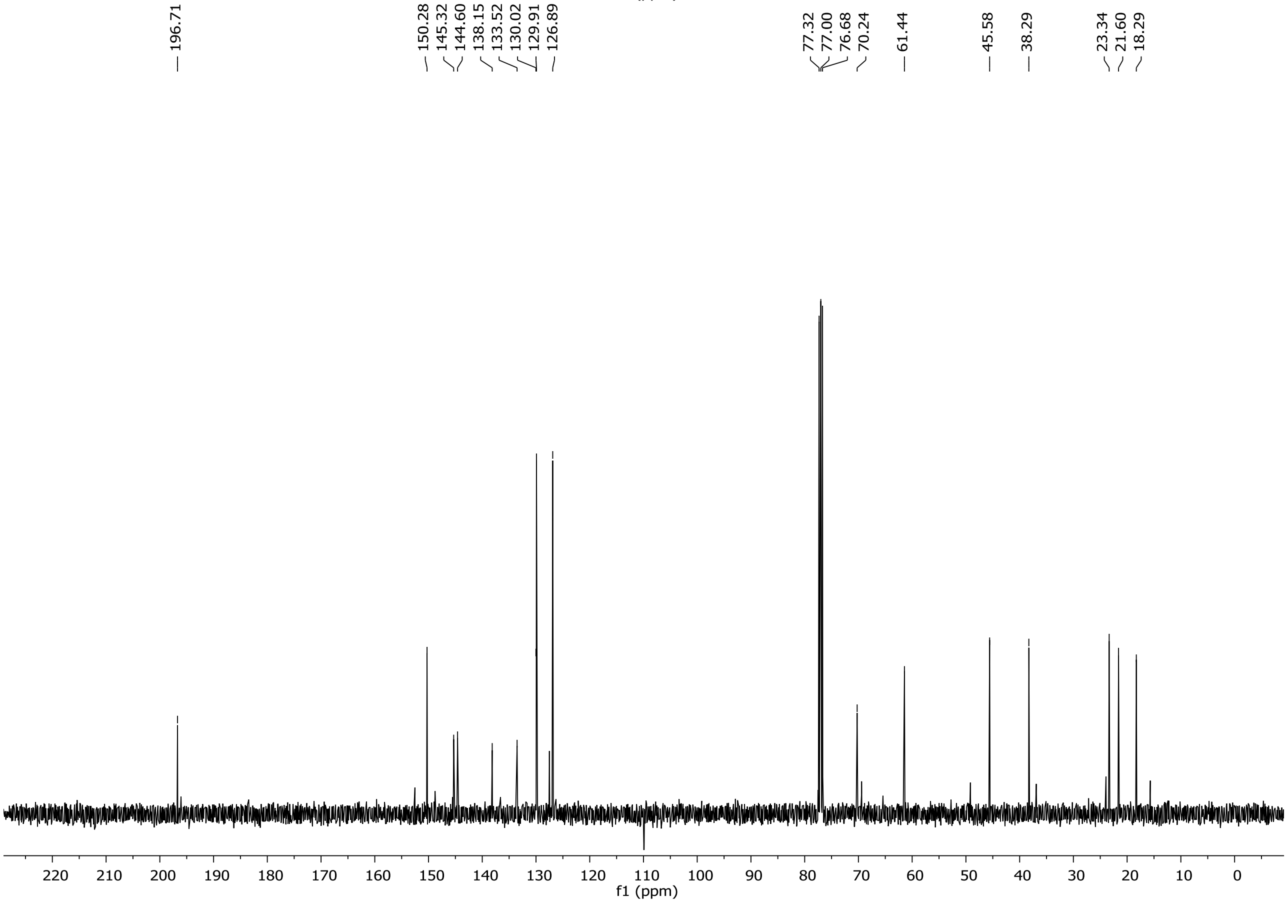
196.71
150.28
145.32
144.60
138.15
133.52
130.02
129.91
126.89
77.32
77.00
76.68
70.24
61.44
45.58
38.29
23.34
21.60
18.29
220 210 200 190 180 170 160 150 140 130 120 110 100 90 80 70 60 50 40 30 20 10 0
f1 (ppm)

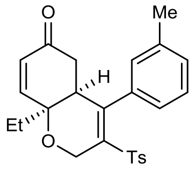
O
Me
H
Et
O
Ts

3d

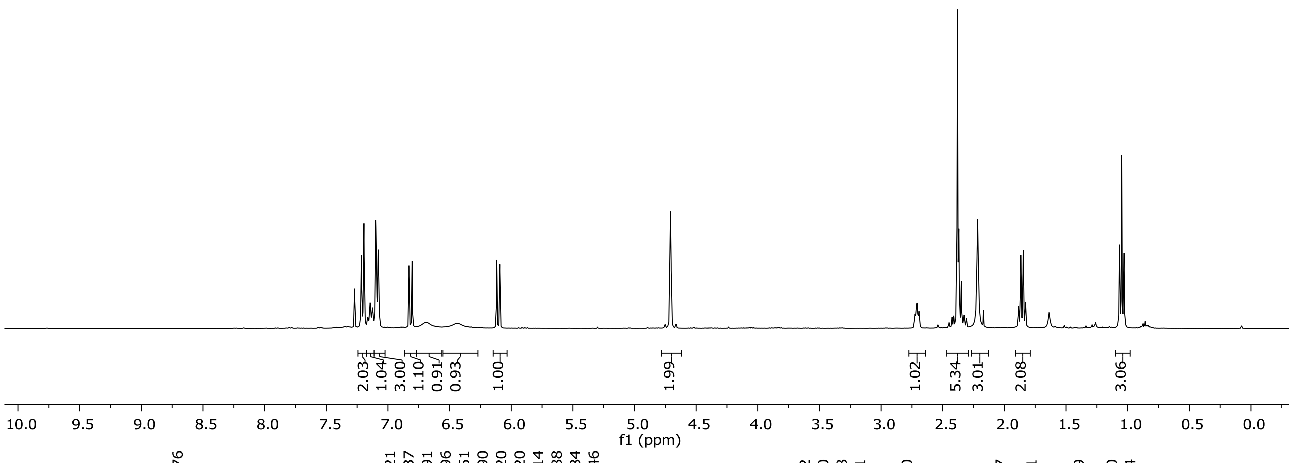
2.03
1.04
3.00
1.10
0.91
0.93
1.00
1.99
1.02
5.34
3.01
2.08
3.06
10.0 9.5 9.0 8.5 8.0 7.5 7.0 6.5 6.0 5.5 5.0 4.5 4.0 3.5 3.0 2.5 2.0 1.5 1.0 0.5 0.0
f1 (ppm)

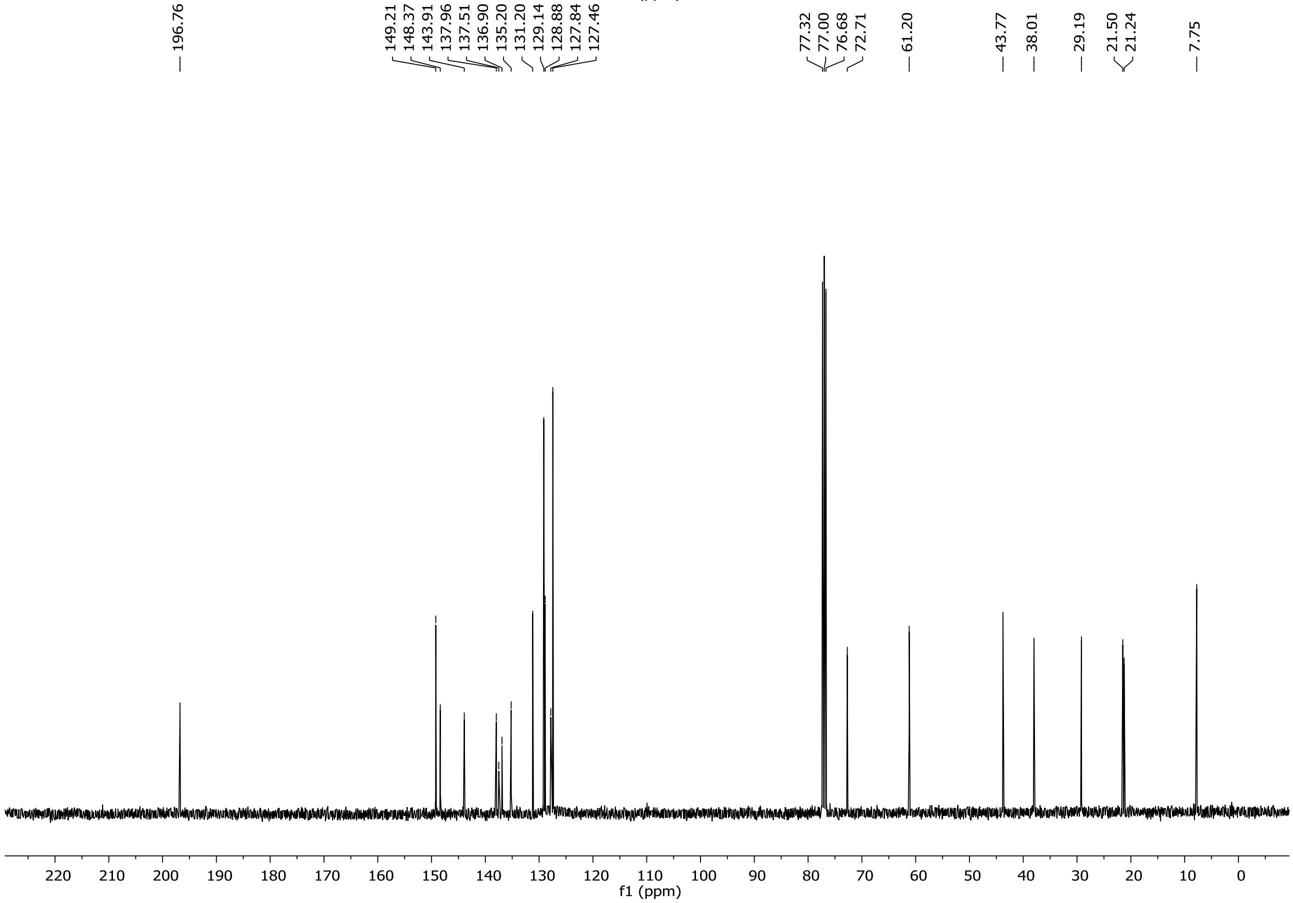
196.76
149.21
148.37
143.91
137.96
137.51
136.90
135.20
131.20
129.14
128.88
127.84
127.46
77.32
77.00
76.68
72.71
61.20
43.77
38.01
29.19
21.50
21.24
7.75
220 210 200 190 180 170 160 150 140 130 120 110 100 90 80 70 60 50 40 30 20 10 0
f1 (ppm)

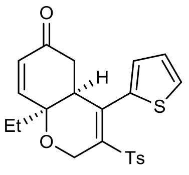
O
H
S
Et
O
Ts

3e

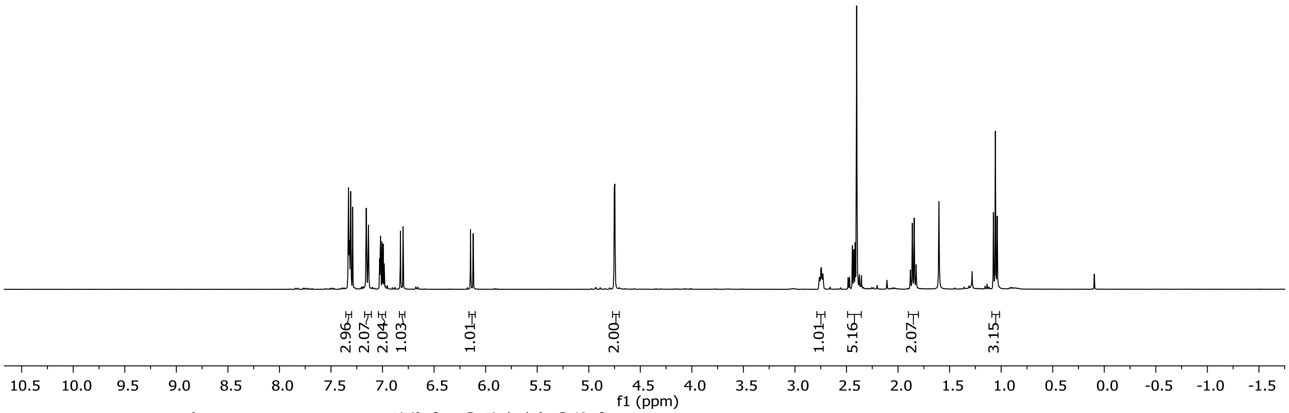
2.96
2.07
2.04
1.03
1.01
2.00
1.01
5.16
2.07
3.15
10.5 10.0 9.5 9.0 8.5 8.0 7.5 7.0 6.5 6.0 5.5 5.0 4.5 4.0 3.5 3.0 2.5 2.0 1.5 1.0 0.5 0.0 -0.5 -1.0 -1.5
f1 (ppm)

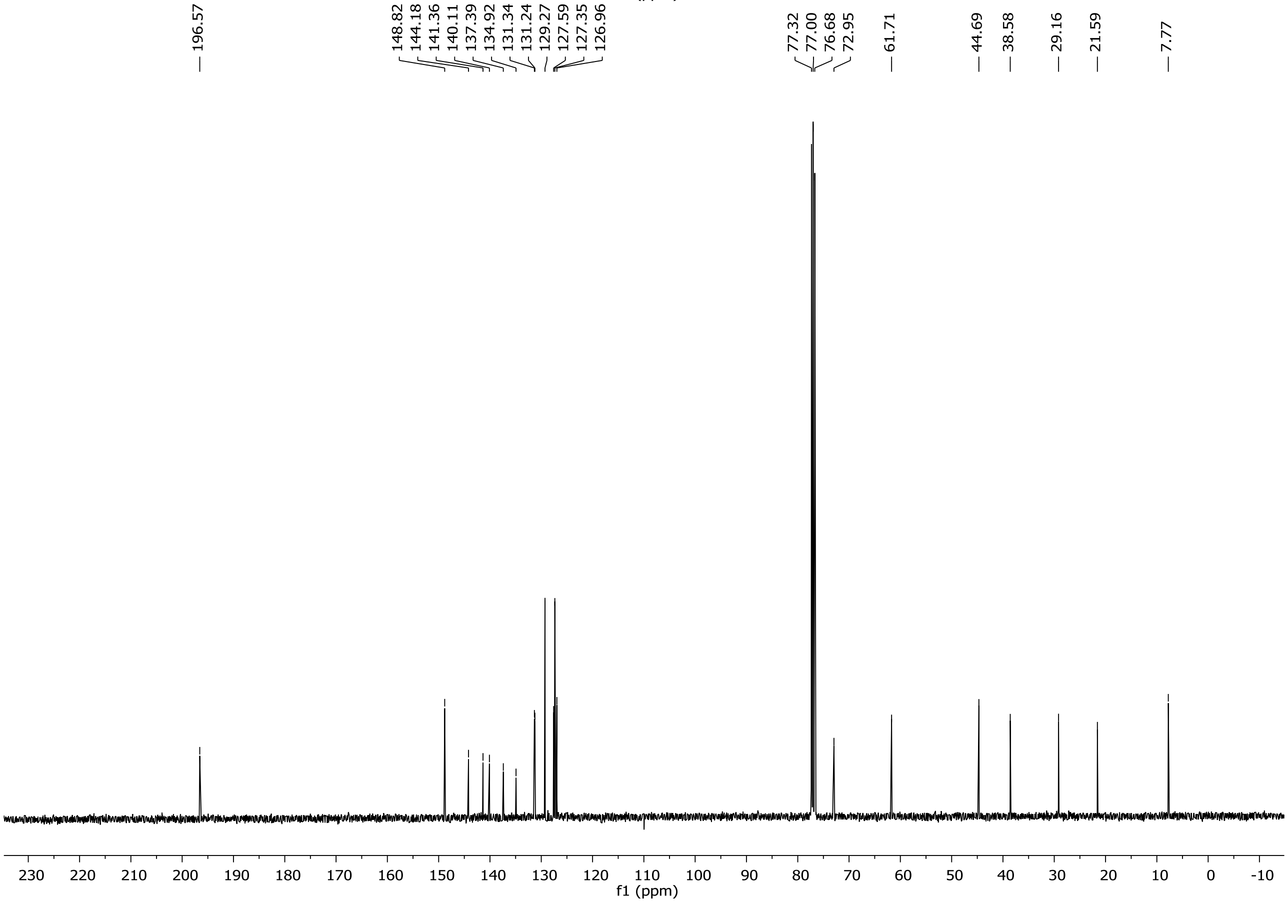
196.57
148.82
144.18
141.36
140.11
137.39
134.92
131.34
131.24
129.27
127.59
127.35
126.96
77.32
77.00
76.68
72.95
61.71
44.69
38.58
29.16
21.59
7.77
230 220 210 200 190 180 170 160 150 140 130 120 110 100 90 80 70 60 50 40 30 20 10 0 -10
f1 (ppm)

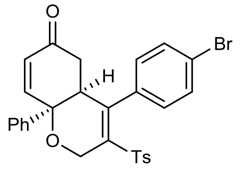
O
H
Br
Ph
O
Ts

3f

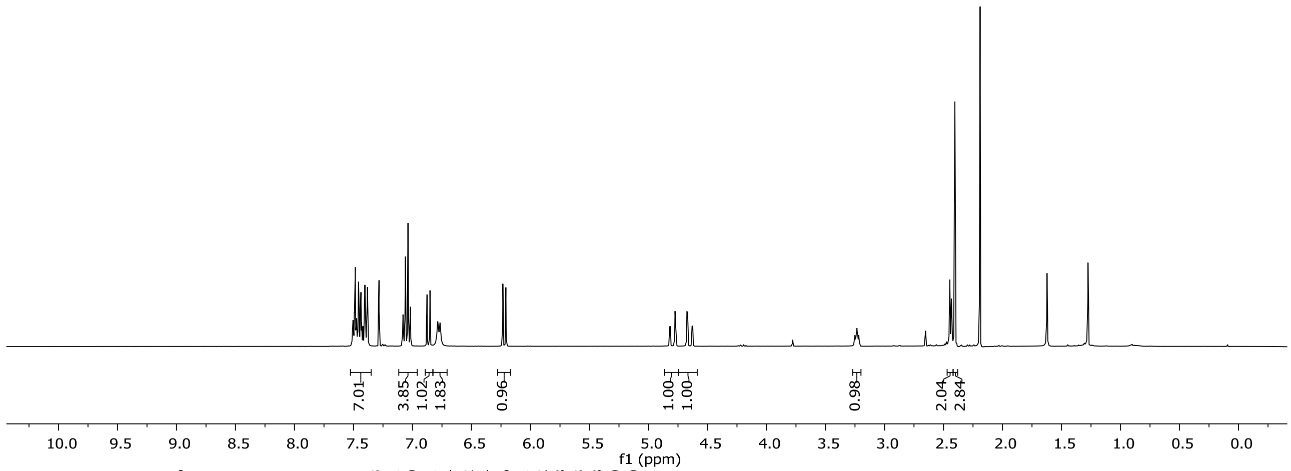
7.01
3.85
1.02
1.83
0.96
1.00
1.00
0.98
2.04
2.84
10.0
9.5
9.0
8.5
8.0
7.5
7.0
6.5
6.0
5.5
5.0
4.5
4.0
3.5
3.0
2.5
2.0
1.5
1.0
0.5
0.0
f1 (ppm)

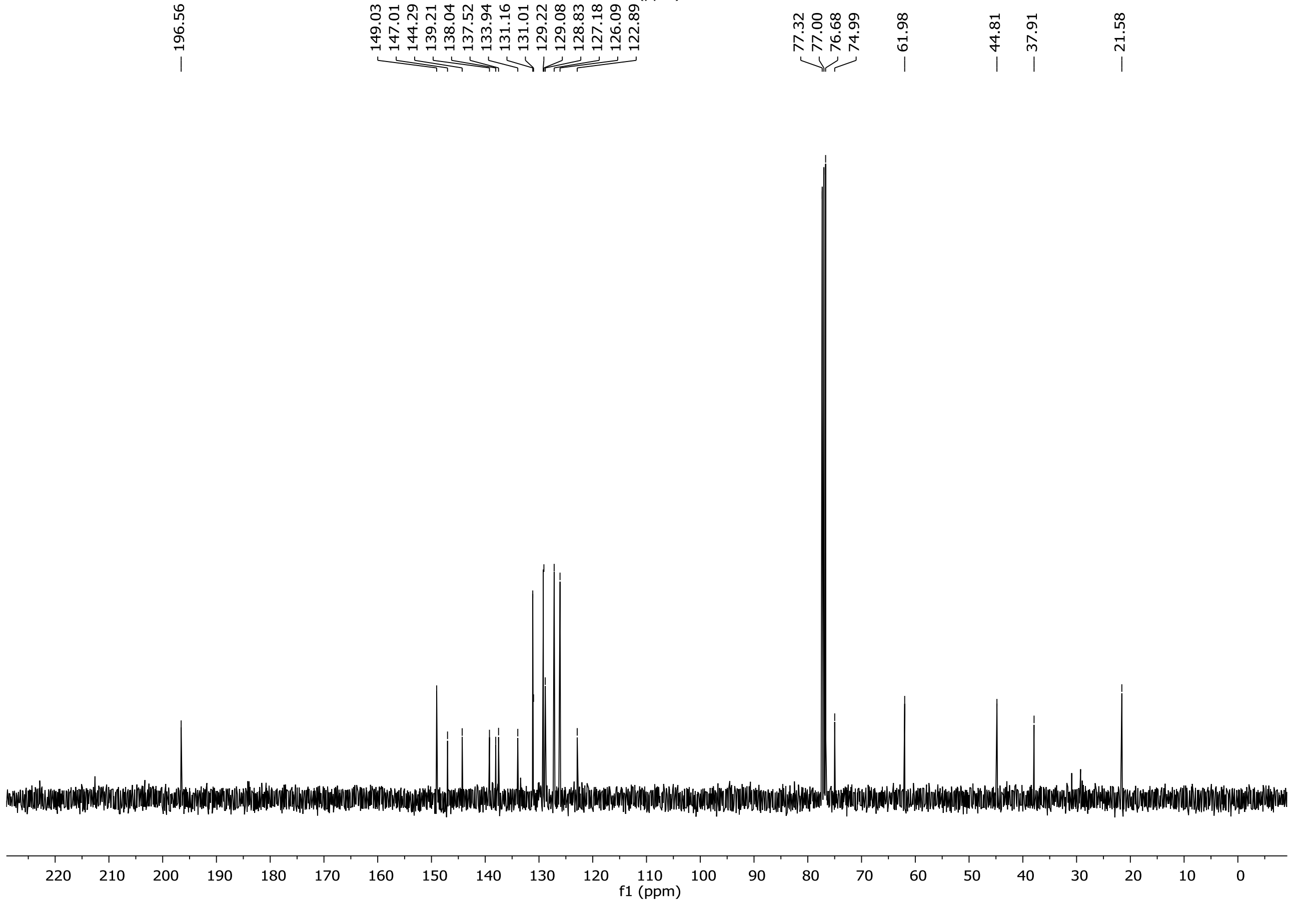
196.56
149.03
147.01
144.29
139.21
138.04
137.52
133.94
131.16
131.01
129.22
129.08
128.83
127.18
126.09
122.89
77.32
77.00
76.68
74.99
61.98
44.81
37.91
21.58
220
210
200
190
180
170
160
150
140
130
120
110
100
90
80
70
60
50
40
30
20
10
0
f1 (ppm)

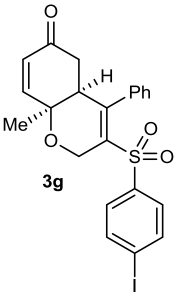
O
H
Ph
Me
O
S
O
O
3g
I

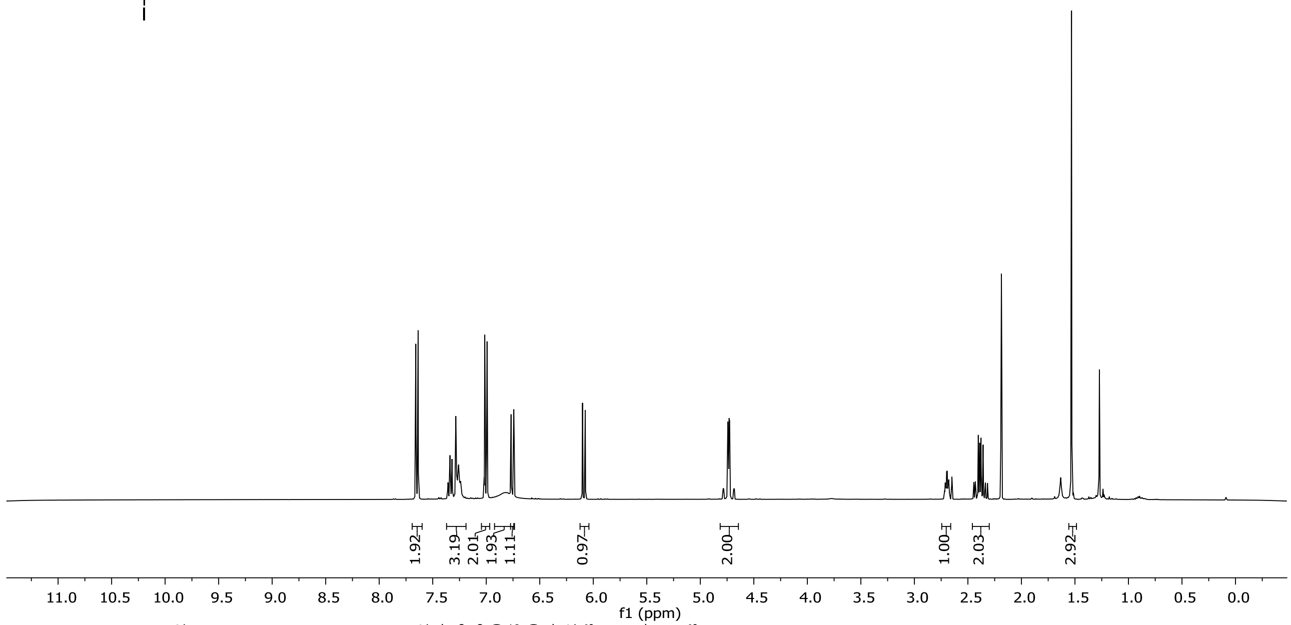
1.92
3.19
2.01
1.93
1.11
0.97
2.00
1.00
2.03
2.92
11.0 10.5 10.0 9.5 9.0 8.5 8.0 7.5 7.0 6.5 6.0 5.5 5.0 4.5 4.0 3.5 3.0 2.5 2.0 1.5 1.0 0.5 0.0
f1 (ppm)

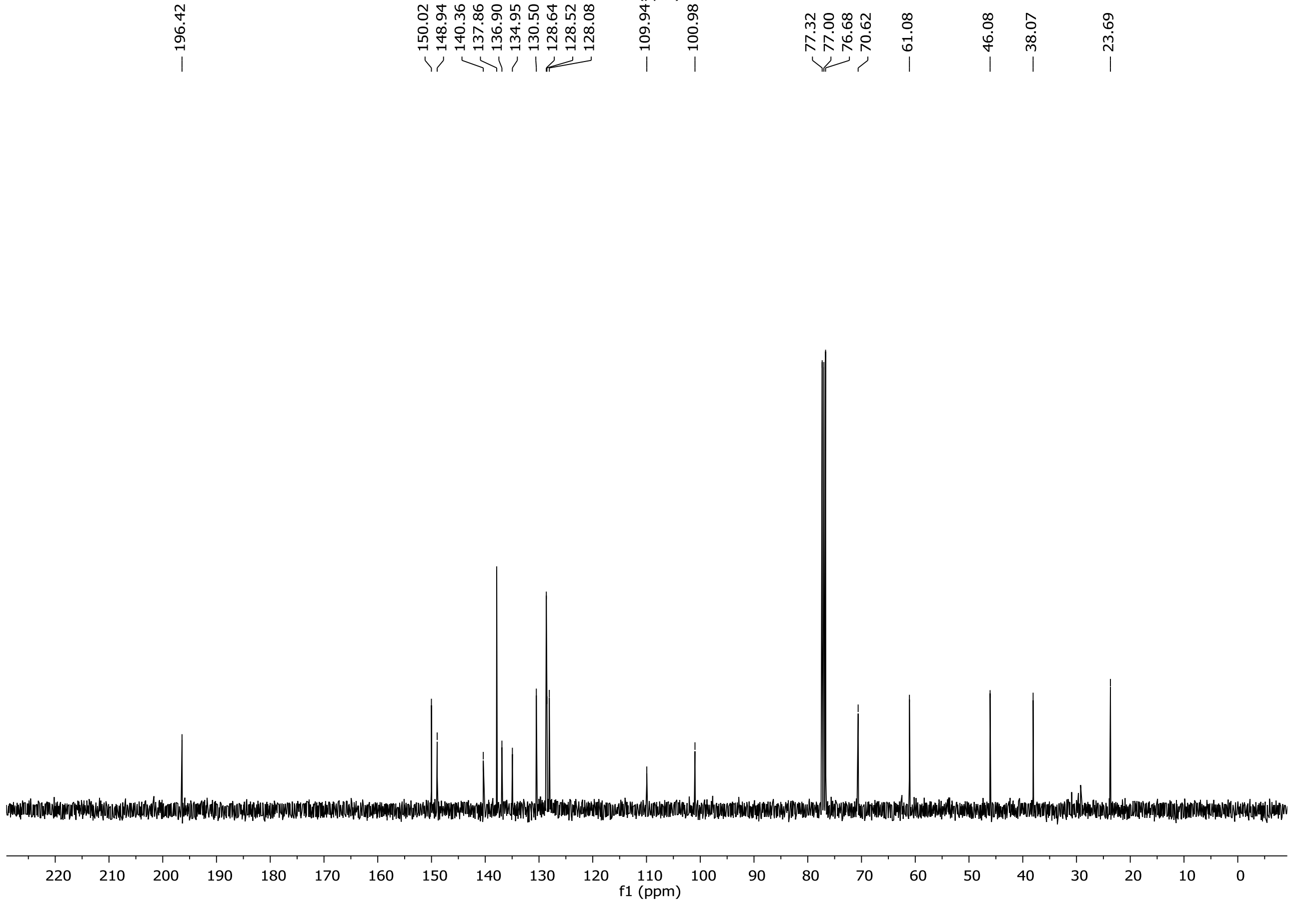
196.42
150.02
148.94
140.36
137.86
136.90
134.95
130.50
128.64
128.52
128.08
109.94
100.98
77.32
77.00
76.68
70.62
61.08
46.08
38.07
23.69
220 210 200 190 180 170 160 150 140 130 120 110 100 90 80 70 60 50 40 30 20 10 0
f1 (ppm)

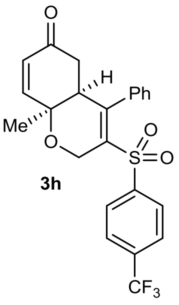
O
H
Ph
Me
O
S
O
O
CF3

3h

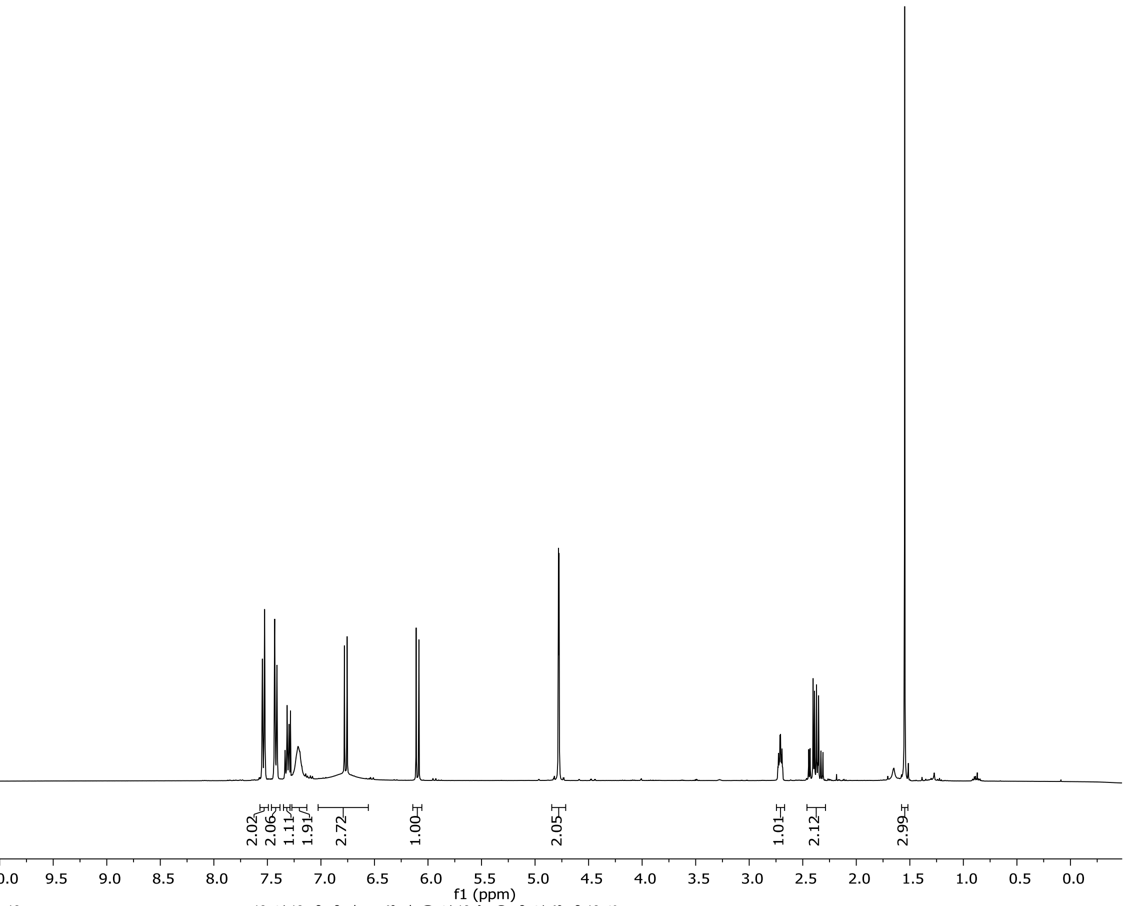
2.02
2.06
1.11
1.91
2.72
1.00
2.05
1.01
2.12
2.99
f1 (ppm)

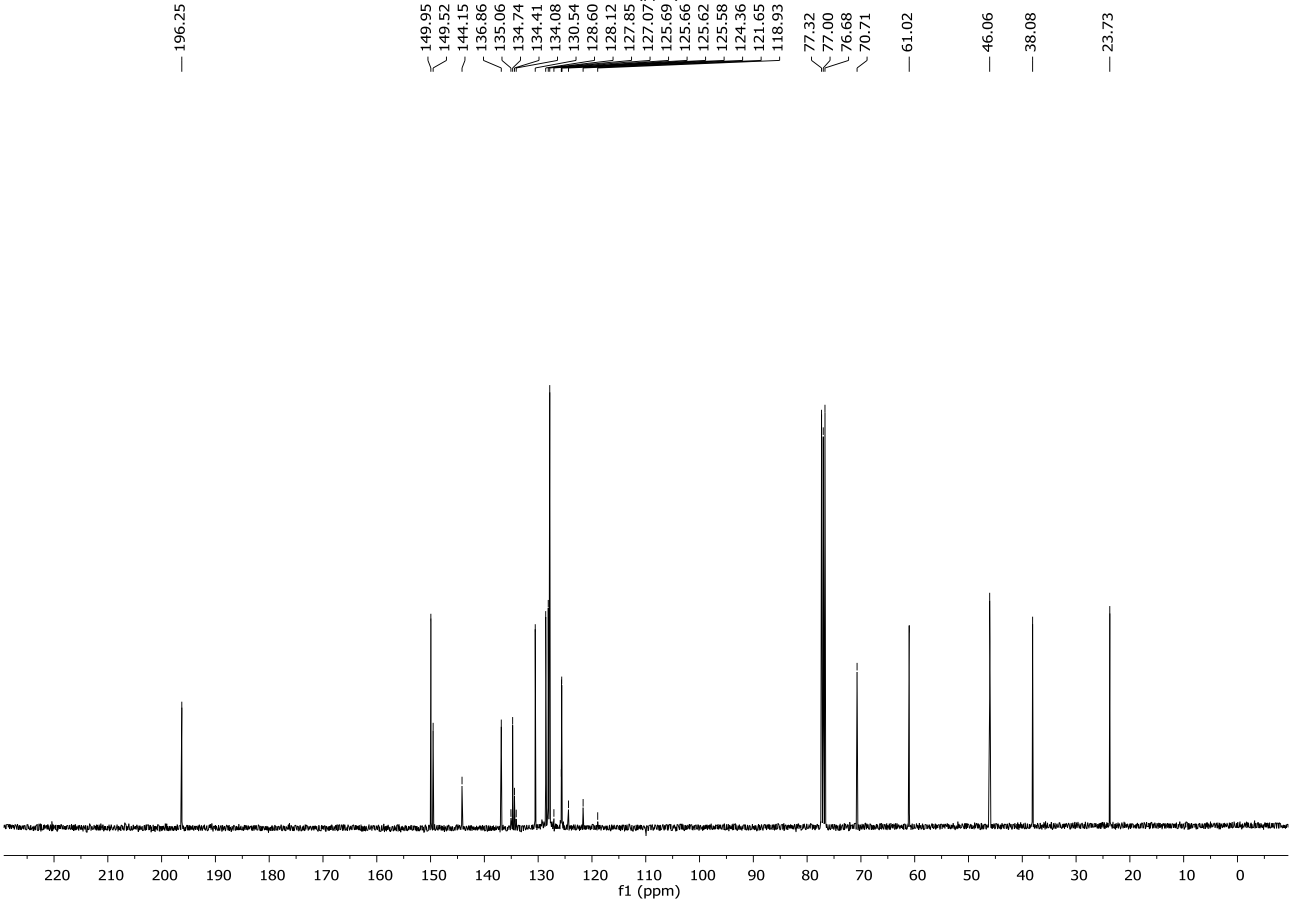
196.25
149.95
149.52
144.15
136.86
135.06
134.74
134.41
134.08
130.54
128.60
128.12
127.85
127.07
125.69
125.66
125.62
125.58
124.36
121.65
118.93
77.32
77.00
76.68
70.71
61.02
46.06
38.08
23.73
f1 (ppm)

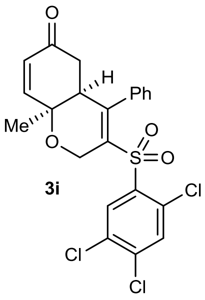

O
H
Ph
Me
O
S
O
O
Cl
Cl
Cl

3i

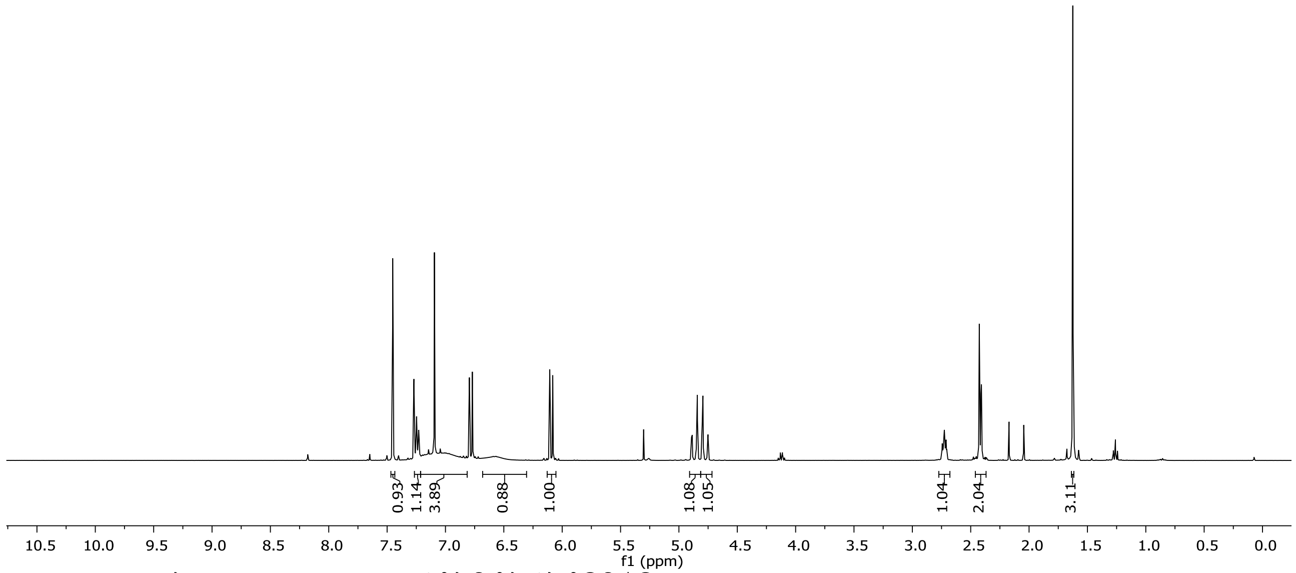

0.93
1.14
3.89
0.88
1.00
1.08
1.05
1.04
2.04
3.11
10.5 10.0 9.5 9.0 8.5 8.0 7.5 7.0 6.5 6.0 5.5 5.0 4.5 4.0 3.5 3.0 2.5 2.0 1.5 1.0 0.5 0.0
f1 (ppm)

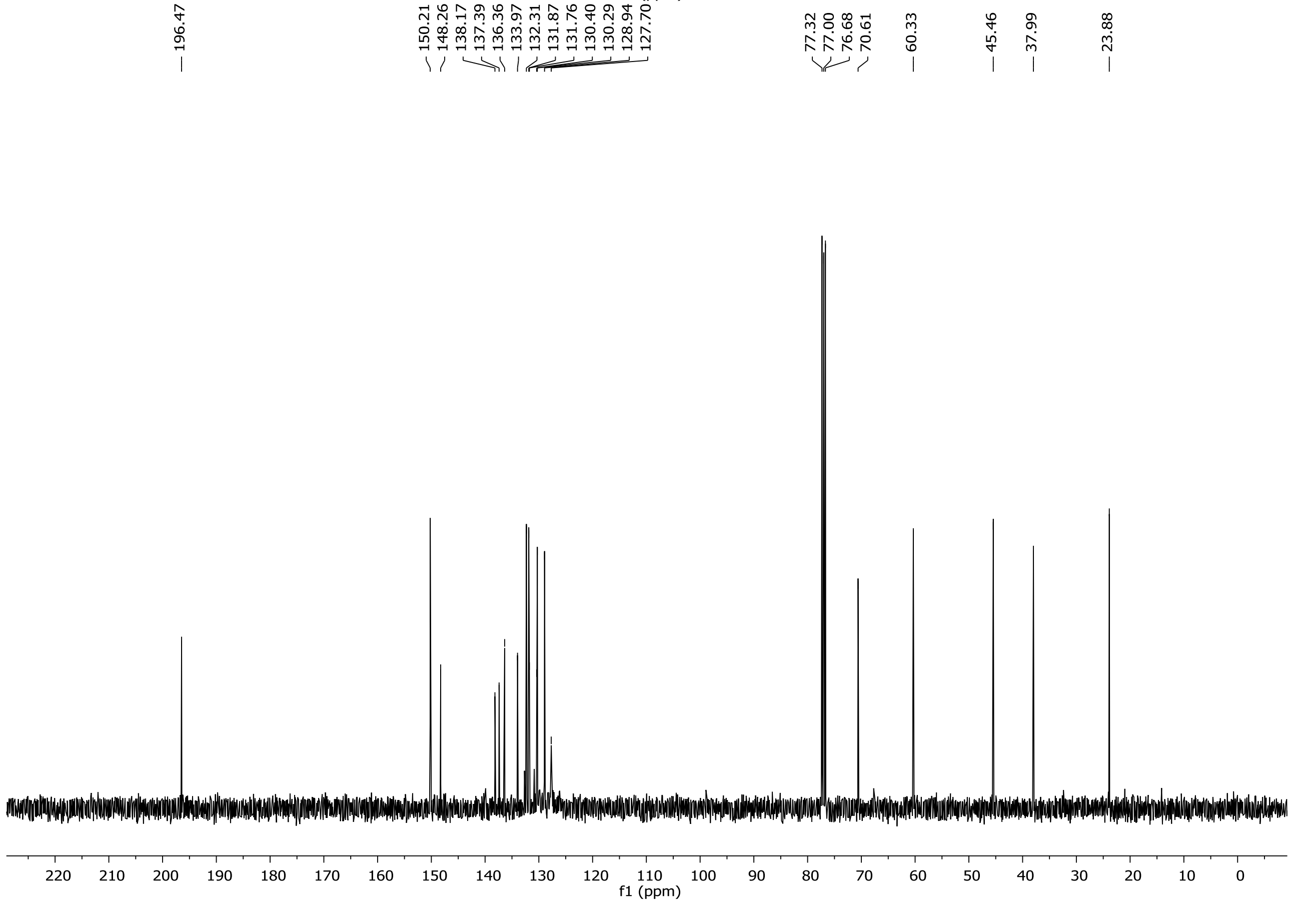

196.47
150.21
148.26
138.17
137.39
136.36
133.97
132.31
131.87
131.76
130.40
130.29
128.94
127.70
77.32
77.00
76.68
70.61
60.33
45.46
37.99
23.88
220 210 200 190 180 170 160 150 140 130 120 110 100 90 80 70 60 50 40 30 20 10 0
f1 (ppm)

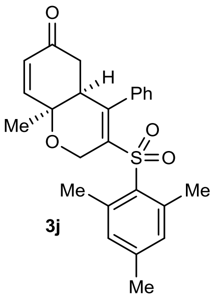
O
H
Ph
Me
O
S
O
O
Me
Me
Me

3j

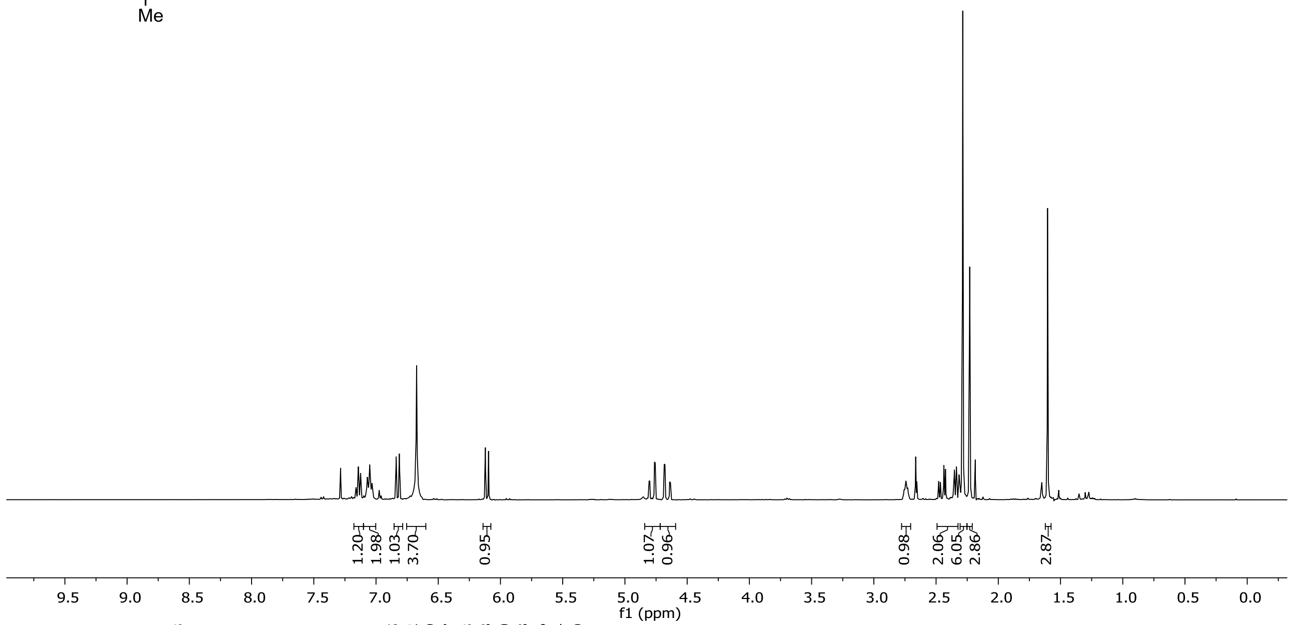
1.20
1.98
1.03
3.70
0.95
1.07
0.96
0.98
2.06
6.05
2.86
2.87
9.5 9.0 8.5 8.0 7.5 7.0 6.5 6.0 5.5 5.0 4.5 4.0 3.5 3.0 2.5 2.0 1.5 1.0 0.5 0.0
f1 (ppm)

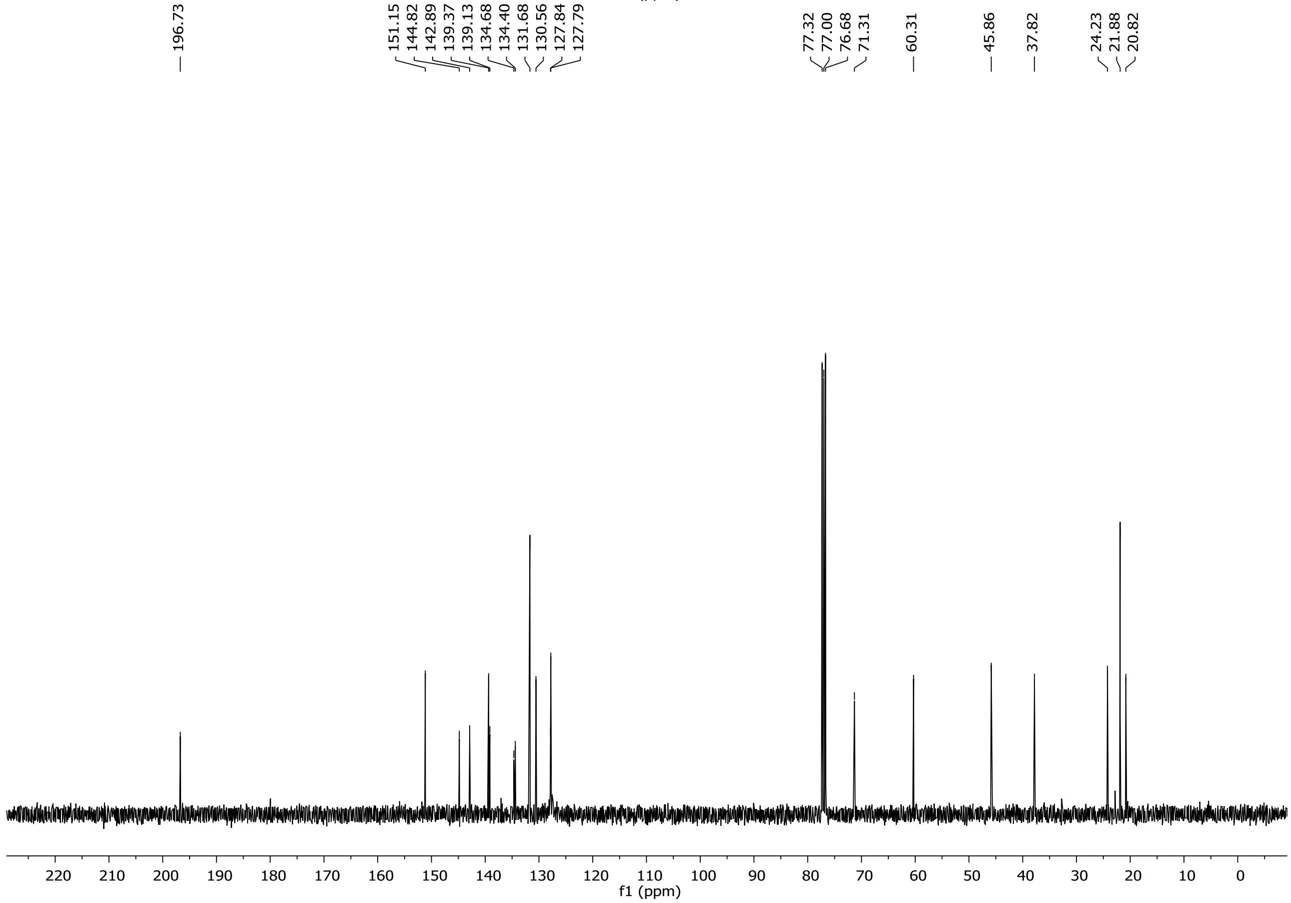
196.73
151.15
144.82
142.89
139.37
139.13
134.68
134.40
131.68
130.56
127.84
127.79
77.32
77.00
76.68
71.31
60.31
45.86
37.82
24.23
21.88
20.82
220 210 200 190 180 170 160 150 140 130 120 110 100 90 80 70 60 50 40 30 20 10 0
f1 (ppm)

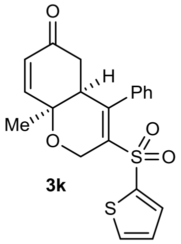
O
H
Ph
Me
O
S
O
O
S

3k

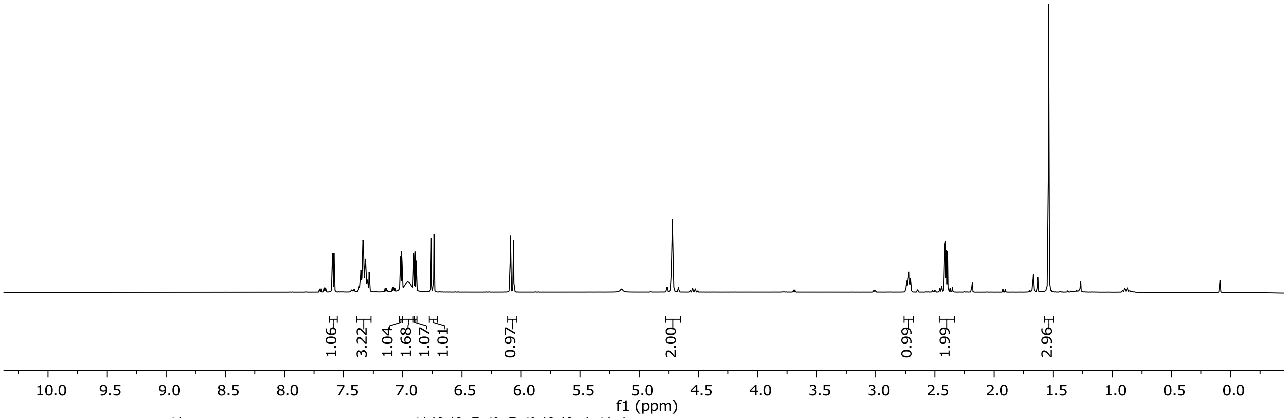
1.06
3.22
1.04
1.68
1.07
1.01
0.97
2.00
0.99
1.99
2.96
10.0 9.5 9.0 8.5 8.0 7.5 7.0 6.5 6.0 5.5 5.0 4.5 4.0 3.5 3.0 2.5 2.0 1.5 1.0 0.5 0.0
f1 (ppm)

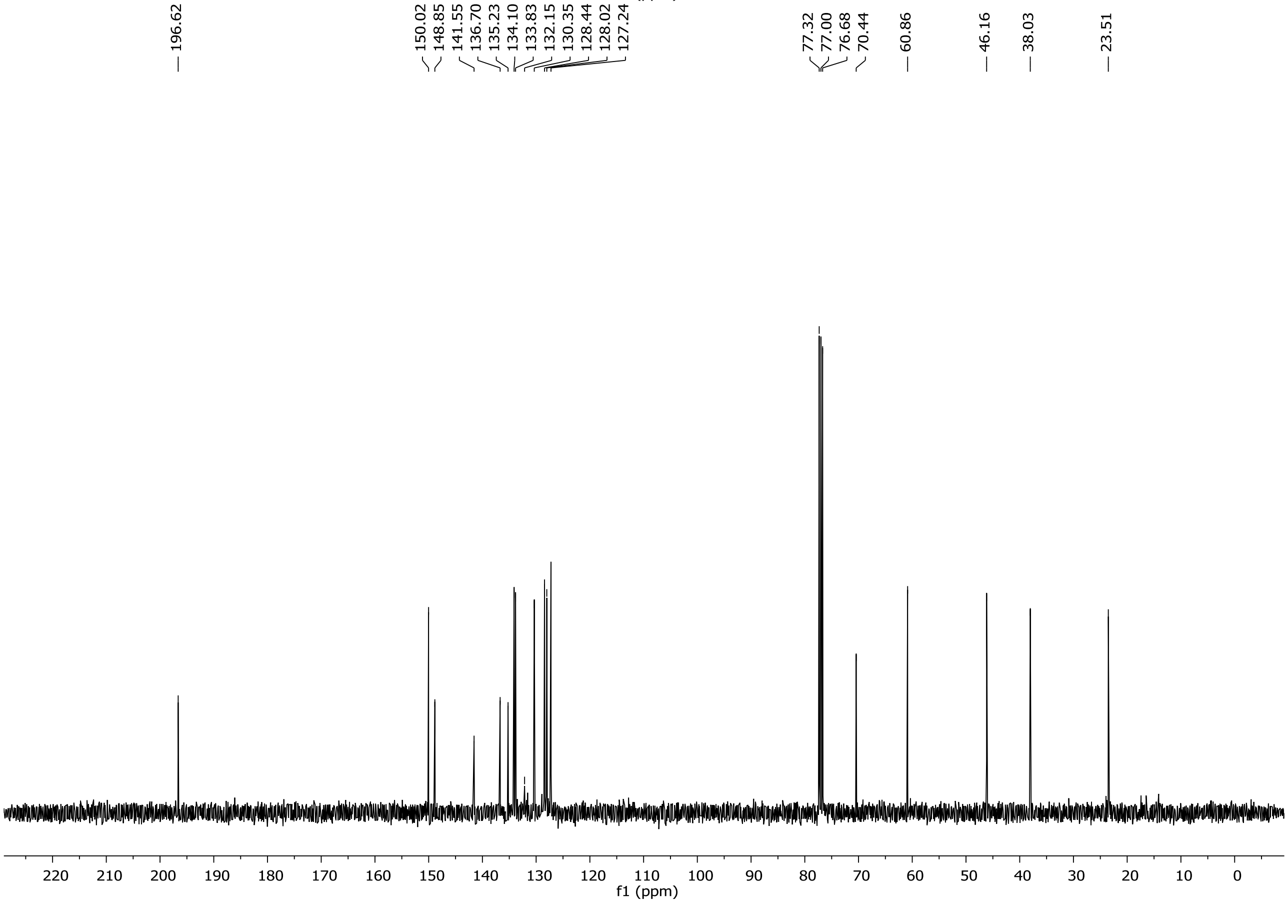
196.62
150.02
148.85
141.55
136.70
135.23
134.10
133.83
132.15
130.35
128.44
128.02
127.24
77.32
77.00
76.68
70.44
60.86
46.16
38.03
23.51
220 210 200 190 180 170 160 150 140 130 120 110 100 90 80 70 60 50 40 30 20 10 0
f1 (ppm)

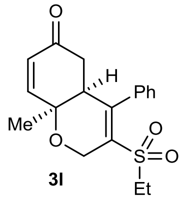
O
H
Ph
Me
O
O
S
O
3l
Et

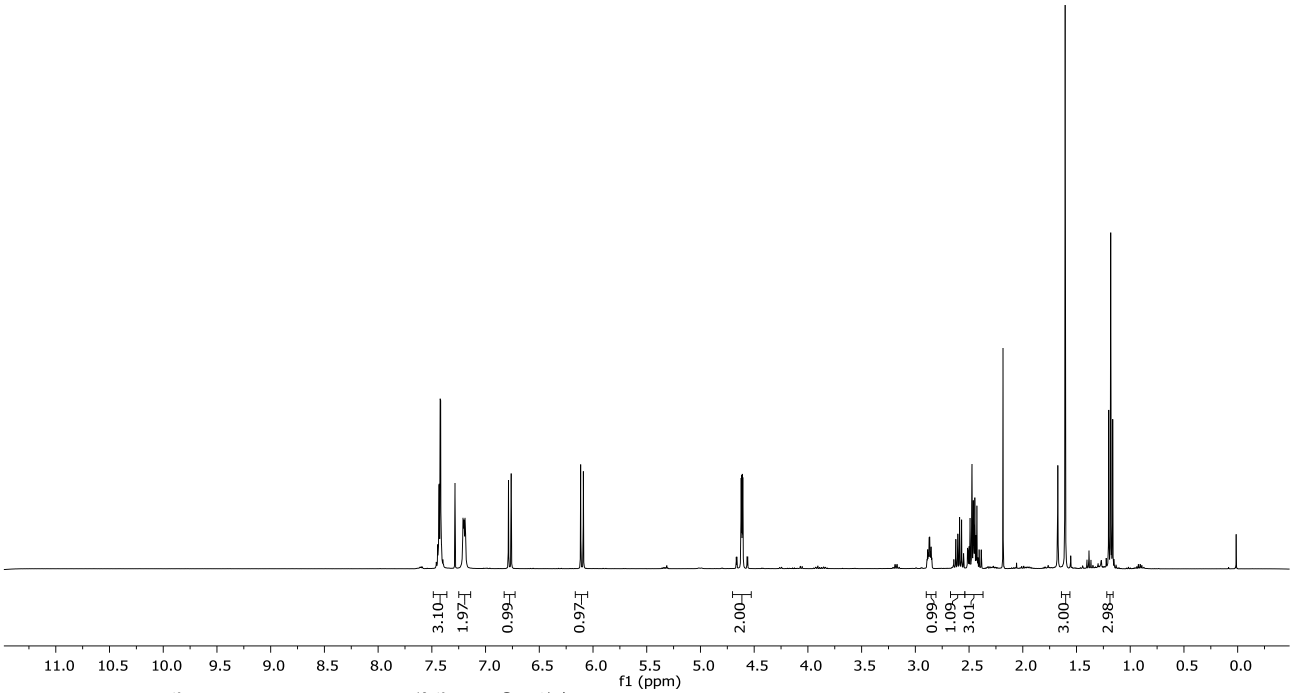
3.10
1.97
0.99
0.97
2.00
0.99
1.09
3.01
3.00
2.98
11.0 10.5 10.0 9.5 9.0 8.5 8.0 7.5 7.0 6.5 6.0 5.5 5.0 4.5 4.0 3.5 3.0 2.5 2.0 1.5 1.0 0.5 0.0
f1 (ppm)

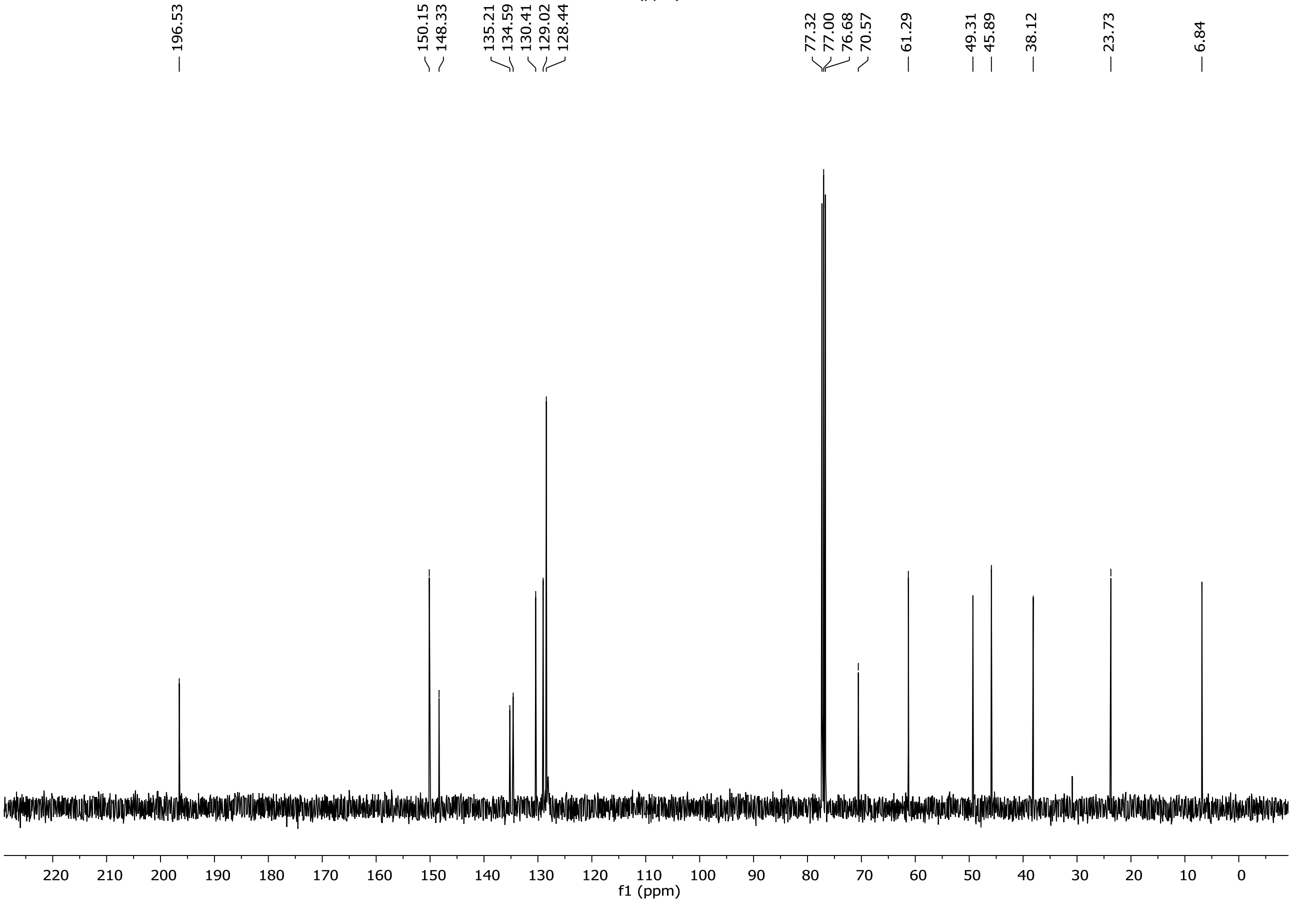
196.53
150.15
148.33
135.21
134.59
130.41
129.02
128.44
77.32
77.00
76.68
70.57
61.29
49.31
45.89
38.12
23.73
6.84
220 210 200 190 180 170 160 150 140 130 120 110 100 90 80 70 60 50 40 30 20 10 0
f1 (ppm)

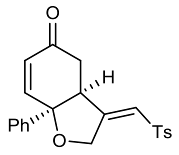
O
H
Ph
O
Ts

6

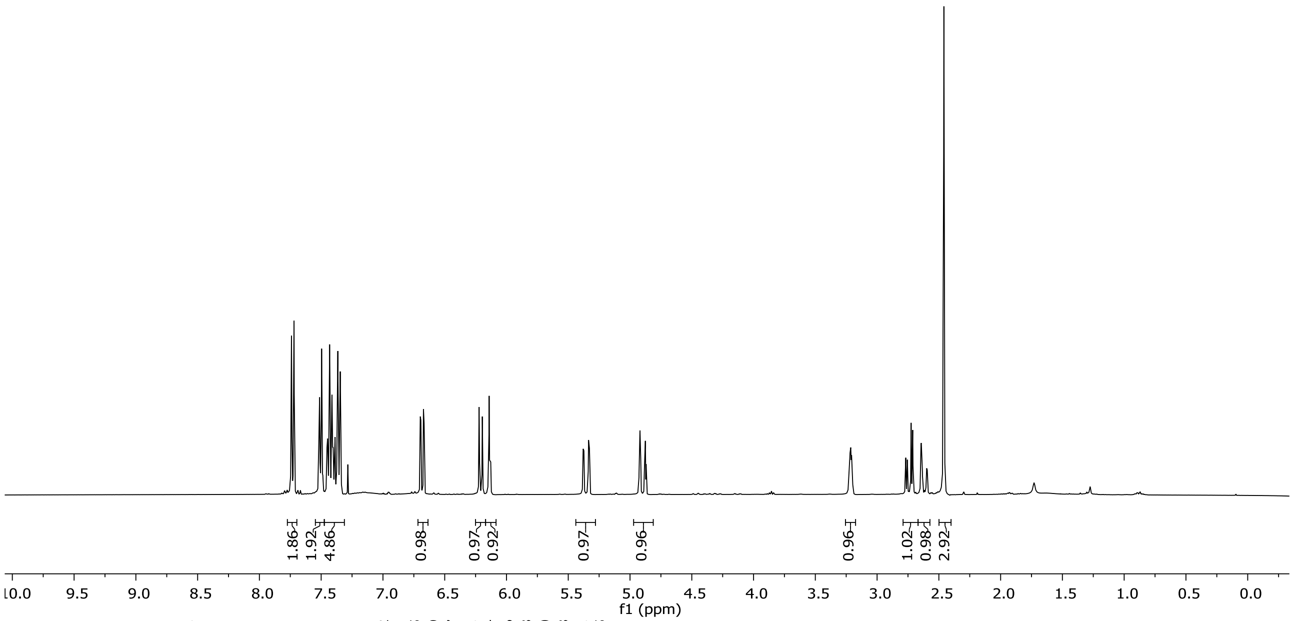
1.86
1.92
4.86
0.98
0.97
0.92
0.97
0.96
0.96
1.02
0.98
2.92
10.0 9.5 9.0 8.5 8.0 7.5 7.0 6.5 6.0 5.5 5.0 4.5 4.0 3.5 3.0 2.5 2.0 1.5 1.0 0.5 0.0
f1 (ppm)

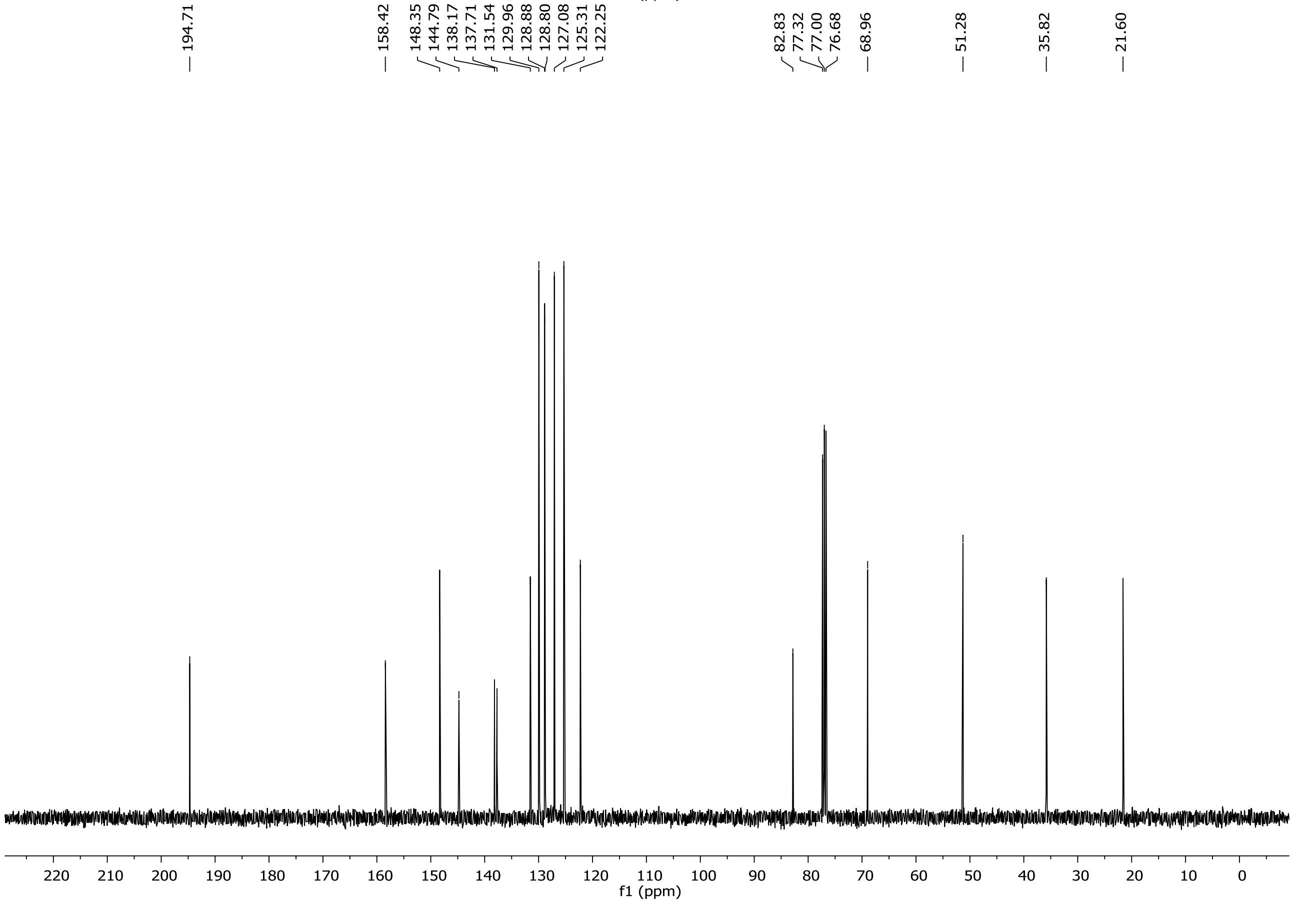
194.71
158.42
148.35
144.79
138.17
137.71
131.54
129.96
128.88
128.80
127.08
125.31
122.25
82.83
77.32
77.00
76.68
68.96
51.28
35.82
21.60
220 210 200 190 180 170 160 150 140 130 120 110 100 90 80 70 60 50 40 30 20 10 0
f1 (ppm)

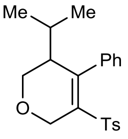

Me
Me
Ph
O
Ts

9a

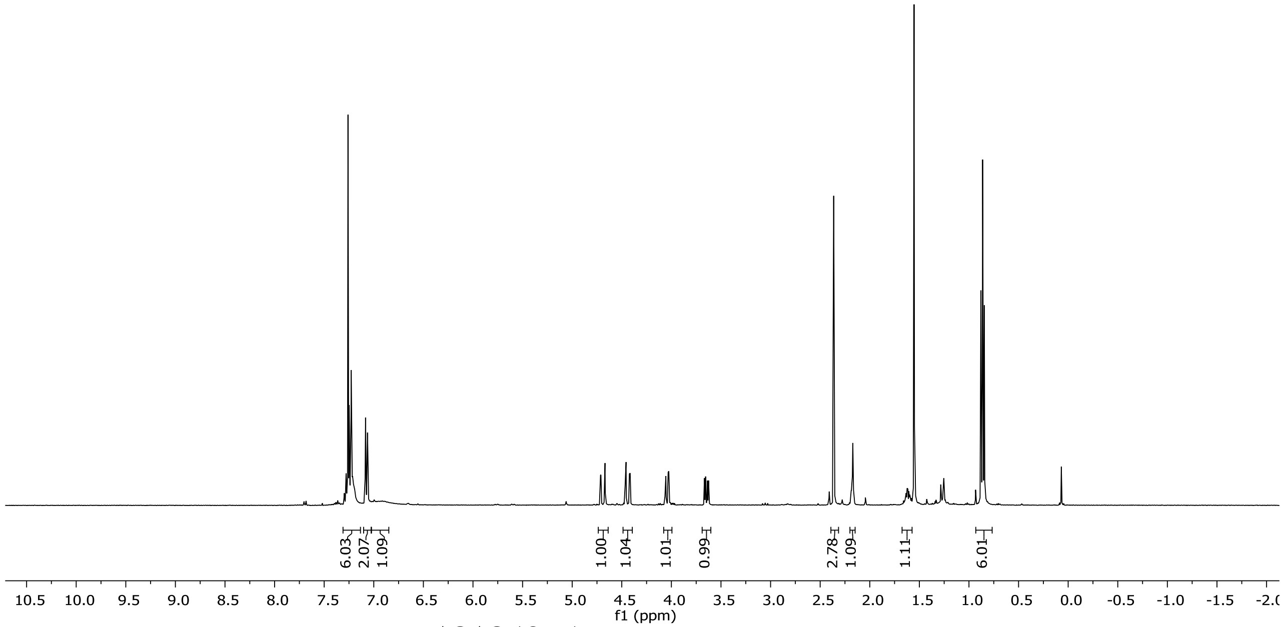

6.03
2.07
1.09
1.00
1.04
1.01
0.99
2.78
1.09
1.11
6.01
10.5 10.0 9.5 9.0 8.5 8.0 7.5 7.0 6.5 6.0 5.5 5.0 4.5 4.0 3.5 3.0 2.5 2.0 1.5 1.0 0.5 0.0 -0.5 -1.0 -1.5
f1 (ppm)

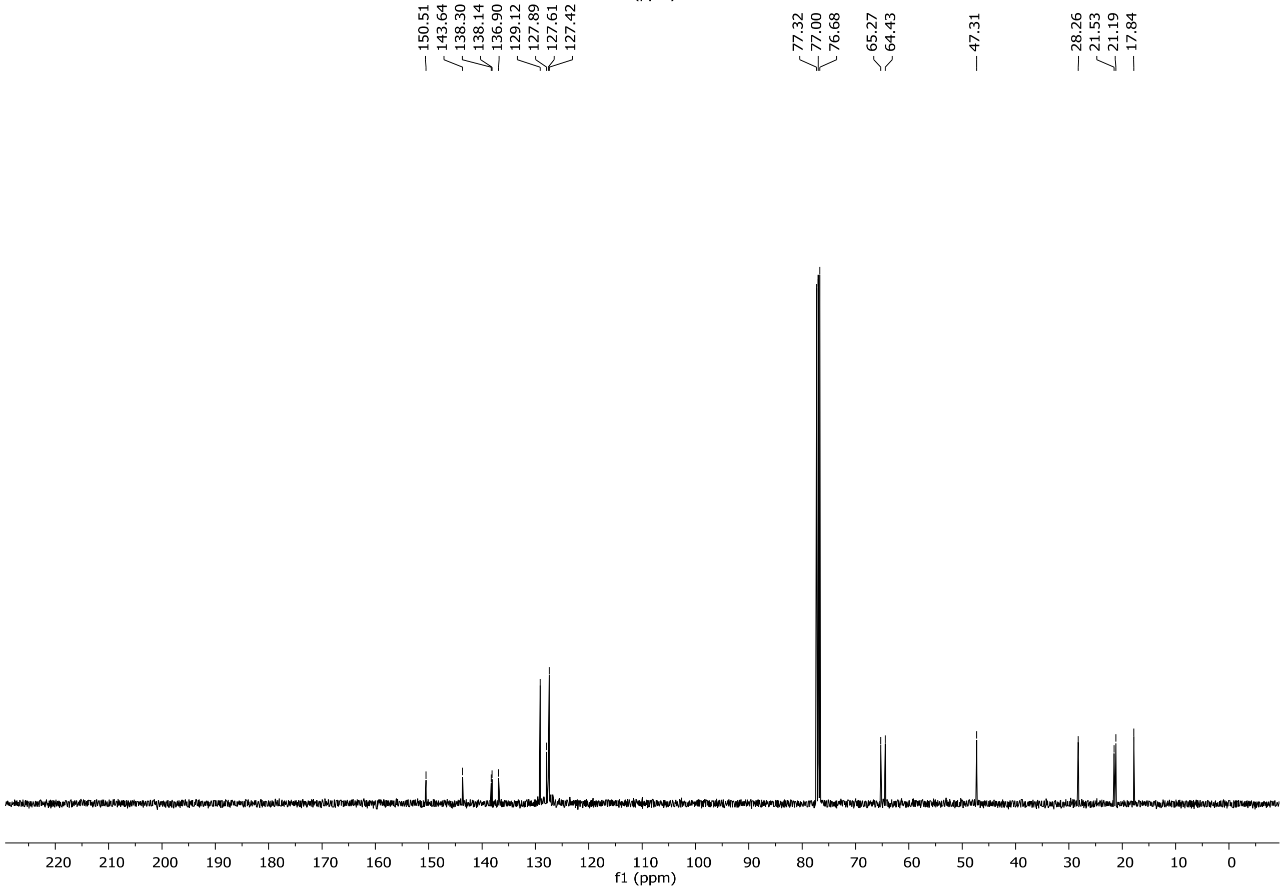

150.51
143.64
138.30
138.14
136.90
129.12
127.89
127.61
127.42
77.32
77.00
76.68
65.27
64.43
47.31
28.26
21.53
21.19
17.84
220 210 200 190 180 170 160 150 140 130 120 110 100 90 80 70 60 50 40 30 20 10 0
f1 (ppm)

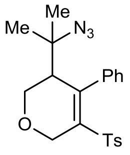
Me
Me
N3
Ph
O
Ts

8a

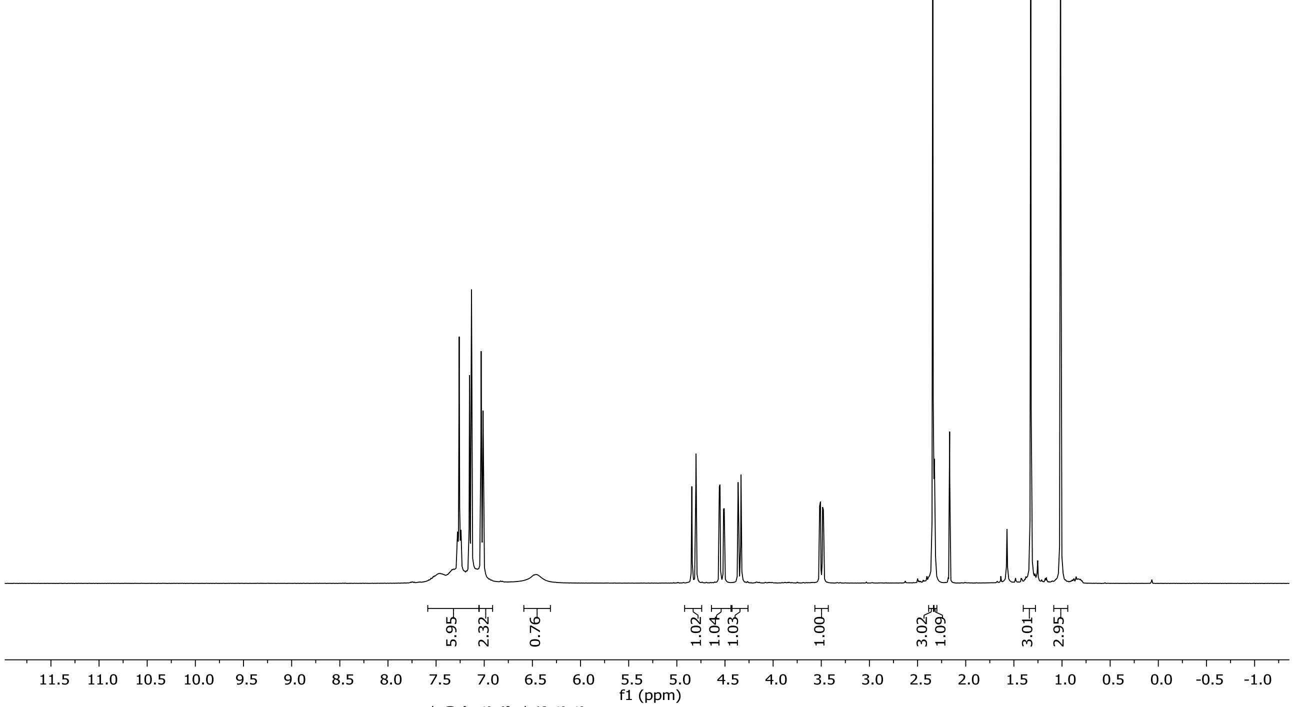
5.95
2.32
0.76
1.02
1.04
1.03
1.00
3.02
1.09
3.01
2.95
f1 (ppm)

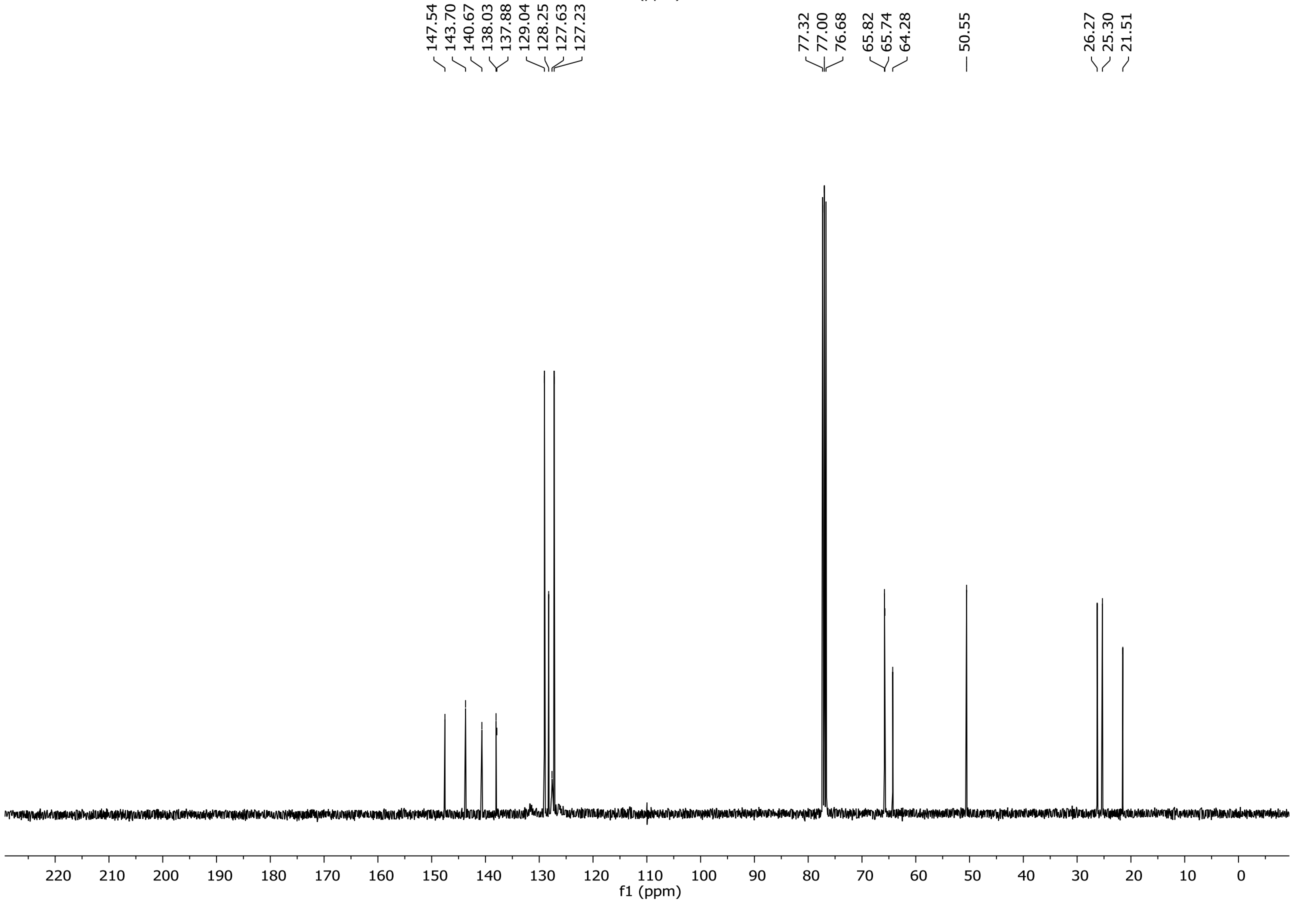
147.54
143.70
140.67
138.03
137.88
129.04
128.25
127.63
127.23
77.32
77.00
76.68
65.82
65.74
64.28
50.55
26.27
25.30
21.51
f1 (ppm)

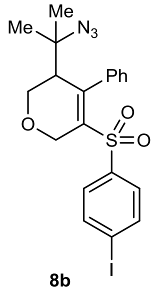
Me
Me
N3
Ph
O
S
O
O
I

8b

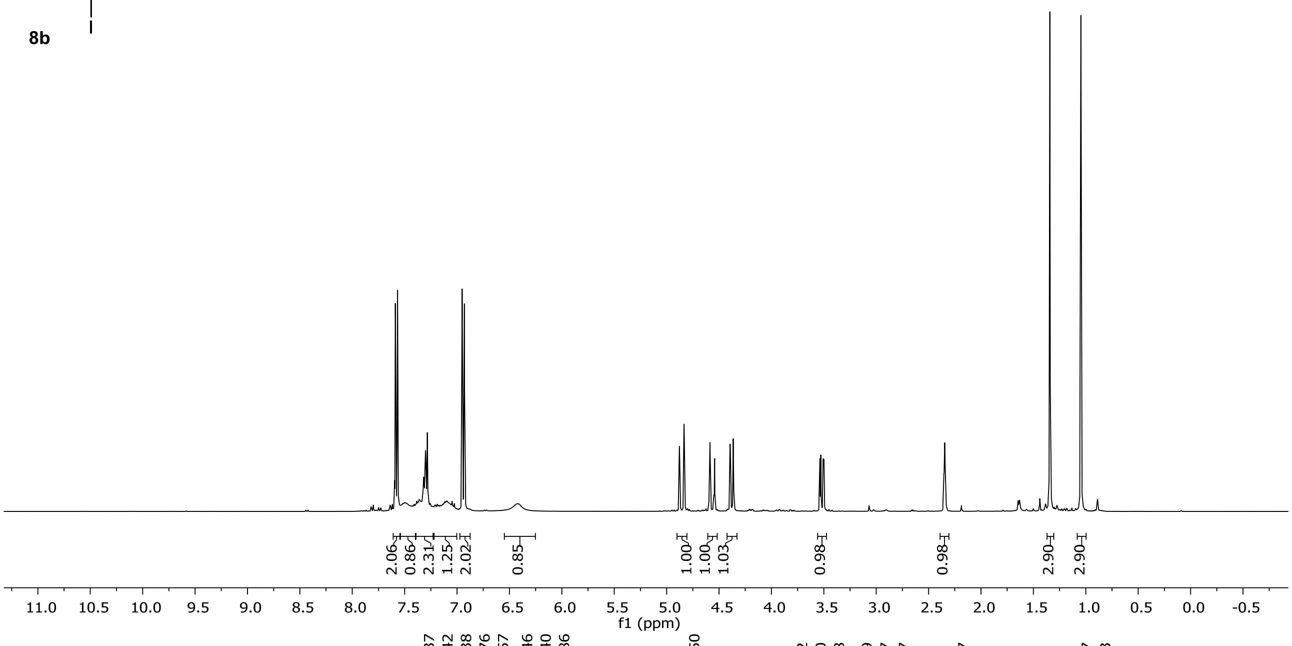
2.06
0.86
2.31
1.25
2.02
0.85
1.00
1.00
1.03
0.98
0.98
2.90
2.90
11.0 10.5 10.0 9.5 9.0 8.5 8.0 7.5 7.0 6.5 6.0 5.5 5.0 4.5 4.0 3.5 3.0 2.5 2.0 1.5 1.0 0.5 0.0 -0.5
f1 (ppm)

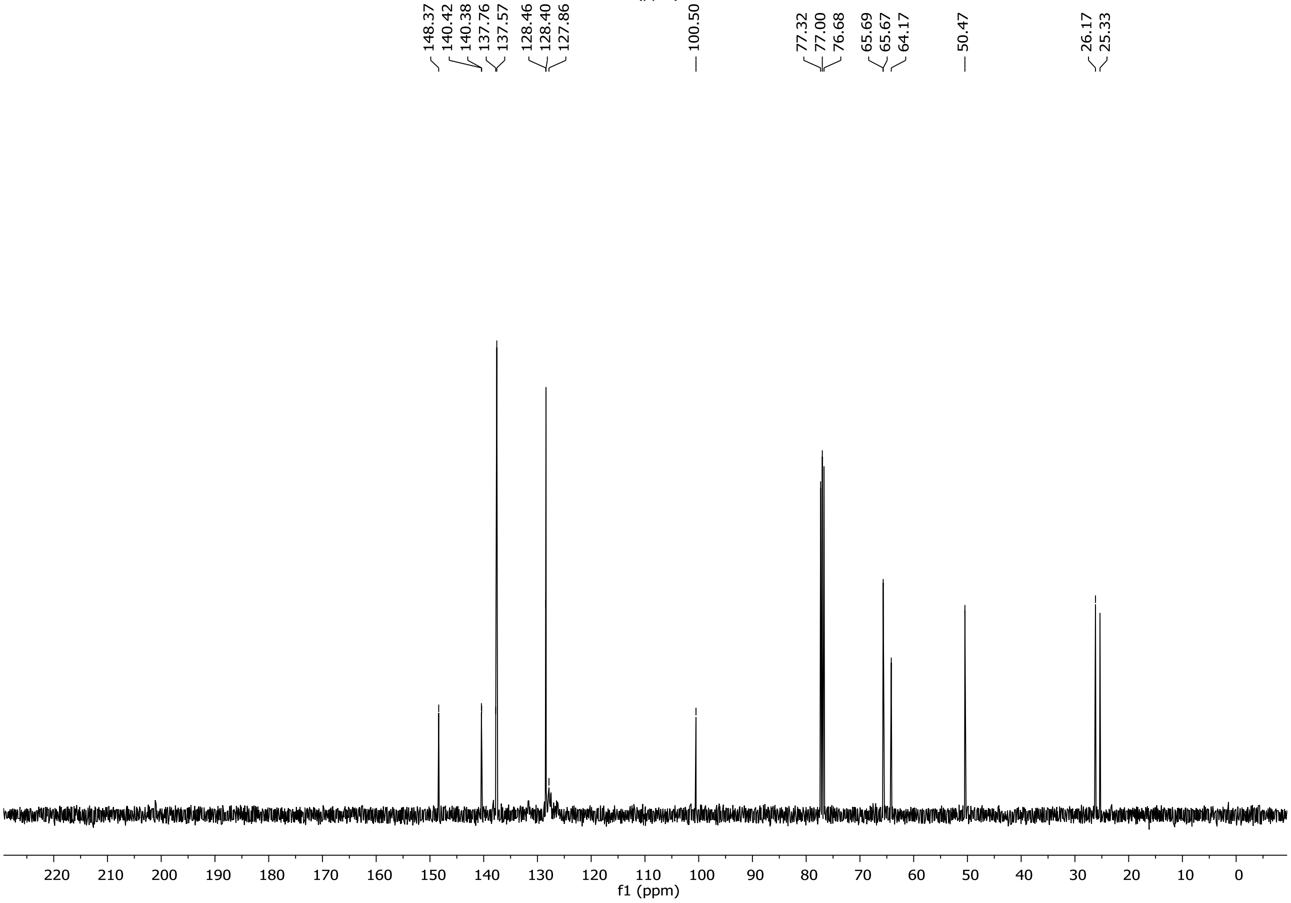
148.37
140.42
140.38
137.76
137.57
128.46
128.40
127.86
100.50
77.32
77.00
76.68
65.69
65.67
64.17
50.47
26.17
25.33
220 210 200 190 180 170 160 150 140 130 120 110 100 90 80 70 60 50 40 30 20 10 0
f1 (ppm)

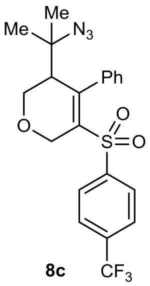
Me
Me
N3
Ph
O
O
S
O
CF3

8c

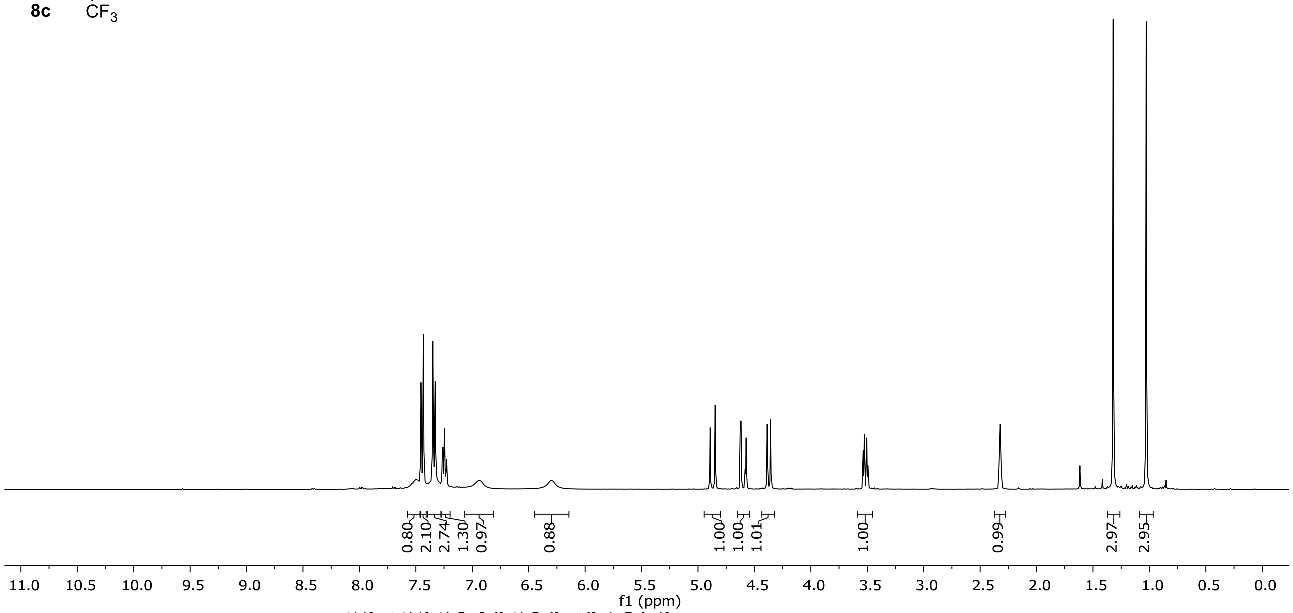
0.80
2.10
2.74
1.30
0.97
0.88
1.00
1.00
1.01
1.00
0.99
2.97
2.95
11.0 10.5 10.0 9.5 9.0 8.5 8.0 7.5 7.0 6.5 6.0 5.5 5.0 4.5 4.0 3.5 3.0 2.5 2.0 1.5 1.0 0.5 0.0
f1 (ppm)

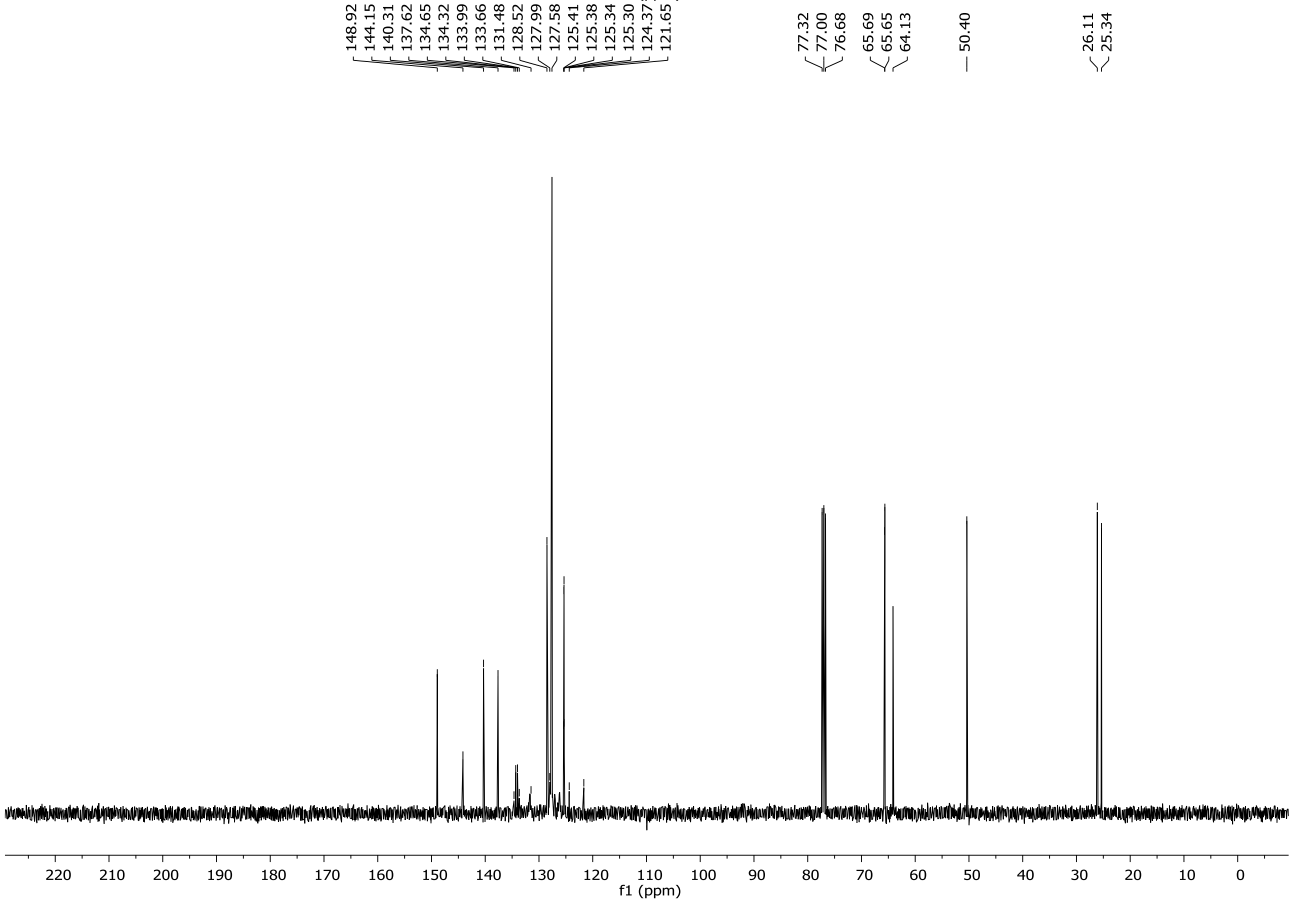
148.92
144.15
140.31
137.62
134.65
134.32
133.99
133.66
131.48
128.52
127.99
127.58
125.41
125.38
125.34
125.30
124.37
121.65
77.32
77.00
76.68
65.69
65.65
64.13
50.40
26.11
25.34
220 210 200 190 180 170 160 150 140 130 120 110 100 90 80 70 60 50 40 30 20 10 0
f1 (ppm)

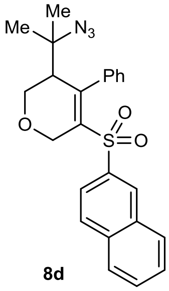
Me
Me
N3
Ph
O
O
S
O

8d

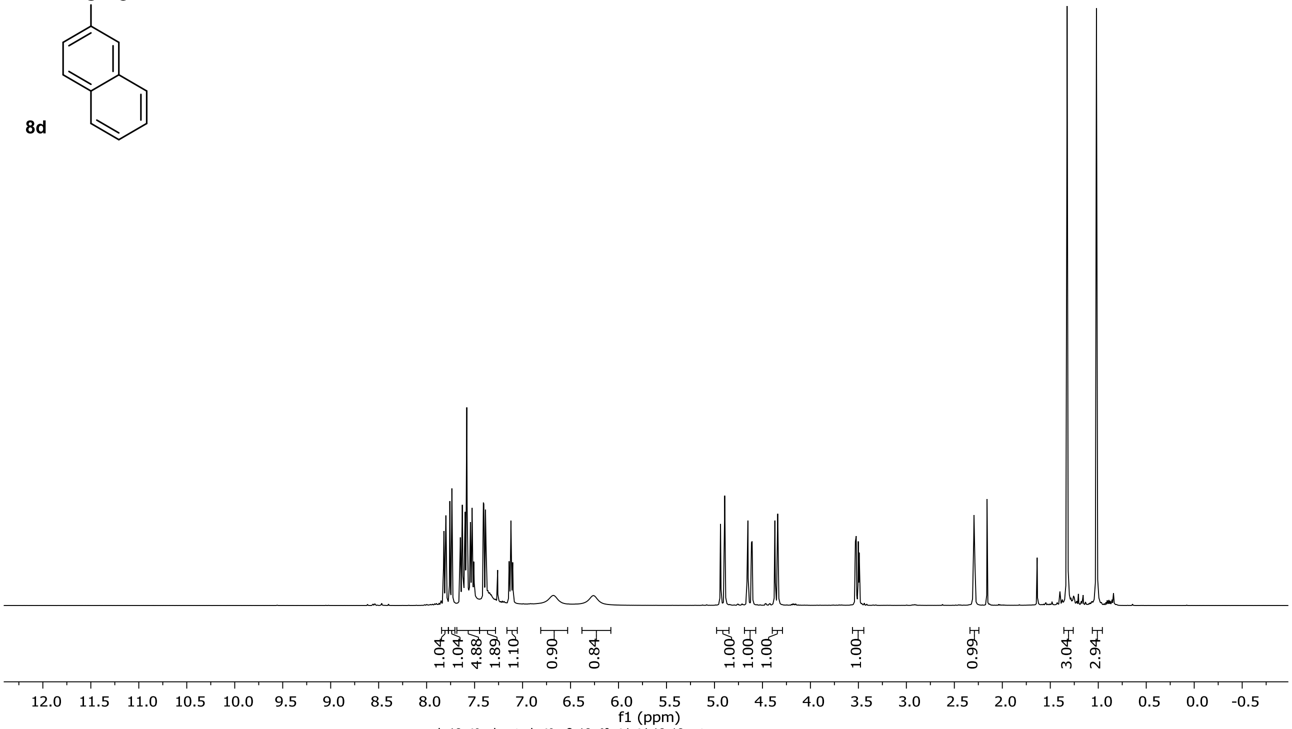
1.04
1.04
4.88
1.89
1.10
0.90
0.84
1.00
1.00
1.00
1.00
0.99
3.04
2.94
12.0 11.5 11.0 10.5 10.0 9.5 9.0 8.5 8.0 7.5 7.0 6.5 6.0 5.5 5.0 4.5 4.0 3.5 3.0 2.5 2.0 1.5 1.0 0.5 0.0 -0.5
f1 (ppm)

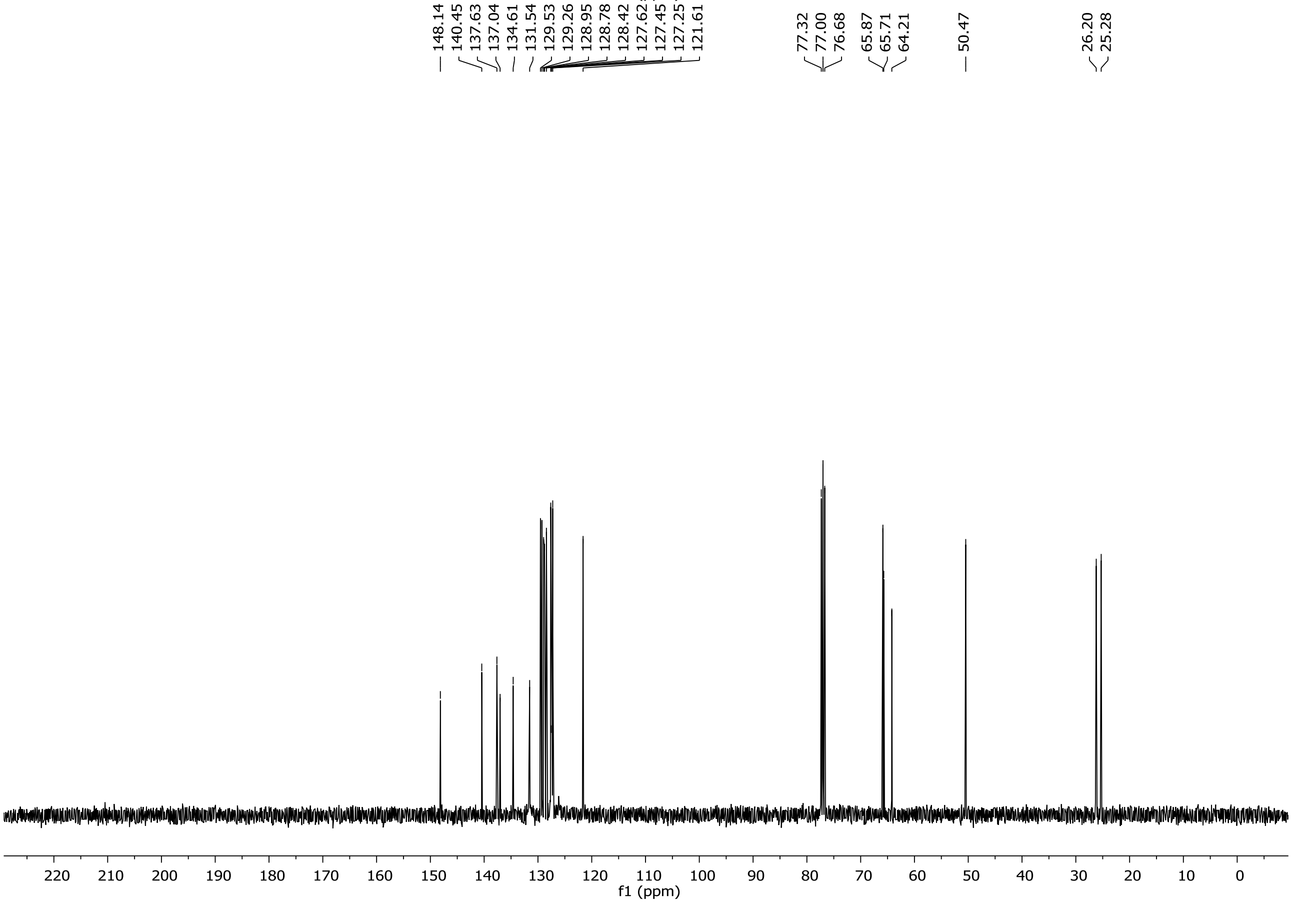
148.14
140.45
137.63
137.04
134.61
131.54
129.53
129.26
128.95
128.78
128.42
127.62
127.45
127.25
121.61
77.32
77.00
76.68
65.87
65.71
64.21
50.47
26.20
25.28
220 210 200 190 180 170 160 150 140 130 120 110 100 90 80 70 60 50 40 30 20 10 0
f1 (ppm)

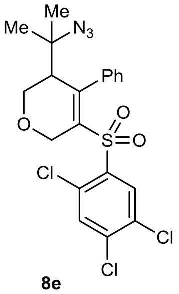

8e

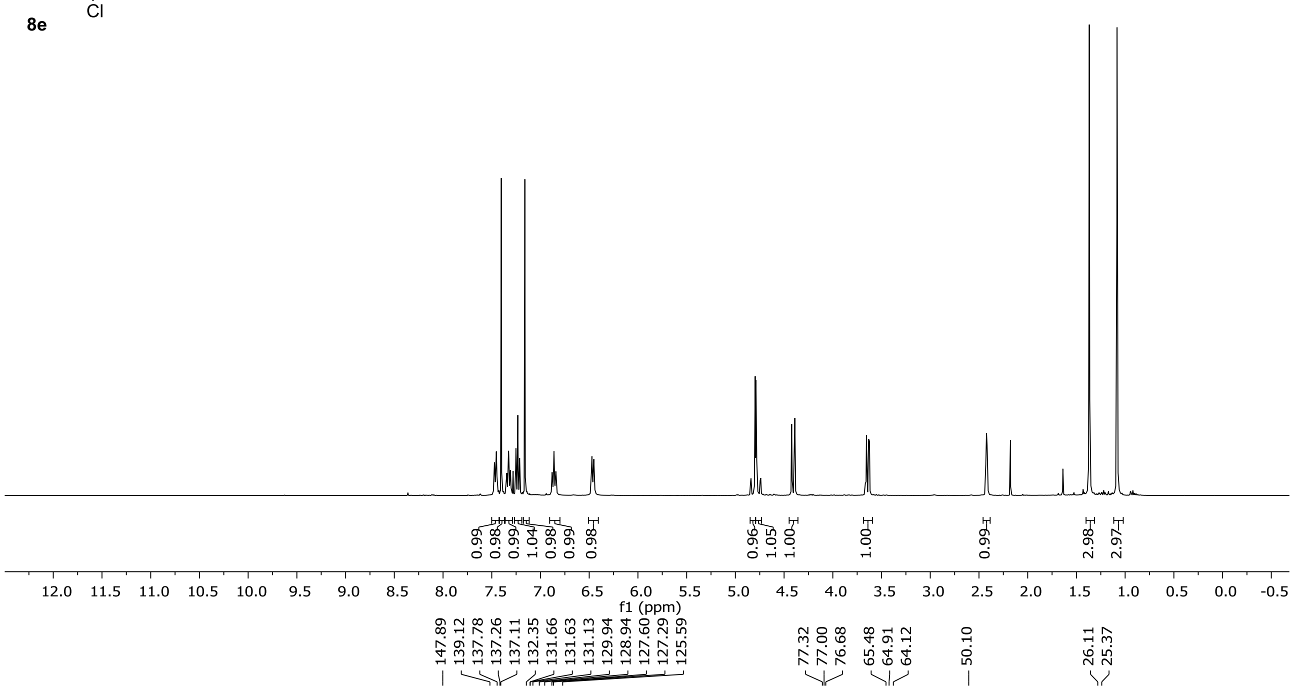

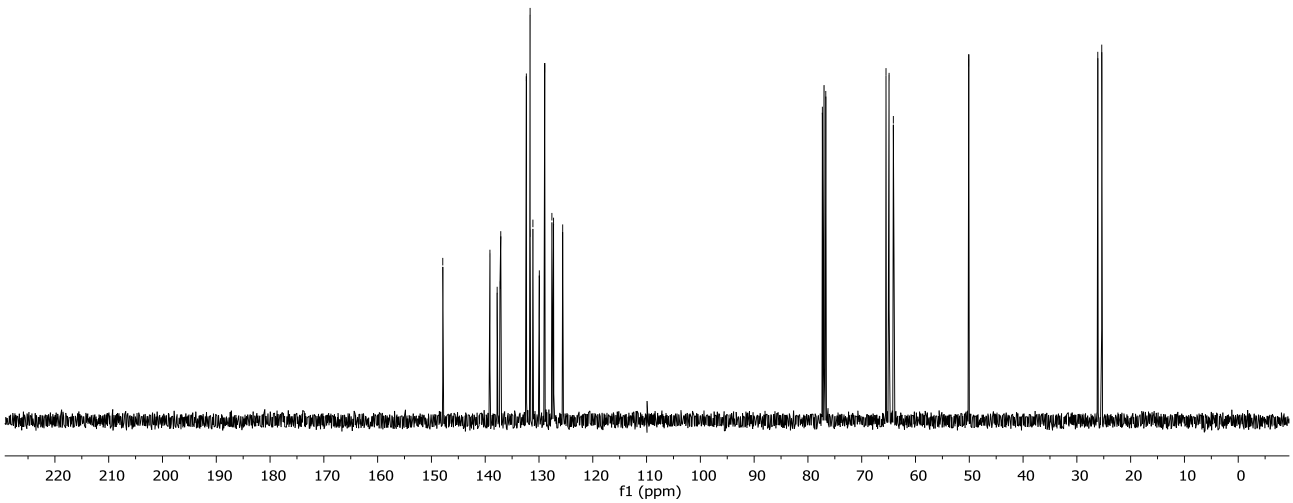

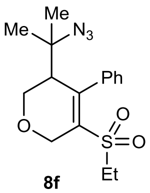
Me
Me
N3
Ph
O
O
S
O
Et

8f

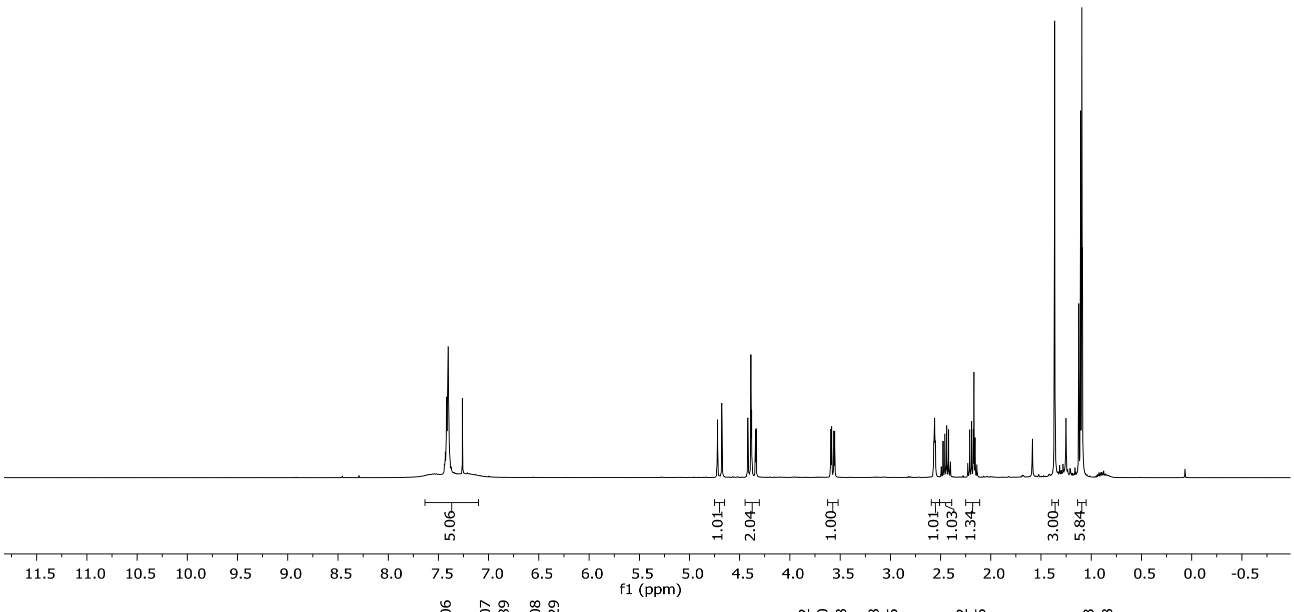
5.06
1.01
2.04
1.00
1.01
1.03
1.34
3.00
5.84
11.5 11.0 10.5 10.0 9.5 9.0 8.5 8.0 7.5 7.0 6.5 6.0 5.5 5.0 4.5 4.0 3.5 3.0 2.5 2.0 1.5 1.0 0.5 0.0 -0.5
f1 (ppm)

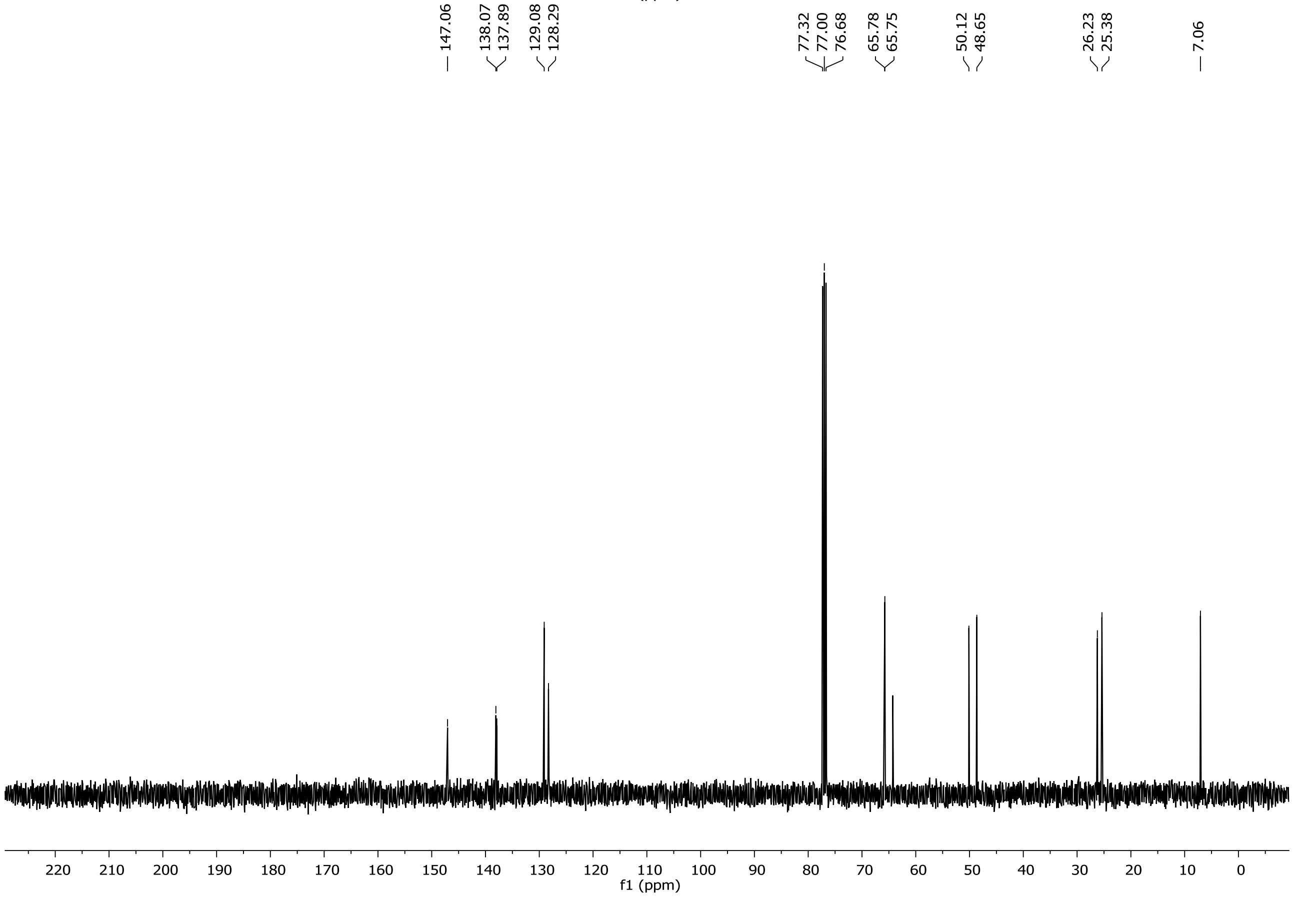
147.06
138.07
137.89
129.08
128.29
77.32
77.00
76.68
65.78
65.75
50.12
48.65
26.23
25.38
7.06
220 210 200 190 180 170 160 150 140 130 120 110 100 90 80 70 60 50 40 30 20 10 0
f1 (ppm)

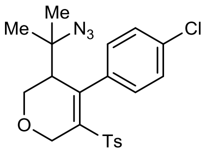
Me
Me
N3
Cl
O
Ts

8g

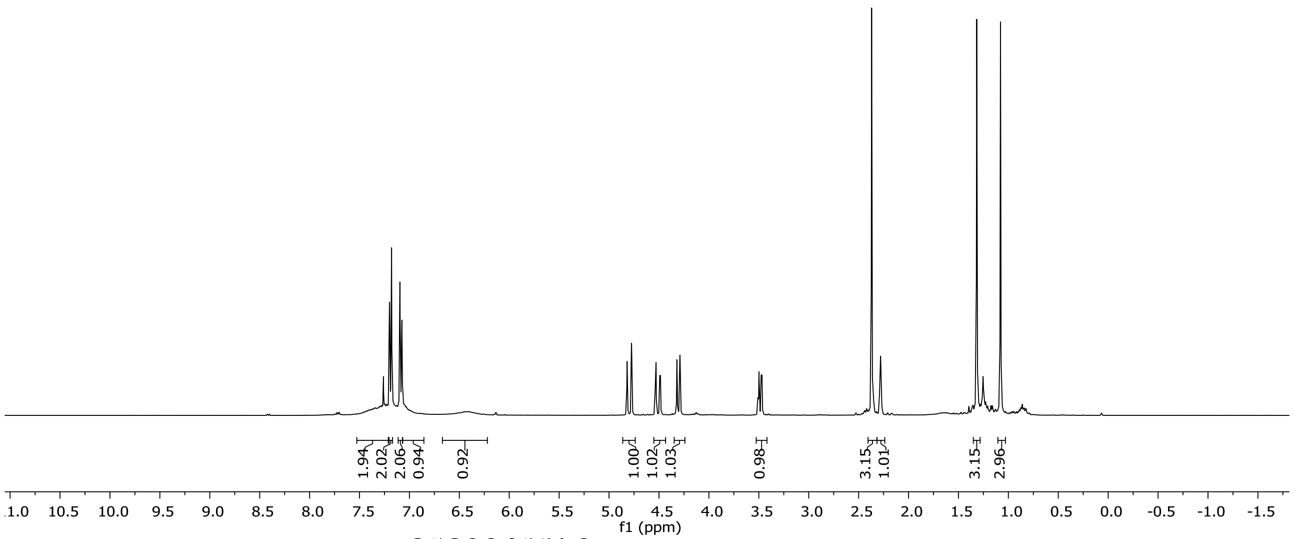
1.94
2.02
2.06
0.94
0.92
1.00
1.02
1.03
0.98
3.15
1.01
3.15
2.96
f1 (ppm)

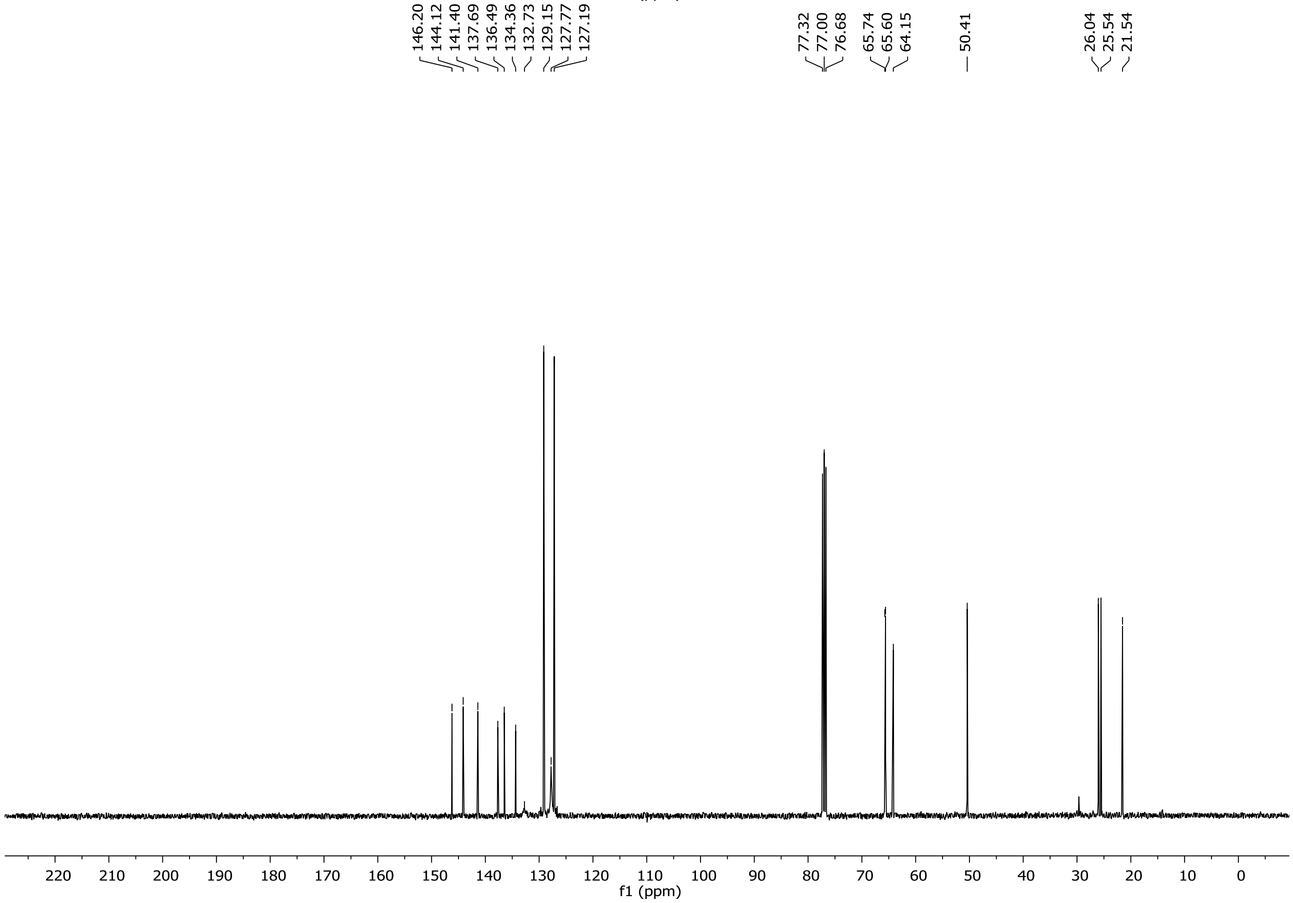
146.20
144.12
141.40
137.69
136.49
134.36
132.73
129.15
127.77
127.19
77.32
77.00
76.68
65.74
65.60
64.15
50.41
26.04
25.54
21.54
f1 (ppm)

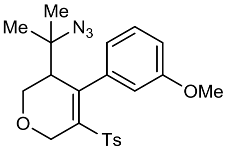
Me
Me
N3
OMe
O
Ts

8h

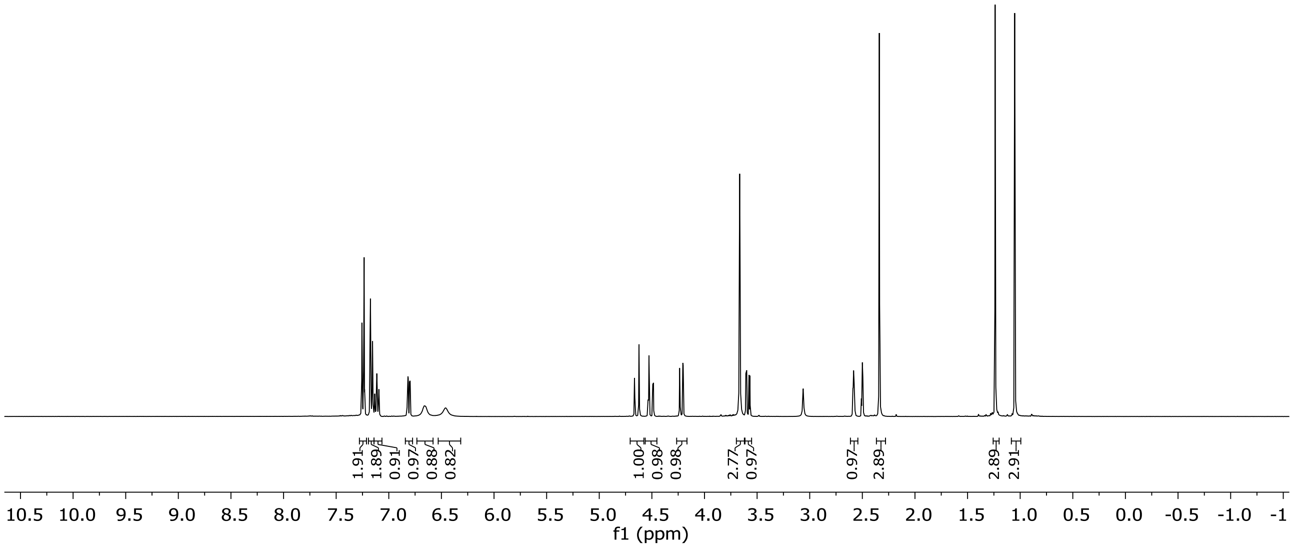
1.91
1.89
0.91
0.97
0.88
0.82
1.00
0.98
0.98
2.77
0.97
0.97
2.89
2.89
2.91
10.5 10.0 9.5 9.0 8.5 8.0 7.5 7.0 6.5 6.0 5.5 5.0 4.5 4.0 3.5 3.0 2.5 2.0 1.5 1.0 0.5 0.0 -0.5 -1.0 -1
f1 (ppm)

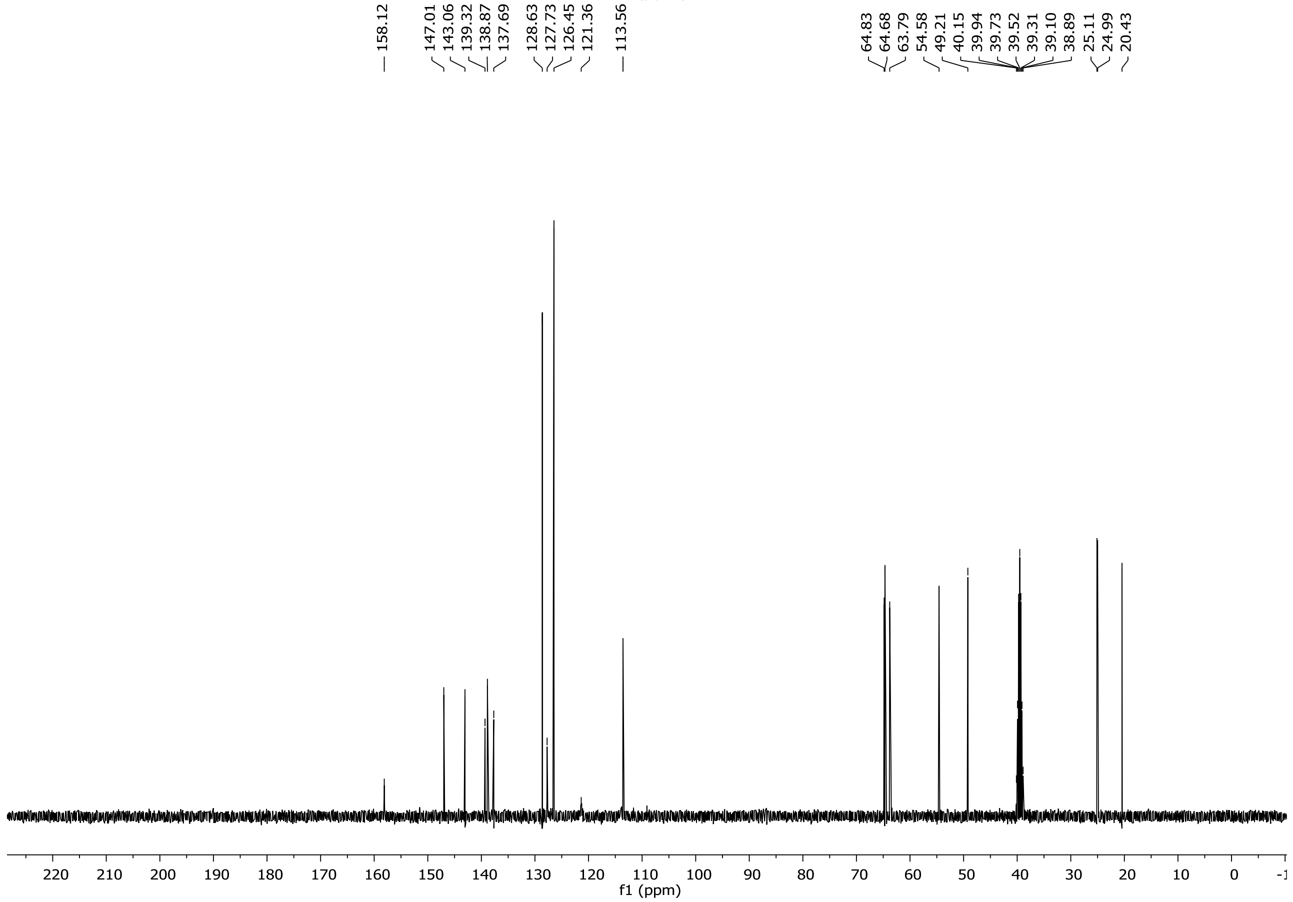
158.12
147.01
143.06
139.32
138.87
137.69
128.63
127.73
126.45
121.36
113.56
64.83
64.68
63.79
54.58
49.21
40.15
39.94
39.73
39.52
39.31
39.10
38.89
25.11
24.99
20.43
220 210 200 190 180 170 160 150 140 130 120 110 100 90 80 70 60 50 40 30 20 10 0 -1
f1 (ppm)

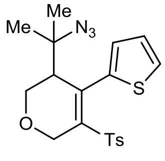
Me
Me
N3
S
O
Ts

8i

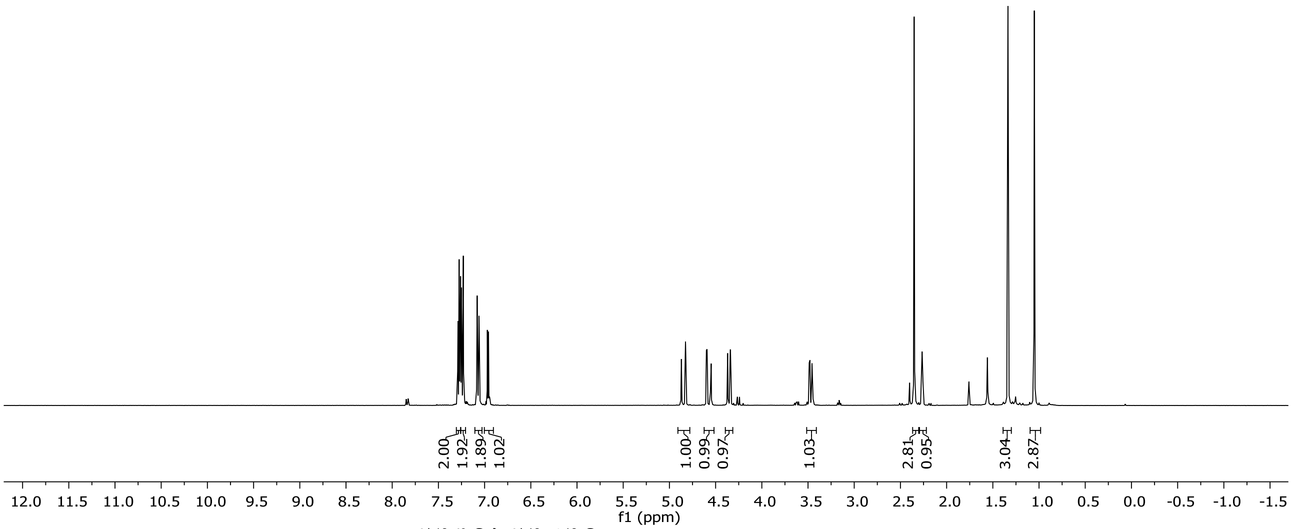
2.00
1.92
1.89
1.02
1.00
0.99
0.97
1.03
2.81
0.95
3.04
2.87
f1 (ppm)

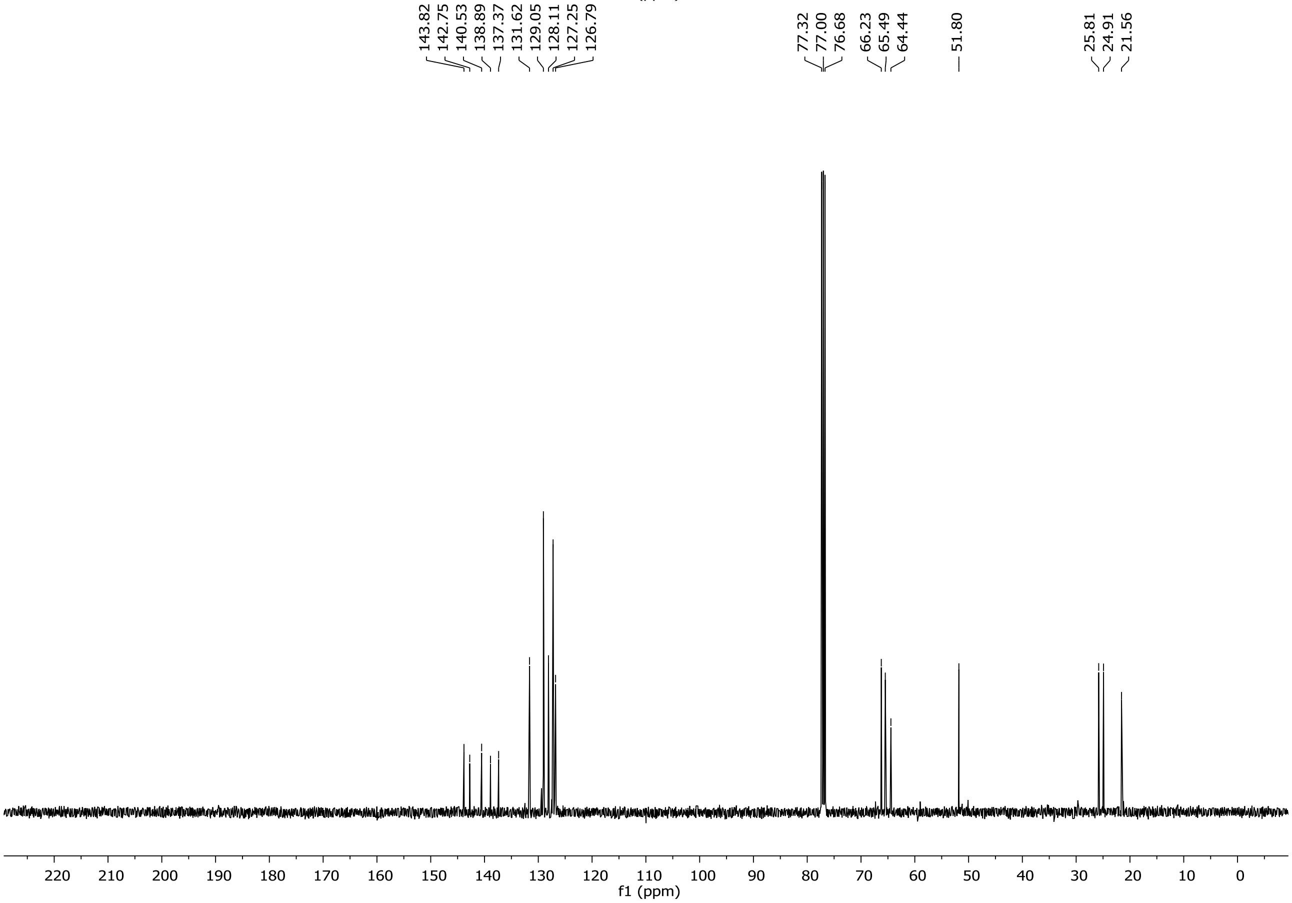
143.82
142.75
140.53
138.89
137.37
131.62
129.05
128.11
127.25
126.79
77.32
77.00
76.68
66.23
65.49
64.44
51.80
25.81
24.91
21.56
f1 (ppm)

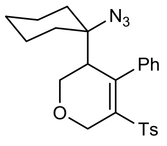
N3
Ph
O
Ts

8j

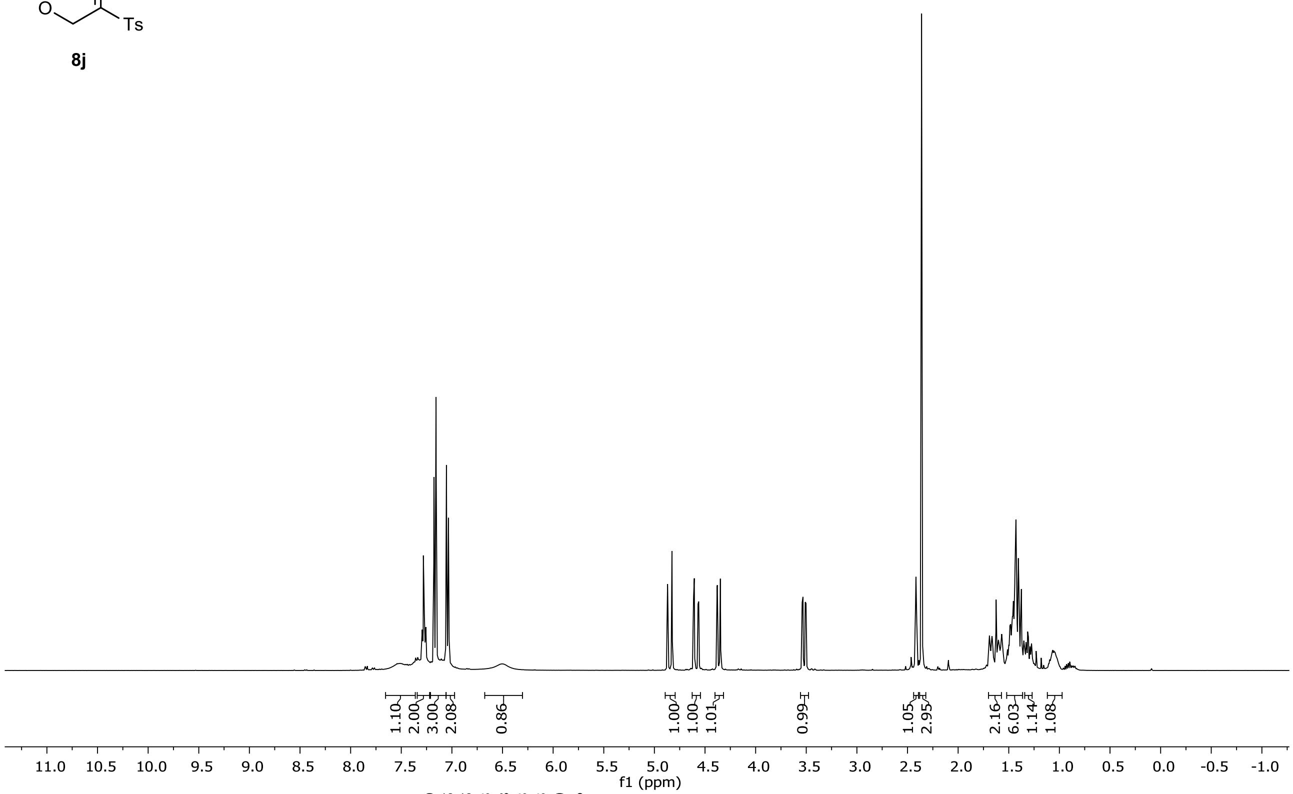
1.10
2.00
3.00
2.08
0.86
1.00
1.00
1.01
0.99
1.05
2.95
2.16
6.03
1.14
1.08
11.0 10.5 10.0 9.5 9.0 8.5 8.0 7.5 7.0 6.5 6.0 5.5 5.0 4.5 4.0 3.5 3.0 2.5 2.0 1.5 1.0 0.5 0.0 -0.5 -1.0
f1 (ppm)

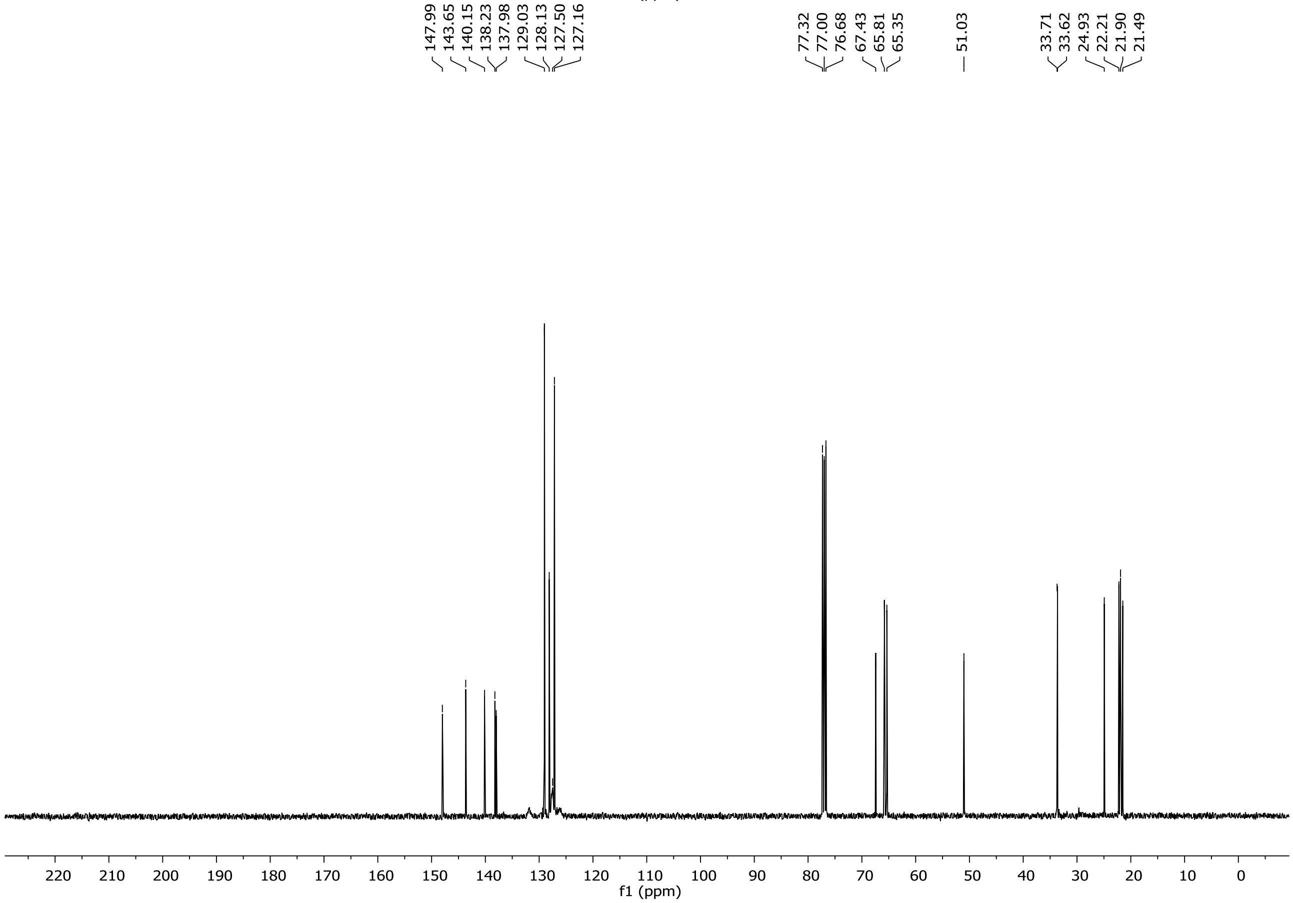
147.99
143.65
140.15
138.23
137.98
129.03
128.13
127.50
127.16
77.32
77.00
76.68
67.43
65.81
65.35
51.03
33.71
33.62
24.93
22.21
21.90
21.49
220 210 200 190 180 170 160 150 140 130 120 110 100 90 80 70 60 50 40 30 20 10 0
f1 (ppm)

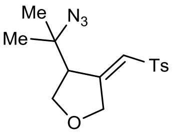
Me
N3
Me
Ts
O

10a

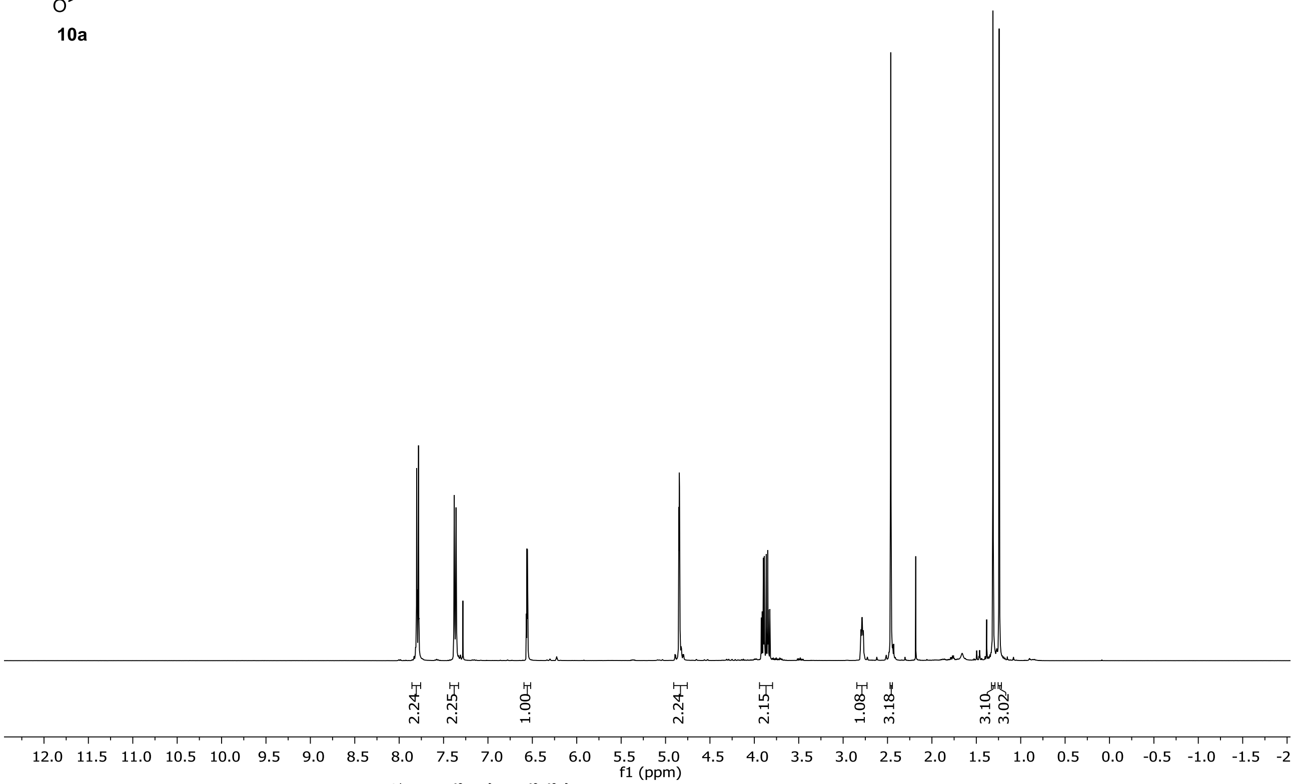
2.24
2.25
1.00
2.24
2.15
1.08
3.18
3.10
3.02
12.0 11.5 11.0 10.5 10.0 9.5 9.0 8.5 8.0 7.5 7.0 6.5 6.0 5.5 5.0 4.5 4.0 3.5 3.0 2.5 2.0 1.5 1.0 0.5 0.0 -0.5 -1.0 -1.5 -2
f1 (ppm)

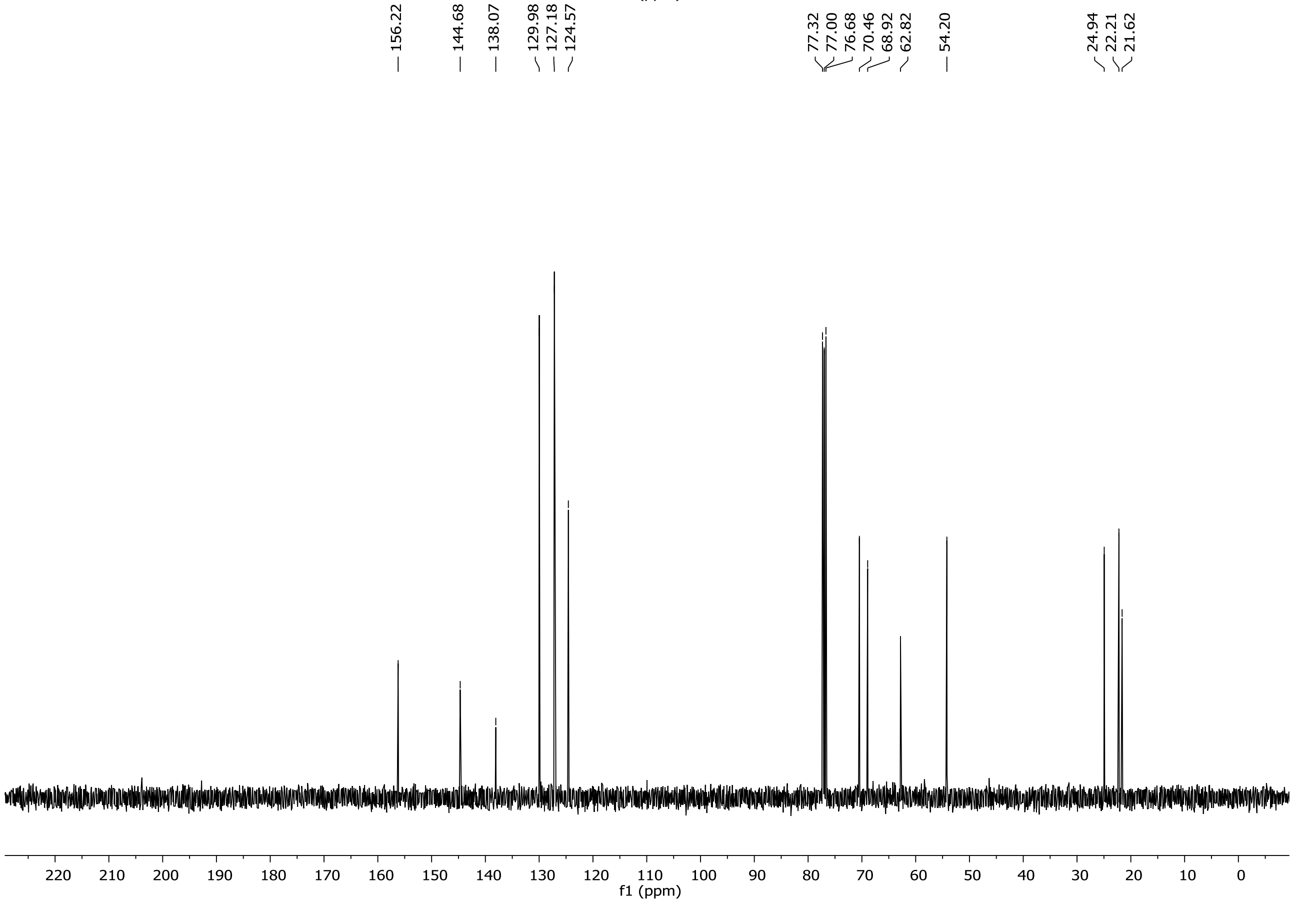
156.22
144.68
138.07
129.98
127.18
124.57
77.32
77.00
76.68
70.46
68.92
62.82
54.20
24.94
22.21
21.62
220 210 200 190 180 170 160 150 140 130 120 110 100 90 80 70 60 50 40 30 20 10 0
f1 (ppm)

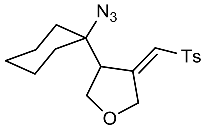
N3
Ts
O

10b

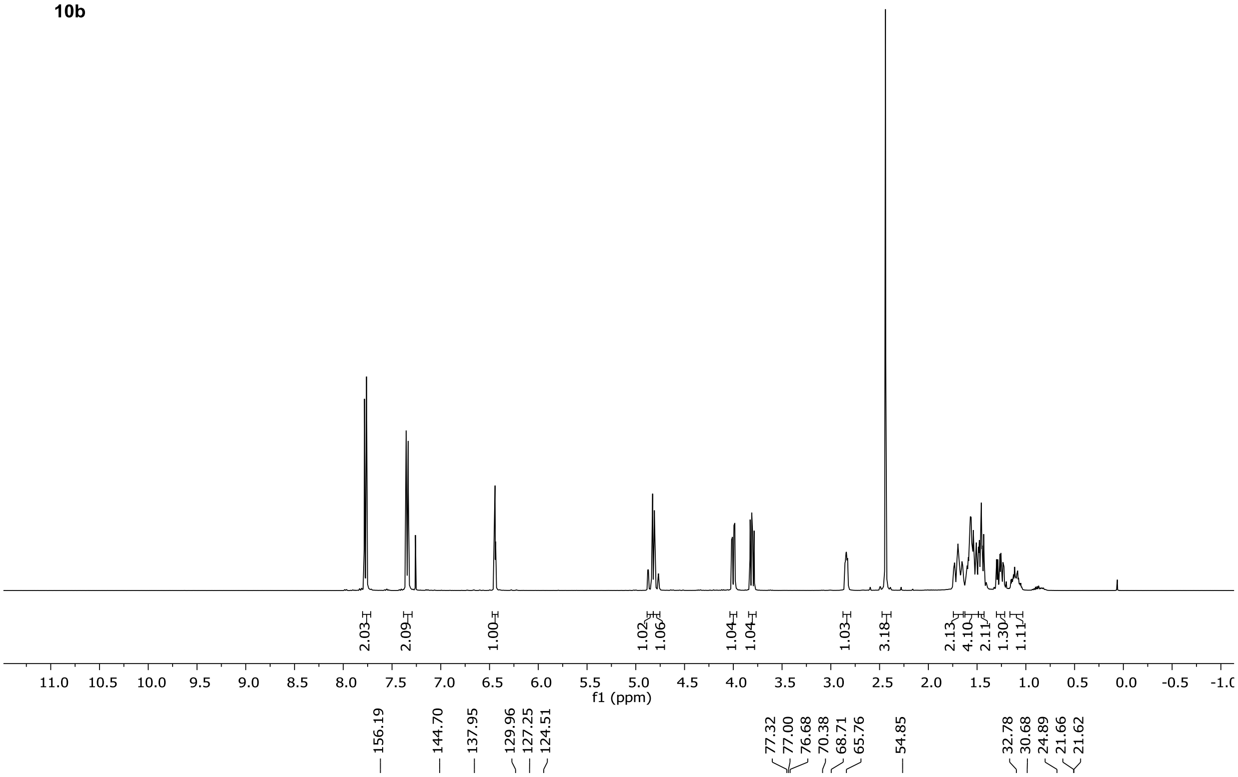
2.03
2.09
1.00
1.02
1.06
1.04
1.04
1.03
3.18
2.13
4.10
2.11
1.30
1.11
11.0 10.5 10.0 9.5 9.0 8.5 8.0 7.5 7.0 6.5 6.0 5.5 5.0 4.5 4.0 3.5 3.0 2.5 2.0 1.5 1.0 0.5 0.0 -0.5
f1 (ppm)
156.19
144.70
137.95
129.96
127.25
124.51
77.32
77.00
76.68
70.38
68.71
65.76
54.85
32.78
30.68
24.89
21.66
21.62

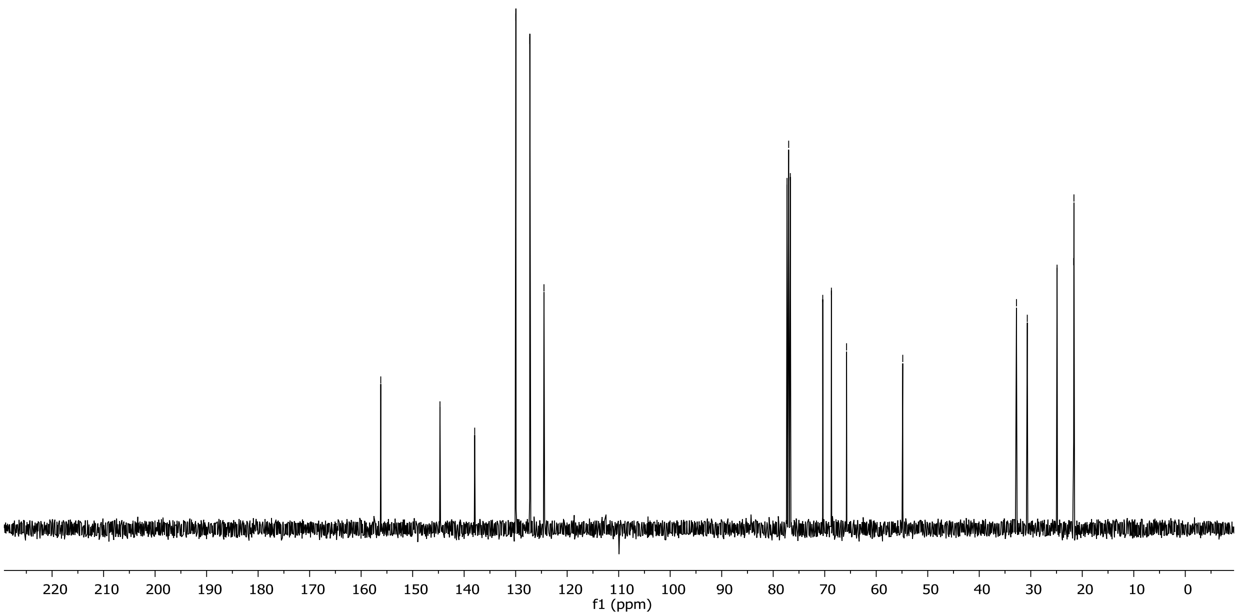
220 210 200 190 180 170 160 150 140 130 120 110 100 90 80 70 60 50 40 30 20 10 0
f1 (ppm)

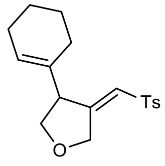

Ts
O

11

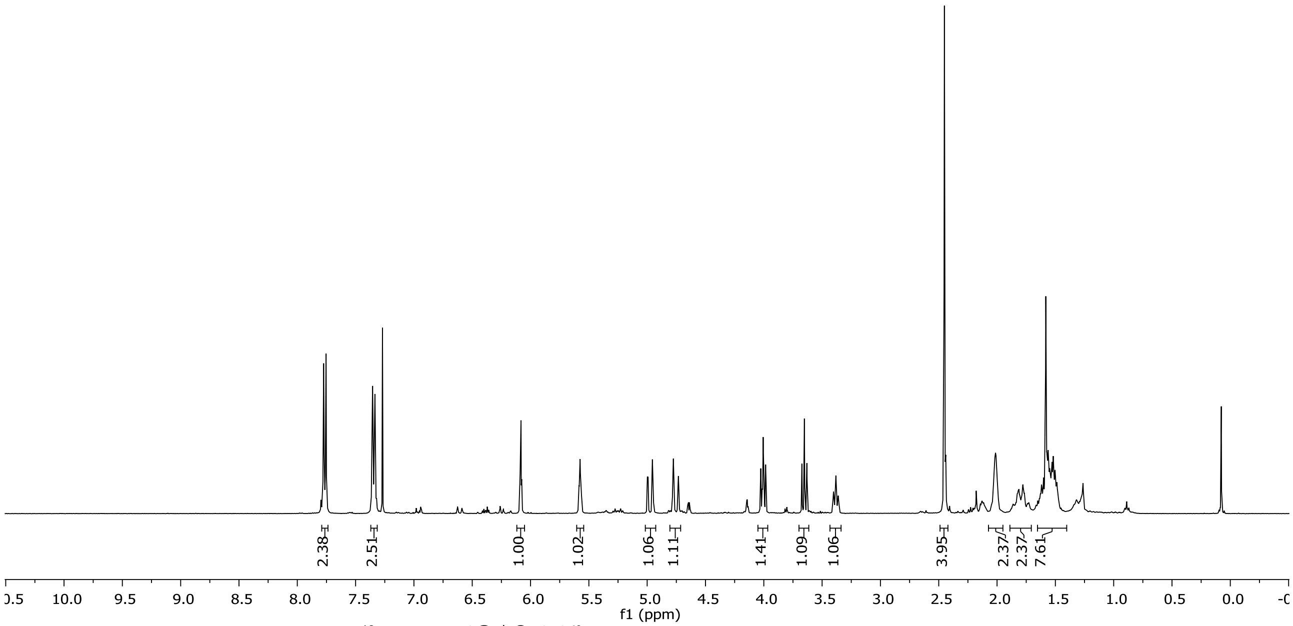

2.38
2.51
1.00
1.02
1.06
1.11
1.41
1.09
1.06
3.95
2.37
2.37
7.61
f1 (ppm)

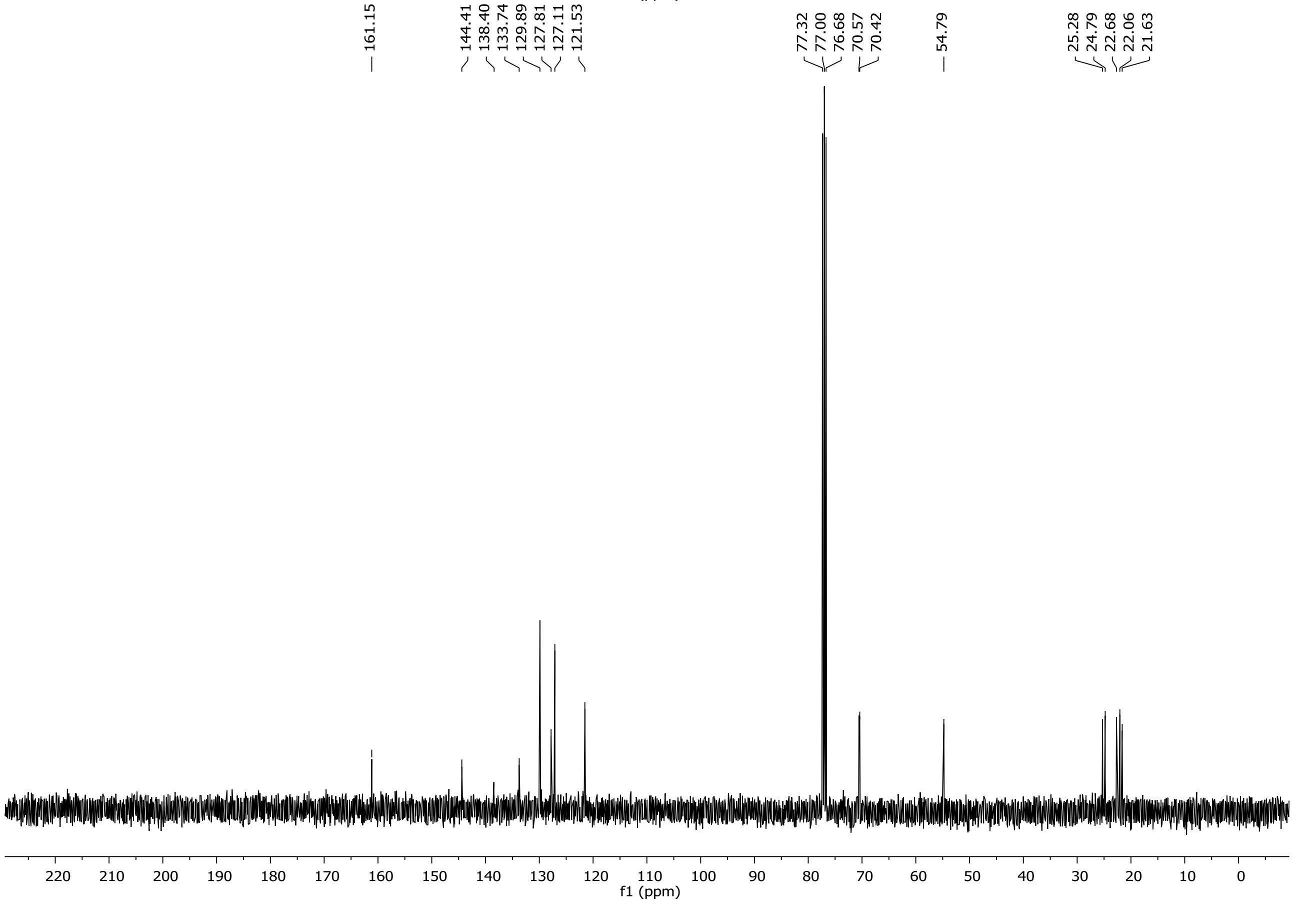

161.15
144.41
138.40
133.74
129.89
127.81
127.11
121.53
77.32
77.00
76.68
70.57
70.42
54.79
25.28
24.79
22.68
22.06
21.63
f1 (ppm)

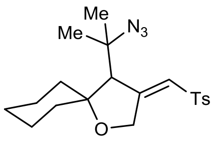
Me
Me
N3
Ts
O

10c

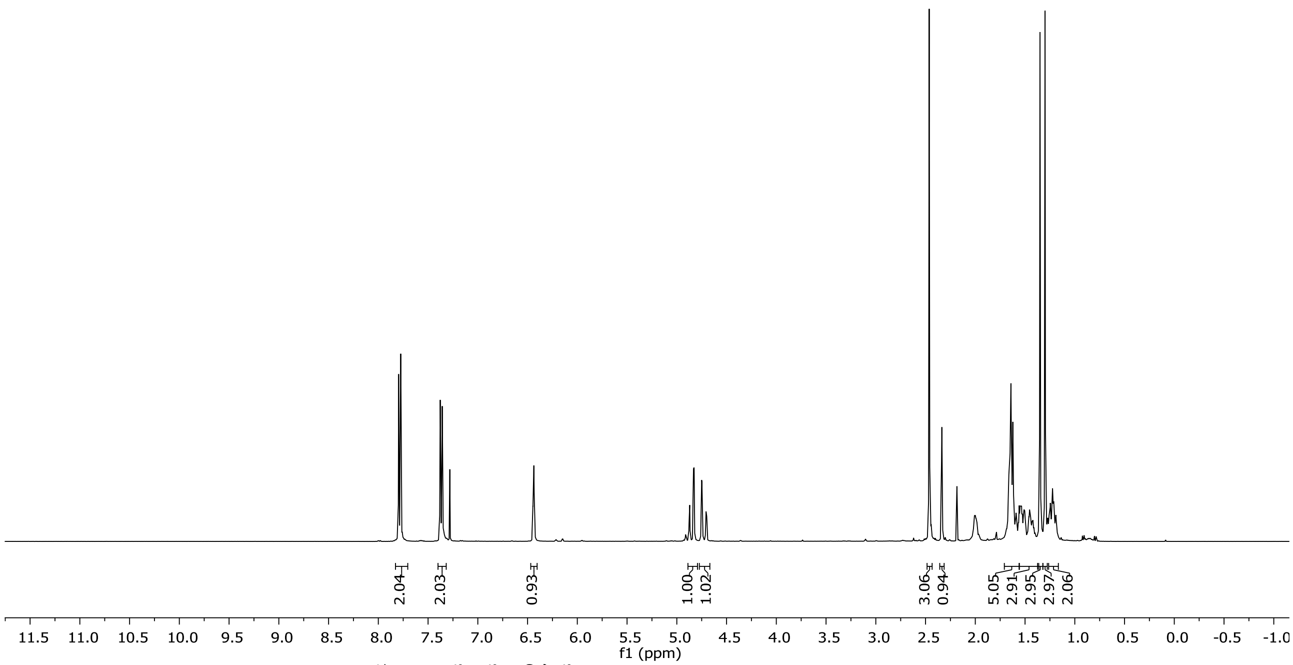
2.04
2.03
0.93
1.00
1.02
3.06
0.94
5.05
2.91
2.95
2.97
2.06
11.5 11.0 10.5 10.0 9.5 9.0 8.5 8.0 7.5 7.0 6.5 6.0 5.5 5.0 4.5 4.0 3.5 3.0 2.5 2.0 1.5 1.0 0.5 0.0 -0.5 -1.0
f1 (ppm)

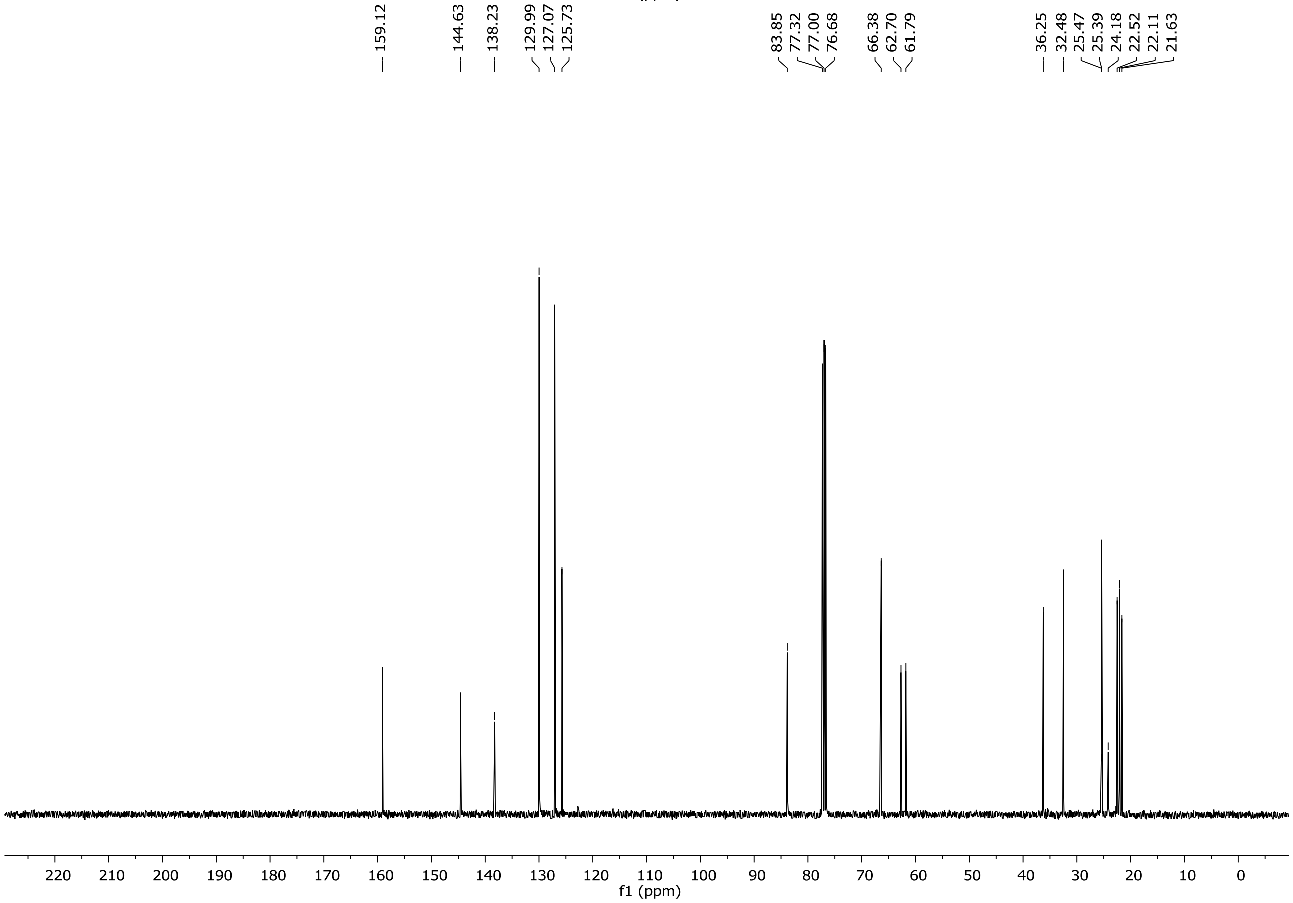
159.12
144.63
138.23
129.99
127.07
125.73
83.85
77.32
77.00
76.68
66.38
62.70
61.79
36.25
32.48
25.47
25.39
24.18
22.52
22.11
21.63
220 210 200 190 180 170 160 150 140 130 120 110 100 90 80 70 60 50 40 30 20 10 0
f1 (ppm)

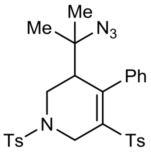
Me
Me
N3
Ph
N
Ts
Ts

8k

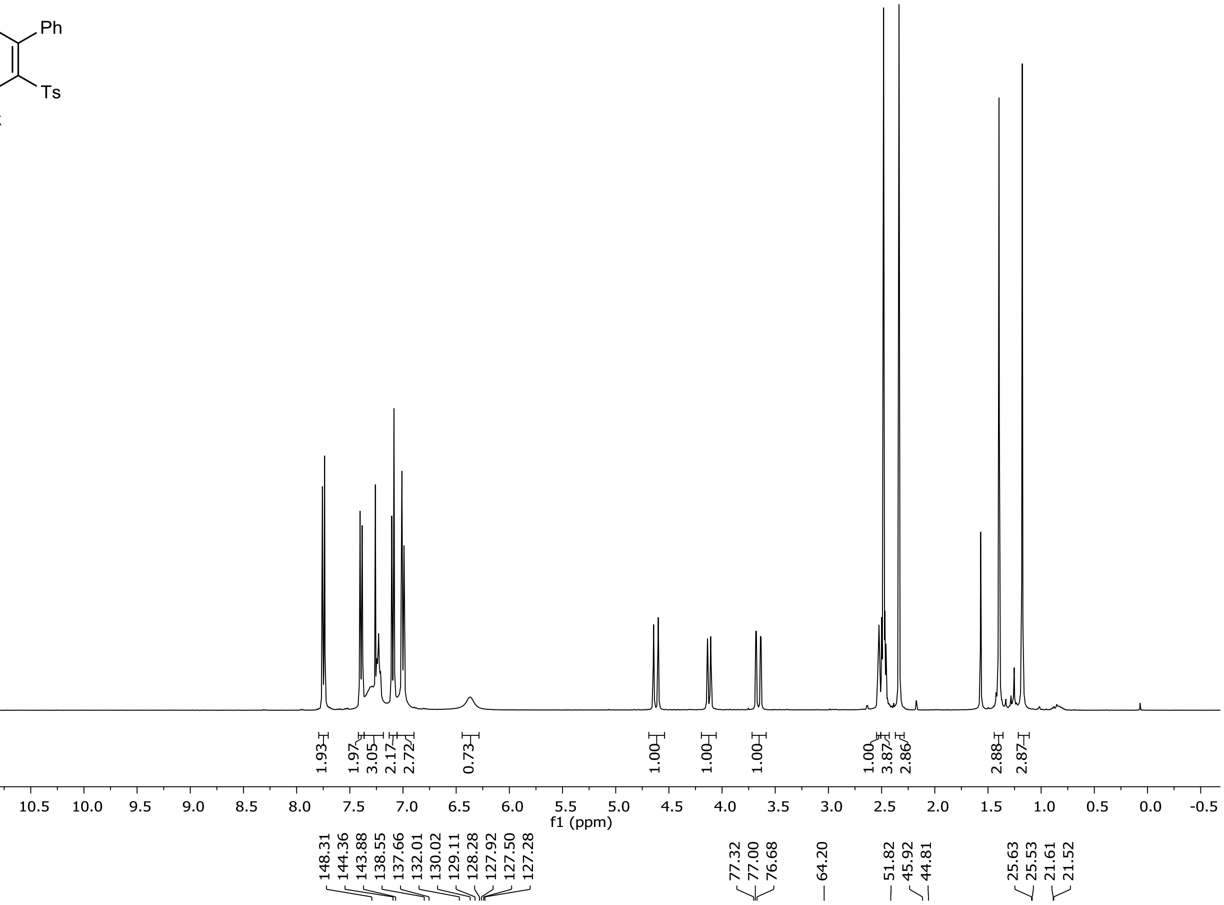
1.93
1.97
3.05
2.17
2.72
0.73
1.00
1.00
1.00
1.00
3.87
2.86
2.88
2.87
11.0 10.5 10.0 9.5 9.0 8.5 8.0 7.5 7.0 6.5 6.0 5.5 5.0 4.5 4.0 3.5 3.0 2.5 2.0 1.5 1.0 0.5 0.0 -0.5
f1 (ppm)
148.31
144.36
143.88
138.55
137.66
132.01
130.02
129.11
128.28
127.92
127.50
127.28
77.32
77.00
76.68
64.20
51.82
45.92
44.81
25.63
25.53
21.61
21.52

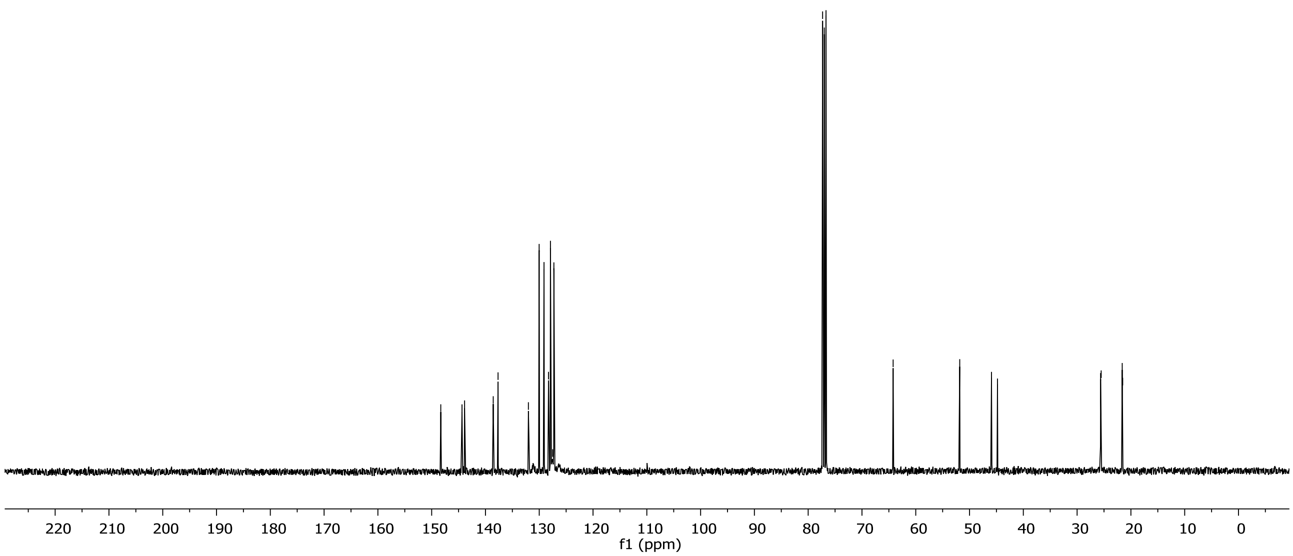
220 210 200 190 180 170 160 150 140 130 120 110 100 90 80 70 60 50 40 30 20 10 0
f1 (ppm)

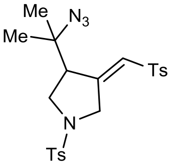
Me
N3
Me
Ts
N
Ts

10d

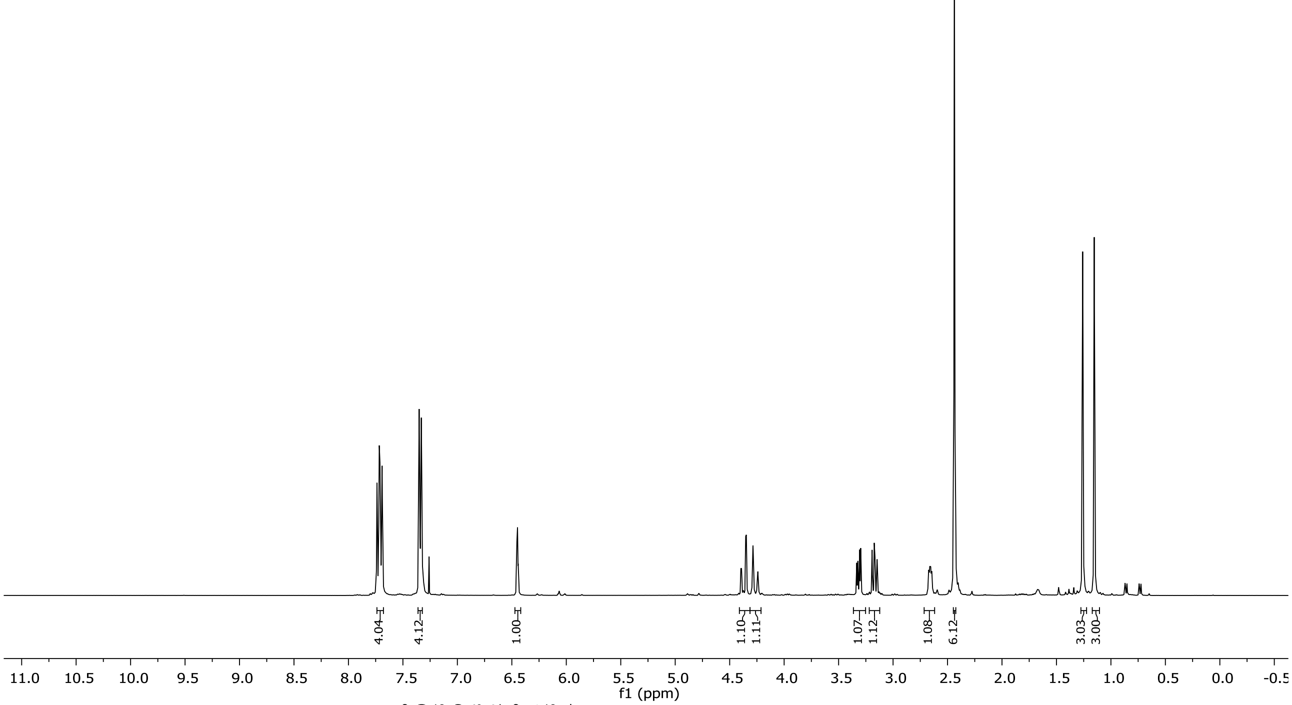
4.04
4.12
1.00
1.10
1.11
1.07
1.12
1.08
6.12
3.03
3.00
11.0 10.5 10.0 9.5 9.0 8.5 8.0 7.5 7.0 6.5 6.0 5.5 5.0 4.5 4.0 3.5 3.0 2.5 2.0 1.5 1.0 0.5 0.0 -0.5
f1 (ppm)

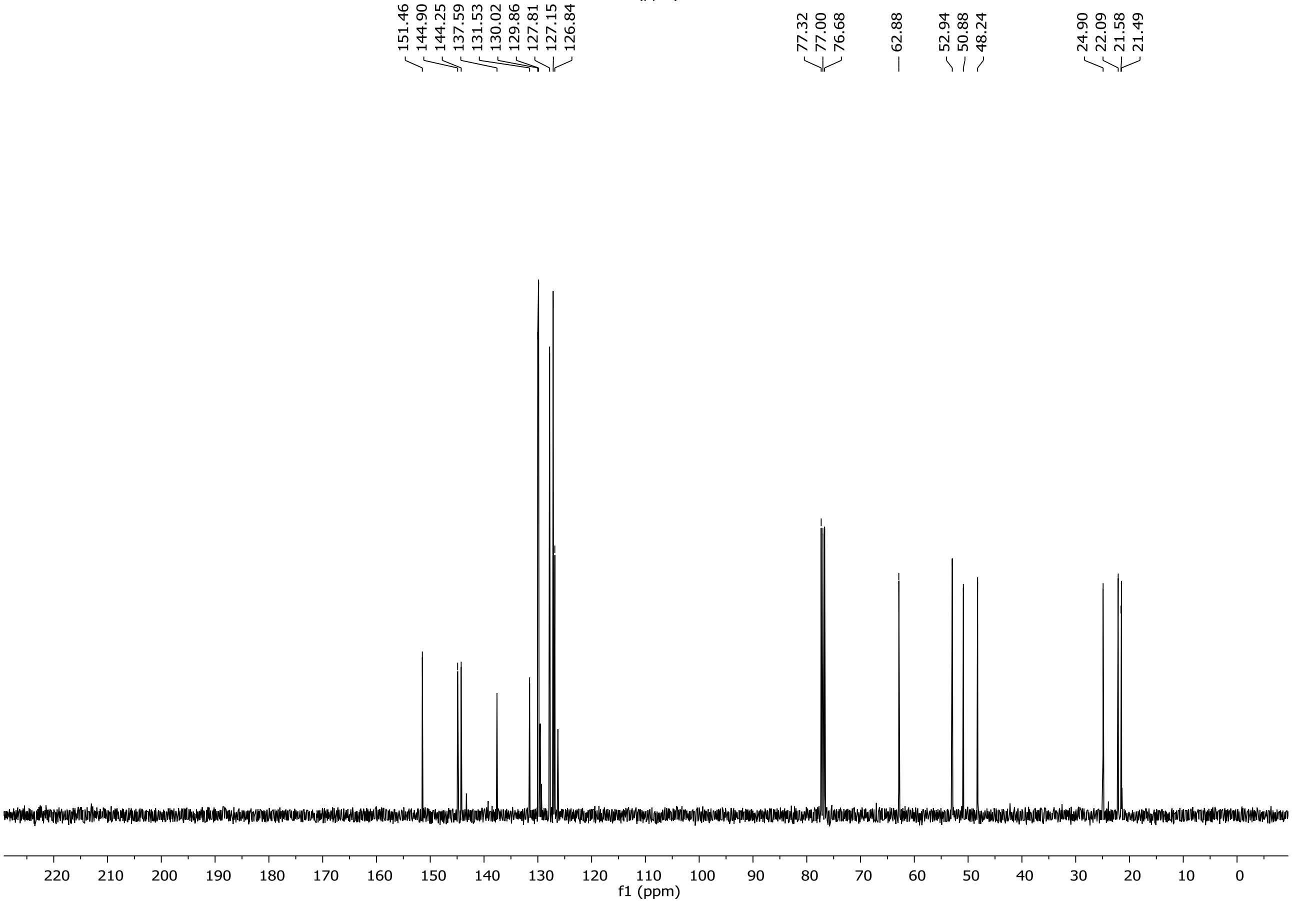
151.46
144.90
144.25
137.59
131.53
130.02
129.86
127.81
127.15
126.84
77.32
77.00
76.68
62.88
52.94
50.88
48.24
24.90
22.09
21.58
21.49
220 210 200 190 180 170 160 150 140 130 120 110 100 90 80 70 60 50 40 30 20 10 0
f1 (ppm)

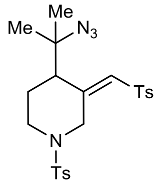
Me
Me
N3
Ts
N
Ts

10e

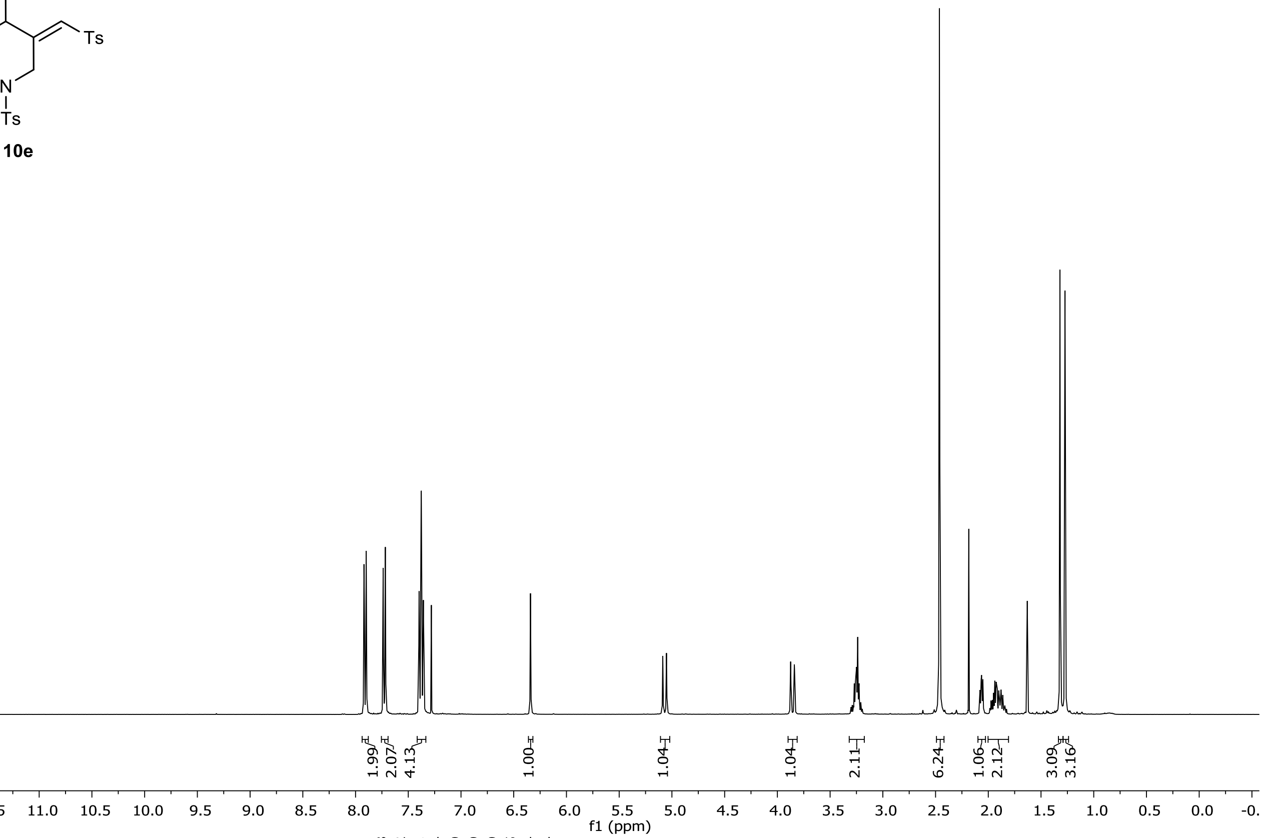
1.99
2.07
4.13
1.00
1.04
1.04
2.11
6.24
1.06
2.12
3.09
3.16
11.5 11.0 10.5 10.0 9.5 9.0 8.5 8.0 7.5 7.0 6.5 6.0 5.5 5.0 4.5 4.0 3.5 3.0 2.5 2.0 1.5 1.0 0.5 0.0 -0.
f1 (ppm)

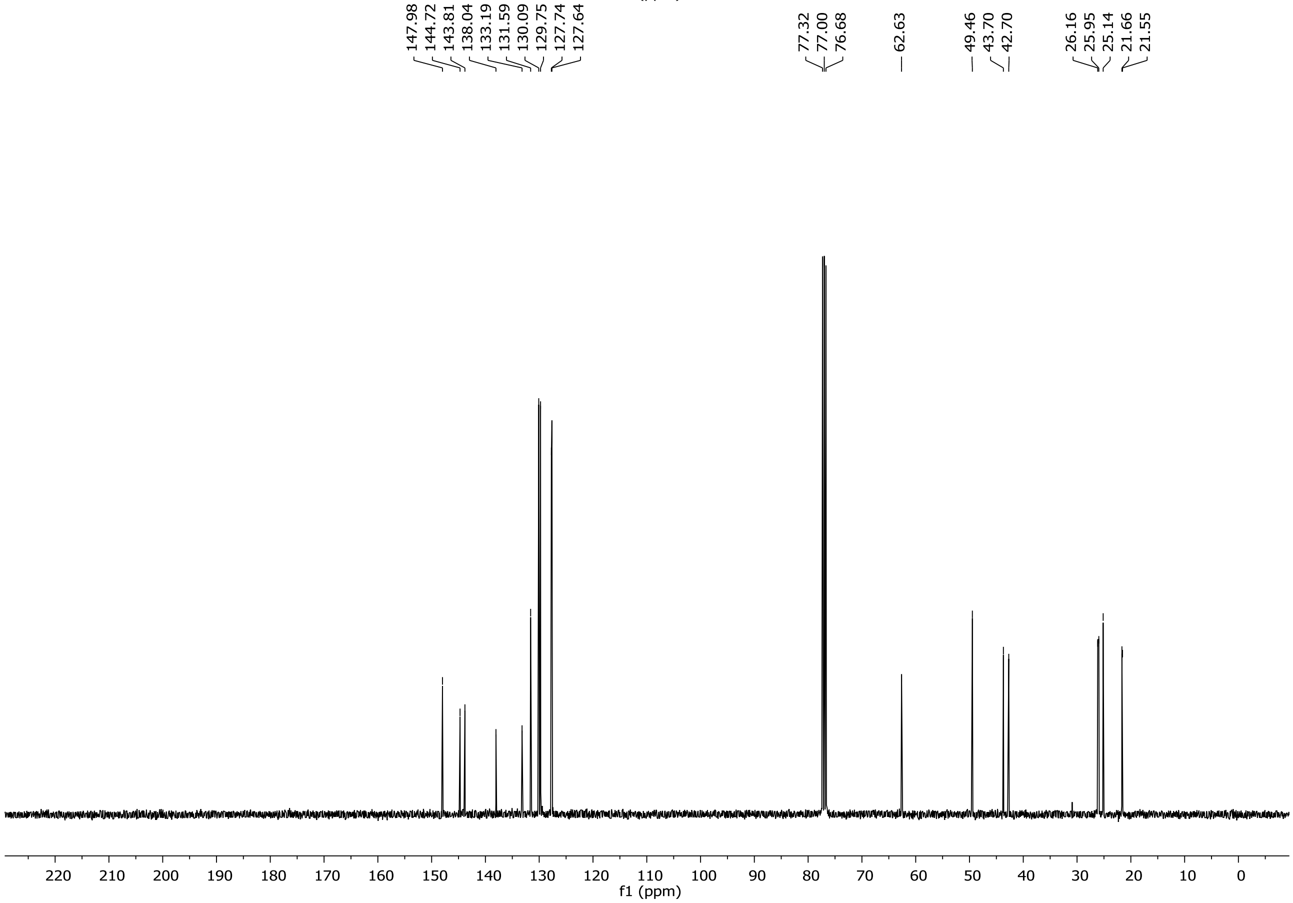
147.98
144.72
143.81
138.04
133.19
131.59
130.09
129.75
127.74
127.64
77.32
77.00
76.68
62.63
49.46
43.70
42.70
26.16
25.95
25.14
21.66
21.55
220 210 200 190 180 170 160 150 140 130 120 110 100 90 80 70 60 50 40 30 20 10 0
f1 (ppm)

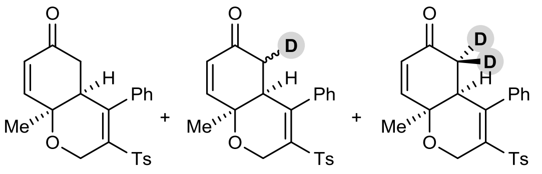

3a (minor) | [D]-**3a** (major) diastereomeric mixture | $[D]_2$-**3a** (trace)

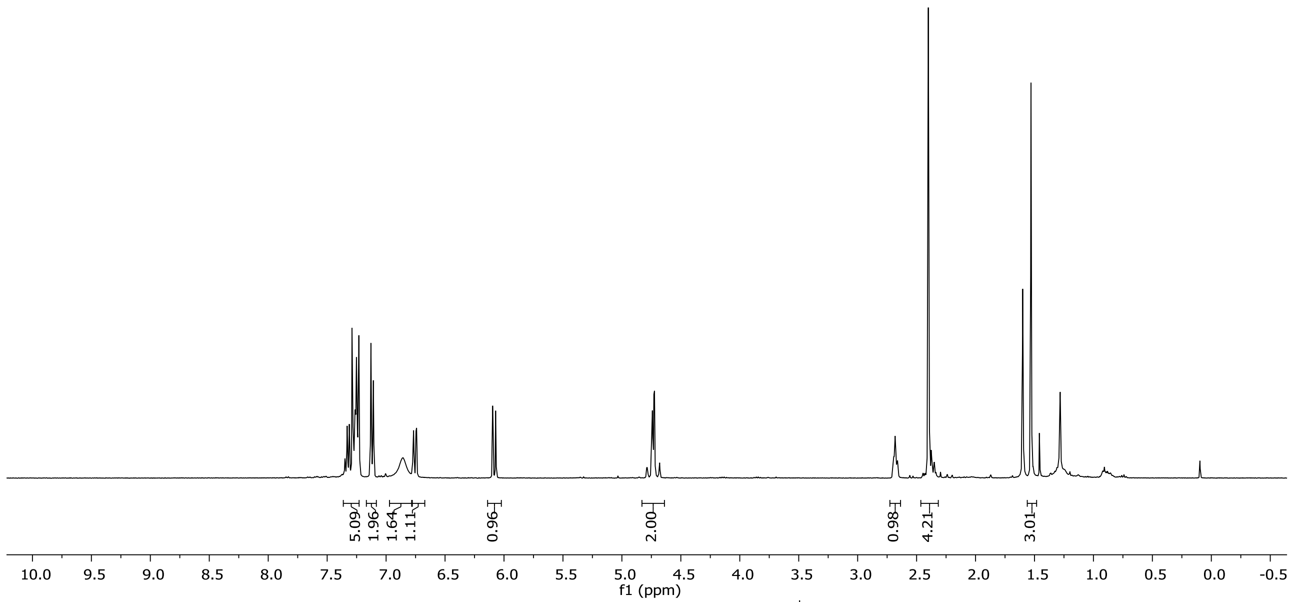

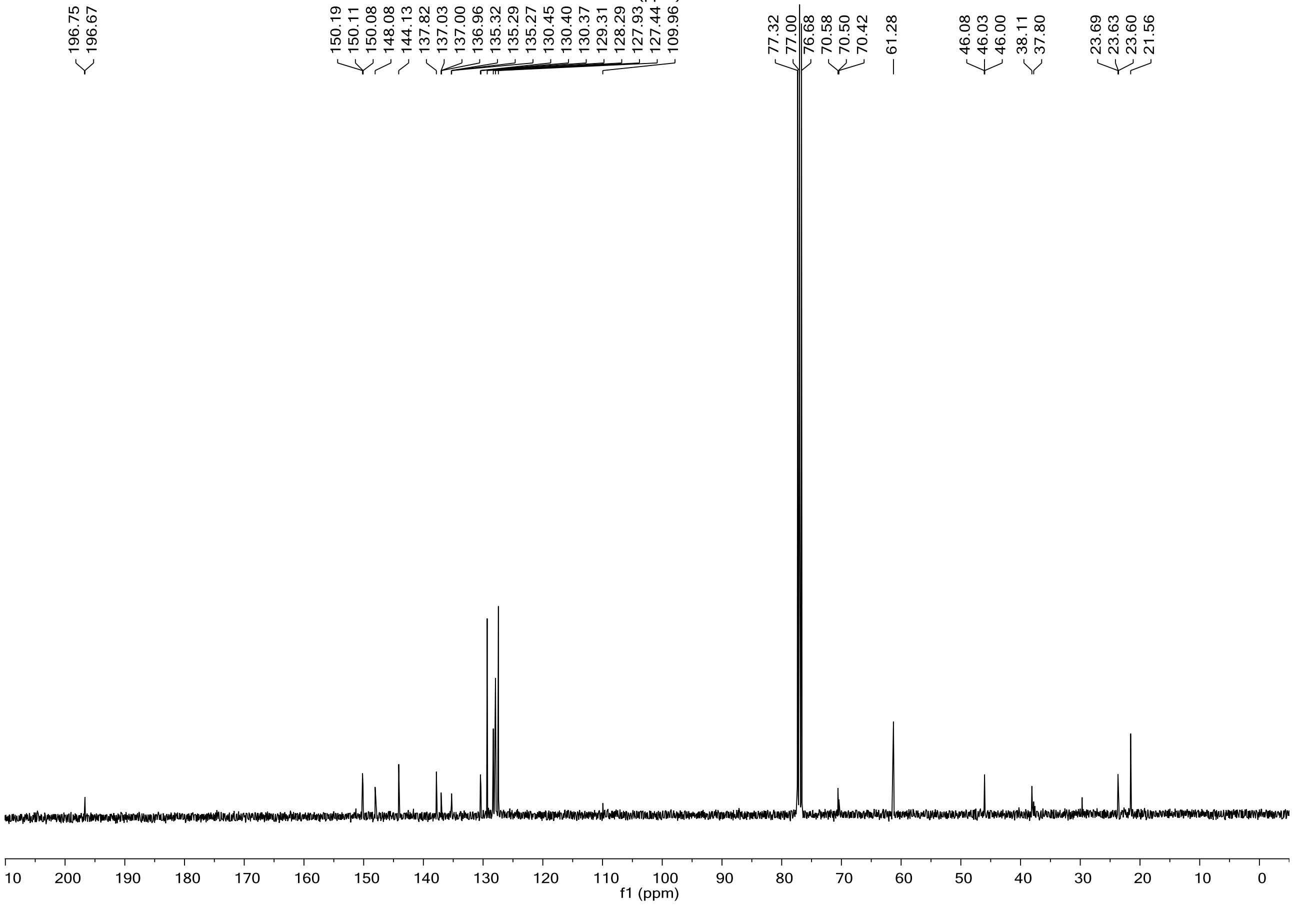

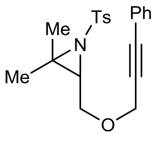
Me
Ts
Ph
N
Me
O

24

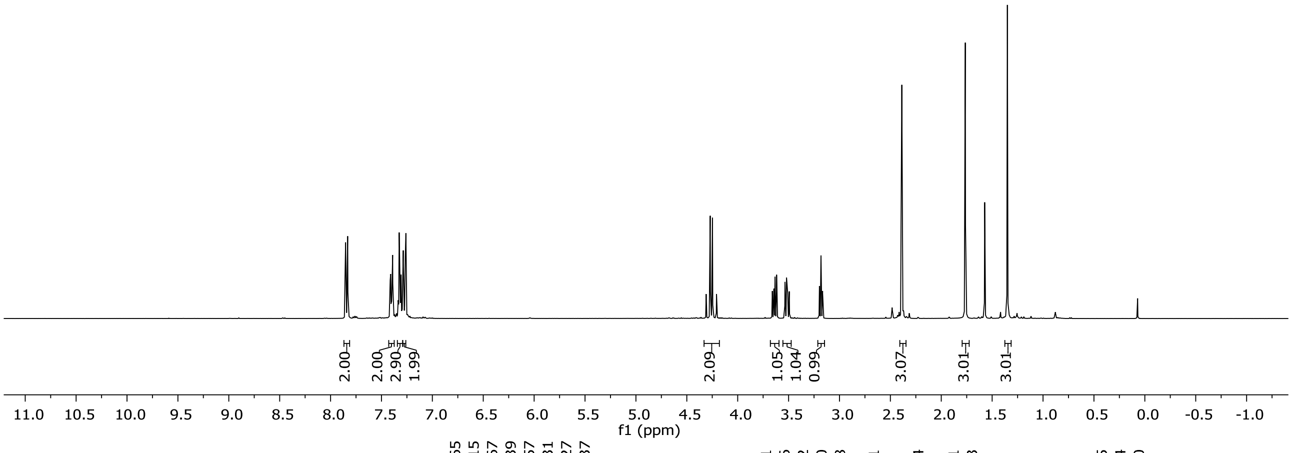
2.00
2.00
2.90
1.99
2.09
1.05
1.04
0.99
3.07
3.01
3.01
11.0 10.5 10.0 9.5 9.0 8.5 8.0 7.5 7.0 6.5 6.0 5.5 5.0 4.5 4.0 3.5 3.0 2.5 2.0 1.5 1.0 0.5 0.0 -0.5 -1.0
f1 (ppm)

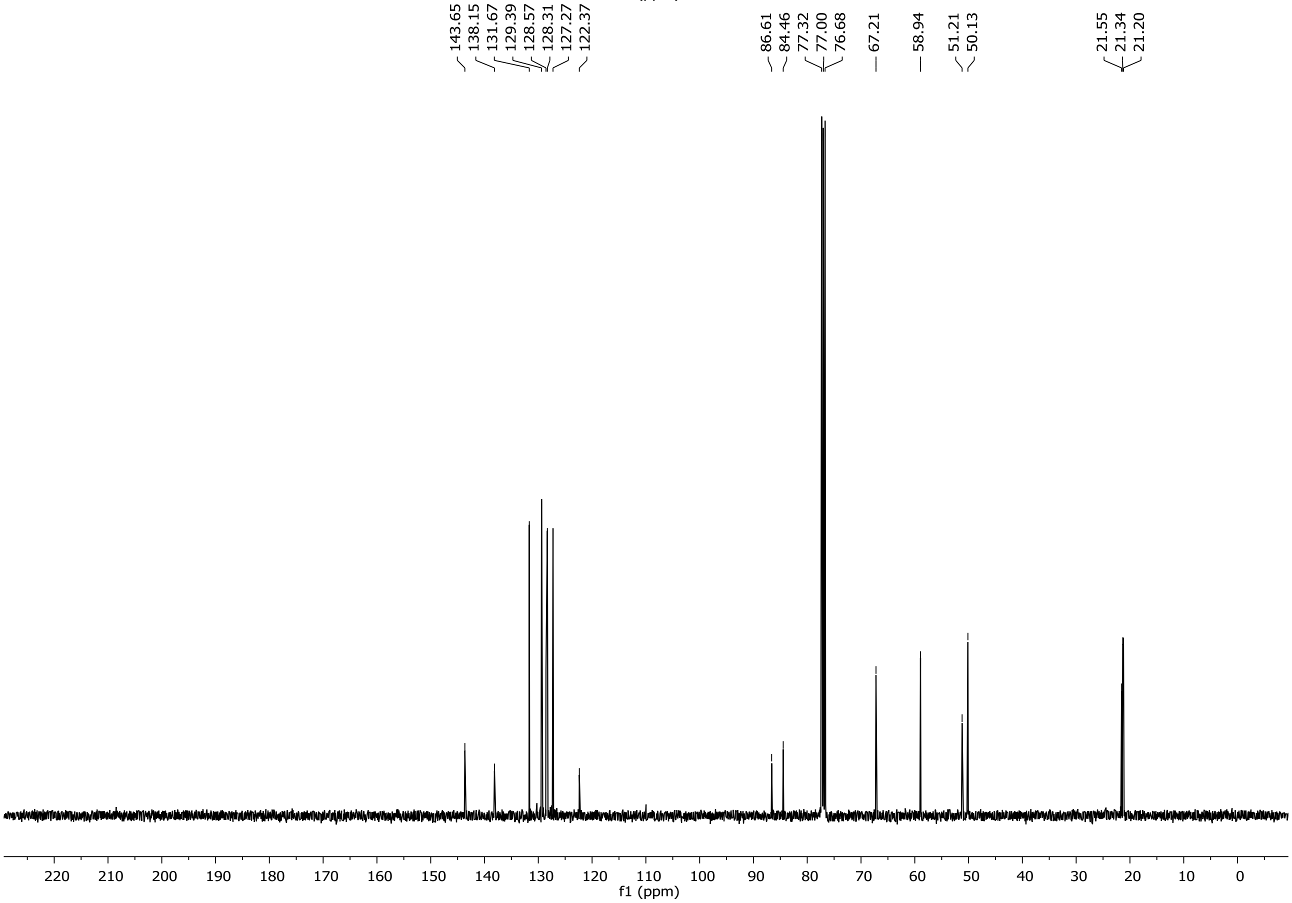
143.65
138.15
131.67
129.39
128.57
128.31
127.27
122.37
86.61
84.46
77.32
77.00
76.68
67.21
58.94
51.21
50.13
21.55
21.34
21.20
220 210 200 190 180 170 160 150 140 130 120 110 100 90 80 70 60 50 40 30 20 10 0
f1 (ppm)

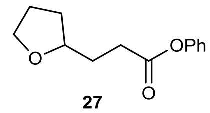
OPh
O
O

27

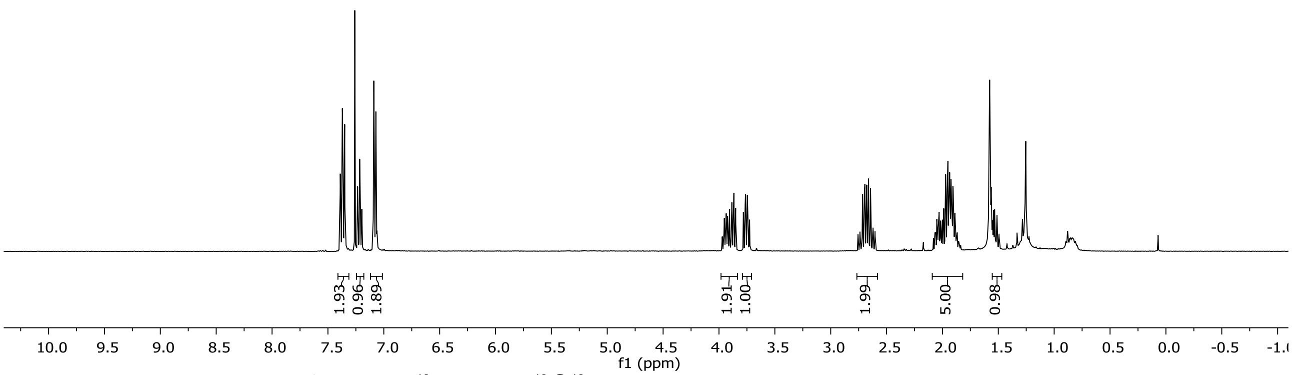
1.93
0.96
1.89
1.91
1.00
1.99
5.00
0.98
10.0 9.5 9.0 8.5 8.0 7.5 7.0 6.5 6.0 5.5 5.0 4.5 4.0 3.5 3.0 2.5 2.0 1.5 1.0 0.5 0.0 -0.5
f1 (ppm)

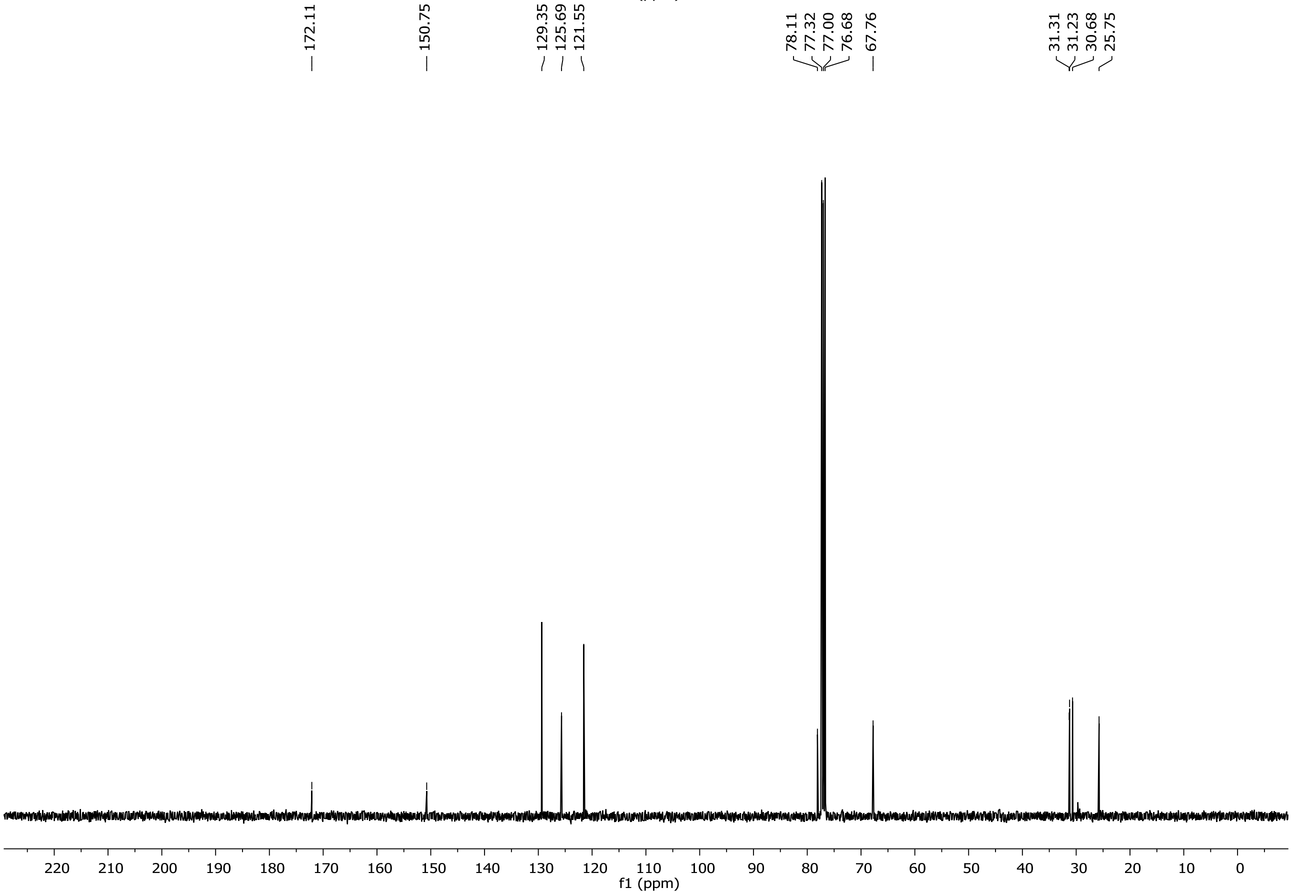
172.11
150.75
129.35
125.69
121.55
78.11
77.32
77.00
76.68
67.76
31.31
31.23
30.68
25.75
220 210 200 190 180 170 160 150 140 130 120 110 100 90 80 70 60 50 40 30 20 10 0
f1 (ppm)

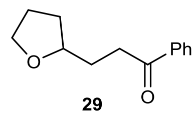
O
Ph
O

29

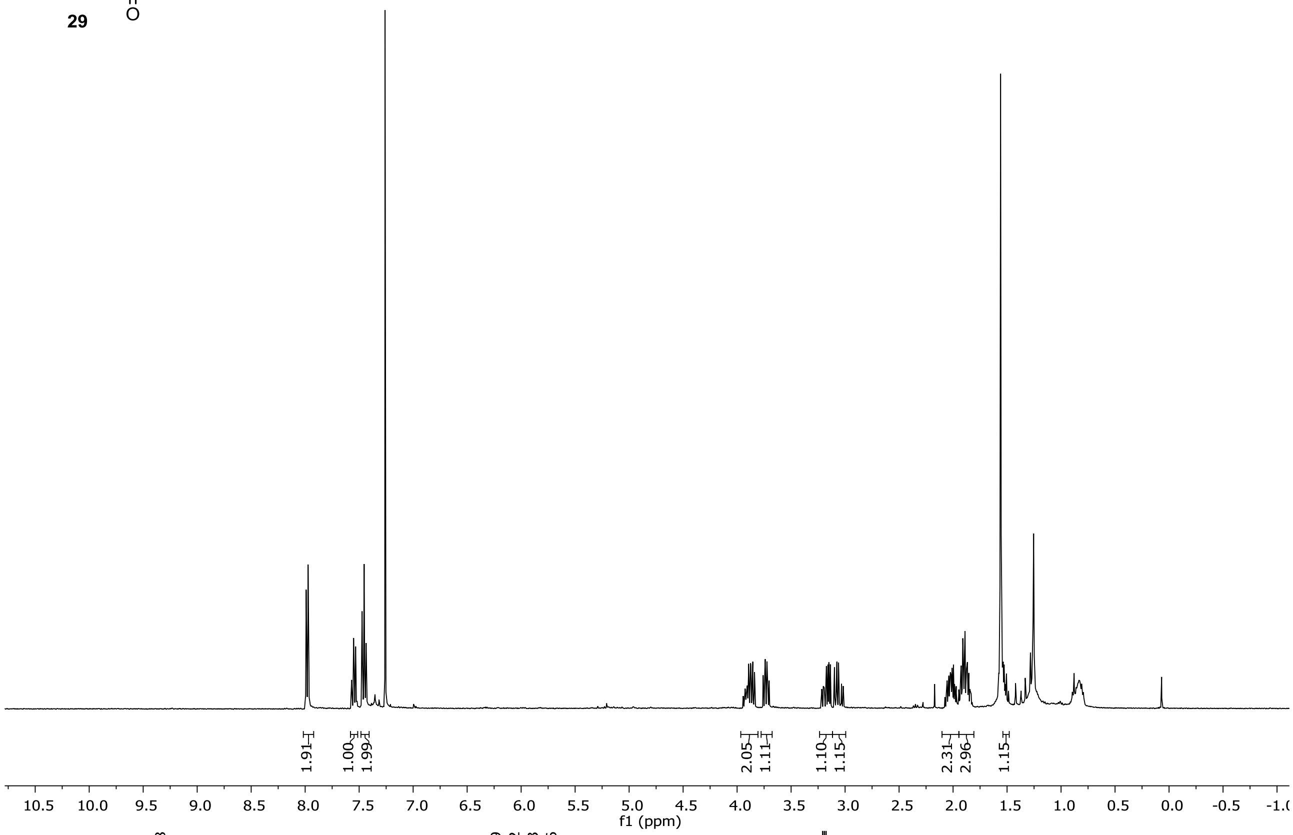
1.91
1.00
1.99
2.05
1.11
1.10
1.15
2.31
2.96
1.15
10.5 10.0 9.5 9.0 8.5 8.0 7.5 7.0 6.5 6.0 5.5 5.0 4.5 4.0 3.5 3.0 2.5 2.0 1.5 1.0 0.5 0.0 -0.5
f1 (ppm)

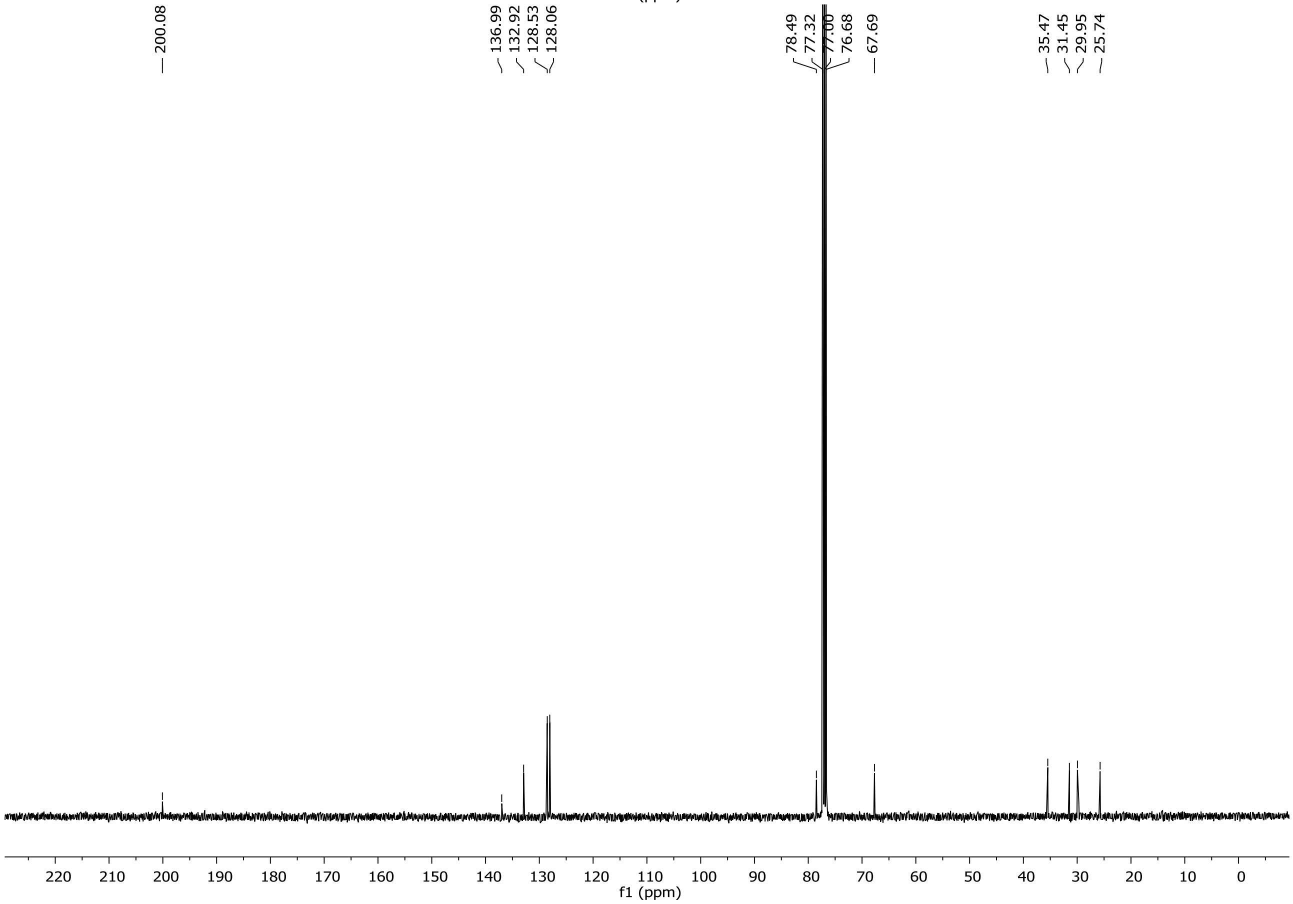
200.08
136.99
132.92
128.53
128.06
78.49
77.32
77.00
76.68
67.69
35.47
31.45
29.95
25.74
220 210 200 190 180 170 160 150 140 130 120 110 100 90 80 70 60 50 40 30 20 10 0
f1 (ppm)

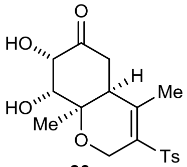
HO
HO
Me
O
H
Me
Ts

30

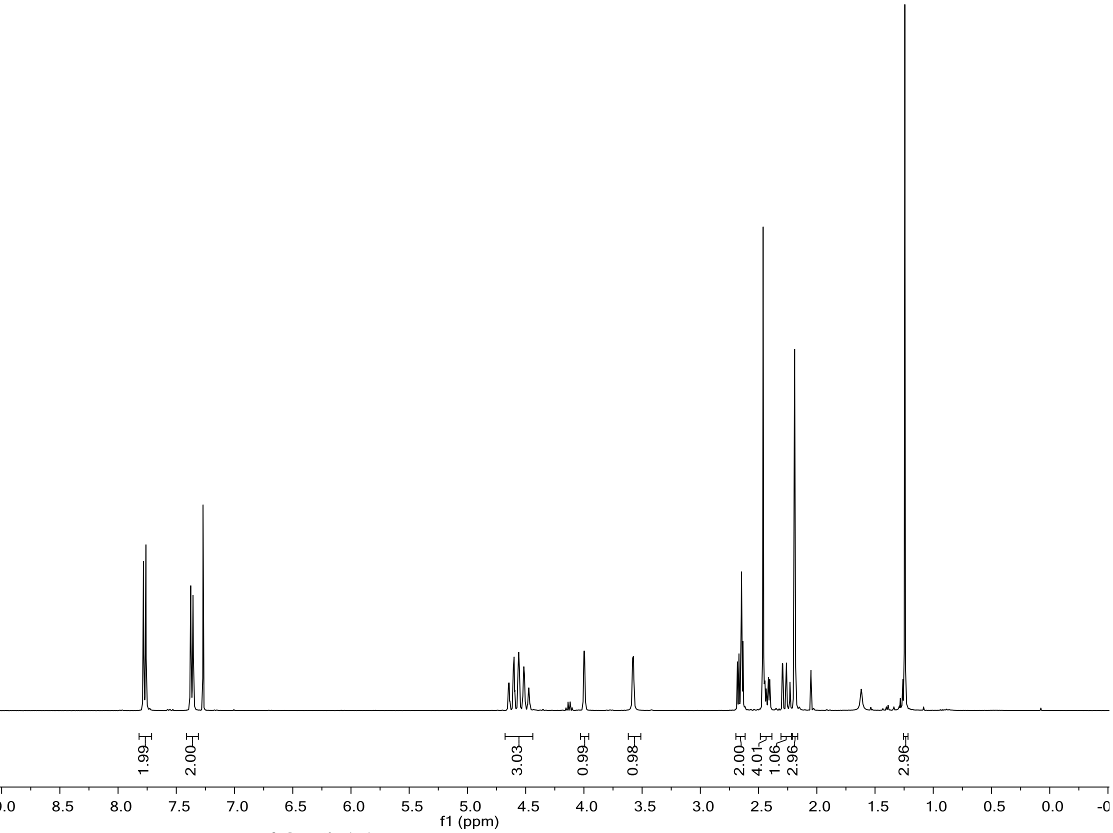
1.99
2.00
3.03
0.99
0.98
2.00
4.01
1.06
2.96
2.96
f1 (ppm)

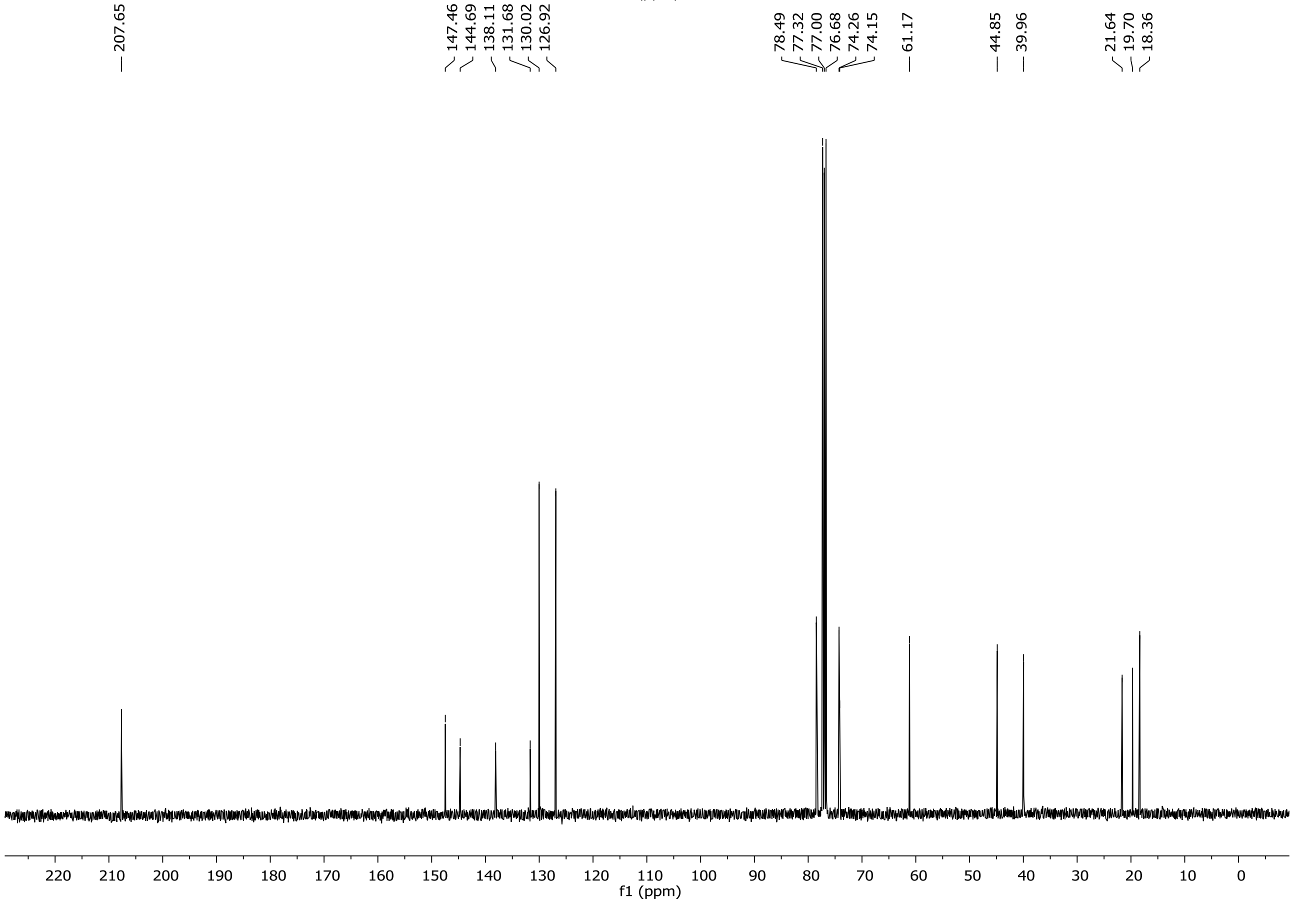
207.65
147.46
144.69
138.11
131.68
130.02
126.92
78.49
77.32
77.00
76.68
74.26
74.15
61.17
44.85
39.96
21.64
19.70
18.36
f1 (ppm)

References

1. M. S. Lowry, W. R. Hudson, R. A. Pascal, Jr., S. Bernhard, *J. Am. Chem. Soc*. **2004**, *126,* 14129–14135.
2. (a) I.-H. Lee, H. Kim, T.-L. Choi, *J. Am. Chem. Soc*. **2013**, *135,* 3760. (b) J. Waser, B. Gaspar, H. Nambu, E. M. Carreira, *J. Am. Chem. Soc*. **2006**, *128*, 11693. (c) J. Raushel, S. M. Pitram, V. V. Fokin, *Org Lett.* **2008**, *10*, 3385. (d) M. Whiting, V. V. Fokin, *Angew. Chem. Int. Ed*. **2006**, *45*, 3157. (e) T. Ryu, J. Min, W. Choi, W. H. Jeon, P. H. Lee, *Org Lett.* **2014**, *16*, 2810. (f) D. Ramanathan, K. Pitchumani, *Eur. J. Org. Chem*. **2015**, 463. (g) N. N. B. Kumar, O. A. Mukhina, A. G. Kutateladze, *J. Am. Chem. Soc*. **2013**, *135,* 9608.
3. J. K. Hexum, R. Tello-Aburto, N. B. Struntz, A. M. Harned, D. A. Harki, *ACS Med. Chem. Lett.* **2012***, 3*, 459.
4. Z.-T. He, B. Tian, Y. Fukui, X. Tong, P. P. Tian, G-Q. Lin, *Angew. Chem. Int. Ed*. **2013**, *52*, 5314.
5. J. Keilitz, S. G. Newman, M. Lautens, *Org. Lett.* **2013**, *15*, 1148.
6. Y. Fukui, P. Liu, Q. Liu, Z.-T. He, N.-Y. Wu, P. Tian, G.-Q. Lin, *J. Am. Chem. Soc.* **2014**, *136*, 15607.
7. C. Clarke, C. A. Incerti-Pradillos, H. W. Lam, *J. Am. Chem. Soc.* **2016**, *138*, 8068.
8. J. Peng, Y. Gao, W. Hu, Y. Gao, M. Hu, W. Wu, Y. Ren, H. Jiang, *Org. Lett.* **2016**, *18*, 5924.
9. N. Okamoto, T. Sueda, H. Minami, Y. Miwa, R. Yanada, *Org. Lett.* **2015**, *17*, 1336.
10. Y.-F. Qiu, X.-Y. Zhu, Y.-X. Li, Y.-T. He, F. Yang, J. Wang, H.-L. Hua, L. Zheng, L.-C. Wang, X.-Y. Liu, Y.-M. Liang, *Org. Lett.* **2015**, *17*, 3694.
11. K. Speck, K. Karaghiosoff, T. Magauer, *Org. Lett.* **2015**, *17*, 1982.
12. B. Xu, U. K. Tambar, *J. Am. Chem. Soc.* **2016**, *138*, 12073.
13. C. Nieto-Oberhuber, P. Pérez-Galán, E. Herrero-Gómez, T. Lauterbach, C. Rodríguez, S. López, C. Bour, A. Rosellón, D. J. Cárdenas, A. M. Echavarren, *J. Am. Chem. Soc.* **2008**, *130,* 269.
14. D. B. Huple, B. D. Mokar, R.-S. Liu, *Angew. Chem. Int. Ed.* **2015**, *54*, 14924.
15. S. Chanthamath, S. Takaki, K. Shibatomi, S. Iwasa, *Angew. Chem. Int. Ed*. **2013**, *52*, 5818.
16. F. Y. Kwong, H. W. Lee, W. H. Lam, L. Qiu, A. S. C. Chan, *Tetrahedron: Asymmetry* **2006**, *17*, 1238.
17. J.-S. Poh, S. Makai, T. von Keutz, D. N. Tran, C. Battilocchio, P. Pasau, S. V. Ley, *Angew. Chem. Int. Ed.* **2017**, *56*, 1864.

18. F. Salvaggio, J. T. Hodgkinson, L. Carro, S. M. Geddis, W. R. J. D. Galloway, M. Welch, D. R. Spring, *Eur. J. Org. Chem.* **2016**, 434.

19. S. P. Chavan, K. P. Pawar, S. Garai, *RSC Adv.* **2014**, *4*, 14468.

20. W. Wang, J. Yang, F. Wang, M. Shi, *Organometallics* **2011**, *30*, 3859.

21. Prepared according to: T. Zhang, Z. Wang, X. Hu, M. Yu, T. Deng, G. Li, H. Lu, *J. Org. Chem.* **2016**, *81*, 4898. Characterization data can be found in: S. L. Pira, E. Boll, O. Melnyk, *Org. Lett.* **2013**, *15*, 5346.

22. V. V. Pavlischuk, A. W. Addison, *Inorg. Chim. Acta.* **2000**, *298*, 97.
